# Zebrafish Bone and General Physiology Are Differently Affected by Hormones or Changes in Gravity

**DOI:** 10.1371/journal.pone.0126928

**Published:** 2015-06-10

**Authors:** Jessica Aceto, Rasoul Nourizadeh-Lillabadi, Raphael Marée, Nadia Dardenne, Nathalie Jeanray, Louis Wehenkel, Peter Aleström, Jack J. W. A. van Loon, Marc Muller

**Affiliations:** 1 Laboratory for Organogenesis and Regeneration, GIGA- Research, University of Liège, B-4000, Liège, Sart-Tilman, Belgium; 2 BasAM, Norwegian University of Life Sciences, Vetbio, 0033 Dep, Oslo, Norway; 3 GIGA & Department of Electrical Engineering and Computer Science, University of Liège, Liège, Belgium; 4 Unité de soutien méth. en Biostatistique et Epidémiologie, University of Liège, B23, Sart Tilman, Liège, Belgium; 5 DESC (Dutch Experiment Support Center), Department of Oral and Maxillofacial Surgery / Oral Pathology, VU University Medical Center & Academic Centre for Dentistry Amsterdam (ACTA), Amsterdam, The Netherlands; 6 ESA-ESTEC, TEC-MMG, NL-2200 AG, Noordwijk, The Netherlands; Ghent University, BELGIUM

## Abstract

Teleost fish such as zebrafish (*Danio rerio*) are increasingly used for physiological, genetic and developmental studies. Our understanding of the physiological consequences of altered gravity in an entire organism is still incomplete. We used altered gravity and drug treatment experiments to evaluate their effects specifically on bone formation and more generally on whole genome gene expression. By combining morphometric tools with an objective scoring system for the state of development for each element in the head skeleton and specific gene expression analysis, we confirmed and characterized in detail the decrease or increase of bone formation caused by a 5 day treatment (from 5dpf to 10 dpf) of, respectively parathyroid hormone (PTH) or vitamin D3 (VitD3). Microarray transcriptome analysis after 24 hours treatment reveals a general effect on physiology upon VitD3 treatment, while PTH causes more specifically developmental effects. Hypergravity (3g from 5dpf to 9 dpf) exposure results in a significantly larger head and a significant increase in bone formation for a subset of the cranial bones. Gene expression analysis after 24 hrs at 3g revealed differential expression of genes involved in the development and function of the skeletal, muscular, nervous, endocrine and cardiovascular systems. Finally, we propose a novel type of experimental approach, the "Reduced Gravity Paradigm", by keeping the developing larvae at 3g hypergravity for the first 5 days before returning them to 1g for one additional day. 5 days exposure to 3g during these early stages also caused increased bone formation, while gene expression analysis revealed a central network of regulatory genes (*hes5*, *sox10*, *lgals3bp*, *egr1*, *edn1*, *fos*, *fosb*, *klf2*, *gadd45ba* and *socs3a*) whose expression was consistently affected by the transition from hyper- to normal gravity.

## Introduction

For many years, the zebrafish has been recognized as an excellent model system for vertebrate developmental biology. More recently, it is increasingly used to study vertebrate physiology, pathology, pharmacology and toxicology [1–5]. Its main advantages are easy maintenance, high fertility, rapid and external development, easy observation of all developmental stages, small size, transparency of the embryos and close contact with surrounding medium (water) allowing easy administration of drugs. In addition, its genome is sequenced and extensively annotated together with well established forward and reverse functional genomics and access to already generated and characterized mutants and transgenic lines of fish (zfin.org).

Skeletal development in zebrafish was first more widely addressed in large scale mutagenesis screening initiatives, resulting in identification of a number of genes required for early formation of the head skeleton [6, 7]. Cranial cartilage is the first skeletal structure to be detected as early as 3 days post-fertilization (dpf), while first calcified intramembranous bone structures start to form at about the same time. Perichondral bone elements slowly build up on the existing cartilage matrix during the following days. In mammals, one of the major genes involved in osteoblast differentiation is *Runx2*. In zebrafish, its ortholog *runx2b* is similarly required for osteoblast differentiation [8] and the onset of osteoblast specific genes [9], such as members of the *dlx* family [10] and *osterix* (*osx*) [11, 12], again with mammalian orthologs. Other expressed genes code for bone extracellular matrix (ECM) proteins osteocalcin (Osc2)[13], collagen10a1a (Col10a1a)[14], Bglap, Spp1 and collagen1a1a (Col1a1a) [9, 15, 16]. The latter is mutated in the *chihuahua* (*chi*) mutant, a model for the human condition of *osteogenesis imperfecta*. Finally, correct calcification of the bone ECM depends on transcellular epithelial calcium uptake through the calcium channel Trpv5/6 [17] and the precise control of phosphate/pyrophosphate homeostasis by the Entpd5 diphosphohydrolase, expressed in osteoblasts [18] together with the widely expressed phosphodiesterase Enpp1 [19]. Taken together, these observations indicate an extensive similarity of the molecular pathways governing bone physiology between teleosts and mammals, validating the zebrafish as a vertebrate model in this field [16, 20–22].

During space flight, human passengers experience profound alterations of their skeletal and muscular system, as well as blood circulatory and immune systems [23–25]. Microgravity is the main differential factor of the environment in space and is probably responsible for the rapid bone loss (osteoporosis) observed in space. Various fish species, such as carp [26], goldfish [27–31], or cichlids [32–39] have been utilized in the past for evaluating the effects of altered gravity. More recently, smaller fishes such as swordtail [37, 40], medaka [41–46] and zebrafish [47–51] have attracted more attention. Most analyses using fishes have concentrated on the impact of altered gravity on graviperception [33, 52], the vestibular system [37, 53, 54] and its involvement in motion sickness [38, 55–57]. Several studies also revealed that general embryogenesis of various organisms is not affected by gravity conditions (review in [46, 49, 50, 58]).

Here, we investigate the effect of increased gravity on the general physiology of zebrafish larvae by using a Large Diameter Centrifuge (LDC) [59] to study whole genome gene expression. We investigate in more detail the effects on head skeleton development and we validate our approach by studying the effects of drug treatments (VitD3 and PTH) known to affect bone formation. Finally, we propose a novel approach to study the effects of microgravity by growing zebrafish in hypergravity for 5 days (from 0–5dpf) before returning them to normal gravity, the Reduced Gravity Paradigm, RGP [60]. The hypothesis for this paradigm dictates that similar effects as observed from the transition going from 1g into micro-g are observed going from a hypergravity level towards a 1g acceleration, a special kind of simulated microgravity or ‘relative microgravity’. However, it may be expected that the magnitude of the effects applying RGP is reduced as compared to the 1g - μg transition.

## Materials and Methods

### Animal procedures

Zebrafish (Danio rerio) were maintained under standard conditions [61] in the GIGA zebrafish facility (licence LA2610359). Briefly, zebrafish (*Danio rerio*) of the AB strain were reared in a recirculating system from Techniplast, Italy at a maximal density of 7 fish/l. The water characteristics were as follows: pH = 7.4, conductivity = 500 μScm-1, temperature = 28°C. The light cycle was controlled (14 h light, 10 h dark). Fish were fed twice daily with dry powder (ZM fish food) adapted to their age and once daily with fresh *Artemia salina* nauplii (ZM fish food). Larvae aged less than 14 days were also fed twice daily with a live paramecia culture. Wild type embryos were used and staged according to [62].

The day before breeding, wild-type adult male and female zebrafish were set up in several breeding tanks, separated by a clear plastic wall. After the light was turned on the next morning, walls are removed, eggs are generated by natural mating and collected from 30 minutes to 2 hours after spawning. After sorting, clean eggs are moved to Petri dishes and incubated at 28°C in E3 medium (5 mM Na Cl, 0.17 mM KCl, 0.33 mM CaCl_2_, 0.33 mM MgSO_4_, 0.00001% Methylene Blue). All protocols for experiments were evaluated by the Institutional Animal Care and Use Committee of the University of Liège and approved under the file numbers 568, 1074, and 1264 (licence LA 1610002).

### Chemicals

Parathyroid hormone (PTH; Merck-Calbiochem, Overijse, Belgium) stock solution (1μg/ml) was prepared in DMSO and stored in aliquots at -20°C. Vitamin D3 (cholecalciferol, VitD3; Sigma-Aldrich, Diegem, Belgium) stock solution (200μl/ml) in DMSO was stored in aliquots at -20°C for maximum one month.

### Chemical treatments

The chemical protocol was inspired by Fleming and collaborators experiments [63]. Larvae at 5dpf were transferred into a 6 well plate (Millipore) containing E3 medium supplemented with the required chemical or vehicle (DMSO) as negative control. The medium was changed every day at the same time. Final concentrations in E3 were at 10ng/ml for PTH and 200ng/ml for VitD3. Each well contained 20 fish in 4ml. They were treated for 1day (n = 50–60 larvae) to perform microarrays and for 5days, from 5 to 9 or 10dpf, to observe the longer-term effects of treatments by different staining (n = 20–30 larvae). Plates were placed into the dark and incubated at 28°C. The larvae were euthanized by tricaine overdose (0.048% w/v) and directly submitted to an RNA extraction at 6dpf (for microarrays) or a 4% para-formaldehyde (PFA; Sigma-Aldrich, Diegem, Belgium) fixation at 6, 9 or 10dpf (for staining).

### Hypergravity experiments in the Large Diameter Centrifuge

A Large Diameter Centrifuge (LDC) was used for hypergravity experiments. It is composed of a central axis linked to 2 perpendicular arms, each arm terminating in 2 opposing gondolas where it is possible to install an incubator containing the samples. The arms provide an 8m diameter for rotation and can provide centrifugal forces of maximum 20g. The zebrafish larvae were incubated in 20 ml E3 in a Petri dish placed in an incubator within a gondola for 3g experiments, and placed either in an incubator on the centrifuge axis (axe) or outside of the centrifuge for 1g controls. In this setting, the medium represents less then 5 mm of water column and thus the 3g acceleration causes an increase in hydrostatic pressure of maximum 0.0015 bar, as compared to the 1bar atmospheric pressure [64].

### Staining methods

Acid-free protocols were adapted [65] to perform Alcian blue (8 GX Sigma-Aldrich, Diegem, Belgium) staining of cartilage structures and Alizarin red S (Sigma-Aldrich, Diegem, Belgium) staining of calcified structures. At 6, 9 or 10dpf, the larvae were fixed in 4% PFA for 2h at room temperature and rinsed several times with PBST.

Cartilage was stained overnight in 10 mM MgCl_2_, 80% EtOH and 0.04% Alcian blue. The larvae were washed in different concentrations of ethanol (80%, 50%, 25%) to remove excess staining. Pigmentation was bleached in a H_2_O_2_ solution (H_2_O_2_ 3%, KOH 0.5%) and finally the larvae were rinsed 3 times in a solution of 25% glycerol / 0.1% KOH and 50% glycerol, 0.1% KOH and finally stored in this solution at 4°C.

During acid-free bone structure staining with Alizarin red, bleaching was performed immediately after fixation, before the staining. After the bleaching, long rinses (at least 20min each) in a 25% glycerol, 0.1% KOH solution are necessary to prevent the fading of the staining. The larvae are stained in a 0.05% Alizarin red solution in water for 30min in the dark on low agitation, rinsed in a 50% glycerol, 0.1% KOH solution to remove excess staining and kept at 4°C in the same solution.

Images of stained larvae (n = 20–30 larvae) were obtained on a binocular (Olympus, cell B software).

### Image analysis

**Fig 1:**
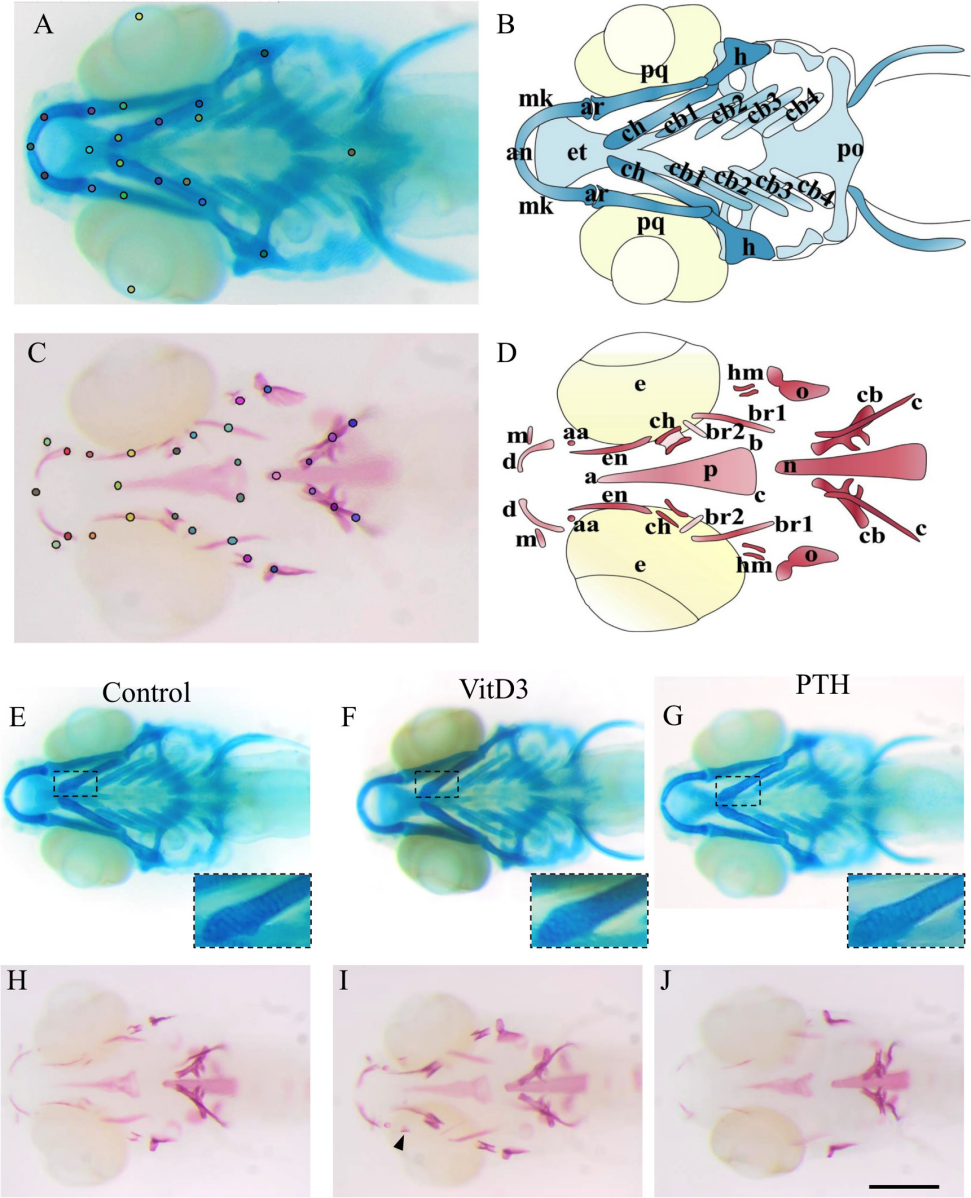
(A-D) Cartilage and bone elements of the head skeleton in 10dpf zebrafish. (A) Alcian blue staining of head cartilage representing the landmarks used for morphometry. (B) Schematic representation of the different head cartilage elements. anterior limit (an), articulation (ar), ceratobranchial pairs 1 to 4 (cb1-4), ceratohyal (ch), ethmoid plate (et), hyosymplectic (h), Meckel's cartilage (mk), palatoquadrate (pq), posterior limit (po). (C) Alizarin red staining of cranial bones representing the landmarks used for morphometry. (D) Schematic representation of the different cranial bone elements with 29 landmarks used for chemicals treatments and 15 landmarks for the 3g and the relative-hypergravity. The 15 landmarks are anguloarticular (aa), anterior (an), branchiostegal ray1 (br1), entopterygoid (en), maxilla (m), notochord (n), opercle (o), parasphenoid (p). Note that the parasphenoid is a triangular bone defined by its anterior summit (a) and two posterior summits (b,c). The 29 landmarks include the 15 named before with branchiostegal ray2 (br2), cleithrum (c), ceratobranchial 5 (cb), ceratohyal (ch), dentary (d), hyomandibular (hm). (**E-J**) **10dpf zebrafish larvae after 5 days chemical treatments.** (E-G) Alcian blue staining of cartilage. (H-J) Alizarin red staining of bone. (E,H) Controls in DMSO. (F,G) no significant effect of, respectively VitD3 and PTH on cartilage development, nor on chondrocyte shape or size (inlays showing close-up). I: increase of bone development after VitD3 treatment. (J) decrease of bone development after PTH treatment. Ventral views, anterior to the left, (E-J) scale bar = 250μm.

Image analysis was performed on the pictures of larvae stained with Alcian blue for cartilage or Alizarin red for bone. Individual cartilage and bone elements were identified according to [10, 15, 66–68]. For morphometric analysis, images were uploaded into the CYTOMINE environment [69] and manually annotated by positioning 21 landmarks for larvae stained for cartilage (Fig 1A) as previously defined in the CYTOMINE ontology. 29 landmarks were placed for larvae stained for bone in hormonal treatments (Fig 1C), of which 15 were selected for the hypergravity experiments. The program then defines the positions of all selected landmarks and computes all the distances (in pixels) and angles (in radian) of all the possibilities between two points of interest. These data were exported into an Excel file and a selection of interesting measures was conducted by performing principal component analysis on data obtained from differently treated larvae to identify invariable or redundant measures. The measures selected were: for cartilage (Alcian blue): Anterior to Ethmoid plate, Anterior to Posterior, Articulation down to Articulation up, Ceratohyal ext. down to Ceratohyal ext. up, Ceratohyal ext. down to Ceratohyal int. down, Ceratohyal ext. up to Ceratohyal int. up, Ethmoid plate to Posterior, Hyosymplectic down to Hyosymplectic up; and for bone (Alizarin red): Anguloarticular down to Anguloarticular up, Anterior to Notochord, Anterior to Parasphenoid a, Branchiostegal ray 1 down to Branchiostegal ray 1 up, Entopterygoid down to Entopterygoid up, Maxilla down to Maxilla up, Opercle down to Opercle up, Parasphenoid a to Parasphenoid b, Parasphenoid b to Parasphenoid c, area of the parasphenoid triangle: parasphenoid a, b, and c, and finally the angles between parasphenoid a and b, a and c, b and c.

Statistics were performed using GraphPad Prism5. A t-test was used for control versus treatment experiments, while a one way ANOVA was used for multiple comparisons.

Morphometric analysis did not inform about the extent of ossification within each larva. Thus, a systematic structure analysis was generated. Each bone structure was classified based on the progress of development into one of the four following categories: absent, early ossification, advanced ossification and over ossification. When values were considered as quantitative, comparison between two groups (control versus chemical treatment or hypergravity in 1g>3g) was assessed by a Student t-test, while comparison between different treatments ("relative microgravity" experiment) was assessed by an analysis of variance (ANOVA). A contingency table considered ordinal values distributed among the 4 classes (from absent to over ossification) or only 3 classes when one class was not present in the sample. Association between classes and treatment was assessed by X² test and by an ordinal logistic regression and the odds ratio (OR). The "relative microgravity" experiment was analyzed in addition by grouping the 3g, 3g>1g and 3g>axe versus the 1g sample.

Statistical analyses were performed using the Statistica Software (version 10). Results were considered statistically significant at the 5% critical level (p < 0.05).

### RNA extraction and reverse transcription

Larvae at 6dpf, after 24h treatment, were used for the RNA extraction. Total RNA was extracted of 60 larvae per experiment using Trizol, followed by the RNeasy Mini kit (Qiagen, Hilden, Germany) according to the manufacturer’s instructions and conserved at -80 degrees using. They were treated with Rnase-free Dnase Set (Qiagen, Hilden, Germany). After extraction, the quality and concentration of total RNA was evaluated by electrophoresis on capillary gel and the ratio of absorbance at 260/280nm by spectrophotometer (Bioanalyzer 2100, Agilent Technologies, Diegem, Belgium). Synthesis of cDNA was performed from 1μg of total RNA, which was reverse transcribed (Transcriptor iScript cDNA Synthesis Kit, Bio-Rad, Nazareth, Belgium) according to the manufacturer’s instructions.

### Real Time-PCR

Gene-specific oligonucleotide primers were designed using Primer3 software to span exon-exon junctions to avoid detection of genomic DNA contamination (see S1 Table for primer sequences) and synthesized by Eurogentec (Seraing, Belgium) or Integrated DNA Technology (Leuven, Belgium). cDNA was used as template for quantitative Real-Time PCR with the SensiMix SYBR Kit (Bioline, London, UK), containing Sybr green. Reactions were performed on an Applied Biosystems 7900 HT sequences Detection System (Applied Biosystems, Foster City, CA) using the onboard software (SDS 2.4). Purity of the amplicons was checked by melting curves at the end of each reaction. Ct values were exported from the onboard software as a text file and imported into a customized Microsoft excel spreadsheet. 1 μl of the RT reaction (1/20 of the total cDNA) was added to 1X SYBR green master mix (Bioline, London, UK), 150 nmol of each primer in 15 μl total volume. Samples were run in triplicate in optically clear 384-well plates (ABgene), sealed with optical adhesive film (Applied Biosystems). "No template" controls were run for all reactions, and all RNA preparations were subjected to sham reverse transcription to check for the absence of genomic DNA amplification. The relative transcript level of each gene was obtained by the 2^-ΔΔCt^ method [70] and normalized relative to the *gapdh* (glyceraldehyde-3-phosphate deshydrogenase) housekeeping gene chosen from a panel of 3 genes (*gapdh*, *ef1-a*, *ß-actin*) as the most stably expressed throughout our experiments (not shown). Data from biological replicates were averaged and shown as mean normalized gene expression ± SD.

Cycling parameters: 50°C x 2 min, 95°C x 10 min, then 40 cycles of the following 95°C x 15 s, 62°C x 20 s. A melting temperature-determining dissociation step was performed at 95°C x 15 s, 60°C x 15 s, and 95°C x 15 s at the end of the amplification phase.

### Microarray expression experiments

For microarray expression analysis, four replicates from each treatment (control and drug or gravity treatment) were analyzed in 2+2 dye-swap hybridizations. One μg total RNA was linearly amplified one round and labeled, using Amino Allyl Message Amp II aRNA amplification kit (Ambion-Life Technologies, Gent, Belgium) as previously described [71]. Five μg of the resulting antisense RNA (aRNA) from the exposed and control groups was labeled either with Cy3-dUTP or Cy5-dUTP (GE Healthcare Bio-Sciences AB, Uppsala, Sweden). The labeled targets were examined for amplification yield and incorporation efficiency by measuring the aRNA concentration at 260 nm, Cy3 incorporation at 550 nm, and Cy5 at 650 nm using Nanodrop (Thermoscientific, Wilmington, DE, USA). A good aRNA probe had a labeling efficiency of 30–50 fluorochromes every 1000 bases. One to 5 μg of each labeled aRNA target was mixed, 9 μl 25× fragmentation buffer (Agilent Technologies, Diegem, Belgium) added, and the final volume adjusted to 225 μl with RNase-free H2O followed by incubation for 30 min at 60°C. The hybridization solution was prepared by adding 220.5 μl of 2× hybridization buffer (Agilent Technologies, Diegem, Belgium) and 4.5 μl sonicated herring sperm DNA (10 μg/μl; Promega, Madison, WI, USA) to the labeled target aRNA. Microarray slides (4x44K zebrafish V2 or V3, Agilent Technologies, Diegem, Belgium) were prehybridized at 42°C, 60 min using 0.1% bovine serum albumin (BSA) Fraction V, 5× SSC, and 0.1% sodium dodecyl sulfate (SDS). Hybridization was performed at 60°C in 16 h using gasket slides, hybridization chamber, and oven (Agilent Technologies, Diegem, Belgium) according to Agilent 60-mer oligo microarray processing protocol. Microarray slides were then washed 3 × 5 min in 0.5 × SSC, 0.01% SDS (first wash at 42°C and next two at room temperature). Finally, slides were washed 3 times in room temp with 0.06× SSC and dried immediately with centrifugation at 800×g for 1 min.

Microarray slides were scanned using a GenePix 4000B (Axon instrument, Foster City, CA). Scanning was performed at a level just before saturation of several spots. Raw data generated from Genepix were imported into the Bioconductor package LIMMA and corrected for background [72]. For within-array and between-array normalization, print tip Loess and scale were used, respectively [72]. An empirical Bayes moderated t-test [72, 73] was applied to detect differently expressed genes across treated and control samples. The p values were corrected for multiple testing using the Benjamini–Hochberg (BH) [74] method and p-values <0.1 were selected as differently expressed genes. The generated gene list was further filtered for genes with low intensity and with small changes in expression. In the averaged normalized MA-Plot, the majority of genes were clustered in between M values of ±0.4 (fold change ±1.3) and selected to be threshold criteria for differently expressed gene list. The VitD3 data were obtained on a SureScan Dx instrument (Agilent Technologies, Diegem, Belgium) and analyzed using the GeneSpring software (Agilent Technologies, Diegem, Belgium) by applying the same settings.

Raw data and complete lists of analyzed data are publicly available at Arrayexpress (https://www.ebi.ac.uk/arrayexpress/) under the accessions: E-MTAB-3285, E-MTAB-3286, E-MTAB-3289, and E-MTAB-3290.

### Ingenuity Pathway Analysis

For pathway and biological function analysis of significantly differently expressed genes, Ingenuity pathway analyses (IPA, QIAGEN Redwood City; http://www.ingenuity.com) were used. The lists with differently expressed genes generated by the microarray analysis were translated into mammalian (human, mouse, and rat) orthologs using the Unigene & Gene Ontology Annotation Tool and uploaded to IPA. The IPA software is an online exploratory tool with a curated database for over 20,000 mammalian genes and 1.9 million published literature references. IPA’s database together with EntrezGene, Gene Ontology, etc., integrates transcriptomics data with mining techniques to predict and build gene networks, pathways, and biological function clusters. The output results are given scores and p-values that are computed based on the number of uploaded genes in the cluster or network and the size of the network or cluster in the Ingenuity knowledge database. Fisher’s exact test is used to determine the probability that each associated biological function is due to chance alone. Scores for IPA networks are the negative logarithm of the p-value, indicating the likelihood of the focus genes (genes uploaded to IPA) in a network being found together due to random chance. Scores of 2 or higher have at least a 99% likelihood of not being generated by chance alone.

## Results

### Effects of drug treatments on head skeletal formation

To characterize in detail the process of cartilage and bone formation in zebrafish, we first wanted to examine the effects of chemical treatments known to affect skeletal development. Treatment of zebrafish larvae with vitamin D (VitD3) was previously shown to result in enhanced bone formation, while continuous treatment with parathyroid hormone (PTH) led to decreased bone formation [63]. We decided to confirm and extend these findings by comparing the effects on skeletal formation to those on gene expression.

VitD3 and PTH treatments were performed continuously from 5dpf to 10dpf. Control and treated larvae were stained by Alcian blue for cartilage extracellular matrix (ECM) and with Alizarin red to detect the calcified bone matrix. At this stage, the head cartilage is well formed and a complete set of cartilage elements is observed (Fig 1A and 1B). In contrast, although ossification begins at 3dpf and the first bone structures are visible at 5dpf, the bone skeleton continues its formation until 30dpf [68]. Nevertheless, at 10dpf, a number of bone elements are observed in the head region, the first vertebral centrae are formed, while others only begin to be calcified (for example the branchiostegal ray2) (Fig 1C and 1D).

In three independent experiments, 27–29 ventral view images of Alcian blue- or Alizarin red-stained larvae were obtained. After 5days of VitD3 or PTH treatment, cartilage stays unchanged as compared to the control by general observation. The structures are well formed, complete with the glycosaminoglycans present in the cartilage matrix judging from the similar staining intensity (Fig 1E–1G). In a close-up view (Fig 1E–1G, inlays), no difference could be observed in cell shape or size between the different treatments. Considering bone calcification, a general observation revealed a clear increase of bone development upon VitD3 treatment (Fig 1I). Some structures appear in advance, such as the retroarticular (Fig 1I arrowhead) bone and the preopercular (not shown) bone, while some other structures are thicker such as the dentary or the ceratohyal, or longer such as the branchiostegal ray2. Nevertheless, the general morphology was unchanged. In contrast, continuous PTH treatment led to a general decrease of bone formation and to a complete absence of some structures, such as the anguloarticulars and branchiostegal ray2 (Fig 1J).

Based on these images, we applied two complementary approaches to obtain a more objective qualitative and quantitative description of the skeleton. The first one is a morphometric approach that evaluates the general aspect of the head skeleton by measuring the distances between and the relative position of all detected bone elements. The images were introduced into the CYTOMINE software (see Materials and Methods, [69]) and each image was annotated by positioning specific landmarks representing the different skeletal elements. For larvae stained for cartilage, 21 landmarks were defined (Fig 1A), while 29 points of interest were positioned within the Alizarin red-stained bone skeleton (Fig 1C). In these pictures, we consider the head separated horizontally in 2 parts. Some structures are unique and located on the symmetry axis, while others are paired and localized symmetrically, such as the dentary, maxilla, entopterygoid, and hyosymplectic. To facilitate recognition, these were labelled “up” and “down”. The software then computes the distances between selected landmarks and the angles formed by lines drawn between selected points.

**Fig 2:**
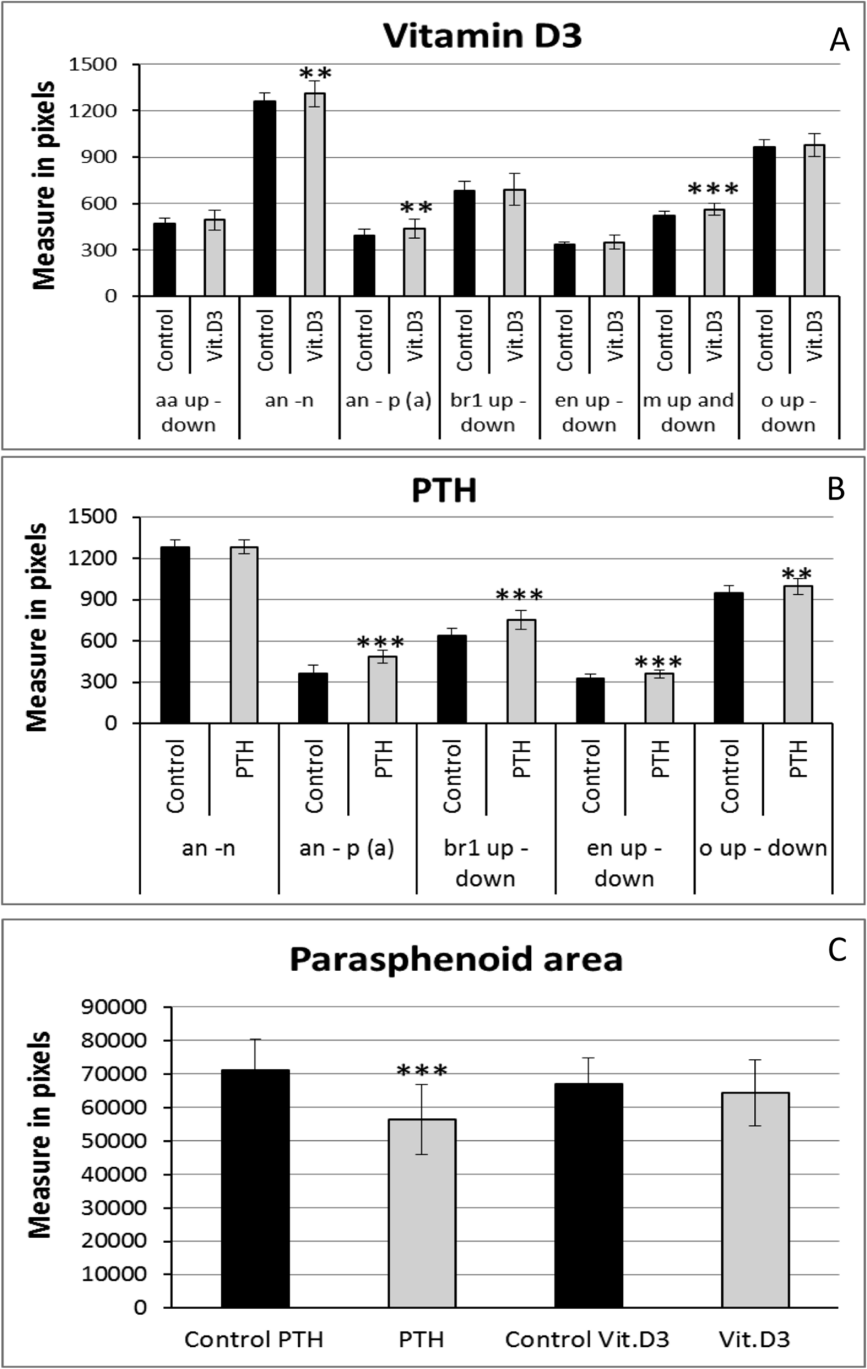
Morphometric analysis results of bone matrix staining after 5 days chemical treatments. The distances are measured in pixels. Mean ± SD and t-test analysis were calculated for each measure on at least 20 individuals. * *p <* 0.05, ** *p <* 0.01 and ***p < 0.001. (A) Distances after VitD3 treatment. (B) Distances after PTH treatment. (C) Area of the parasphenoid bone results after 5 days PTH or VitD3 treatment. Abbreviations as in Fig 1. **A)** Analysis of the bone skeleton after VitD3 treatment revealed a significant increase of the distance between maxillae (m), consistent with a broader jaw as already observed by cartilage morphometry. The length of the head skeleton is also increased upon VitD3 treatment with a longer distance between the anterior part of the head (an) and the notochord (n), and between an and the parasphenoid (p) bone. Other measures are not significantly modified (A, C). B) PTH treatment caused an increase of the distance between the anterior part of the head and the summit “a” of the parasphenoid, mainly due to a significant decrease of the size of the parasphenoid (p) (C). Some structures are missing, such as the anguloarticular (aa), branchiostegal ray2 (br2), ceratohyal (ch) and/or maxilla (m). However, a significant broadening of the posterior head skeleton is revealed by the increased distance between left and right ("up" and "down") branchiostegal rays1 (br1), entopterygoids (en) and also the opercula (o) (B).

Morphometric analysis in VitD3-treated larvae cartilage revealed an increase of the distance between articulation (ar) "up" and "down", leading to a broader jaw as compared to untreated animals, while all the other distances remained unchanged (S1A and S1C Fig). Morphometric cartilage analysis of larvae treated with PTH for 5 days revealed an increase in length of the ceratohyal cartilages (ch, S1B Fig). Analysis of the bone skeleton after VitD3 treatment revealed a significant increase of the distance between maxillae (m, Fig 2A), consistent with a broader jaw as already observed by cartilage morphometry. The length of the head skeleton is also increased upon VitD3 treatment with a longer distance between the anterior part of the head (an) and the notochord (n) or the parasphenoid (p). Other measures are not significantly modified (Fig 2A and 2C). PTH treatment led to a significant decrease of the size of the parasphenoid (p, Fig 2C). Some structures are missing, such as the anguloarticular (aa), branchiostegal ray2 (br2), ceratohyal (ch) and/or maxilla (m) and a significant broadening of the posterior head skeleton is revealed by the increased distance between left and right ("up" and "down") branchiostegal rays1 (br1), entopterygoids (en), and opercula (o) (Fig 2B).

**Fig 3:**
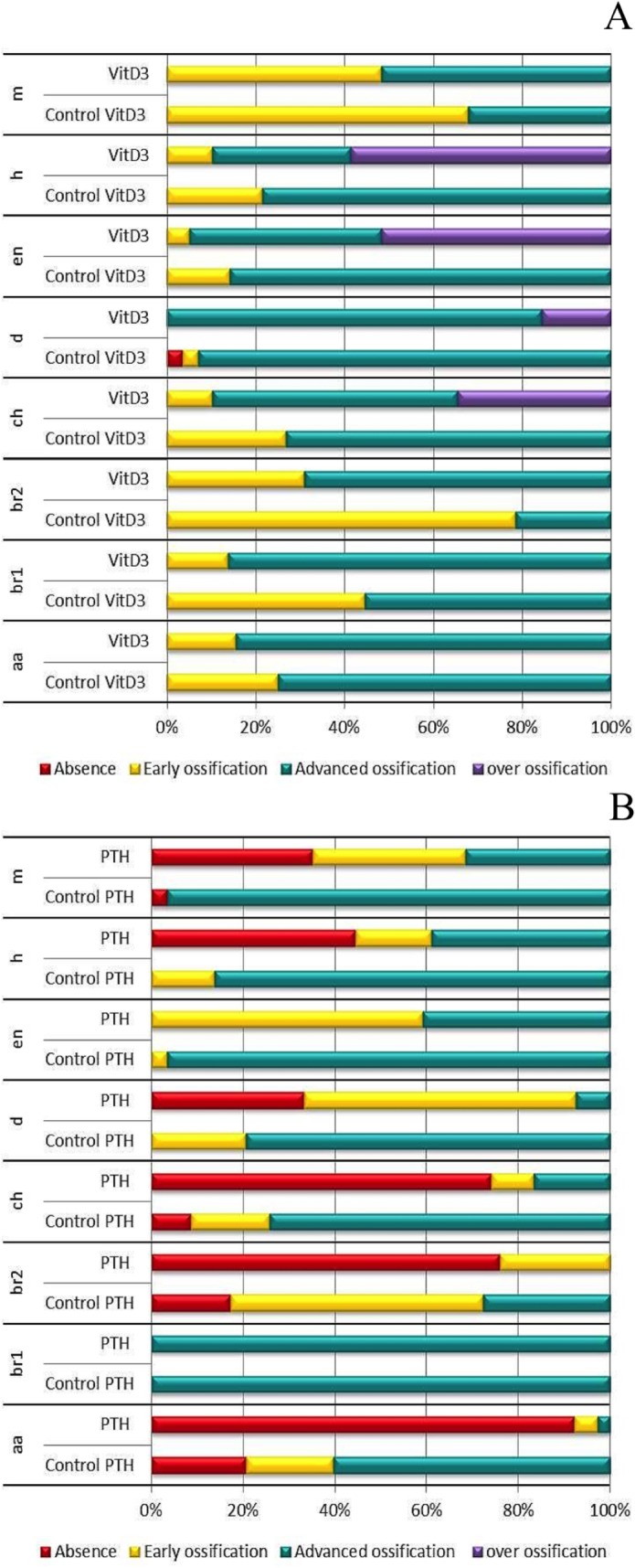
Extent of bone formation in 10dpf larvae after 5days chemical treatments. Bone development is classified for each element into different categories: Absent (no structure present; red), early ossification (beginning of the bone ossification; yellow), advanced ossification (the structure is present and already developed as the control; green) and over ossification (the structure is more developed compared to the control; purple). Cumulated frequencies in % are represented for each element. As no significant difference was observed for paired structures between left and right (up and down), their scores have been combined. Statistical analysis was performed by X² of Pearson and a logistic regression. (A) Cumulated frequency after 5days VitD3 treatment. To obtain this, values were attributed to each element according to its category and added up for each larva: 0 for absent, 1 for early, 2 for advanced, and 4 for over ossification (B) Cumulated frequency after 5days PTH treatment.

The second approach consists in the evaluation of the intensity and progression of bone formation of the different bone structures, and their level of ossification. In each image, every bone structure is assigned a score, ranging from absent (red), early ossification (yellow), advanced (green) or over-ossified (purple) in comparison to a typical image of a control larva of the same age. The distribution of the scores obtained for the different elements in VitD3- or PTH-treated larvae and the corresponding controls is shown in Fig 3 and the results of the statistical analysis are given in S2 and S3 Tables.

After 5 days VitD3 treatment, all the structures are present and some are over-ossified like the hyomandibular, the entopterygoid, the dentary and the ceratohyal bones. Early (delayed) ossification is decreased for all the structures shown, as compared to controls, while advanced ossification increased in the maxilla, branchiostegal ray1, branchiostegal ray2 and anguloarticular (Fig 3A). Statistical analysis (S2 Table) reveals that only the anguloarticular and the maxilla up do not change significantly in this condition. All the other structures (br1, br2, m down, ch, d, en, hm) are significantly increased, with the hyomandibulars, entopterygoids and ceratohyals displaying the most drastic effect. These results confirm a very significant positive effect of VitD3 treatment on bone formation.

PTH treatment resulted in nearly opposite effects to VitD3. Only the entopterygoid and the branchiostegal ray1 are present in each fish (Fig 3B) with the branchiostegal ray1 unaffected and the entopterygoid displaying 60% of early ossification in PTH-treated larvae compared to 3,45% in controls. All the other structures were absent in at least 20% of the total 27 fish analyzed. The strongest effect was seen in the anguloarticular bone with 94% of absence compared to 21% absence, 19% early ossification and 60% of advanced ossification in the controls. Specific statistical analysis confirmed that PTH treatment significantly (p<0,001) reduced nearly all the structures except branchiostegal ray1 (S3 Table).

To obtain a global score describing the head skeleton in the different conditions, the individual structure scores in each image were added up and a mean global score was obtained showing that VitD3 treatment significantly increases bone development (from a score of 26±3 in the controls to 33±4 in the VitD3 treatment), while PTH treatment significantly decreases ossification to approximately half of untreated control (from a score of 27± 4 to 13±5,5).

In summary, these complete image analyses reveals that VitD3 treatment conserves the general skeletal morphology, but leads to a longer head and a larger jaw. Bone calcification is stronger for most elements, and some elements calcify earlier. In contrast, PTH treatment conserves the general cartilage morphology except for an increased length of the ceratohyal. In bone, PTH treatment leads to a general decrease of ossification. Some structures are missing and the parasphenoid is significantly decreased.

### Modification of gene expression upon drug treatment

To gain deeper insight into the molecular mechanisms involved in the observed skeletal modifications, we analyzed the expression of several genes selected for their known function in bone formation. One class of genes codes for structural proteins such as collagens (Col1a1, Col1a2, Col10a1a) or bone specific ECM proteins such as secreted acidic cysteine rich protein (Sparc, previously named osteonectin or Osn), secreted phosphoprotein 1(Spp1, previously named osteopontin or Osp) and bone gamma-carboxyglutamate protein (Bglap, previously named osteocalcin or Ocn). The second class of interest consists of those genes coding for factors involved in regulation of cartilage and bone differentiation, including the *pth1a* gene coding for PTH as well as transcription factor genes *dlx5a*, *dlx6a*, *runx2b* and *osx*.

**Fig 4:**
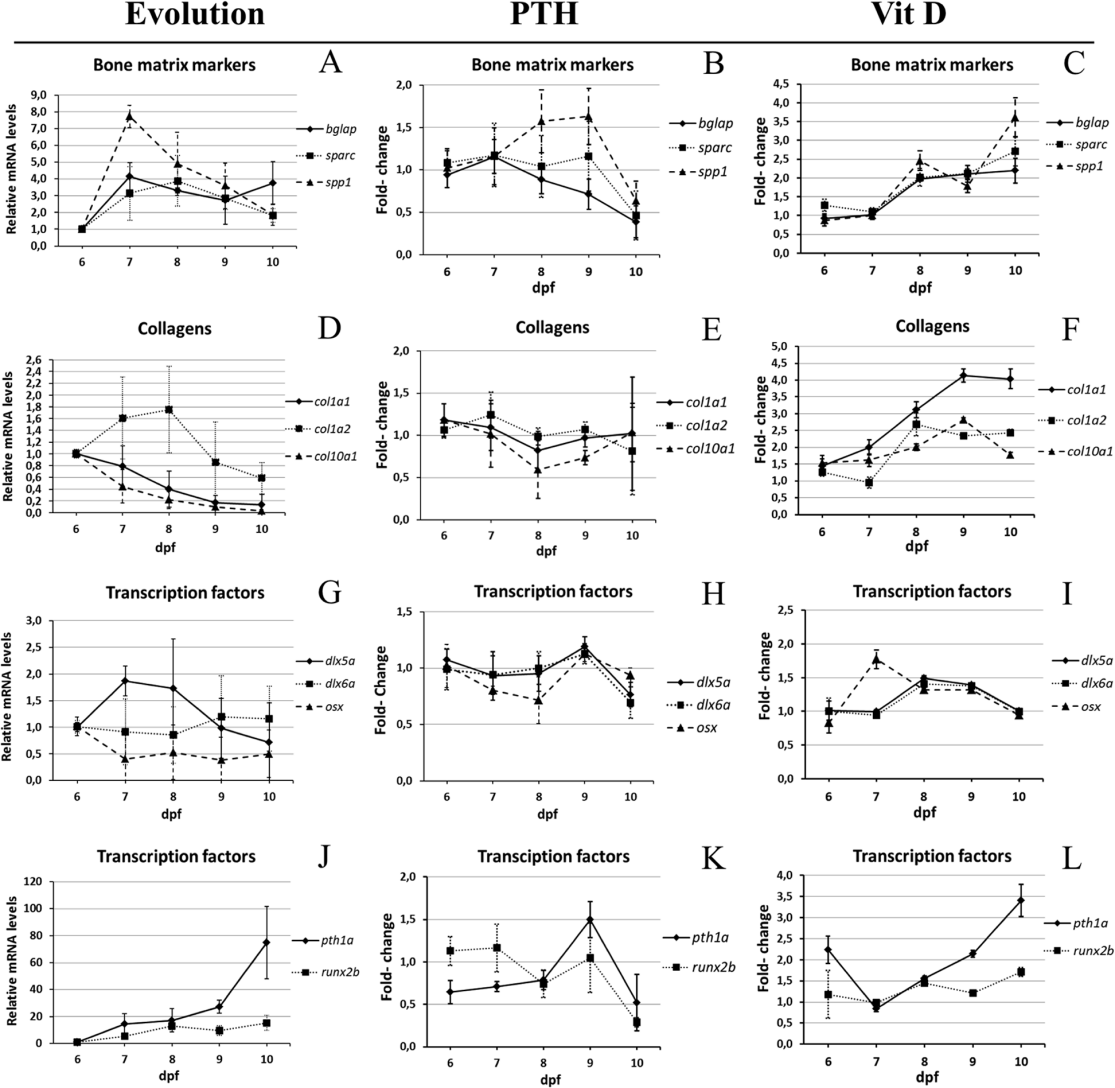
Expression of bone-specific genes during development between 6 and 10dpf. (A,D,G,J) Specific mRNA levels at 6dpf relative to the *gapdh* house-keeping gene were used as reference, and then compared to the corresponding level in larvae of different age. (E-F,H-I,K-L) Specific mRNA levels in treated larvae were determined relative to the *gapdh* reference house-keeping gene and then compared to the corresponding level in untreated controls of the same age. (A-C) Bone matrix markers *bglap*, *sparc*, *spp1*. (D-F) Collagens *col1a1*, *col1a2*, *col10a1a*. (G-I) Transcription factors *dlx5a*,*dlx6a* and *osx*. (J-L) Parathyroid hormone *pth1a* and transcription factor *runx2b*.

We first decided to follow the expression of these genes during the 6–10dpf period in untreated animals, using the glyceraldehyde-3-phosphate dehydrogenase (*gapdh*) house-keeping gene as reference (selected from 3 candidate housekeeping genes, see Materials and Methods). Compared to their expression at 6dpf, we observe an increase of *sparc*, *bglap*, *spp1* and *col1a1* at 7dpf, followed by a decrease at 8dpf for *sparc*, *bglap* and *spp1*, while the *col1a1* gene peaked at 8dpf and decreased its expression at later stages (Fig 4A and 4D). The *pth1a* gene expression strongly increased 76-fold during the 6–10dpf period, while *runx2b* displayed a 10-fold increase. The transcription factor gene *dlx5a* displayed an expression peak at 7 and 8dpf and decreased after that, while *dlx6a* was unaffected and *osx* surprisingly revealed a 2-fold decrease from 6 to 7dpf (Fig 4G and 4J).

We then investigated the modulation of expression of these genes during drug treatment starting at 5dpf. Compared to untreated controls, VitD3 treatment led to a clear and significant increase in expression of all the structural protein genes: *sparc*, *bglap*, *spp1*, *col1a1* and, to a lesser extent *col1a2* and *col10a1a* (Fig 4C and 4F). These results correlate well with the observed increase in bone calcification observed at 10dpf. Among the regulatory factor genes, only *pth1a* revealed a strong up-regulation that increased during the treatment, while *dlx5a* and *dlx6a* were transiently induced at 8 and 9dpf. Finally, *runx2b* displayed a weak but significant increase up to 1.5-fold at 10dpf, and *osx1* was only transiently induced 2-fold at 7dpf (Fig 4I and 4J).

On the other hand, relative to untreated controls, PTH treatment resulted in a transient increase of *spp1* at 8–9dpf, while *sparc*, and *bglap* were unchanged before a decrease at 10dpf (Fig 4B). Surprisingly, no significant effect of PTH treatment was observed on the expression of the collagen genes (Fig 4E). Among the regulatory factors, *osx* expression remained constant, while *pth1a*, *dlx5a*, *dlx6a* and *runx2b* declined at 10 dpf (Fig 4H and 4K). Taken together, these observations are consistent with the observed decrease in bone matrix calcification at 10dpf.

### Whole genome analysis of gene expression modulation by drugs

To obtain a global view of the physiological changes caused by PTH and VitD3 treatment, we performed a microarray whole genome expression analysis. We compared 6dpf control larvae to larvae treated between 5dpf and 6dpf with the corresponding compounds, in order to capture early regulatory events rather then secondary regulations leading ultimately to the observed modulations of bone formation at 10dpf.

Four independent experiments were carried out and total RNA was extracted from control and VitD3-treated 6dpf larvae. A complete list of genes affected more than 1.3-fold (log2 fold change 0.4) by VitD3 treatment is given in S4 Table (p-value<0.1). Six genes were selected from the list for validation by RT-qPCR, which demonstrated the reliability of the microarray data (Table 1). Confirming that the VitD3 pathway was indeed activated, the most highly induced gene is *cyp24a1*, encoding a member of the cytochrome P450 superfamily of enzymes involved in the degradation of 1,25-dihydroxyvitamine D3. Modulation of the insulin pathway is indicated by the significant induction of *igfbp1* and *igf2*. According to Ingenuity Pathway Analysis (IPA; Materials and Methods), other biological functions that were affected by vitamin D treatment (S5 Table) are related to lipid, small molecule, amino acid, carbohydrate and drug metabolism, followed by organismal and cardiovascular system development. A striking feature of the affected genes list is the abundance of genes involved in molecular transport, from ion channels to ATP-dependent pumps (S4 Table), consistent with a profound adaptation to the changes in metabolism that were also previously observed [75–77]. Among the transcription regulatory factors, we note the decreased expression of *ppara* and of *foxo3*, involved in lipid metabolism, as well as *fosb* and *twist1*, while *klf11* and *klf13* were significantly induced (S4 Table). As these experiments were performed using mRNA from the entire larvae, we attempted to focus on individual organ systems by filtering the affected gene set against available databases of genes involved in muscle or cartilage/bone function (GO annotation of human gene orthologs using IPA knowledge base). A network of regulatory interactions could be constructed, comprising genes common to both systems and genes specific for each organ (S2 Fig). Major hubs, such as the protooncogene *MYC* controlling cell proliferation, components of the insulin-like pathway such as *IGFBP1* and *IGF2*, or the cytokine receptor regulator *SOCS1* are common to both systems. Specific to muscle, regulators such as *PPARA* or *FOXO3* are down-regulated, while STAT3, mediating the cytokine receptor response, is up-regulated. Interestingly, muscle structural genes such as *TTN* (Titine) are inhibited. Other affected genes are bone-specific transcription factors, such as *ATF4* and *FOSB*, a member of the WNT pathway (*WNT3*) or the carbohydrate (glycoprotein)-binding protein *LECT1* (Lectin1).

**Table 1:** Comparison of fold change values from the microarray dataset with those observed by RT-qPCR for VitD3 and PTH treatment.

	VitD3				PTH			
	microarray		RT-PCR		microarray		RT-PCR	
Gene	Fold Change	p-value	Fold Change	p-value	Fold Change	p-value	Fold Change	p-value
*cad*	1.424	0.094	2.017	< 0.001				
*cyp24a1*	8.938	0.005	10.969	< 0.001				
*igfbp1*	3.782	0.004	5.250	< 0.001				
*socs1*	0.355	0.066	0.447	< 0.001				
*slc26a3*	0.525	0.028	0.654	< 0.001				
*slc6a18*	0.726	0.029	0.895	0.002	0.203	0.066	0.883	0.035
*fgf4*	0.520	0.110	0.777	< 0.001	0.450	0.079	0.831	< 0.001
*mcph1*					1.934	0.056	1.130	0.026
*ndrg2*					1.545	0.060	1.101	0.036
*rxra*					1.990	0.076	1.247	< 0.001
*nrbp2*					2.514	0.056	1.210	0.010

The fold change and statistical significance (p-values) are given from the microarray data and the RT-qPCR confirmation experiments. The data for the genes selected for confirmation of microarray results, respectively for VitD3 or PTH, are shaded in grey. s*lc6a18* and *fgf4* were chosen for their regulation by PTH and the results for VitD3 regulation are also shown

**Fig 5:**
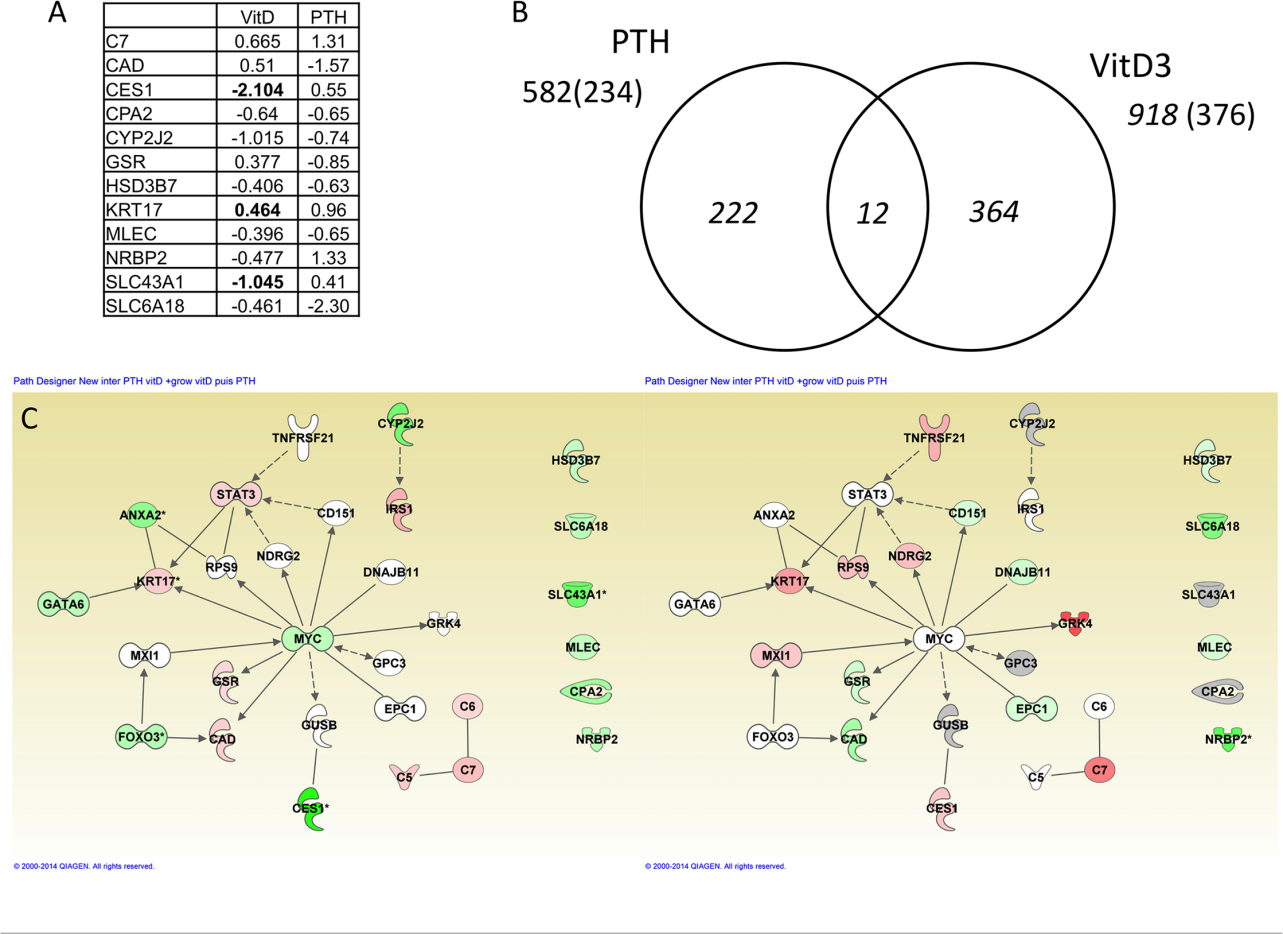
Comparison of genes affected after PTH or VitD3 treatment between 5–6dpf. (A) List of common genes and their respective log2(fold change) in the two conditions. (B) Comparison of the number of genes affected by PTH or VitD3 treatment. The number of probes resulting in different hybridization signals is given, with the numbers in parenthesis and the graph showing the numbers of IPA-annotated genes. (C) Network constructed using the common genes and extended using the genes affected in one of the two conditions. The color overlay indicates the fold change after VitD3 (left) or PTH (right) treatment. Genes up-regulated (red), down-regulated (green), (*) indicates that the gene is represented by two or more probes on the microarray.

PTH treatment between 5dpf and 6dpf resulted in less modulation of gene expression (S6 Table). Six genes were selected from the list to include up- and down-regulated genes for independent confirmation of the microarray expression results by RT-qPCR (Table 1). Interestingly, we observed a decrease (2.5-fold) in the expression of the endogenous *pth1a* gene (*PTH* in S6 Table), thus confirming the previous RT-PCR results (Fig 4K) and suggesting that the PTH treatment was effective, as the larvae exerted a compensatory response by decreasing endogenous PTH production. In rat and human osteoblastic cells, PTH receptor mRNA was shown to be down-regulated upon PTH treatment [78, 79], in contrast we observe a significant induction (1.9-fold) of PTH receptor (*pth1rb*), suggesting more complex regulatory networks in using an *in vivo* model as opposed to *in vitro* cultures. Additional affected genes are the repressed *cyp21a2* and *hsd3b7*, indicating a decrease in steroid degradation. The increased expression of *rxra* nuclear receptor mRNA (S6 Table) contrasts with the observed VitD3 effects (S4 Table), where pathways involving Rxra and its nuclear receptor dimerization partner *Ppara* were down-regulated (S4 and S5 Tables). IPA comparison between PTH and VitD3 effects reveals that, unlike the general metabolic effects exerted by VitD3, the most prominent biological functions affected by PTH treatment were related to cell development, signaling and embryonic development (S7 Table). The most highly developmentally affected systems were hematopoiesis and the skeletal, muscular and cardiovascular systems. Further analysis revealed up-regulation of a number of genes involved in or dependant on calcium metabolism, such as calreticulin (*CALR*), integrin α9 (*ITGA9*), calcitonin receptor like (*CALCRL*) or arginine vasopressin receptor a1 (*AVPR1A*). Comparison of the genes affected by the two hormones exerting opposite effects on bone formation, VitD3 and PTH, revealed only 12 genes in common (Fig 5A and 5B). Using these 12 genes allows building a regulatory network around the protooncogene *MYC* and containing several genes that are differentially regulated in these two conditions (Fig 5A and 5C), suggesting opposing effects on mitochondrial (GSR), pyrimidine (CAD) or lipid metabolism (CES1).

### Effects of hypergravity on bone and general development

**Fig 6:**
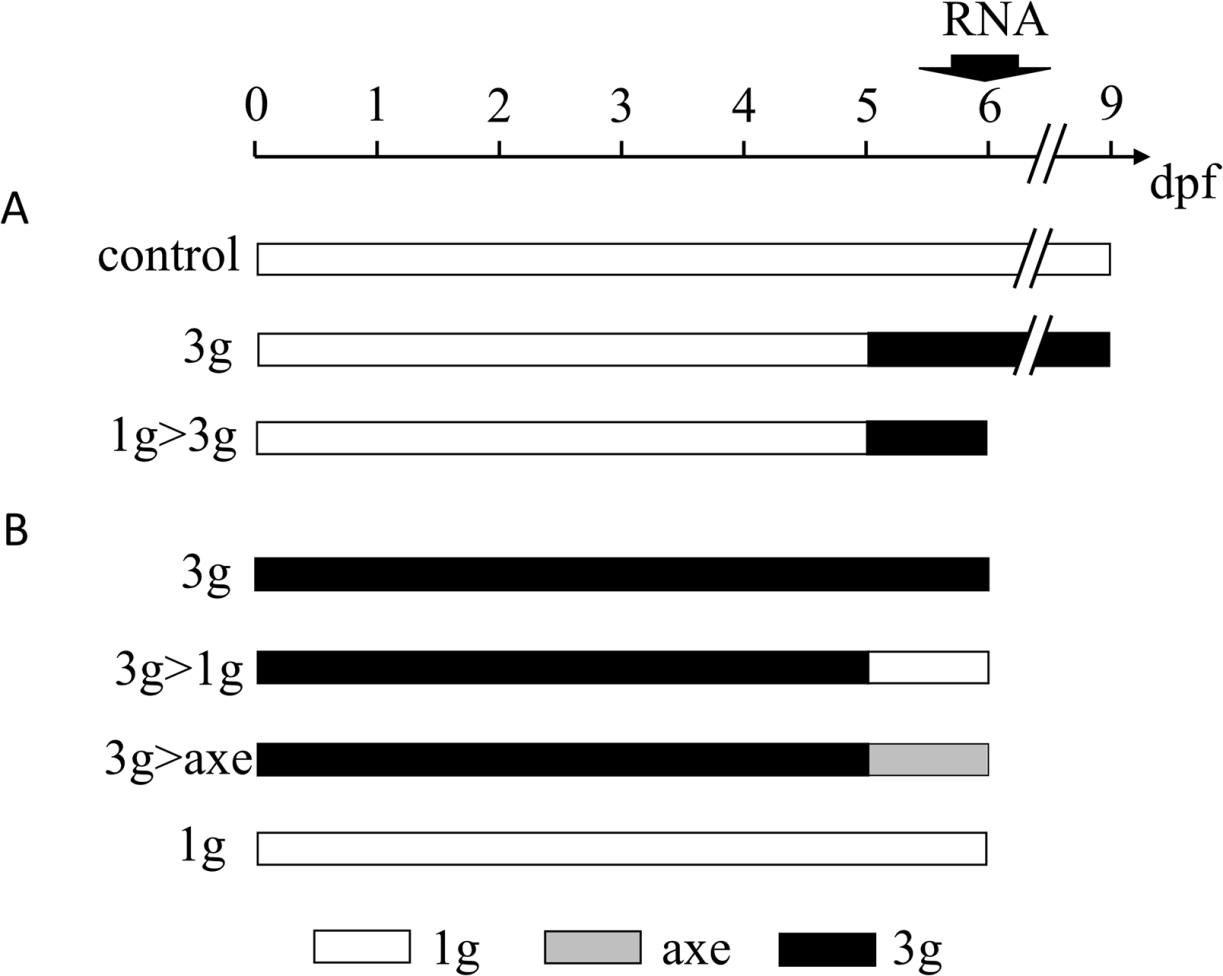
Schematic overview of the different hypergravity experiments. (A) larvae are placed at hypergravity at 5dpf until 9dpf (3g), while (control) larvae are kept at normal gravity for 9 days. Total mRNA was extracted at 6dpf and batches of larvae were fixed at 9dpf for Alizarin red staining of bone matrix. (B) Experiment in which the control larvae were placed at 3g and kept at 3g until 6dpf (3g), or returned at 5dpf to 1g outside (3g>1g) or on the axis of the centrifuge (3g>axe) for one day. An additional batch of larvae was kept at normal gravity until 6dpf (1g). RNA extraction and Alizarin red staining are performed at 6dpf. For abbreviations see legend to Fig 1.

**Fig 7:**
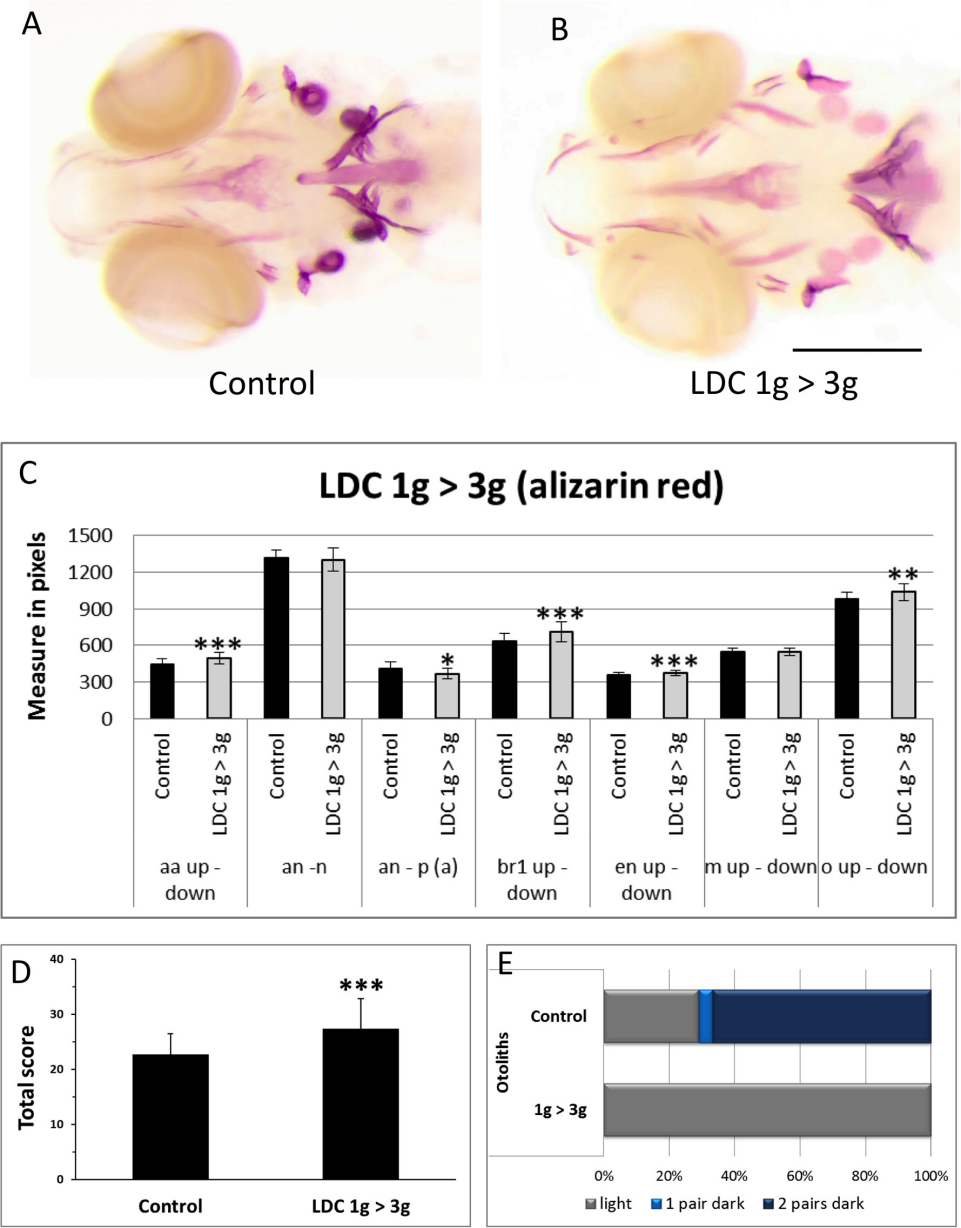
Effect of 3g hypergravity between 5–9dpf on bone formation. (A,B) Alizarin red staining of 9dpf control larvae (A) and larvae treated for 4 days in 3g hypergravity after 5 days at 1g (B). Ventral view, anterior to the left. (C) Comparison of morphometric measurements for some selected distances within the heads of control and 3g-treated larvae. Mean ± SD and t-test analysis were calculated for each measure on at least 20 individuals. * *p <* 0.05, ** *p <* 0.01 and ***p < 0.001. (D) Global score for bone formation in control and 3g treated larvae. (E) Comparison of cumulated frequencies of, respectively light, 1 pair dark or two pairs dark otoliths in control and 3g treated larvae. For abbreviations see legend to Fig 1.

To compare the effects caused by bio-chemical or hormonal treatment on bone formation to those exerted by bio-physical/mechanical constraints, we investigated the effects due to increased gravity using the large diameter centrifuge (LDC) at the European Space Agency, ESA (Noordwijk, Netherlands). In a first experiment, zebrafish larvae were grown at normal gravity (1g) until 5dpf. One half of the population was brought to 3g hypergravity in the LDC for another 4 days, while the other half was kept at 1g (see Fig 6). At 9dpf, the larvae were stained with Alizarin red for bone matrix (Fig 7A and 7B) and analyzed as described above. No difference was observed between the two samples when total length of the larvae or size of the eye or lens was determined (not shown). In the morphometric analysis, the 3g larvae present a larger head skeleton with a significant increase of the distance between the 2 anguloarticular bones, branchiostegal rays1, entopterygoid and the opercles (Fig 7C). In bone formation analysis (S3A Fig, S8 Table), the anguloarticular, branchiostegal ray2 and hyomandibular presented a clear over ossification, while the ceratohyal presented a significantly higher proportion of advanced ossification. In contrast, the dentary, maxilla and entopterygoid were not significantly affected (S3A Fig). The global score obtained by addition of the scores of all the separate structures revealed a significant increase of bone formation (from a score of 23± 4 to 27± 5.5) (Fig 7D). A clearly weaker calcification was observed in the otoliths. More than 60% of the controls show 2 pairs of dark otoliths (Fig 7A, 7B and 7E) compared to only lightly stained otoliths in the 3g group.

In addition, total mRNA was extracted from the larvae at 6dpf and whole genome gene expression was compared between larvae exposed for 1 day to 3g and 1g controls. The number of genes found to be modulated by hypergravity was 499, although the extent of induction or repression was surprisingly low (S9 Table), but significant as confirmed by RT-qPCR for 5 selected genes (S10 Table). Interestingly, among the affected biological functions (S11 Table), cellular and organism developmental processes ranked highest, only molecular transport appears in second position. More specifically, development and function of the skeletal and muscular system and connective tissue ranked highest, followed by the nervous and endocrine systems and finally hematological and cardiovascular systems. Among the specifically affected genes, many transporter and ion channel genes are present, reminiscent of the observations after VitD3 treatment. Interestingly, among the transcription factors, vitamin D receptor (*vdr*) is weakly, but significantly down-regulated, similar to the nuclear receptor *pparg*. Other prominent transcription factor genes are the homeo-box containing *pou3f3* and its potential partners *meis1* and *onecut1*. Construction of specific networks in three different organ systems using IPA (S4 Fig) revealed the inhibition of hubs like *MYC*, *PPARG*, vitamin D receptor (*VDR*), *NFKBIA* inhibitor in all systems, but also an extensive network specific to the cardiovascular system with, interestingly, a down-regulation of the growth factor receptor/Ras mediator gene *GRB2*.

### Effects of reduced gravity on bone and general development: "relative microgravity"

As an approach to investigate some of the effects on zebrafish physiology to be expected when going into real microgravity, we applied a protocol that we would qualify as "Reduced Gravity Paradigm" or "relative microgravity". The principle is to grow the zebrafish larvae for a defined period (5 days) in a hypergravity environment (this case 3g), before returning them to normal gravity for one additional day (Fig 6B). The effect of this decrease in gravity on bone formation and gene expression was then investigated.

**Fig 8:**
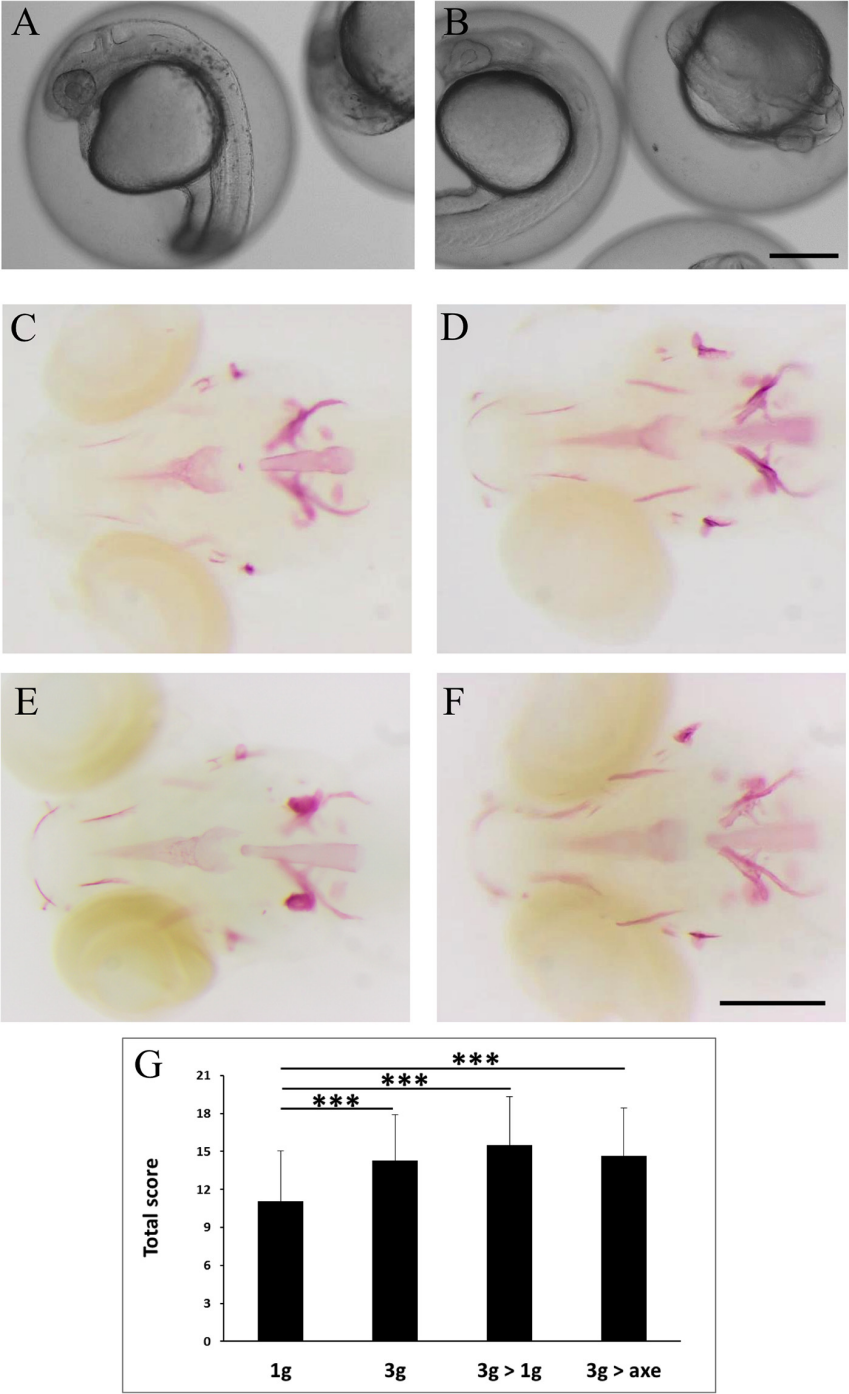
Effect of "relative microgravity" between 5–6dpf on bone formation. **(**A, B) comparison of pigmentation at 24hpf in 1g (A) and 3g (B) larvae. (C-F) Alizarin red staining of larvae kept at 1g until 6dpf (1g, C), control larvae kept at 3g until 6dpf (3g, D), larvae kept at 3g until 5dpf and returned to 1g off the centrifuge (3g>1g, E) or on the axis (3g>axe, F), Ventral view, anterior to the left. (G) Global scores for bone formation in control and the different treated larvae.

Zebrafish fertilized eggs were subjected at 4hpf to 3g hypergravity until 5dpf. For comparison, a parallel batch was grown at normal gravity outside of the centrifuge chamber (1g). The morphology of the embryos and larvae was monitored every day by microscopic observation, no striking effect was observed on developmental processes such as segmentation, organogenesis or hatching time. Only a clearly decreased (delayed) pigmentation was observed at 24hpf (Fig 8A and 8B), which was rapidly resolved as pigmentation was indistinguishable in 1g and 3g embryos at 2dpf.

At 5dpf, the larvae exposed for 5 days to 3g in the LDC, were separated in three distinct batches, one was left in the LDC for another day (3g) while the other two were returned to normal gravity for one day. One batch was kept in a separate incubator outside of the centrifuge chamber (3g>1g); the other was placed in an incubator positioned on the axis of the LDC (3g>axe), in order to maintain a rotation movement without increasing the gravitational force. The 1g batch continued to grow at normal gravity outside of the centrifuge chamber for the entire 6 days.

**Fig 9:**
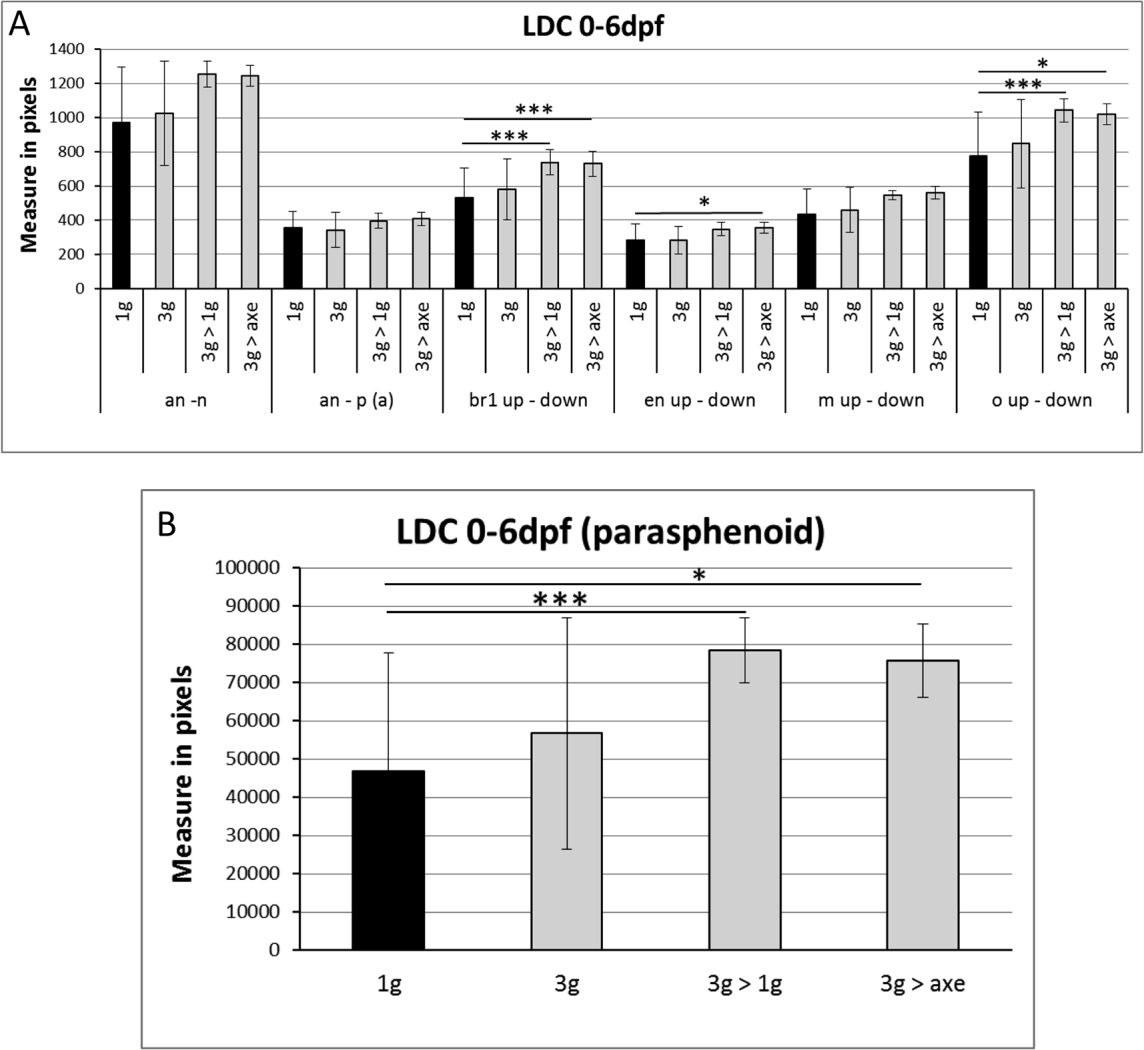
Morphometric analysis of bone elements at 6dpf after "relative microgravity”. The distances are measured in pixels. Mean ± SD and t-test analysis were calculated for each measure on at least 20 individuals. (A) Distances between the different cranial bone elements. (B) Area of the parasphenoid bone. * *p <* 0.05 and ***p < 0.001. For abbreviations see legend to Fig 1.

At 6dpf, all larvae were collected and stained for calcified structures using Alizarin red. Compared to larvae grown for 6 days at 1g, the bone structures in the head of all 3g exposed larvae appeared more intense (Fig 8C–8F), more specifically the anguloarticular, maxillary and, to a lesser extent the ceratohyal, hyomandibular and branchiostegal ray 1 (S12 Table). The global score was significantly increased in all samples exposed to 3g for 5 or 6 days (Fig 8G). Morphological analysis revealed a significant increase in the distance between branchiostegal rays 1, enteropterygoids and opercles, and an increase in the parasphenoid area (Fig 9).

### A central gene network is rapidly activated in reduced gravity

At 6dpf, all larvae were collected and used for mRNA extraction. Gene expression was determined by micro-array analysis, larvae exposed to 3g for the entire 6 days were chosen as control (S13–S15 Tables). Relative to this hypergravity sample, a remarkable similarity was observed in the biological functions affected in the normal gravity larvae (Table 2). Among the top ten functions modulated in each condition we found, on the one hand cell growth and proliferation, development, death and survival, organization and function, on the other hand embryonic and organismal (organ) development with a focus on connective tissue and cardiovascular development in the 6 days control at 1g. Only 3g>axe larvae presented 7 affected genes related to "auditory and vestibular system", related to their stay on a purely rotating position.

**Table 2:** Biological functions associated to "relative microgravity"-affected genes.

	1g		3g > axe		3g > 1g	
Category	p-value	N	p-value	N	p-value	N
Cellular Growth and Proliferation	5.71^E-11^-4.98^E-03^	181	6.87^E-07^-6.95^E-03^	70	5.78^E-09^-3.15^E-03^	213
Cell Cycle	1.15^E-10^-4.98^E-03^	93	3.61^E-05^-7.6^E-03^	20	8.79^E-06^-2.63^E-03^	85
Organismal Survival	8.1^E-09^-8.1^E-09^	119	1.23^E-04^-7.24^E-03^	46	1.87^E-06^-3.23^E-03^	139
Cellular Development	4.9^E-08^-4.98^E-03^	156	6.89^E-08^-8.09^E-03^	60	2.17^E-06^-3.31^E-03^	205
Connective Tissue Development and Function	4.9^E-08^-4.98^E-03^	44	1.55^E-04^-6.95^E-03^	26	1.91^E-05^-3.3^E-03^	75
Tissue Development	4.9^E-08^-4.6^E-03^	104	6.89^E-08^-8.11^E-03^	65	2.26^E-06^-3.31^E-03^	177
Cell Death and Survival	1.79^E-07^-4.99^E-03^	157	6.01^E-09^-8.25^E-03^	59	1.49^E-10^-3.35^E-03^	203
DNA Replication. Recombination. and Repair	1.11^E-06^-4.98^E-03^	77	4.27^E-03^-7.37^E-03^	7	2.22^E-03^-2.63^E-03^	8
Cardiovascular System Developt and Function	9.78^E-06^-3.03^E-03^	28	1.1^E-05^-7.81^E-03^	28	3.76^E-06^-3.02^E-03^	92
Hematological System Developt and Function	9.78^E-06^-4.98^E-03^	57	4.53^E-05^-8.11^E-03^	32	6.3^E-06^-3.31^E-03^	114
Cellular Assembly and Organization	1.75^E-05^-4.98^E-03^	80	4.21^E-06^-7.65^E-03^	42	1.83^E-05^-3.02^E-03^	90
Cellular Movement	2.64^E-05^-4.55^E-03^	94	2.43^E-04^-7.89^E-03^	35	1.82^E-06^-3.02^E-03^	132
Cell Morphology	3.38^E-05^-3.03^E-03^	89	2.43^E-04^-7.65^E-03^	47	7.12^E-08^-2.7^E-03^	144
Amino Acid Metabolism	4.27^E-05^-4.98^E-03^	23	2.37^E-03^-6.69^E-03^	3	1.08^E-05^-1.83^E-03^	13
Small Molecule Biochemistry	4.27^E-05^-4.98^E-03^	100	1.6^E-05^-7.6^E-03^	36	1.08^E-05^-2.74^E-03^	72
Embryonic Development	4.66^E-05^-4.98^E-03^	83	6.89^E-08^-7.81^E-03^	52	2.46^E-07^-3.22^E-03^	141
Organismal Development	4.66^E-05^-4.93^E-03^	85	6.89^E-08^-7.81^E-03^	63	2.46^E-07^-3.22^E-03^	203
Cell-To-Cell Signaling and Interaction	7.89^E-05^-4.39^E-03^	27	6.44^E-05^-7.6^E-03^	17	2.07^E-03^-2.07^E-03^	7
Cellular Function and Maintenance	1.27^E-04^-4.39^E-03^	67	4.21^E-06^-7.65^E-03^	37	1.83^E-05^-3.02^E-03^	148
Energy Production	1.72^E-04^-2.24^E-03^	22	5.95^E-03^-7.37^E-03^	6		
Lipid Metabolism	1.72^E-04^-4.98^E-03^	60	6.84^E-05^-7.6^E-03^	30	4.52^E-05^-2.74^E-03^	58
Renal and Urological Developt and Function	1.72^E-04^-2.93^E-03^	25	2.04^E-04^-7.6^E-03^	5	2.56^E-03^-2.56^E-03^	2
Nucleic Acid Metabolism	1.74^E-04^-3.03^E-03^	36	1.6^E-05^-7.37^E-03^	15	2.63^E-03^-2.63^E-03^	3
Tissue Morphology	1.75^E-04^-4.98^E-03^	80	4.53^E-05^-7.81^E-03^	47	2.49^E-06^-3.15^E-03^	129
Cellular Compromise	2.25^E-04^-9.79^E-04^	13	5.37^E-04^-7.6^E-03^	15	4.79^E-04^-9.19^E-04^	9
Molecular Transport	2.25^E-04^-4.98^E-03^	89	1.6^E-05^-7.6^E-03^	41	4.52^E-05^-2.74^E-03^	106
Lymphoid Tissue Structure and Developt	2.29^E-04^-4.55^E-03^	24	1.18^E-03^-6.38^E-03^	17	2.24^E-05^-3.01^E-03^	36
Gene Expression	4.45^E-04^-4.39^E-03^	83	2.04^E-04^-7.81^E-03^	37	6.97^E-08^-2.28^E-03^	134
Carbohydrate Metabolism	5.2^E-04^-3.1^E-03^	43	6.84^E-05^-7.52^E-03^	19	2.63^E-03^-2.63^E-03^	3
Hematopoiesis	5.21^E-04^-4.55^E-03^	10	2.78^E-03^-7.76^E-03^	14	6.3^E-06^-3.31^E-03^	70
Hair and Skin Development and Function	6.67^E-04^-4.49^E-03^	19	1.88^E-03^-1.88^E-03^	6	8.79^E-06^-3.35^E-03^	47
Nervous System Developt and Function	8.28^E-04^-4.98^E-03^	40	8.72^E-06^-7.6^E-03^	46	2.37^E-05^-2.7^E-03^	76
Organ Morphology	8.73^E-04^-3.03^E-03^	18	3.32^E-05^-6.97^E-03^	33	4.58^E-06^-3.11^E-03^	87
Organ Development	1.06^E-03^-4.24^E-03^	33	**1.25** ^**E-06**^ **-7.6** ^**E-03**^	35	**2.26** ^**E-06**^ **-3.01** ^**E-03**^	109
Skeletal and Muscular Developt and Function	1.06^E-03^-4.28^E-03^	34	1.55^E-04^-7.6^E-03^	23	2.26^E-06^-3.11^E-03^	67
Immune Cell Trafficking	1.54^E-03^-1.71^E-03^	3	8.11^E-03^-8.11^E-03^	5	2.23^E-04^-2.79^E-03^	53
Reproductive System Developt and Function	1.54^E-03^-4.6^E-03^	10	2.37^E-03^-7.6^E-03^	8	1.21^E-03^-2.81^E-03^	21
Visual System Development and Function	1.7^E-03^-1.71^E-03^	6	**1.25** ^**E-06**^ **-4.5** ^**E-03**^	17	4.58^E-06^-1.78^E-03^	35
Post-Translational Modification	1.71^E-03^-4.98^E-03^	4	8.8^E-05^-5.83^E-03^	15	3.03^E-04^-1.43^E-03^	48
Digestive System Development and Function	2.64^E-03^-4.24^E-03^	8	1.17^E-04^-8.04^E-03^	24	6.28^E-05^-1.79^E-03^	52
Hepatic System Development and Function	2.64^E-03^-4.24^E-03^	6	2.04^E-04^-6.95^E-03^	15	1.02^E-04^-7.51^E-04^	28
Protein Synthesis	2.98^E-03^-2.98^E-03^	41	8.8^E-05^-3.54^E-03^	27	2.6^E-04^-2.24^E-03^	66
Vitamin and Mineral Metabolism	3.55^E-03^-3.55^E-03^	8	1^E-03^-3.18^E-03^	6	2.56^E-03^-2.56^E-03^	2
Organismal Functions	4.98^E-03^-4.98^E-03^	2	6.73^E-04^-4.27^E-03^	8	3.15^E-03^-3.15^E-03^	13
Protein Trafficking	4.98^E-03^-4.98^E-03^	2			2.45^E-03^-2.45^E-03^	19
Cell Signaling			8.8^E-05^-5.02^E-03^	14	6.4^E-04^-1.05^E-03^	46
Protein Degradation			3.54^E-03^-3.54^E-03^	13	2.24^E-03^-2.24^E-03^	13
Behavior			3.61^E-04^-3.82^E-03^	22	5.52^E-06^-3.15^E-03^	77
Auditory and Vestibular System Developt and Function			5.5^E-04^-3.82^E-03^	7		

Ingenuity Pathway Analysis of the lists of genes affected at 6dpf after 6 days at 1g (1g), or after 5 days at 3g and returned to 1g on the centrifuge axis (3g>axe) or outside of the centrifuge room (3g>1g), each time compared to 3g hypergravity treatment for 6 days (3g). Columns indicate respectively the function, the range of p-values (significance) associated to various sub-functions, and the number of genes concerned (N).

**Fig 10:**
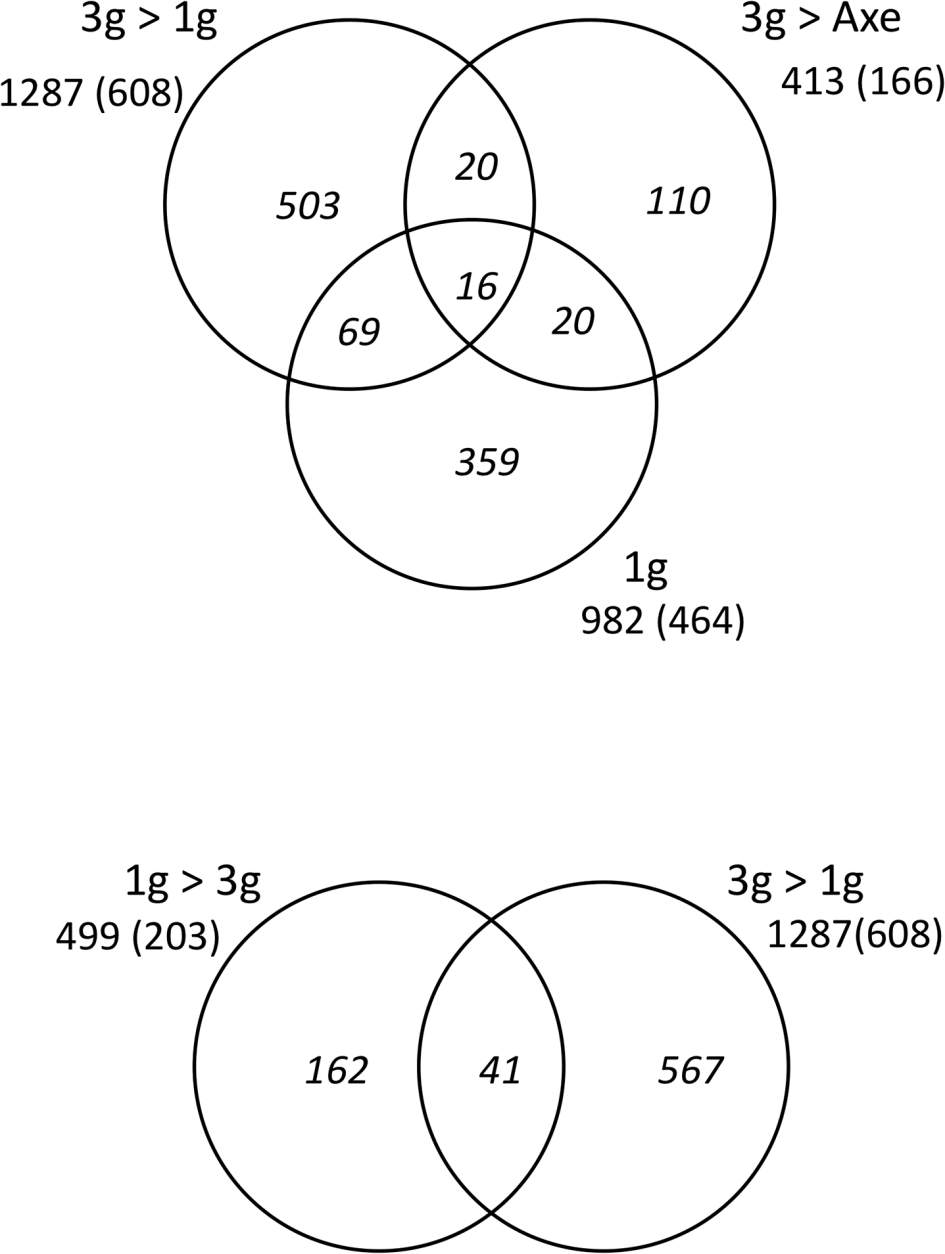
Number of genes affected in the various hypergravity experiments. The absolute number of probes resulting in a statistically significant hybridization signal is given for each condition. In parentheses, the corresponding number of genes with an annotation in IPA is given, while the Venn diagrams represent the number of genes unique to each condition and genes common to two or three conditions.

**Fig 11:**
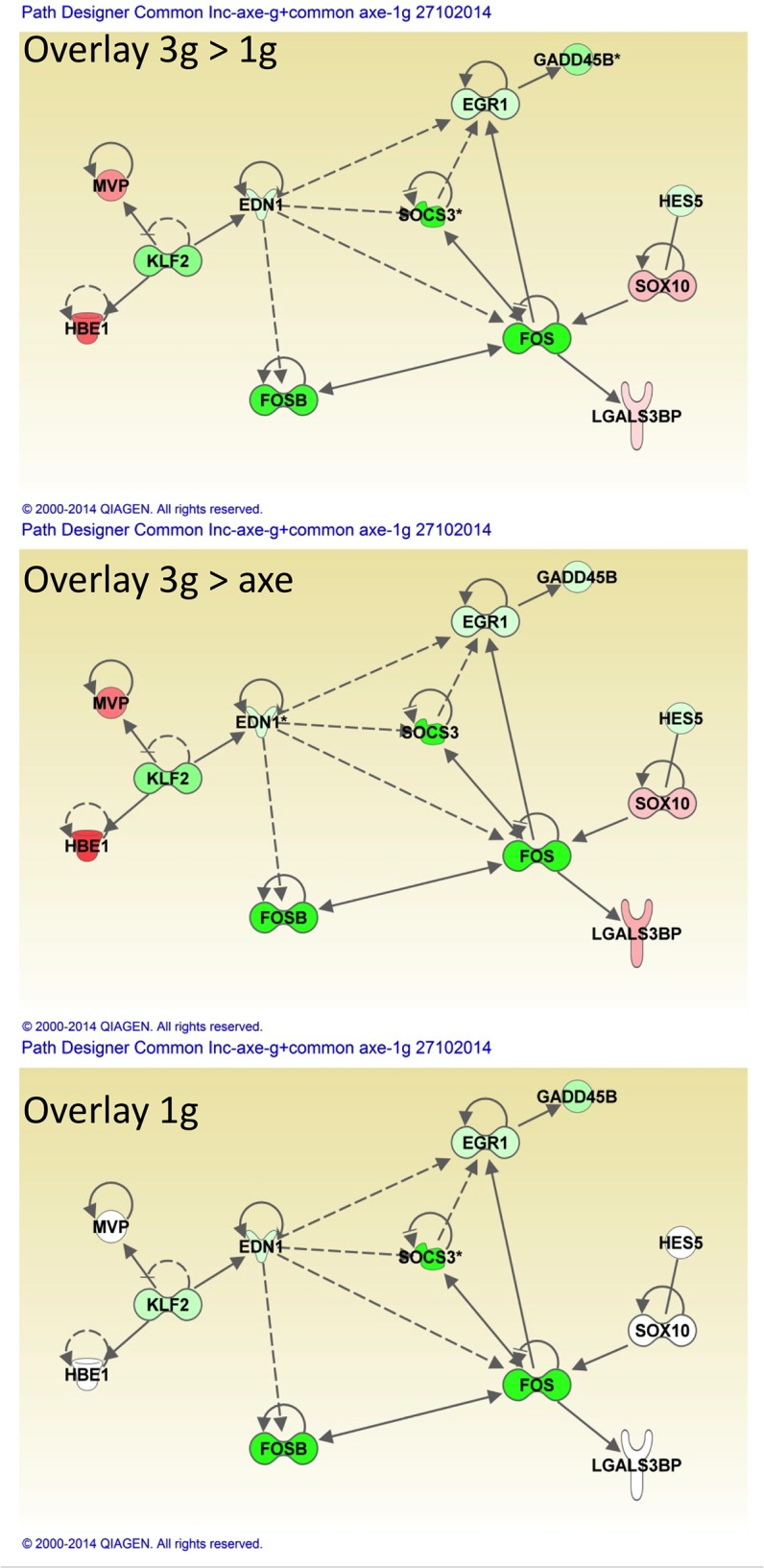
Network of genes affected in "relative microgravity" experiments. A network was constructed using the genes common to all three experiments, or the genes common only to 3g>1g and 3g>axe. Color overlay indicates the fold change relative to the 3g sample taken as control. Genes up-regulated (red), down-regulated (green), (*) indicates that the gene is represented by two or more probes on the microarray.

When comparing the affected genes in the three conditions, it appears that 16 genes are common to all three (Fig 10), while 20 genes are common only to the 1g samples between days 5 and 6 (3g>1g and 3g>axe). Respectively, 69 and 20 genes are common between the static 1g for 1 day (3g>1g) or rotating 1g (3g>axe) for 1 day and the larvae having spent all 6 days at 1g (1g). Several genes, mostly common to all three conditions, were selected and the modulation of their expression was confirmed by RT-qPCR (S16 Table). Regulatory networks were constructed using the genes common to all three conditions, but also using those common to the 1g for one day condition (3g>1g and 3g>1axe) (Fig 11). Strikingly, a network composed of 7 genes (FOS, FOSB, EGR1, EDN1, SOCS3, GADD45B, KLF2) that were affected in exactly the same manner in all three conditions could be constructed, indicating that they represent a central network that is affected by gravitational conditions. Most importantly, these central genes were affected to the same extent, relative to the 3g for 6 days control, whether the larvae were kept at 1g during the entire experiment or only for the last day, suggesting that their expression levels are specific to this gravitational condition and are rapidly (within one day) adapted to new conditions. Five additional genes (*MVP*, *HBE1*, *HES5*, *SOX10*, *LGALS3BP*) were only affected after 1 day at lower gravity (both 3g>1g and 3g>1axe), indicating that they may be actually involved in the mechanism for rapid adaptation to lower gravity. Further analyses were performed using all the genes common to any two of the conditions (S5 Fig), also analyzed according to their potential function in individual organ systems (S6 Fig). By extending the network that way, other nodes become apparent, such as the nuclear receptor PPARG, the protein chaperone HSP90AA1 and the regulatory peptide endothelin (EDN1) (S5 Fig). Expression of *NFKBIA*, a target gene for the NFkB pathway coding for an inhibitor of this pathway, was decreased in two conditions, potentially causing the decreased expression of the antiproliferative factor BTG2 [80] observed in all three conditions.

Finally, we compared the genes affected in the 1g>3g experiment, which experienced a shift from 1g to 3g on day 5, with those affected in the 3g>1g experiment where the larvae were returned to 1g after 5 days at 3g. Among the affected genes, 41 were common to both experiments (Fig 10) that could be assembled in a regulatory network (S7 Fig). Two regulatory genes attracted our attention due to their increased expression in the 3g environment (note the fold change relative to the 1g control in the 1g>3g, and relative to the 3g sample in the 3g>1g experiment): SOX3 is a transcription factor shown to be involved in neural, pituitary and craniofacial development [81], while the *HEY1* gene is a target of Notch signaling and was shown to regulate bone homeostasis [82]. Two other genes, coding for embryonic hemoglobin HBE1 and the oligopeptide transporter SLC15A1 were down-regulated at 3g.

## Discussion

Zebrafish present remarkable degrees of similarity with mammals in the molecular mechanisms involved in their developmental biology and physiology. Moreover, their ease of husbandry, high fecundity, and small size paves the way for a possible future space experiment, triggering the proposal of their use for the study of gravitational biology [83–89]. We decided to explore the effects of increased gravity (hyper-g) on zebrafish larvae using the large diameter centrifuge (LDC). This device allows applying a well-controlled and constant centrifugal force (1g-20g) by minimizing, through the large diameter of the rotating arms, the possible effects of Coriolis force [64].

Our aim was to concentrate on the effects on bone formation, therefore we chose to start the experiments at 5dpf, when perichondral ossification is taking place within all major cranial cartilage elements and intramembranous bone formation is ongoing. We evaluated the effects on cartilage and bone formation by staining these structures after several days of treatment, at 9 or 10dpf. For a more detailed, more accurate and more objective evaluation of skeletal development, we developed two different, but complimentary methods for analyzing images of stained zebrafish larvae. The first one uses a number of landmarks placed manually within the images (using the software environment CYTOMINE) and allows automatic extraction of distances and angles between these landmarks, ultimately resulting in a morphometric description of the head skeleton. The second one is based on manually assigning a developmental score to each cranial bone element within each image, enabling us to calculate a mean score for each element and a global score for each individual.

To validate these approaches, we performed two treatments of zebrafish larvae whose effects had been previously described [63]. The first treatment uses exogenous vitamin D3 (VitD3)[90] to increase bone formation, indeed the general VitD3 metabolism in teleosts is similar to that in mammals, teleosts possess two vitamin D receptors (VDRs) and knock-down of VDRa expression causes a decrease of calcium ion uptake [90]. PTH and related peptides are known hypercalcemic agents in mammals, however their function is more controversial in teleosts, depending on the species [91]. Although teleosts do not present a parathyroid gland, they do produce PTH in the gills, probably in cells identified by the expression of *gcm2*, a gene whose orthologues are required for parathyroid development in chicken and mouse [92, 93]. PTH administration induced hypercalcemia in fugu (*Tetraodon nigrividans*) by inducing both osteoblast and osteoclast function and by decreasing scale calcium content [94]. Genes homologous to the mammalian PTH-related peptides (PTHrP) were found in teleosts, they are more widely expressed [95], they increase calcium uptake in sturgeon (*Acipenser nacarii*) [96]and were shown to play different roles in craniofacial development in zebrafish [97]. Blocking PTH signaling through the use of a PTH/PTHrP antagonist resulted in a decreased hypercalcemic response to estradiol in sea bream (*Sparus aurata*) [98]. Finally, four stanniocalcin (*stc*) genes are present in fugu and zebrafish, only *stc1-a* expression was sensitive to the calcium concentration in water [99].[98] while PTHrP and Stc were shown to have opposing effects on calcium uptake in intestinal explants [100]. Depending on the mode of administration (intermittent or continuous) PTH and PTHrP were shown, respectively to increase or decrease bone formation in zebrafish [101] or seabream [102].

**Fig 12:**
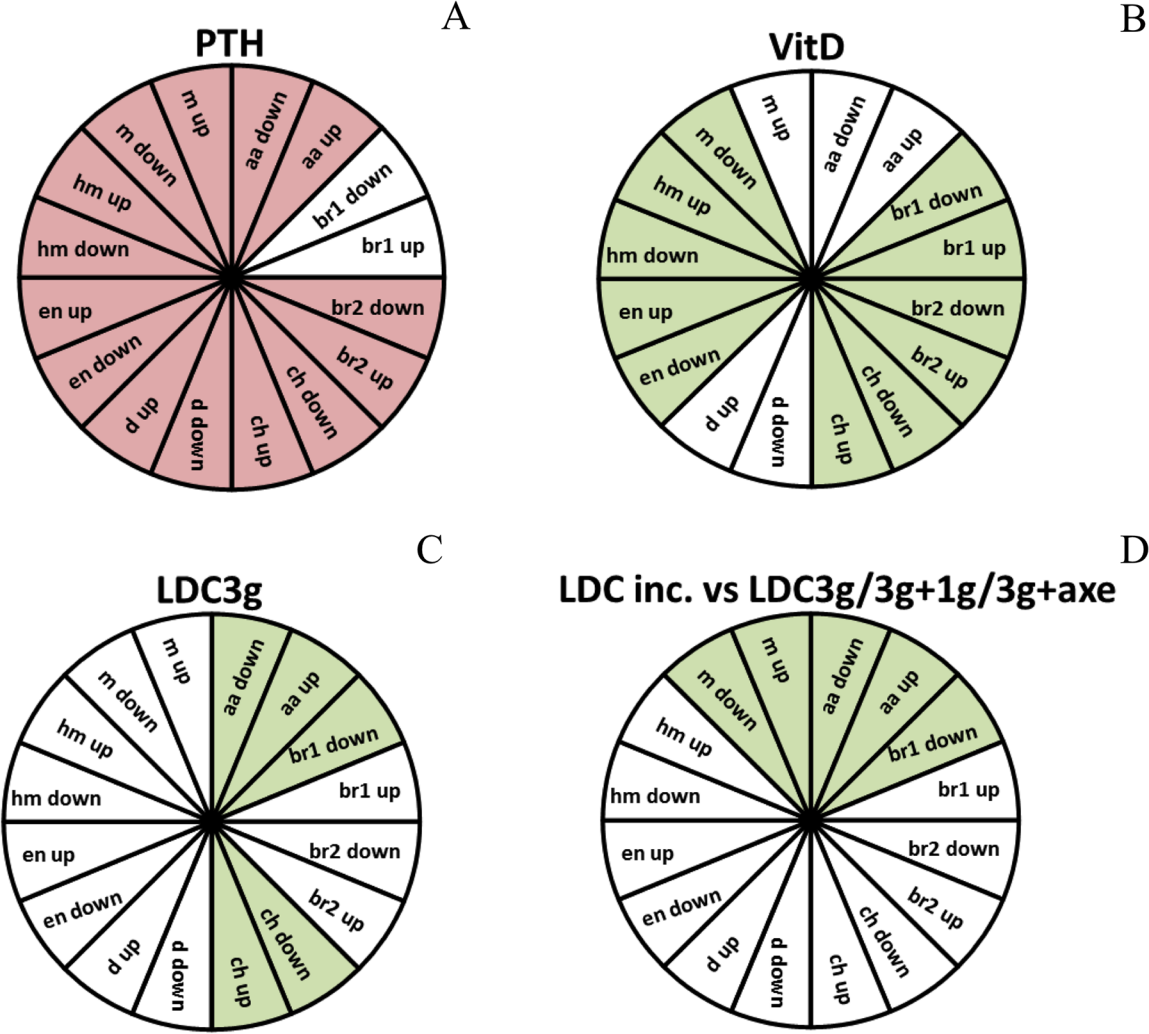
Summary graphs comparing the bone formation scores for each structure in the different experiments. Statistical analysis was performed by X² of Pearson and a logistic regression. In red, the scores are significantly increased. In green, the scores are significantly decreased. (A) PTH. (B) VitD3. (C) 3g hypergravity between 5–6dpf (D) "relative microgravity". For abbreviations see legend to Fig 1.

We confirm the effects described in zebrafish on general bone formation and in addition, the combined approach allowed us give a more detailed description of these effects. Although the general morphology was preserved in both cases, VitD3 treatment lead to a broader jaw both in cartilage and bone and a longer head in bone, while PTH treatment leads to an increased length of the ceratohyal cartilage, a general decrease of ossification, a decreased length of the parasphenoid bone and a broadening of the posterior head skeleton. The discrepancy between cartilage and bone concerning the longer head probably results from the fact that the landmarks used in bone (parasphenoid and notochord) do not have a real equivalent in cartilage and may mineralize independently from it. When we applied the same method to larvae subjected to hypergravity, we observed a broadening of the entire head skeleton (increased distance between symmetrically paired elements), for both types of treatment: 3g between 5–9dpf (1g>3g experiment, Fig 7A–7C), and 3g between 0 and 6dpf (experiments 3g, 3g>1g and 3g>axe, Fig 8). Similarly, the developmental scoring method allowed a more differentiated description of the observed effects (Fig 12). While VitD3 treatment caused a generally significant increase in ossification of most elements, this was less prominent for the maxillary and absent for the anguloarticular. Conversely, the decrease of ossification caused by PTH treatment was significant for all elements except branchiostegal rays 1. Increased ossification was significant only in the anguloarticular and ceratohyal after 3g treatment between 5–9dpf (1g>3g), but extended to the maxillary in the earlier treatments from 0–5 or 6dpf. Importantly, exposing the larvae for 6 days to 3g (3g condition) or returning them to 1g for the last day (3g>1g and 3g>axe) did not significantly affect bone formation, indicating that 1 day of altered gravity is not sufficient to cause morphological changes in the skeleton. Understanding of the molecular mechanisms underlying these differential effects on the various skeletal elements and their morphology will require further investigation.

Exposure to 3g starting at 5dpf (1g>3g condition) led to increased bone calcification in the anguloarticular and ceratohyals at 9dpf (Fig 7), while the otoliths were clearly less stained. The decrease in otolith calcification was already previously described [103, 104] and was proposed to involve a regulatory mechanism linking gravity sensing to the production of carbonic anhydrase and other matrix proteins in the inner ear [105–107]. Thus, the decrease in otolith calcification after prolonged exposure to 3g was expected, but it also emphasizes the specificity of the observed increase in ossification.

During early exposure to 3g (in the "relative microgravity" experiments), we observed a transient delay in pigmentation at 24hpf, which was rapidly resorbed at 48hpf. This finding is reminiscent of the transient decrease in the number of melanocytes that was observed at 24hpf during early exposure to simulated microgravity using a Rotating Wall Vessel device [51]. It is at present unclear whether a common mechanism may explain such a similar delay both in hypergravity and in simulated microgravity.

We then turned to studying differences in gene expression caused by the various treatments. We chose to perform these studies using mRNA from entire larvae, as methods for isolation of specific cells, such as dissection or fluorescent cell sorting might not be available in a future space experiment. First, we followed expression of bone-specific genes during normal development between 6 and 10dpf. We observed a sharp rise of mRNA coding for bone matrix proteins Sparc, Bglap, Spp1 and Col1a2 followed by a rapid decrease after 7dpf, suggesting that the major part of the bone matrix is formed at 7dpf and that further ossification is mainly due to mineral deposition. This is consistent with the observed sharp decrease of *osx* expression, followed with some delay by *dlx5a* expression, both indicating a decrease in osteoblast differentiation. The continuous decrease in the levels of *col10a1a* mRNA could be related to the proposed inhibitory effect of this factor on biomineralization [108, 109], while the large increase of *runx2b* and *pth1a* mRNA during the entire period could be related to some other functions of these factors [110, 111]. Following the modulation of gene expression during chemical treatments revealed a clear upward trend for bone matrix protein-encoding genes upon VitD3 treatment and a clear downward trend during PTH treatment. These trends are consistent with the assumption that bone matrix secretion plays a functional role in the observed increase or decrease, respectively, in bone formation. Expression of *osx* is increased during the first day of VitD3 treatment and decreased during PTH treatment, again consistent with a respectively prolonged or shortened period of osteoblast differentiation, also further supported by the increase of *dlx5a* and *dlx6a* expression at 8–9dpf during VitD3 treatment.

Finally, to determine the effects on whole genome gene expression of the various treatments, we chose to concentrate on mRNA levels only after one day of treatment, as we are mainly interested in regulatory events. A summary of all the genes affected by any of the studied conditions is shown in S17 Table. Again, we validated our approach by investigating the effects of known regulators of bone formation. As expected, VitD3 treatment induced *cyp24a1* expression, while PTH administration led to a decrease in endogenous *pth1a* expression. Furthermore, VitD3 treatment caused significant changes in overall metabolism, as shown by the involvement of affected genes in molecular transport or lipid metabolism. Probably for this reason, functions related to embryogenesis or organ morphology rank much lower in the list of affected pathways. These findings are consistent with previous results, obtained using a deep sequencing (RNA-seq) approach, which also showed a high proportion of metabolic pathways affected by VitD3 treatment, administered either between 2 and 6–7dpf or between 6–7dpf [112]. In contrast, PTH treatment affected less genes, but these were more involved in developmental processes. Interestingly, several genes were regulated in opposite directions upon VitD3 or PTH treatment (Fig 5A and 5C), suggesting that they may be involved in the opposite effects on bone mineralization that we observed. However, when we classified the genes according to their known involvement in specific organ function (S4 Fig), these genes were more specifically known for their function in muscle, indicating that further investigations are required.

When comparing genes and pathways affected by hypergravity, cellular growth and proliferation functions ranked very high, followed by cellular, tissue and organismal development (Table 2). Among the canonical pathways affected (S18 Table), we found those involving IGF, as already mentioned, and those involving pituitary hormones Prl and Gh as well as nuclear receptors. Interestingly, finer analysis of the affected biological functions revealed that all hypergravity conditions acted on organism survival and cell apoptosis (S19 Table), although no effect on larval survival or growth was observed in our experiments. Affected regulatory networks comprise PPARG, involved in adipocyte differentiation and regulating blood glucose uptake, consistent with the presence of other genes connected to insulin function. This observation may be related to previous experiments in rodents that showed a decrease in fat mass in hypergravity [113, 114]. Another gene consistently induced by hypergravity in mammals is the *Hsp70* stress response gene [113, 114]. In zebrafish kept for the first two days at 3g, increased expression of a fluorescent reporter transgene *hsp70-gfp* hypergravity was shown mainly in the lens [49], however no induction of the *hsp70* gene was observed here, probably due to the later observation stages. This indicates that older fish larvae are probably less stressed by hypergravity than are mammalian systems. Note that changes in the *fli1-gfp* transgene expression were also only observed for exposures before 24hpf [115]. Similarly, a decrease of *ß-actin-gfp* transgene expression was described in Rohon-Beard neurons [115], which disappear after 80hpf. Other important nodes are the NFKBIA inhibitor of the NF-kB pathway, involved in immune and inflammatory responses, and the multifunctional MYC gene.

The c-FOS gene was first described as the cellular homolog of the viral oncogene causing murine osteosarcoma [116], while gene knock-out mice suffered from severe defects in bone development and haematopoiesis [117]. First microgravity experiments in murine carcinoma cells revealed a decreased induction of c-Fos and its heterodimeric partner c-Jun by growth factors [118, 119]. Decreased c-Fos expression in microgravity was also observed in osteoblastic cells [120, 121], while exposure to intense hypergravity (50–90g) caused an increased expression of c-Fos and Egr1 [122]. More moderate hypergravity conditions (3g) also revealed rapid (36 min) induction of c-Fos expression in osteoblasts [123], while both hypergravity loading and unloading caused increased expression in rat brains [124, 125]. This latter c-Fos induction was then considered as an indicator for neural activity in specific brain regions, in particular those related to vestibular sensing and processing [126–128]. Here, we show that exposure of zebrafish embryos to 3g hypergravity during the first 5–6 days of development leads to increased expression of *fos*, as part of a regulatory network composed of 6 other genes (*fosb*, *egr1*, *edn1*, *socs3a*, *gadd45b*, *klf2a*) that are induced in 3g conditions. Among these, the *fos* homolog *fosb* and the Zn-finger transcription factor gene *egr1* belong to the immediate-early class of genes that are rapidly induced by growth factors. In mouse, FosB knock-out leads to behavioral defects [129], while Egr1 null mice display sterility, impaired growth and pituitary development [130, 131]. Egr1 was also rapidly induced in osteoblast cells upon mechanical stress [132]. In zebrafish [133], *egr1* was shown to be part of a regulatory cascade controlling cartilage development [134] that is induced by Fgf signaling [135]. Edn1 is a vasoconstrictor peptide whose absence causes elevated blood pressure and craniofacial abnormalities [136] in mouse, while a zebrafish *edn1* mutant displayed mainly defects in cranial cartilage development [7, 137]. Socs3 is a suppressor of cytokine signaling; in mouse it was shown to inhibit placental and fetal liver erythropoiesis [138], while a zebrafish mutant in the paralog *socs3a* was deficient in hair cell development and regeneration in the inner ear and the lateral line neuromasts [139]. Gadd45b is a factor causing growth arrest upon DNA-damage, but also involved in hematopoiesis and immune response [140]. Finally, loss of the Klf2 gene in mouse causes defects in vascular, skeletal and craniofacial development and in erythropoiesis [141], while a zebrafish *klf2a* mutant displayed impaired cardiac valve development due to a deficient response to blood flow [142]. Klf2a was further shown to be required for nitric oxyde (NO) synthesis during artery and hematopoietic stem cell development [143], a process that is also highly involved in bone development [144–146]. Taken together, the network formed by these seven genes that are up-regulated in 3g conditions carries the potential to affect most processes that are known to be influenced by gravitational changes; from vestibular gravity sensing to hematopoiesis, immune response, vascular system and finally the skeletal system as was illustrated here. Moreover, this network is activated not only in larvae grown at 3g relative to larvae grown at 1g for 6 days, but also relative to larvae grown at 3g for 5 days and then returned to 1g for only one day (Figs 10 and 11). Increased expression of this gene network appears to be specific for hypergravity, while expression rapidly returns to normal after 1 day at 1g.

Five genes could be connected to this regulatory network that were specifically up-regulated (*MVP*, *HBE1*, *SOX10*, *LGALS3BP*) or down-regulated (*HES5*) after return to 1g conditions for 1 day (Fig 11). In mouse, Sox10 knock-out leads to neurological defects [147], while *sox10* mutant zebrafish are deficient in melanocyte pigmentation and inner ear development [148–150]. Similarly, Hes5 was shown to regulate neurogenesis [151], but also human cartilage differentiation under the control of Notch signaling [152]. Lgals3bp was shown to play a role in immune response and cell adhesion [153]. HBE1 codes for one of the embryonic hemoglobins, suggesting alterations in oxygen transport under different gravity conditions. MVP is a component of the ribonucleoprotein "vault" structures involved in nucleo-cytoplasmic transport and signal transduction [154]. Interestingly, loss of function studies for Mvp in zebrafish revealed defects in brain development and the response to mechanical stimulus (touch) [155]. The precise role of these genes in detection of decreased gravity and signal transmission to other physiological systems remains to be established.

Comparison of the 1g>3g and the 3g>1g experiments revealed the increased expression in hypergravity of two regulatory genes, SOX3 and HEY1, which both may play a role in bone development and/or homeostasis [81, 82], while HBE1 and SLC15A1 were down-regulated at 3g. Interestingly, only HBE1 is also regulated in the 3g>axe experiment, further supporting a general effect on oxygen transport, while only GADD45B expression was affected in all 3g experiments. None of the other genes composing the common regulatory network in "relative microgravity" was affected in the 1g>3g experiment. Actually, the overall effect of 1 day exposure to 3g was surprisingly small at the genome level, compared to the other hypergravity experiments (Tables S11, S13–S15), a result that is reminiscent of that observed previously in mammalian renal cells [156]. This observation suggests that the "Reduced Gravity Paradigm" is not simply a reversed hypergravity experiment, but rather that it represents a specific experimental condition. Future experiments will reveal whether this approach may be considered as a good approximation of microgravity.

In conclusion, we present an approach to objectively characterize cranial skeletal development in zebrafish larvae by morphometric image analysis and used this method to further characterize the effects of VitD3 and PTH on cartilage and bone formation. We have followed the expression of selected bone-related genes during 5 days of VitD3 or PTH treatment and analyzed whole genome gene expression after 1-day treatment. We have compared and correlated these results to the effects of hypergravity exposure on cranial skeleton formation. Finally, we have implemented a new type of hypergravity experiment, the "Reduced Gravity Paradigm", which allowed identification of a regulatory network of seven genes that are up-regulated in 3g, as well as several genes whose expression is rapidly modified when switching between 1g and 3g regimes. Future investigations will reveal whether these gene regulations are specific for particular organ systems and how they contribute to the overall physiological adaptation to altered gravitational environments.

## Supporting Information

S1 Fig Morphometric analysis of cartilage staining after 5 days chemical treatments. The distances are measured in pixels. Mean ± SD and t-test analysis were calculated for each measure on at least 20 individuals. * *p <* 0.05 and ***p < 0.001. (A, C) Distance after VitD3 treatment. (B, D) Distance after PTH treatment. Abbreviations as in 1. A) Morphometric analysis in VitD3-treated larvae cartilage revealed an increase of the distance between articulation (ar) "up" and "down", leading to a broader jaw as compared to untreated animals, while (A, C) all the other distances remained unchanged. B) Morphometric cartilage analysis of larvae treated with PTH for 5 days revealed a significant increase in length of the ceratohyal cartilages only (D).
(JPG)

S2 Fig Gene pathways affected after VitD3 treatment between 5–6dpf. Genes filtered according to the described function for their human homologs using IPA in muscle or bone function. Genes up-regulated (red), down-regulated (green), (*) indicates that the gene is represented by two or more probes on the microarray.
(JPG)

S3 Fig Changes in the extent of bone formation in hypergravity experiments. Bone development is classified for each element into different categories: Absent (no structure present; red), early ossification (beginning of the bone ossification; yellow), advanced ossification (the structure is present and already developed as the control; green) and over ossification (the structure is more developed compared to the control; purple). Cumulated frequencies in % are represented for each element. As no significant difference was observed for paired structures between left and right (up and down), their scores have been combined. Statistical analysis was performed by X² of Pearson and a logistic regression. (A) Cumulated frequency after 3g between 5–9dpf. (B) Cumulated frequency at 6dpf in the larvae left for 6 days at 3g, or the "relative microgravity" experiments (3g-axe and 3g>1g) relative to the 1g control. For abbreviations see legend to 1.
(JPG)

S4 Fig Regulatory networks related to different tissues after 3g hypergravity between 5–6dpf. Genes filtered according to the described function for their human homologs using IPA in bone, muscle, or cardiovascular system function. Genes up-regulated (red), down-regulated (green), (*) indicates that the gene is represented by two or more probes on the microarray.
(JPG)

S5 Fig Network of genes affected in "relative microgravity" experiments. A network was constructed using the genes common to any two of the three experiments. The color overlay indicates the fold change in each experiment (1g, 3g>1g and 3g>axe) relative to the 3g sample taken as control. Genes up-regulated (red), down-regulated (green), (*) indicates that the gene is represented by two or more probes on the microarray.
(JPG)

S6 Fig Tissue-specific networks of genes affected in "relative microgravity" experiments. Networks were constructed using the genes common to any two of the three experiments and filtered according to the described function for their human homologs using IPA in bone, muscle or cardiovascular system function. The color overlay indicates the fold change in the 1g experiment (1g, 3g>1g and 3g>axe) relative to the 3g sample taken as control. Genes up-regulated (red), down-regulated (green), (*) indicates that the gene is represented by two or more probes on the microarray.
(JPG)

S7 Fig Network of genes affected in "relative microgravity" and 3g between 5–6dpf (1g>3g) experiments. A network was constructed using the genes common to the 3g>1g and 1g>3g experiments. The color overlay indicates the fold change in each experiment relative to the respective control: control is 1g for the 1g>3g, and 3g for the 3g>1g experiment. Genes up-regulated (red), down-regulated (green), (*) indicates that the gene is represented by two or more probes on the microarray.
(JPG)

S1 Table List of oligonucleotides used for RT-qPCR experiments. (DOCX)

S2 Table Ossification scores for individual bone elements in control and 5 days VitD3-treated larvae. (A) The bone structures distributed in 2 categories (early and advanced ossification) (B) The bone structures distributed in 3 categories (early, advanced and over ossification)
(DOCX)

S3 Table Ossification scores for individual bone elements in control and 5 days PTH-treated larvae. (A) The bone structures distributed in 2 categories (early and advanced ossification) (B) The bone structures distributed in 3 categories (absent, early and advanced ossification)
(DOCX)

S4 Table Genes affected in larvae treated with VitD3 between 5–6dpf relative to control. The indicates the human homolog of the gene, its "Entrez" gene name, the log ratio of VitD3-treated larvae compared to control, the presence of duplicate probes on the microarray (D) and the type of protein it encodes. Genes are arranged according to their type and in alphabetical order.
(DOCX)

S5 Table Biological functions associated to genes affected by VitD3. Ingenuity Pathway Analysis of the list of genes affected at 6dpf after VitD3 treatment for 24 hours. Columns indicate respectively the function, the range of p-values (significance) associated to various sub-functions, and the number of genes concerned (N).
(DOCX)

S6 Table Genes affected in larvae treated with PTH between 5–6dpf relative to control. The table indicates the human homolog of the gene, its "Entrez" gene name, the log ratio of PTH-treated larvae compared to control, the presence of duplicate probes on the microarray (D) and the type of protein it encodes. Genes are arranged according to their type and in alphabetical order.
(DOCX)

S7 Table Biological functions associated to genes affected by PTH. Ingenuity Pathway Analysis of the list of genes affected at 6dpf after PTH treatment for 24 hours. Columns indicate respectively the function, the range of p-values (significance) associated to various sub-functions, and the number of genes concerned (N).
(DOCX)

S8 Table Ossification scores for individual bone elements in control and 3g-treated larvae between days 5–6dpf. The fraction (in %) of larvae presenting the indicated score for each element is given, together with the statistical evaluation of a significant difference compared to control. (A) The bone structures distributed in 2 categories (early and advanced ossification) (B) The bone structures distributed in 3 categories (absent, early and advanced ossification)
(DOCX)

S9 Table Genes affected in larvae placed at 3g between 5 and 6dpf (1g>3g) relative to control. The indicates the human homolog of the gene, its "Entrez" gene name, the log ratio compared to larvae kept at 1g between 0 and 6dpf, the presence of duplicate probes on the microarray (D) and the type of protein it encodes. Genes are arranged according to their type and in alphabetical order.
(DOC)

S10 Table Comparison of fold change values from the microarray dataset with those observed by RT-qPCR for larvae placed at 3g between 5 and 6dpf (1g>3g) relative to control. The fold change and statistical significance (p-values) are given from the microarray data and the RT-qPCR confirmation experiments.
(DOCX)

S11 Table Biological functions associated to genes affected by hypergravity between 5–6dpf (1g>3g). Ingenuity Pathway Analysis of the list of genes affected at 6dpf after 3g hypergravity treatment for 24 hours (1g>3g). Columns indicate respectively the category of function, the range of p-values (significance) associated to various sub-functions, and the number of genes concerned.
(DOCX)

S12 Table Ossification scores for individual bone elements in larvae placed at 1g or 3g for 6 days or returned to 1g the last day. The fraction (in %) of larvae presenting the indicated score for each element is given, together with the statistical evaluation of a significant difference compared to control. (A) The bone structures distributed in 2 categories (early and advanced ossification) (B) The bone structures distributed in 3 categories (absent, early and advanced ossification)
(DOCX)

S13 Table Genes affected in larvae left at 1g relative to those left at 3g for 6 days. The indicates the human homolog of the gene, its "Entrez" gene name, the log ratio of (1g) larvae compared to larvae kept at 3g between 0 and 6dpf, the presence of duplicate probes on the microarray (D) and the type of protein it encodes. Genes are arranged according to their type and in alphabetical order.
(DOCX)

S14 Table Genes affected in larvae returned to 1g on the axis of the centrifuge between day 5–6dpf (3g>axe) relative to those left at 3g for 6 days. The indicates the human homolog of the gene, its "Entrez" gene name, the log ratio of (3g>axe) larvae compared to larvae kept at 3g between 0 and 6dpf, the presence of duplicate probes on the microarray (D) and the type of protein it encodes. Genes are arranged according to their type and in alphabetical order.
(DOCX)

S15 Table Genes affected in larvae returned to 1g outside of the centrifuge between day 5–6dpf (3g>1g) relative to those left at 3g for 6 days. The indicates the human homolog of the gene, its "Entrez" gene name, the log ratio of (3g>1g) larvae compared to larvae kept at 3g between 0 and 6dpf, the presence of duplicate probes on the microarray (D) and the type of protein it encodes. Genes are arranged according to their type and in alphabetical order.
(DOCX)

S16 Table Comparison of fold change (FC) values from the microarray dataset with those observed by RT-qPCR in the "relative microgravity" experiments. The fold change and statistical significance (p-values) are given from the microarray data and the RT-qPCR confirmation experiments. In the 3g>axe experiment, the human KLF2 gene in S12 is actually the *klf2b* zebrafish ortholog, in contrast to the *klf2a* ortholog shown here.
(DOCX)

S17 Table Heat map representation of gene regulation in the different conditions. The gene symbol and name is given, as well as the log(fold-change) values in the different experiments. Induction values are underlined in red (>1) or orange (between 0.378 and 1), repression values are underlined in blue (-0.378/-1) or green (<-1).
(DOCX)

S18 Table Heat map representation of canonical pathways affected in the different conditions. The corresponding—Log(p-value) obtained in IPA analysis was used for classification and are coded by underlining: red means >3, orange between 1 and 3, and yellow means <1.
(DOCX)

S19 Table Heat map representation of biological functions affected in the different conditions. The corresponding—Log(p-value) obtained in IPA analysis was used for classification and are coded by underlining: red means >4, orange between 1 and 3, and yellow means <1.
(DOCX)
