# Supplementary figures and images for: Zebrafish Bone and General Physiology Are Differently Affected by Hormones or Changes in Gravity
Source: PLoS One. 2015 Jun 10;10(6):e0126928. doi: 10.1371/journal.pone.0126928 (PMC4465622; doi:10.1371/journal.pone.0126928)

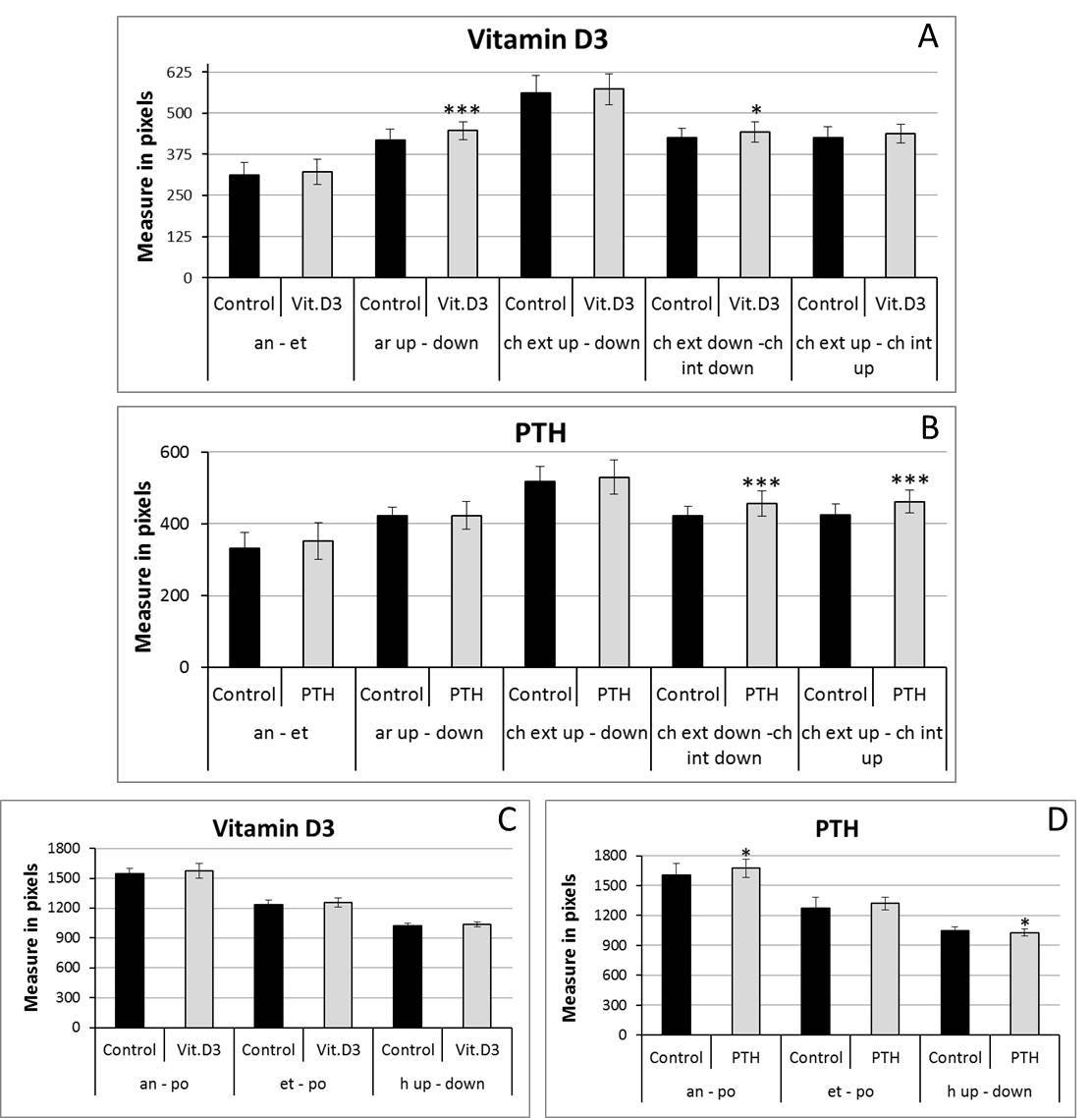

Supplement: S1 Fig — The distances are measured in pixels. Mean ± SD and t-test analysis were calculated for each measure on at least 20 individuals. * p < 0.05 and ***p < 0.001. (A, C) Distance after VitD3 treatment. (B, D) Distance after PTH treatment. Abbreviations as in 1. A) Morphometric analysis in VitD3-treated larvae cartilage revealed an increase of the distance between articulation (ar) "up" and "down", leading to a broader jaw as compared to untreated animals, while (A, C) all the other distances remained unchanged. B) Morphometric cartilage analysis of larvae treated with PTH for 5 days revealed a significant increase in length of the ceratohyal cartilages only (D). (JPG) [file pone.0126928.s001.jpg]

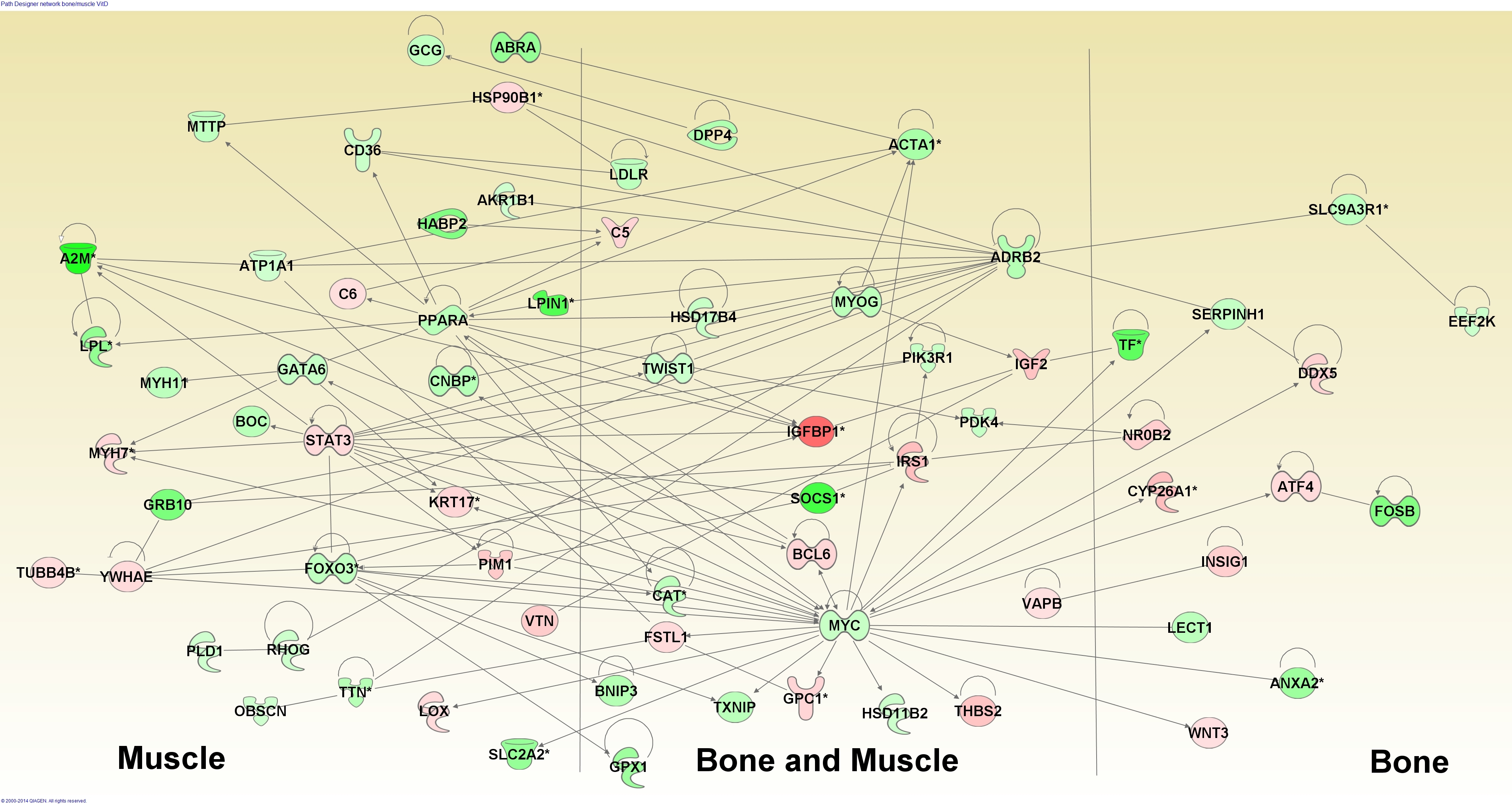

Supplement: S2 Fig — Genes filtered according to the described function for their human homologs using IPA in muscle or bone function. Genes up-regulated (red), down-regulated (green), (*) indicates that the gene is represented by two or more probes on the microarray. (JPG) [file pone.0126928.s002.jpg]

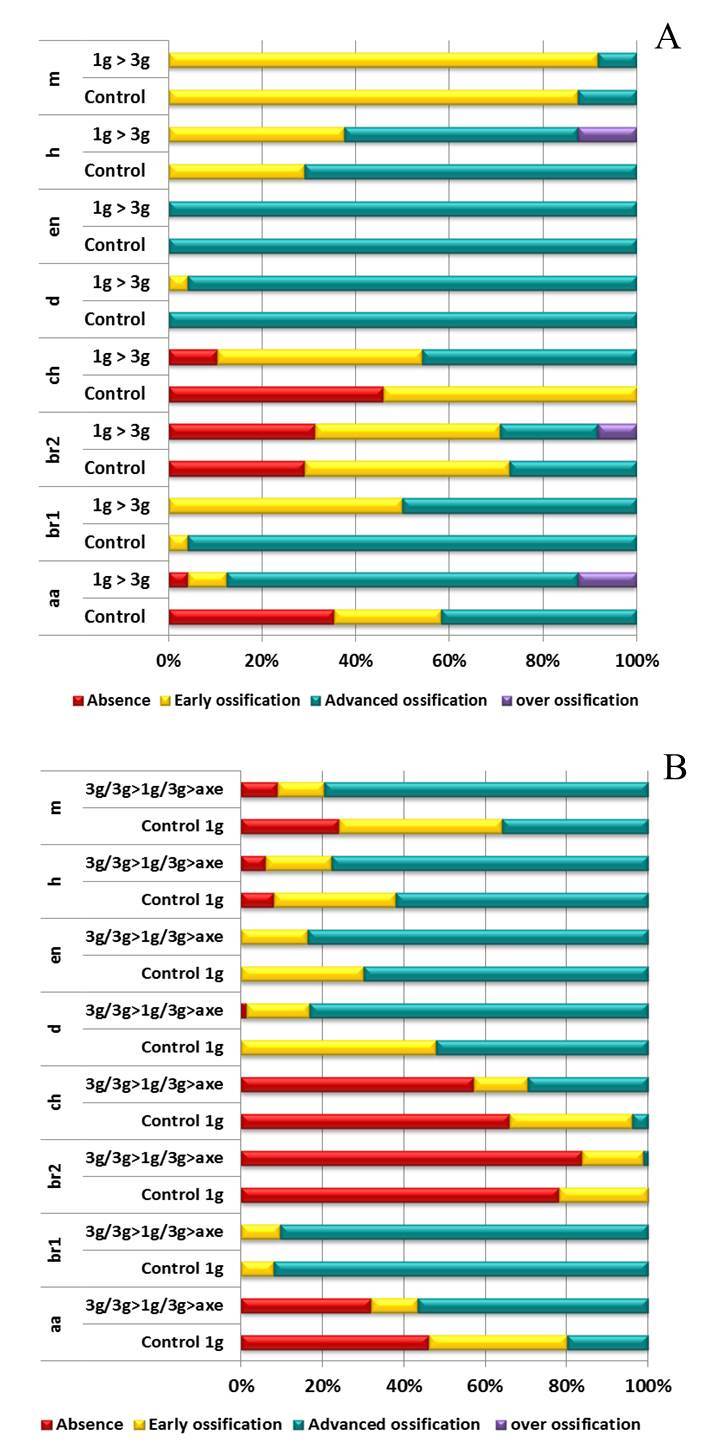

Supplement: S3 Fig — Bone development is classified for each element into different categories: Absent (no structure present; red), early ossification (beginning of the bone ossification; yellow), advanced ossification (the structure is present and already developed as the control; green) and over ossification (the structure is more developed compared to the control; purple). Cumulated frequencies in % are represented for each element. As no significant difference was observed for paired structures between left and right (up and down), their scores have been combined. Statistical analysis was performed by X² of Pearson and a logistic regression. (A) Cumulated frequency after 3g between 5–9dpf. (B) Cumulated frequency at 6dpf in the larvae left for 6 days at 3g, or the "relative microgravity" experiments (3g-axe and 3g>1g) relative to the 1g control. For abbreviations see legend to 1. (JPG) [file pone.0126928.s003.jpg]

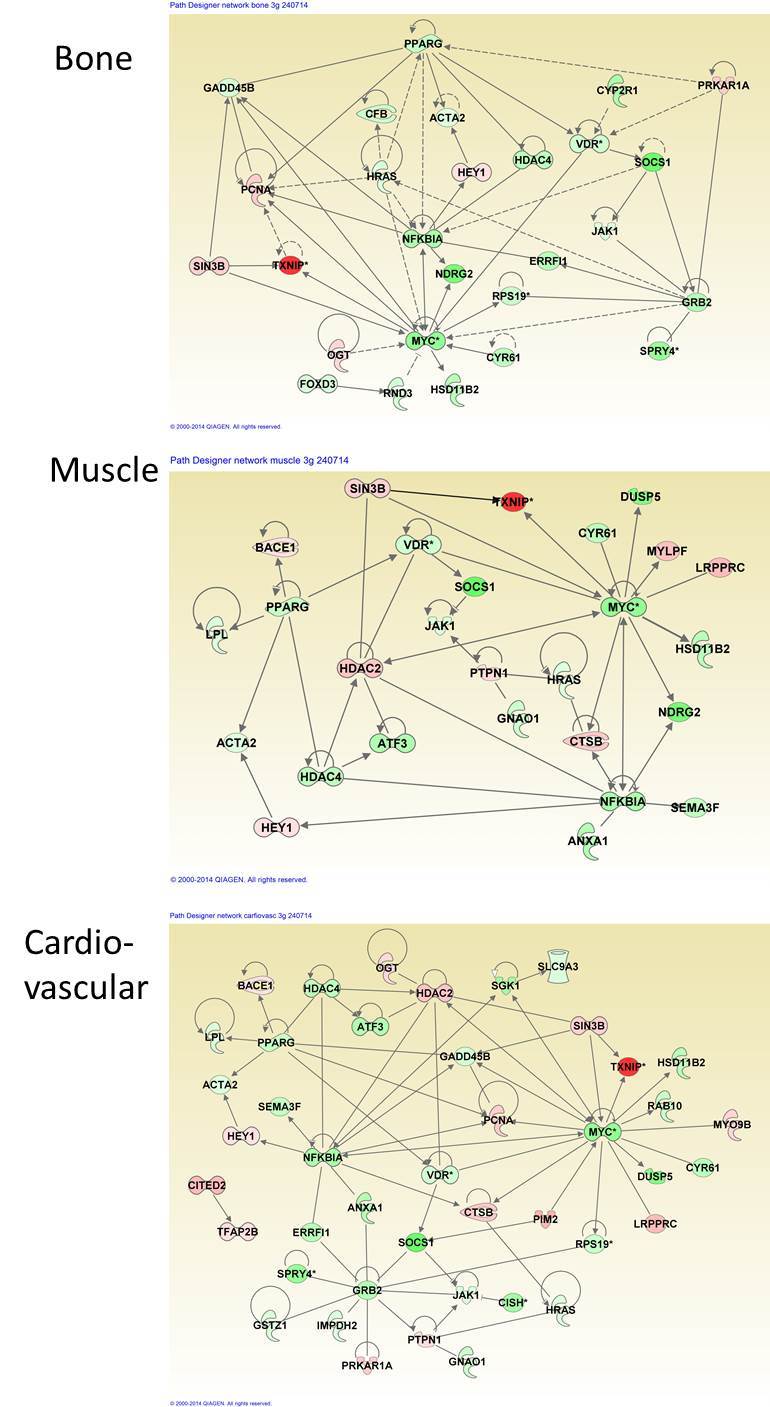

Supplement: S4 Fig — Genes filtered according to the described function for their human homologs using IPA in bone, muscle, or cardiovascular system function. Genes up-regulated (red), down-regulated (green), (*) indicates that the gene is represented by two or more probes on the microarray. (JPG) [file pone.0126928.s004.jpg]

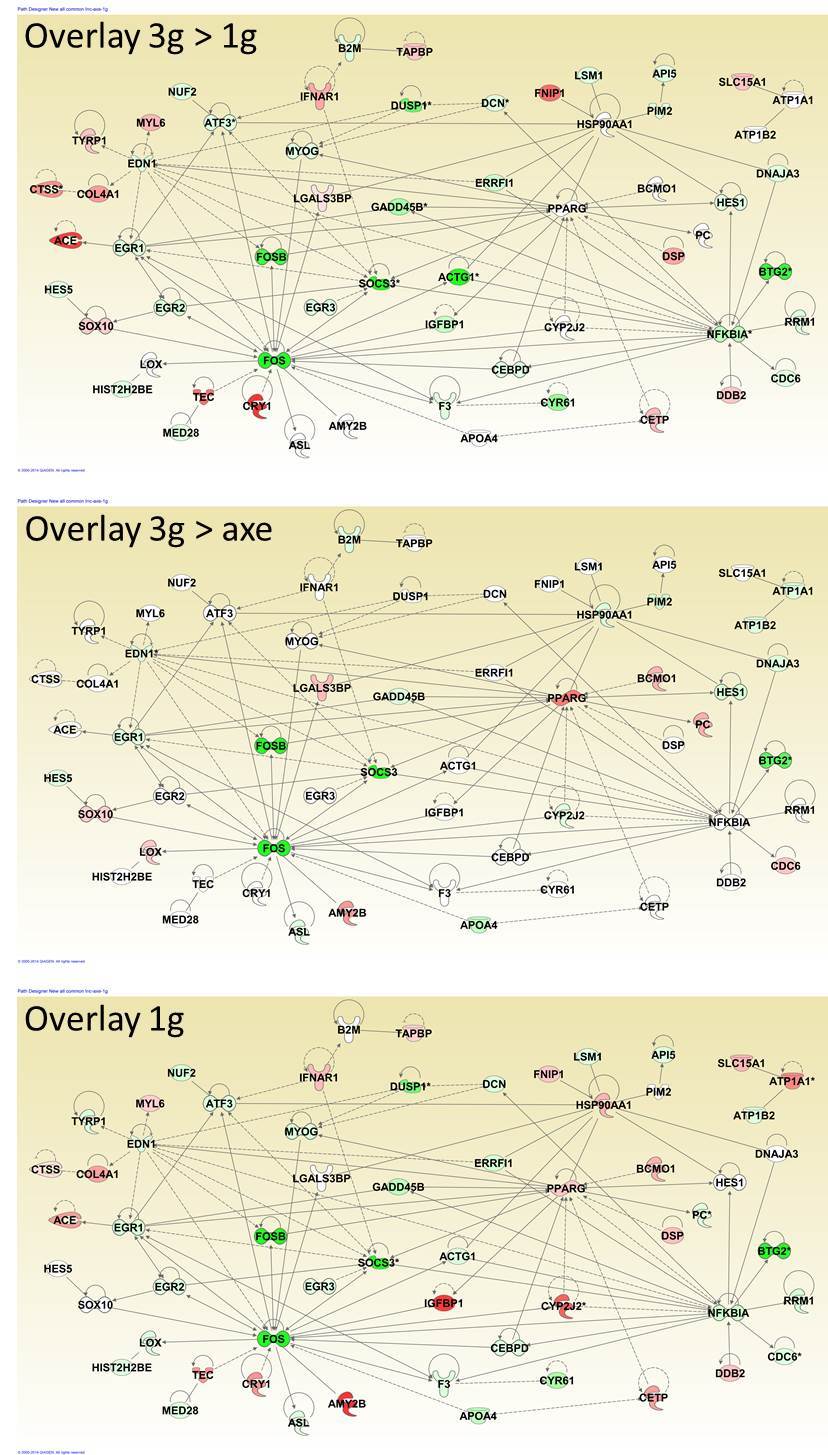

Supplement: S5 Fig — A network was constructed using the genes common to any two of the three experiments. The color overlay indicates the fold change in each experiment (1g, 3g>1g and 3g>axe) relative to the 3g sample taken as control. Genes up-regulated (red), down-regulated (green), (*) indicates that the gene is represented by two or more probes on the microarray. (JPG) [file pone.0126928.s005.jpg]

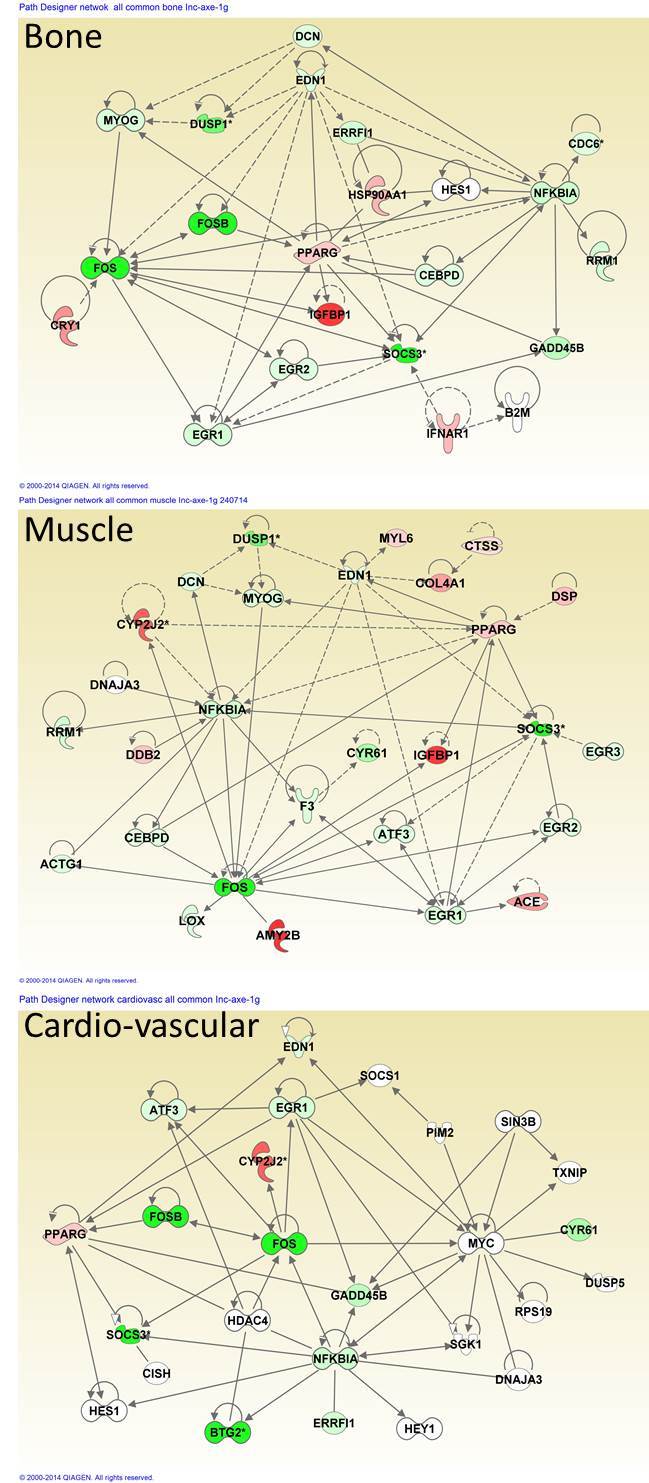

Supplement: S6 Fig — Networks were constructed using the genes common to any two of the three experiments and filtered according to the described function for their human homologs using IPA in bone, muscle or cardiovascular system function. The color overlay indicates the fold change in the 1g experiment (1g, 3g>1g and 3g>axe) relative to the 3g sample taken as control. Genes up-regulated (red), down-regulated (green), (*) indicates that the gene is represented by two or more probes on the microarray. (JPG) [file pone.0126928.s006.jpg]

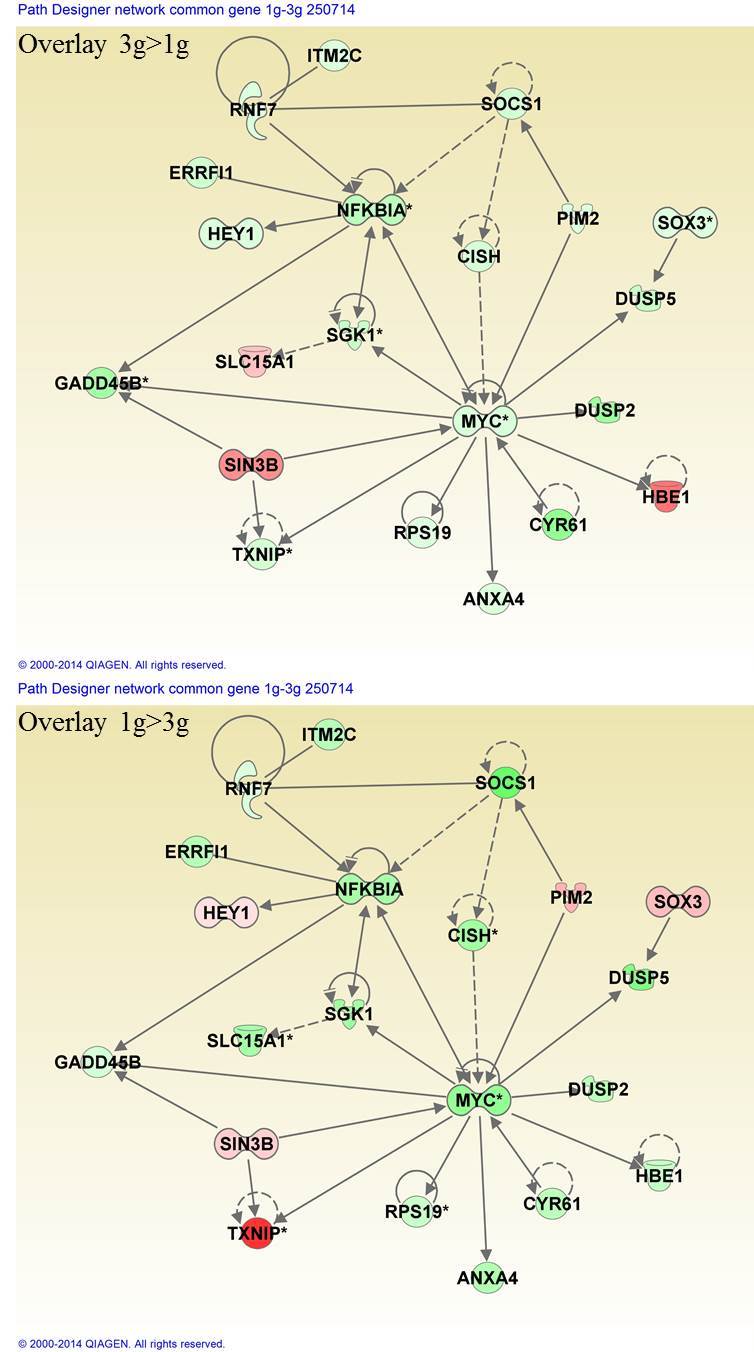

Supplement: S7 Fig — A network was constructed using the genes common to the 3g>1g and 1g>3g experiments. The color overlay indicates the fold change in each experiment relative to the respective control: control is 1g for the 1g>3g, and 3g for the 3g>1g experiment. Genes up-regulated (red), down-regulated (green), (*) indicates that the gene is represented by two or more probes on the microarray. (JPG) [file pone.0126928.s007.jpg]
