# Supplementary material for: Zebrafish Bone and General Physiology Are Differently Affected by Hormones or Changes in Gravity
Source: PLoS One. 2015 Jun 10;10(6):e0126928. doi: 10.1371/journal.pone.0126928 (PMC4465622; doi:10.1371/journal.pone.0126928)
Supplement: S1 Table — (DOCX) [file pone.0126928.s008.docx]

|  | **Primer** | **Forward (5' -> 3')** | **Reverse (5' -> 3')** | **Amplicon size (bp)** |
| --- | --- | --- | --- | --- |
| **Housekeeping gene** | **gadph** | GTGGAGTCTACTGGTGTCTTC | GTGCAGGAGGCATTGCTTACA |  |
|  |  |  |  |  |
| Relative | btg2 | GGTGTTCAGAGACGGACTCG | GAGGGTCCATTTCATGGTTG | 110 |
| microgravity | cebpb | TATGCAAGCAGCCAGTCAAC | TGGTACTGGGGCAAAGAGTC | 106 |
|  | fos | GGTATTACCCGCTCAACCAG | TGACAGTTGGCACGAAAGAG | 75 |
|  | fos b | TGCCGCTAAGTGTAGGAACC | CAGGCGCTCTTTCTCCTTC | 130 |
|  | klf2 | TGCACTTTTTCTGGATGTGG | TCCCAACTGCAATGATAGGG | 127 |
|  | socs3 | GGGAAGACAAGAGCCGAGAC | ACACACCAAACCCTGAGCTG | 114 |
|  |  |  |  |  |
| VitD3 and PTH | bglap | TCTTCCTGACTCCTCAGATACTAAAC | TTCCAGCCCTCTTCTGTCTC | 116 |
|  | col1a1 | CACAGAAGACCGGACCCTAC | CTTTGAGGCGAGGGAAGTT |  |
|  | col1a2 | CGTACTTGCCGTGACATCAG | GTCTGGCCAGTAGAGAAGTCG | 125 |
|  | col10a1 | TGCCCATGGTGAGAGATATG | GTGCCTGGTTCTCCTGCTAC | 105 |
|  | dlx5a | CCAATACCACGGAGTCAATG | GCTGTGGAGTATGAGCCGTA |  |
|  | dlx6a | AATCACCGTTTCCAGCAGAC | CGCCTTGTTTCAACAGCTTC | 142 |
|  | osx | AAATCAGCTCGTGGTTCTGG | GCTGTGGACAGGTTTCTTCC | 132 |
|  | pth1a | CAGGCCTCTGAGAAGCAAAC | GTTTCATCTGCAGCCAGTCC | 104 |
|  | runx2b | GTGGCCACTTACCACAGAGC | TCGGAGAGTCATCCAGCTT | 88 |
|  | sparc | AGGTGGAGACCGGAGAGTTT | CCCTTCTTGCAGTGATGGTT | 91 |
|  | spp1 | CGCCACAGTCTTCTGTGTACC | TTGAACAATTACAAGCTCTTCTGAG | 76 |
|  |  |  |  |  |
| VitD3 confirmation | cad | TCATTGGCGCAAAGACATAC | ACCCGTGATTCTGAGAGGTG | 107 |
|  | cyp24a1 | TGGAGATCAAACCATGGAAAG | CCGTCCAGCTTCATGACTTC | 145 |
|  | fgf4 | AAATCACCGGCGTACACAAC | CGTAAAGCTTCCCTTTGCTG | 132 |
|  | igfbp1 | TCCCGAGAGCTGGAGACC | AGCAGGTGATGCAGTGAGC | 127 |
|  | slc26a3 | AAGCCTACCGCAAACACAAG | CTTCATCCACCCAATGACAG | 116 |
|  | slc6a18 | AATGGGACAACAAGGTCCAG | CAGGTACGGGATCAGAAACG | 125 |
|  | socs1 | TGTATTGCCTGCTCTTGGAG | TGATTCCCTTCCACTGAACTG |  |
|  |  |  |  |  |
| PTH confirmation | fgf4 | AAATCACCGGCGTACACAAC | CGTAAAGCTTCCCTTTGCTG | 132 |
|  | mcph1 | TACGCCAGCTCTGAAAAACC | AACATTCGGAGTTGGTCAGC | 101 |
|  | ndrg2 | AAGCACCAAACCTGCTCAAC | CTCGTACGGAGCCTGATCTC | 131 |
|  | nrbp2 | GCATCGAGAGTGCGTACTTG | TCCACCTGCATCAGGTCCTC | 142 |
|  | rxra | GAGTGGGCGAAGAGGATTC | CCTGTGGCCAACAGTATTCC | 146 |
|  | slc6a18 | AATGGGACAACAAGGTCCAG | CAGGTACGGGATCAGAAACG | 125 |
|  |  |  |  |  |
| Hypergravity 3g | nr1d1 | CCGCAGTAGACACGAACAAC | CGAAGCAGGGTTGTGTAAGG | 141 |
|  | rhcg | CGAGGAGGCAGACACTAACTG | CAGGAAGGTCATGAGGAACC | 148 |
|  | socs1 | TGTATTGCCTGCTCTTGGAG | TGATTCCCTTCCACTGAACTG | 144 |
|  | spry4 | ATCGCAACGACCTGTTCATC | AATGTGGTGAGGAACCCTTG | 124 |
|  | txnip | GAGTCGGATGCGCTAAAGTC | CAGGCCTGAGAGTGATGGAG | 141 |

Table S1
