# Supplementary material for: Zebrafish Bone and General Physiology Are Differently Affected by Hormones or Changes in Gravity
Source: PLoS One. 2015 Jun 10;10(6):e0126928. doi: 10.1371/journal.pone.0126928 (PMC4465622; doi:10.1371/journal.pone.0126928)
Supplement: S2 Table — (A) The bone structures distributed in 2 categories (early and advanced ossification) (B) The bone structures distributed in 3 categories (early, advanced and over ossification) (DOCX) [file pone.0126928.s009.docx]

Table S2

A

|  |  |  |  | Score of ossification (Y) | | X² pearson | Logistic regression | |
| --- | --- | --- | --- | --- | --- | --- | --- | --- |
| Structures | Treat | N | Mean | early | advanced | p-value | OR (IC 95%) | p-value |
| anguloarticular down | Control | 28 | 0.75 | 20 (71.43%) | 8 (28.57%) |  | 1 |  |
|  | VitD3 | 29 | 0.86 | 18 (62.07%) | 11 (37.93%) | 0.454 | 1.528 (0.503-4.642) | 0.455 |
| anguloarticular up | Control | 28 | 0.75 | 7 (25.00%) | 21 (75.00%) |  | 1 |  |
|  | VitD3 | 29 | 0.83 | 5 (17.24%) | 24 (82.76%) | 0.473 | 1.600 (0.441-5.803) | 0.475 |
| branchiostegal ray1 down | Control | 28 | 0.54 | 13 (46.43%) | 15 (53.57%) |  | 1 |  |
|  | VitD3 | 29 | 0.86 | 4 (13.76%) | 25 (86.24%) | **0.007** | 5.417 (1.490-19.690) | **0.010** |
| branchiostegal ray1 up | Control | 28 | 0.57 | 12 (42.86%) | 16 (57.14%) |  | 1 |  |
|  | VitD3 | 29 | 0.86 | 4 (13.76%) | 25 (86.24%) | **0.015** | 4.688 (1.285-17.096) | **0.019** |
| branchiostegal ray2 down | Control | 28 | 0.21 | 22 (78.57%) | 6 (21.43%) |  | 1 |  |
|  | VitD3 | 29 | 0.59 | 12 (41.38%) | 17 (58.62%) | **0.004** | 5.194 (1.618-16.680) | **0.006** |
| branchiostegal ray2 up | Control | 28 | 0.21 | 22 (78.57%) | 6 (21.43%) |  | 1 |  |
|  | VitD3 | 29 | 0.79 | 6 (20.69%) | 23 (79.31%) | **<0.001** | 14.056 (3.933-50.232) | **<0.001** |
| maxilla down | Control | 28 | 0.36 | 18 (64.29%) | 10 (35.71%) |  | 1 |  |
|  | VitD3 | 29 | 0.66 | 10 (34.48%) | 19 (65.25%) | **0.024** | 3.420 (1.152-10.153) | **0.027** |
| maxilla up | Control | 28 | 0.29 | 20 (71.43%) | 8 (28.57%) |  | 1 |  |
|  | VitD3 | 29 | 0.38 | 18 (62.07%) | 11 (37.93%) | 0.454 | 1.528 (0.503-4.642) | 0.455 |

B

|  |  |  |  | Score of ossification (Y) | | | X² pearson | | Ordinal logistic regression | |
| --- | --- | --- | --- | --- | --- | --- | --- | --- | --- | --- |
| Structures | Treat | N | Mean | early | advanced | over | p-value | OR (IC 95%) | | p-value |
| ceratohyal down | Control | 28 | 1.71 | 8 (28.57%) | 20 (71.43%) | 0 (0%) |  | 1 | |  |
|  | VitD3 | 29 | 2.21 | 4 (13.79%) | 15 (51.72%) | 10 (34.48%) | **0.002** | 6.075 (1.747-21.127) | | **0.005** |
| ceratohyal up | Control | 28 | 1.75 | 7 (25%) | 21 (75%) | 0 (0%) |  | 1 | |  |
|  | VitD3 | 29 | 2.28 | 2 (6.90%) | 17 (58.62%) | 10 (34.48%) | **0.001** | 11.764 (2. 406-57.514) | | **0.002** |
| dentary down | Control | 28 | 1.93 | 2 (7.14%) | 26 (92.86%) | 0 (0%) |  | 1 | |  |
|  | VitD3 | 29 | 2.14 | 0 (0%) | 25 (86.21%) | 4 (13.79% | 0.050 | / | | / |
| dentary up | Control | 28 | 1.93 | 2 (7.14%) | 26 (92.86%) | 0 (0%) |  | 1 | |  |
|  | VitD3 | 29 | 2.17 | 0 (0%) | 24 (82.76%) | 5 (17.24%) | **0.029** | / | | / |
| entopterygoid down | Control | 28 | 1.86 | 4 (14.29%) | 24 (85.71%) | 0 (0%) |  | 1 | |  |
|  | VitD3 | 29 | 2.48 | 1 (3.45%) | 13 (44.83%) | 15 (51.72%) | **<0.001** | 33.972 (4.040-285.690) | | **0.001** |
| entopterygoid up | Control | 28 | 1.86 | 4 (14.29%) | 24 (85.71%) | 0 (0%) |  | 1 | |  |
|  | VitD3 | 29 | 2.45 | 2 (6.90%) | 12 (41.38%) | 15 (51.72%) | **<0.001** | 16.542 (3.299-82.948) | | **<0.001** |
| hyomandibular down | Control | 28 | 1.82 | 5 (17.86%) | 23 (82.14%) | 0 (0%) |  | 1 | |  |
|  | VitD3 | 29 | 2.41 | 3 (10.35%) | 11 (37.93%) | 15 (51.72%) | **<0.001** | 11.226 (2.794-45.400) | | **<0.001** |
| hyomandibular up | Control | 28 | 1.75 | 7 (25%) | 21 (75%) | 0 (0%) |  | 1 | |  |
|  | VitD3 | 29 | 2.54 | 3 (10.35%) | 7 (24.14%) | 19 (65.52%) | **<0.001** | 19.373 (4.695-79.936) | | **<0.001** |
