# Supplementary material for: Zebrafish Bone and General Physiology Are Differently Affected by Hormones or Changes in Gravity
Source: PLoS One. 2015 Jun 10;10(6):e0126928. doi: 10.1371/journal.pone.0126928 (PMC4465622; doi:10.1371/journal.pone.0126928)
Supplement: S3 Table — (A) The bone structures distributed in 2 categories (early and advanced ossification) (B) The bone structures distributed in 3 categories (absent, early and advanced ossification) (DOCX) [file pone.0126928.s010.docx]

Table S3

A

|  |  |  |  | Score of ossification (Y) | | X² pearson | Logistic regression | |
| --- | --- | --- | --- | --- | --- | --- | --- | --- |
| Structures | Treat | N | Mean | early | advanced | p-value | OR (IC 95%) | p-value |
| branchiostegal ray1 down | Control | 29 | 1.00 | 0 (0%) | 29 (100%) |  | 1 |  |
|  | PTH | 27 | 0.93 | 2 (7.41%) | 25 (92.59%) | 0.136 | / | 0.995 |
| branchiostegal ray1 up | Control | 29 | 1.00 | 0 (0%) | 29 (100%) |  | 1 |  |
|  | PTH | 27 | 0.85 | 4 (14.81%) | 23 (85.19%) | **0.031** | / | 0.995 |
| entopterygoid down | Control | 29 | 0.97 | 1 (3.45%) | 28 (96.55%) |  | 1 |  |
|  | PTH | 27 | 0.41 | 16 (59.26%) | 11 (40.74%) | **<0.001** | 0.025 (0.003-0.208) | **<0.001** |
| entopterygoid up | Control | 29 | 0.97 | 1 (3.45%) | 28 (96.55%) |  | 1 |  |
|  | PTH | 27 | 0.41 | 16 (59.26%) | 11 (40.74%) | **<0.001** | 0.025 (0.003-0.208) | **<0.001** |

B

|  |  |  |  | Score of ossification (Y) | | | X² pearson | Ordinal logistic regression | |
| --- | --- | --- | --- | --- | --- | --- | --- | --- | --- |
| Structures | Treat | N | Mean | absence | early | advanced | p-value | OR (IC 95%) | p-value |
| anguloarticular down | Control | 29 | 1.28 | 8 (27.59%) | 5 (17.24%) | 16 (55.17%) |  | 1 |  |
|  | PTH | 27 | 0.11 | 25 (92.60%) | 1 (3.7%) | 1 (3.7%) | **<0.001** | 0.031 (0.006-0.1577) | **<0.001** |
| anguloarticular up | Control | 29 | 1.52 | 4 (13.79%) | 6 (20.69%) | 19 (65.52%) |  | 1 |  |
|  | PTH | 27 | 0.04 | 26 (96.3%) | 1 (3.7%) | 0 (0%) | **<0.001** | 0.006 (0.001-0.055) | **<0.001** |
| branchiostegal ray2 down | Control | 29 | 1.03 | 6 (20.69%) | 16 (55.17%) | 7 (24.14%) |  | 1 |  |
|  | PTH | 27 | 0.19 | 22 (81.48%) | 5 (18.52%) | 0 (0%) | **<0.001** | 0.054 (0.014-0.201) | **<0.001** |
| branchiostegal ray2 up | Control | 29 | 1.17 | 4 (13.79%) | 16 (55.17%) | 9 (31.04%) |  | 1 |  |
|  | PTH | 27 | 0.30 | 19 (70.37%) | 8 (29.63%) | 0 (0%) | **<0.001** | 0.055 (0.015-0.207) | **<0.001** |
| ceratohyal down | Control | 29 | 1.66 | 2 (6.90%) | 6 (20.69%) | 21 (72.41%) |  | 1 |  |
|  | PTH | 27 | 0.41 | 21 (77.78%) | 1 (3.7%) | 5 (18.52%) | **<0.001** | 0.047 (0.013-0.169) | **<0.001** |
| ceratohyal up | Control | 29 | 1.66 | 3 (10.35%) | 4 (13.79%) | 22 (75.86%) |  | 1 |  |
|  | PTH | 27 | 0.44 | 19 (70.37%) | 4 (14.81%) | 4 (14.81%) | **<0.001** | 0.052 (0.015-0.183) | **<0.001** |
| dentary down | Control | 29 | 1.79 | 0 (0%) | 6 (20.69%) | 23 (79.31%) |  | 1 |  |
|  | PTH | 27 | 0.78 | 8 (29.63%) | 17 (62.96%) | 2 (7.41%) | **<0.001** | 0.018 (0.003-0.010) | **<0.001** |
| dentary up | Control | 29 | 1.79 | 0 (0%) | 6 (20.69%) | 23 (79.31%) |  | 1 |  |
|  | PTH | 27 | 0.70 | 10 (37.04%) | 15 (55.55%) | 2 (7.41%) | **<0.001** | 0.018 (0.003-0.095) | **<0.001** |
| hyomandibular down | Control | 29 | 1.86 | 0 (0%) | 4 (13.79%) | 25 (86.21%) |  | 1 |  |
|  | PTH | 27 | 0.93 | 12 (44.44%) | 5 (18.52%) | 10 (37.04%) | **<0.001** | 0.075 (0.020-0.282) | **<0.001** |
| hyomandibular up | Control | 29 | 1.86 | 0 (0%) | 4 (13.79%) | 25 (86.21%) |  | 1 |  |
|  | PTH | 27 | 0.96 | 12 (44.44%) | 4 (14.81%) | 11 (40.74%) | **<0.001** | 0.087 (0.023-0.323) | **<0.001** |
| maxilla down | Control | 29 | 1.93 | 1 (3.45%) | 0 (0%) | 28 (96.55%) |  | 1 |  |
|  | PTH | 27 | 0.93 | 11 (40.74%) | 7 (25.93%) | 9 (33.33%) | **<0.001** | 0.019 (0.002-0.163) | **<0.001** |
| maxilla up | Control | 29 | 1.93 | 1 (3.45%) | 0 (0%) | 28 (96.55%) |  | 1 |  |
|  | PTH | 27 | 1.00 | 8 (29.63%) | 11 (40.74%) | 8 (29.63%) | **<0.001** | 0.017 (0.002-0.142) | **<0.001** |
