# Supplementary material for: Zebrafish Bone and General Physiology Are Differently Affected by Hormones or Changes in Gravity
Source: PLoS One. 2015 Jun 10;10(6):e0126928. doi: 10.1371/journal.pone.0126928 (PMC4465622; doi:10.1371/journal.pone.0126928)
Supplement: S4 Table — The indicates the human homolog of the gene, its "Entrez" gene name, the log ratio of VitD3-treated larvae compared to control, the presence of duplicate probes on the microarray (D) and the type of protein it encodes. Genes are arranged according to their type and in alphabetical order. (DOCX) [file pone.0126928.s011.docx]

| Symbol | **Entrez Gene Name** | **Log Ratio VitD3** | **p-value** | **N** | **Type(s)** |
| --- | --- | --- | --- | --- | --- |
| A2M | alpha-2-macroglobulin | -1.165 | 5.01E-02 | D | transporter |
| A2M | alpha-2-macroglobulin | -1.793 | 6.14E-02 | D | transporter |
| ACTR6 | ARP6 actin-related protein 6 homolog (yeast) | -0.644 | 2.70E-02 |  | transporter |
| AP1S1 | adaptor-related protein complex 1. sigma 1 subunit | 0.736 | 5.82E-02 |  | transporter |
| APOA4 | apolipoprotein A-IV | -1.756 | 1.63E-02 | D | transporter |
| APOA4 | apolipoprotein A-IV | -0.647 | 8.52E-02 | D | transporter |
| APOA4 | apolipoprotein A-IV | -1.392 | 1.22E-02 | D | transporter |
| APOA4 | apolipoprotein A-IV | -0.681 | 9.49E-02 | D | transporter |
| ATP1A1 | ATPase. Na+/K+ transporting. alpha 1 polypeptide | -0.385 | 7.00E-02 |  | transporter |
| ATP2B3 | ATPase. Ca++ transporting. plasma membrane 3 | 0.425 | 7.17E-02 |  | transporter |
| ATP9B | ATPase. class II. type 9B | 0.431 | 5.68E-02 |  | transporter |
| CACNA2D2 | calcium channel. voltage-dependent. alpha 2/delta subunit 2 | 0.384 | 4.79E-02 |  | ion channel |
| CNGA3 | cyclic nucleotide gated channel alpha 3 | -0.613 | 4.55E-02 |  | ion channel |
| FABP2 | fatty acid binding protein 2. intestinal | -0.752 | 5.78E-02 | D | transporter |
| FABP2 | fatty acid binding protein 2. intestinal | -0.805 | 3.76E-02 | D | transporter |
| FOLR1 | folate receptor 1 (adult) | -1.087 | 2.01E-02 | D | transporter |
| FOLR1 | folate receptor 1 (adult) | -0.896 | 3.04E-02 | D | transporter |
| GJB3 | gap junction protein. beta 3. 31kDa | -0.483 | 6.57E-02 |  | transporter |
| HBZ | hemoglobin. zeta | -0.594 | 4.10E-02 | D | transporter |
| HBZ | hemoglobin. zeta | -0.488 | 3.19E-02 | D | transporter |
| HBZ | hemoglobin. zeta | -0.629 | 9.27E-02 | D | transporter |
| HBZ | hemoglobin. zeta | -0.669 | 8.67E-02 | D | transporter |
| HBZ | hemoglobin. zeta | -0.673 | 9.16E-02 | D | transporter |
| KCNMB2 | potassium large conductance calcium-activated channel. subfamily M. beta member 2 | -0.630 | 5.61E-02 |  | ion channel |
| LDLR | low density lipoprotein receptor | -0.530 | 3.25E-02 |  | transporter |
| MTTP | microsomal triglyceride transfer protein | -0.512 | 8.32E-02 |  | transporter |
| PDZD3 | PDZ domain containing 3 | -0.903 | 5.77E-02 |  | transporter |
| PEA15 | phosphoprotein enriched in astrocytes 15 | 0.408 | 3.50E-02 |  | transporter |
| PLLP | plasmolipin | -0.428 | 5.22E-02 |  | transporter |
| Rrbp1 | ribosome binding protein 1 | -0.401 | 2.77E-02 |  | transporter |
| SCN4B | sodium channel. voltage-gated. type IV. beta subunit | -0.412 | 3.16E-02 |  | ion channel |
| SERINC5 | serine incorporator 5 | 0.379 | 5.07E-02 |  | transporter |
| SLC10A3 | solute carrier family 10. member 3 | 0.474 | 9.07E-03 |  | transporter |
| SLC11A2 | solute carrier family 11 (proton-coupled divalent metal ion transporter). member 2 | 0.401 | 3.39E-02 |  | transporter |
| SLC16A2 | solute carrier family 16. member 2 (thyroid hormone transporter) | 0.834 | 5.93E-02 |  | transporter |
| SLC25A15 | solute carrier family 25 (mitochondrial carrier; ornithine transporter) member 15 | 0.498 | 4.11E-02 |  | transporter |
| SLC25A43 | solute carrier family 25. member 43 | -0.686 | 9.15E-02 |  | transporter |
| SLC26A3 | solute carrier family 26 (anion exchanger). member 3 | -0.929 | 2.80E-02 |  | transporter |
| SLC27A2 | solute carrier family 27 (fatty acid transporter). member 2 | -0.549 | 2.12E-02 |  | transporter |
| SLC28A2 | solute carrier family 28 (concentrative nucleoside transporter). member 2 | -0.868 | 3.93E-02 | D | transporter |
| SLC28A2 | solute carrier family 28 (concentrative nucleoside transporter). member 2 | -0.526 | 7.42E-02 | D | transporter |
| SLC2A2 | solute carrier family 2 (facilitated glucose transporter). member 2 | -0.543 | 2.87E-02 | D | transporter |
| SLC2A2 | solute carrier family 2 (facilitated glucose transporter). member 2 | -0.830 | 8.52E-02 | D | transporter |
| SLC35A1 | solute carrier family 35 (CMP-sialic acid transporter). member A1 | 0.420 | 6.18E-02 |  | transporter |
| SLC37A4 | solute carrier family 37 (glucose-6-phosphate transporter). member 4 | -0.400 | 9.73E-02 |  | transporter |
| SLC43A1 | solute carrier family 43 (amino acid system L transporter). member 1 | -1.042 | 2.47E-02 | D | transporter |
| SLC43A1 | solute carrier family 43 (amino acid system L transporter). member 1 | -0.998 | 1.09E-02 | D | transporter |
| SLC43A1 | solute carrier family 43 (amino acid system L transporter). member 1 | -1.069 | 3.46E-02 | D | transporter |
| SLC43A1 | solute carrier family 43 (amino acid system L transporter). member 1 | -1.074 | 3.80E-02 | D | transporter |
| SLC5A2 | solute carrier family 5 (sodium/glucose cotransporter). member 2 | -0.451 | 5.70E-02 |  | transporter |
| SLC5A9 | solute carrier family 5 (sodium/sugar cotransporter). member 9 | -0.744 | 2.82E-02 |  | transporter |
| SLC6A18 | solute carrier family 6 (neutral amino acid transporter). member 18 | -0.461 | 2.93E-02 |  | transporter |
| SLC6A19 | solute carrier family 6 (neutral amino acid transporter). member 19 | -0.769 | 2.22E-02 |  | transporter |
| SLC6A9 | solute carrier family 6 (neurotransmitter transporter. glycine). member 9 | 0.390 | 6.83E-02 |  | transporter |
| SLC7A3 | solute carrier family 7 (cationic amino acid transporter. y+ system). member 3 | 0.465 | 7.70E-02 |  | transporter |
| SYT15 | synaptotagmin XV | -0.380 | 5.80E-02 |  | transporter |
| TCN2 | transcobalamin II | -0.476 | 5.10E-02 |  | transporter |
| TF | transferrin | -1.122 | 1.45E-02 | D | transporter |
| TF | transferrin | -1.179 | 2.34E-02 | D | transporter |
| TF | transferrin | -1.289 | 9.25E-03 | D | transporter |
| TF | transferrin | -0.959 | 1.04E-02 | D | transporter |
| TF | transferrin | -1.132 | 1.15E-02 | D | transporter |
| TF | transferrin | -1.152 | 3.26E-02 | D | transporter |
| TF | transferrin | -1.138 | 9.54E-03 | D | transporter |
| Tmed11 | transmembrane emp24 protein transport domain containing | -0.717 | 3.17E-02 |  | transporter |
| TTPA | tocopherol (alpha) transfer protein | -0.426 | 9.78E-02 |  | transporter |
| TTYH3 | tweety family member 3 | 0.402 | 7.16E-02 |  | ion channel |
| ZP3 | zona pellucida glycoprotein 3 (sperm receptor) | -0.788 | 3.73E-02 |  | transporter |
| ABRA | actin-binding Rho activating protein | -0.848 | 7.28E-02 |  | transcription regulator |
| ANKRD33 | ankyrin repeat domain 33 | -0.547 | 7.27E-02 |  | transcription regulator |
| ATF4 | activating transcription factor 4 | 0.453 | 9.66E-02 |  | transcription regulator |
| BCL6 | B-cell CLL/lymphoma 6 | 0.525 | 4.10E-02 |  | transcription regulator |
| CALCOCO1 | calcium binding and coiled-coil domain 1 | -1.011 | 8.40E-02 |  | transcription regulator |
| CNBP | CCHC-type zinc finger. nucleic acid binding protein | -0.585 | 2.01E-02 | D | transcription regulator |
| CNBP | CCHC-type zinc finger. nucleic acid binding protein | -0.531 | 3.29E-02 | D | transcription regulator |
| CNBP | CCHC-type zinc finger. nucleic acid binding protein | -0.468 | 5.01E-02 | D | transcription regulator |
| ETV4 | ets variant 4 | 0.423 | 4.87E-02 |  | transcription regulator |
| FOSB | FBJ murine osteosarcoma viral oncogene homolog B | -0.988 | 7.37E-02 |  | transcription regulator |
| FOXK1 | forkhead box K1 | 0.426 | 6.79E-02 |  | transcription regulator |
| FOXO3 | forkhead box O3 | -0.386 | 4.34E-02 | D | transcription regulator |
| FOXO3 | forkhead box O3 | -0.507 | 7.90E-02 | D | transcription regulator |
| FOXO3 | forkhead box O3 | -0.401 | 4.36E-02 | D | transcription regulator |
| FOXQ1 | forkhead box Q1 | 0.692 | 4.92E-02 |  | transcription regulator |
| GATA6 | GATA binding protein 6 | -0.460 | 8.38E-02 |  | transcription regulator |
| GSPT1 | G1 to S phase transition 1 | 0.438 | 8.68E-02 |  | translation regulator |
| HIF3A | hypoxia inducible factor 3. alpha subunit | 0.476 | 2.53E-02 | D | transcription regulator |
| HIF3A | hypoxia inducible factor 3. alpha subunit | 0.482 | 6.22E-02 | D | transcription regulator |
| HIF3A | hypoxia inducible factor 3. alpha subunit | 0.387 | 8.16E-02 | D | transcription regulator |
| KLF11 | Kruppel-like factor 11 | 1.451 | 2.42E-02 | D | transcription regulator |
| KLF11 | Kruppel-like factor 11 | 2.629 | 8.99E-03 | D | transcription regulator |
| KLF13 | Kruppel-like factor 13 | 1.185 | 2.28E-02 | D | transcription regulator |
| KLF13 | Kruppel-like factor 13 | 1.032 | 3.64E-02 | D | transcription regulator |
| MEIS2 | Meis homeobox 2 | 0.393 | 3.99E-02 |  | transcription regulator |
| MYC | v-myc avian myelocytomatosis viral oncogene homolog | -0.443 | 8.64E-02 |  | transcription regulator |
| MYOG | myogenin (myogenic factor 4) | -0.545 | 1.07E-02 |  | transcription regulator |
| NCOA4 | nuclear receptor coactivator 4 | 0.464 | 3.89E-02 | D | transcription regulator |
| NCOA4 | nuclear receptor coactivator 4 | 0.467 | 3.06E-02 | D | transcription regulator |
| NR0B2 | nuclear receptor subfamily 0. group B. member 2 | 0.631 | 2.65E-02 |  | ligand-dependent nuclear receptor |
| NRARP | NOTCH-regulated ankyrin repeat protein | 0.528 | 8.64E-02 | D | transcription regulator |
| NRARP | NOTCH-regulated ankyrin repeat protein | 0.473 | 8.63E-02 | D | transcription regulator |
| PPARA | peroxisome proliferator-activated receptor alpha | -0.556 | 2.94E-02 |  | ligand-dependent nuclear receptor |
| RYBP | RING1 and YY1 binding protein | 0.707 | 4.61E-02 | D | transcription regulator |
| RYBP | RING1 and YY1 binding protein | 0.548 | 7.74E-02 | D | transcription regulator |
| SMARCC1 | SWI/SNF related. matrix associated. actin dependent regulator of chromatin. subfamily c. member 1 | 0.516 | 8.92E-02 | D | transcription regulator |
| SMARCC1 | SWI/SNF related. matrix associated. actin dependent regulator of chromatin. subfamily c. member 1 | 0.578 | 6.43E-02 | D | transcription regulator |
| SOX4 | SRY (sex determining region Y)-box 4 | 0.459 | 4.76E-02 | D | transcription regulator |
| SOX4 | SRY (sex determining region Y)-box 4 | 0.395 | 2.72E-02 | D | transcription regulator |
| SOX4 | SRY (sex determining region Y)-box 4 | 0.480 | 9.74E-02 | D | transcription regulator |
| STAT3 | signal transducer and activator of transcription 3 (acute-phase response factor) | 0.471 | 1.22E-02 |  | transcription regulator |
| TWIST1 | twist family bHLH transcription factor 1 | -0.416 | 7.22E-02 |  | transcription regulator |
| ZNF423 | zinc finger protein 423 | 0.557 | 5.30E-02 |  | transcription regulator |
| ADRB2 | adrenoceptor beta 2. surface | -0.603 | 2.52E-02 |  | G-protein coupled receptor |
| CD36 | CD36 molecule (thrombospondin receptor) | -0.421 | 7.09E-02 |  | transmembrane receptor |
| CUBN | cubilin (intrinsic factor-cobalamin receptor) | -0.444 | 3.16E-02 |  | transmembrane receptor |
| GPC1 | glypican 1 | 0.535 | 2.44E-02 | D | transmembrane receptor |
| GPC1 | glypican 1 | 0.376 | 2.86E-02 | D | transmembrane receptor |
| GPR112 | G protein-coupled receptor 112 | -0.595 | 7.88E-02 |  | G-protein coupled receptor |
| GPR139 | G protein-coupled receptor 139 | 0.556 | 3.32E-03 |  | G-protein coupled receptor |
| ITGB4 | integrin. beta 4 | 0.535 | 2.90E-02 |  | transmembrane receptor |
| LYVE1 | lymphatic vessel endothelial hyaluronan receptor 1 | 0.806 | 6.75E-02 |  | transmembrane receptor |
| OPN1LW | opsin 1 (cone pigments). long-wave-sensitive | -1.538 | 3.33E-03 |  | G-protein coupled receptor |
| OPRL1 | opiate receptor-like 1 | -0.613 | 4.39E-02 |  | G-protein coupled receptor |
| PGRMC1 | progesterone receptor membrane component 1 | -0.388 | 4.56E-02 |  | transmembrane receptor |
| RELT | RELT tumor necrosis factor receptor | 0.460 | 6.03E-02 |  | transmembrane receptor |
| C5 | complement component 5 | 0.543 | 8.88E-04 |  | cytokine |
| EBI3 | Epstein-Barr virus induced 3 | -1.268 | 3.77E-02 | D | cytokine |
| EBI3 | Epstein-Barr virus induced 3 | -1.512 | 3.66E-02 | D | cytokine |
| IGF2 | insulin-like growth factor 2 (somatomedin A) | 0.761 | 9.30E-03 |  | growth factor |
| PDGFC | platelet derived growth factor C | 0.592 | 8.87E-02 |  | growth factor |
| BAIAP2 | BAI1-associated protein 2 | 0.381 | 3.52E-03 |  | kinase |
| BCKDK | branched chain ketoacid dehydrogenase kinase | -0.413 | 7.09E-02 |  | kinase |
| CDKN3 | cyclin-dependent kinase inhibitor 3 | -0.445 | 6.96E-02 |  | phosphatase |
| CHKA | choline kinase alpha | -0.509 | 4.48E-02 |  | kinase |
| EEF2K | eukaryotic elongation factor-2 kinase | -0.414 | 2.01E-02 |  | kinase |
| FBP1 | fructose-1.6-bisphosphatase 1 | -0.551 | 8.30E-02 | D | phosphatase |
| FBP1 | fructose-1.6-bisphosphatase 1 | -0.384 | 8.33E-02 | D | phosphatase |
| FBP1 | fructose-1.6-bisphosphatase 1 | -0.556 | 7.13E-02 | D | phosphatase |
| GNE | glucosamine (UDP-N-acetyl)-2-epimerase/N-acetylmannosamine kinase | -0.400 | 3.30E-02 |  | kinase |
| GRK7 | G protein-coupled receptor kinase 7 | -0.752 | 6.95E-02 | D | kinase |
| GRK7 | G protein-coupled receptor kinase 7 | -0.618 | 8.45E-02 | D | kinase |
| LPIN1 | lipin 1 | -1.165 | 6.70E-02 | D | phosphatase |
| LPIN1 | lipin 1 | -1.208 | 6.14E-02 | D | phosphatase |
| LPIN1 | lipin 1 | -1.370 | 6.74E-02 | D | phosphatase |
| MEX3B | mex-3 RNA binding family member B | 0.548 | 8.21E-02 | D | kinase |
| MEX3B | mex-3 RNA binding family member B | 0.508 | 6.91E-02 | D | kinase |
| NRBP2 | nuclear receptor binding protein 2 | -0.477 | 7.35E-02 |  | kinase |
| OBSCN | obscurin. cytoskeletal calmodulin and titin-interacting RhoGEF | -0.382 | 5.35E-02 |  | kinase |
| PCK1 | phosphoenolpyruvate carboxykinase 1 (soluble) | 0.511 | 8.54E-02 | D | kinase |
| PCK1 | phosphoenolpyruvate carboxykinase 1 (soluble) | 0.577 | 8.54E-02 | D | kinase |
| PCK2 | phosphoenolpyruvate carboxykinase 2 (mitochondrial) | -0.632 | 1.94E-02 | D | kinase |
| PCK2 | phosphoenolpyruvate carboxykinase 2 (mitochondrial) | -0.668 | 2.59E-02 | D | kinase |
| PDK2 | pyruvate dehydrogenase kinase. isozyme 2 | -0.622 | 1.45E-02 | D | kinase |
| PDK2 | pyruvate dehydrogenase kinase. isozyme 2 | -0.700 | 3.27E-02 | D | kinase |
| PDK2 | pyruvate dehydrogenase kinase. isozyme 2 | -0.475 | 5.08E-02 | D | kinase |
| PDK4 | pyruvate dehydrogenase kinase. isozyme 4 | -0.455 | 4.37E-02 |  | kinase |
| PHKA1 | phosphorylase kinase. alpha 1 (muscle) | -0.449 | 5.97E-02 |  | kinase |
| PIK3R1 | phosphoinositide-3-kinase. regulatory subunit 1 (alpha) | -0.450 | 9.29E-02 |  | kinase |
| PIM1 | pim-1 oncogene | 0.678 | 2.57E-02 |  | kinase |
| PKLR | pyruvate kinase. liver and RBC | -0.759 | 6.79E-02 | D | kinase |
| PKLR | pyruvate kinase. liver and RBC | -0.688 | 8.46E-02 | D | kinase |
| PKLR | pyruvate kinase. liver and RBC | -0.701 | 9.77E-02 | D | kinase |
| PKLR | pyruvate kinase. liver and RBC | -0.874 | 1.17E-02 | D | kinase |
| PPM1H | protein phosphatase. Mg2+/Mn2+ dependent. 1H | 0.446 | 2.87E-02 |  | phosphatase |
| PPP4C | protein phosphatase 4. catalytic subunit | 0.401 | 7.85E-03 |  | phosphatase |
| PTP4A3 | protein tyrosine phosphatase type IVA. member 3 | 0.583 | 4.67E-02 | D | phosphatase |
| PTP4A3 | protein tyrosine phosphatase type IVA. member 3 | 0.478 | 6.33E-02 | D | phosphatase |
| STK19 | serine/threonine kinase 19 | -0.456 | 3.89E-02 |  | kinase |
| STK39 | serine threonine kinase 39 | -0.426 | 6.98E-03 | D | kinase |
| STK39 | serine threonine kinase 39 | -0.545 | 4.04E-02 | D | kinase |
| TPK1 | thiamin pyrophosphokinase 1 | -0.481 | 1.69E-02 |  | kinase |
| TTN | titin | -0.522 | 6.08E-03 | D | kinase |
| TTN | titin | -0.424 | 6.24E-02 | D | kinase |
| TTN | titin | -0.578 | 3.95E-02 | D | kinase |
| TTN | titin | -0.375 | 2.22E-02 | D | kinase |
| TWF2 | twinfilin actin-binding protein 2 | -0.418 | 5.80E-02 |  | kinase |
| ACE2 | angiotensin I converting enzyme 2 | -0.407 | 5.38E-02 |  | peptidase |
| ANPEP | alanyl (membrane) aminopeptidase | -0.444 | 1.22E-02 |  | peptidase |
| CNDP2 | CNDP dipeptidase 2 (metallopeptidase M20 family) | -0.540 | 4.32E-02 |  | peptidase |
| CPA2 | carboxypeptidase A2 (pancreatic) | -0.640 | 8.02E-02 |  | peptidase |
| CTRB2 | chymotrypsinogen B2 | -0.791 | 4.38E-02 |  | peptidase |
| CTSH | cathepsin H | -0.407 | 3.05E-02 |  | peptidase |
| CTSS | cathepsin S | 0.377 | 4.77E-02 |  | peptidase |
| DPP4 | dipeptidyl-peptidase 4 | -0.628 | 4.78E-02 |  | peptidase |
| EPHX1 | epoxide hydrolase 1. microsomal (xenobiotic) | -0.725 | 9.10E-03 | D | peptidase |
| EPHX1 | epoxide hydrolase 1. microsomal (xenobiotic) | -0.625 | 1.93E-02 | D | peptidase |
| F7 | coagulation factor VII (serum prothrombin conversion accelerator) | -0.569 | 6.00E-02 |  | peptidase |
| F9 | coagulation factor IX | 0.668 | 9.22E-03 |  | peptidase |
| HABP2 | hyaluronan binding protein 2 | -1.004 | 6.57E-02 |  | peptidase |
| LONP1 | lon peptidase 1. mitochondrial | 0.447 | 7.49E-02 |  | peptidase |
| MMP11 | matrix metallopeptidase 11 (stromelysin 3) | 0.883 | 5.26E-03 |  | peptidase |
| PAPPA | pregnancy-associated plasma protein A. pappalysin 1 | 0.723 | 3.21E-02 | D | peptidase |
| PAPPA | pregnancy-associated plasma protein A. pappalysin 1 | 0.588 | 4.75E-02 | D | peptidase |
| PEPD | peptidase D | -0.461 | 6.22E-02 | D | peptidase |
| PEPD | peptidase D | -0.407 | 4.87E-03 | D | peptidase |
| TMPRSS13 | transmembrane protease. serine 13 | 0.378 | 3.67E-02 |  | peptidase |
| USP14 | ubiquitin specific peptidase 14 (tRNA-guanine transglycosylase) | 0.491 | 5.08E-03 |  | peptidase |
| USP37 | ubiquitin specific peptidase 37 | 0.387 | 8.75E-02 |  | peptidase |
| AASDHPPT | aminoadipate-semialdehyde dehydrogenase-phosphopantetheinyl transferase | -0.541 | 4.88E-02 | D | enzyme |
| AASDHPPT | aminoadipate-semialdehyde dehydrogenase-phosphopantetheinyl transferase | -0.609 | 5.49E-02 | D | enzyme |
| ACAA1 | acetyl-CoA acyltransferase 1 | -0.413 | 2.26E-02 | D | enzyme |
| ACAA1 | acetyl-CoA acyltransferase 1 | -0.473 | 3.14E-02 | D | enzyme |
| ACADS | acyl-CoA dehydrogenase. C-2 to C-3 short chain | -0.389 | 2.18E-02 |  | enzyme |
| ACOX1 | acyl-CoA oxidase 1. palmitoyl | -0.845 | 6.53E-02 |  | enzyme |
| ACSF2 | acyl-CoA synthetase family member 2 | -0.494 | 2.08E-02 |  | enzyme |
| ACSL3 | acyl-CoA synthetase long-chain family member 3 | 0.467 | 6.01E-02 |  | enzyme |
| ACTA1 | actin. alpha 1. skeletal muscle | -0.526 | 3.90E-02 | D | other |
| ACTA1 | actin. alpha 1. skeletal muscle | -0.621 | 8.67E-02 | D | other |
| ACTA1 | actin. alpha 1. skeletal muscle | -0.628 | 3.00E-02 | D | other |
| ACTA1 | actin. alpha 1. skeletal muscle | -0.446 | 4.16E-02 | D | other |
| ACTA1 | actin. alpha 1. skeletal muscle | -0.694 | 7.54E-02 | D | other |
| ACTA1 | actin. alpha 1. skeletal muscle | -0.591 | 1.61E-02 | D | other |
| AGMAT | agmatine ureohydrolase (agmatinase) | -0.429 | 5.74E-02 |  | enzyme |
| AKR1B1 | aldo-keto reductase family 1. member B1 (aldose reductase) | -0.385 | 3.86E-02 |  | enzyme |
| ALAS2 | aminolevulinate. delta-. synthase 2 | -0.682 | 4.22E-02 | D | enzyme |
| ALAS2 | aminolevulinate. delta-. synthase 2 | -0.565 | 7.58E-02 | D | enzyme |
| ALDH4A1 | aldehyde dehydrogenase 4 family. member A1 | -0.460 | 1.67E-02 |  | enzyme |
| ANXA2 | annexin A2 | -0.786 | 7.11E-02 | D | other |
| ANXA2 | annexin A2 | -0.597 | 3.78E-02 | D | other |
| ANXA2 | annexin A2 | -0.660 | 6.56E-02 | D | other |
| AOC1 | amine oxidase. copper containing 1 | -0.684 | 4.09E-02 |  | enzyme |
| ARMC2 | armadillo repeat containing 2 | -0.495 | 7.47E-02 |  | other |
| ARRDC2 | arrestin domain containing 2 | 0.376 | 3.89E-02 |  | other |
| ATAD2B | ATPase family. AAA domain containing 2B | 0.446 | 5.54E-02 |  | other |
| ATAD3A | ATPase family. AAA domain containing 3A | 0.380 | 5.92E-03 |  | other |
| BCAT2 | branched chain amino-acid transaminase 2. mitochondrial | -0.638 | 2.30E-02 | D | enzyme |
| BCAT2 | branched chain amino-acid transaminase 2. mitochondrial | -0.636 | 4.97E-02 | D | enzyme |
| BCAT2 | branched chain amino-acid transaminase 2. mitochondrial | -0.453 | 8.24E-02 | D | enzyme |
| BNIP3 | BCL2/adenovirus E1B 19kDa interacting protein 3 | -0.599 | 4.91E-02 |  | other |
| BOC | BOC cell adhesion associated. oncogene regulated | -0.556 | 2.27E-02 |  | other |
| C4orf33 | chromosome 4 open reading frame 33 | -0.376 | 3.97E-02 |  | other |
| C6 | complement component 6 | 0.418 | 5.60E-03 |  | other |
| C7 | complement component 7 | 0.665 | 8.71E-02 |  | other |
| CA7 | carbonic anhydrase VII | -0.566 | 4.59E-02 |  | enzyme |
| CAD | carbamoyl-phosphate synthetase 2. aspartate transcarbamylase. and dihydroorotase | 0.510 | 9.35E-02 |  | enzyme |
| CADPS | Ca++-dependent secretion activator | -0.443 | 8.96E-02 |  | other |
| CAT | catalase | -0.515 | 1.71E-02 | D | enzyme |
| CAT | catalase | -0.423 | 8.00E-02 | D | enzyme |
| CCBL2 | cysteine conjugate-beta lyase 2 | -0.381 | 4.82E-02 |  | enzyme |
| CCDC125 | coiled-coil domain containing 125 | -0.380 | 7.53E-02 |  | other |
| CEACAM20 | carcinoembryonic antigen-related cell adhesion molecule 20 | -0.549 | 6.26E-02 |  | other |
| CEP85 | centrosomal protein 85kDa | -0.511 | 9.95E-02 |  | other |
| CES1 | carboxylesterase 1 | -2.047 | 4.85E-02 | D | enzyme |
| CES1 | carboxylesterase 1 | -2.244 | 4.64E-02 | D | enzyme |
| CFH | complement factor H | -1.151 | 7.01E-02 |  | other |
| CHD8 | chromodomain helicase DNA binding protein 8 | 0.382 | 3.18E-02 |  | enzyme |
| CHPT1 | choline phosphotransferase 1 | -0.441 | 2.47E-02 |  | enzyme |
| CHST2 | carbohydrate (N-acetylglucosamine-6-O) sulfotransferase 2 | -0.429 | 7.11E-02 |  | enzyme |
| CISH | cytokine inducible SH2-containing protein | -1.615 | 7.77E-02 | D | other |
| CISH | cytokine inducible SH2-containing protein | -1.269 | 3.92E-02 | D | other |
| CISH | cytokine inducible SH2-containing protein | -1.294 | 5.72E-02 | D | other |
| CISH | cytokine inducible SH2-containing protein | -1.261 | 4.79E-02 | D | other |
| CISH | cytokine inducible SH2-containing protein | -1.779 | 4.33E-02 | D | other |
| CISH | cytokine inducible SH2-containing protein | -1.260 | 3.41E-02 | D | other |
| CISH | cytokine inducible SH2-containing protein | -0.983 | 6.59E-02 | D | other |
| CLEC4E | C-type lectin domain family 4. member E | 0.422 | 7.50E-02 | D | other |
| CLEC4E | C-type lectin domain family 4. member E | 0.443 | 7.01E-02 | D | other |
| COL9A2 | collagen. type IX. alpha 2 | -0.417 | 5.61E-02 |  | other |
| CREB3L3 | cAMP responsive element binding protein 3-like 3 | -0.475 | 1.42E-02 |  | other |
| CROT | carnitine O-octanoyltransferase | -1.574 | 5.98E-02 |  | enzyme |
| CTDSPL | CTD (carboxy-terminal domain. RNA polymerase II. polypeptide A) small phosphatase-like | 0.496 | 1.93E-02 | D | other |
| CTDSPL | CTD (carboxy-terminal domain. RNA polymerase II. polypeptide A) small phosphatase-like | 0.558 | 5.62E-02 | D | other |
| CTH | cystathionase (cystathionine gamma-lyase) | -0.581 | 3.92E-02 | D | enzyme |
| CTH | cystathionase (cystathionine gamma-lyase) | -0.536 | 7.30E-02 | D | enzyme |
| CUZD1 | CUB and zona pellucida-like domains 1 | -0.562 | 7.23E-02 | D | other |
| CUZD1 | CUB and zona pellucida-like domains 1 | -0.629 | 4.81E-02 | D | other |
| CYB5R2 | cytochrome b5 reductase 2 | -0.437 | 2.99E-02 |  | enzyme |
| CYP24A1 | cytochrome P450. family 24. subfamily A. polypeptide 1 | 2.955 | 2.87E-03 | D | enzyme |
| CYP24A1 | cytochrome P450. family 24. subfamily A. polypeptide 1 | 3.160 | 5.18E-03 | D | enzyme |
| CYP26A1 | cytochrome P450. family 26. subfamily A. polypeptide 1 | 0.830 | 5.03E-02 | D | enzyme |
| CYP26A1 | cytochrome P450. family 26. subfamily A. polypeptide 1 | 0.779 | 2.02E-02 | D | enzyme |
| CYP27A1 | cytochrome P450. family 27. subfamily A. polypeptide 1 | -0.477 | 7.42E-02 |  | enzyme |
| Cyp2ac1 | cytochrome P450. family 2. subfamily ac. polypeptide 1 | 0.836 | 1.74E-02 | D | other |
| Cyp2ac1 | cytochrome P450. family 2. subfamily ac. polypeptide 1 | -0.639 | 9.50E-02 | D | other |
| Cyp2ac1 | cytochrome P450. family 2. subfamily ac. polypeptide 1 | 1.134 | 1.52E-02 | D | other |
| Cyp2ac1 | cytochrome P450. family 2. subfamily ac. polypeptide 1 | -0.605 | 1.88E-02 | D | other |
| Cyp2ac1 | cytochrome P450. family 2. subfamily ac. polypeptide 1 | 1.069 | 8.69E-02 | D | other |
| Cyp2g1 | cytochrome P450. family 2. subfamily g. polypeptide 1 | -0.375 | 5.17E-02 | D | enzyme |
| Cyp2g1 | cytochrome P450. family 2. subfamily g. polypeptide 1 | -0.457 | 9.67E-02 | D | enzyme |
| CYP2J2 | cytochrome P450. family 2. subfamily J. polypeptide 2 | -1.015 | 6.16E-02 |  | enzyme |
| CYP3A7 | cytochrome P450. family 3. subfamily A. polypeptide 7 | -0.911 | 8.51E-02 | D | enzyme |
| CYP3A7 | cytochrome P450. family 3. subfamily A. polypeptide 7 | -0.414 | 2.54E-02 | D | enzyme |
| CYP8B1 | cytochrome P450. family 8. subfamily B. polypeptide 1 | -0.686 | 2.20E-02 |  | enzyme |
| D1Pas1 | DNA segment. Chr 1. Pasteur Institute 1 | 0.411 | 1.52E-02 |  | other |
| DAO | D-amino-acid oxidase | -0.464 | 1.11E-02 | D | enzyme |
| DAO | D-amino-acid oxidase | -0.576 | 5.06E-02 | D | enzyme |
| DBT | dihydrolipoamide branched chain transacylase E2 | -0.458 | 2.62E-02 | D | enzyme |
| DBT | dihydrolipoamide branched chain transacylase E2 | -0.422 | 4.47E-02 | D | enzyme |
| DCLRE1B | DNA cross-link repair 1B | -0.422 | 3.50E-02 |  | enzyme |
| DCT | dopachrome tautomerase | -0.431 | 1.24E-02 | D | enzyme |
| DCT | dopachrome tautomerase | -0.410 | 3.93E-02 | D | enzyme |
| DDC | dopa decarboxylase (aromatic L-amino acid decarboxylase) | -0.542 | 9.33E-02 |  | enzyme |
| DDT | D-dopachrome tautomerase | -0.627 | 4.14E-02 |  | enzyme |
| DDX5 | DEAD (Asp-Glu-Ala-Asp) box helicase 5 | 0.576 | 9.21E-02 |  | enzyme |
| DECR1 | 2.4-dienoyl CoA reductase 1. mitochondrial | -0.562 | 4.76E-02 |  | enzyme |
| DHRS13 | dehydrogenase/reductase (SDR family) member 13 | -0.607 | 9.83E-02 |  | enzyme |
| DHTKD1 | dehydrogenase E1 and transketolase domain containing 1 | -1.004 | 5.69E-02 | D | enzyme |
| DHTKD1 | dehydrogenase E1 and transketolase domain containing 1 | -0.830 | 6.43E-02 | D | enzyme |
| DHX32 | DEAH (Asp-Glu-Ala-His) box polypeptide 32 | 0.733 | 2.25E-02 |  | enzyme |
| DIO1 | deiodinase. iodothyronine. type I | -0.573 | 4.26E-03 | D | enzyme |
| DIO1 | deiodinase. iodothyronine. type I | -0.609 | 8.94E-02 | D | enzyme |
| DMGDH | dimethylglycine dehydrogenase | -0.462 | 1.64E-02 |  | enzyme |
| DMRT1 | doublesex and mab-3 related transcription factor 1 | -0.834 | 2.21E-02 |  | other |
| DNAJC4 | DnaJ (Hsp40) homolog. subfamily C. member 4 | -0.446 | 8.95E-02 |  | other |
| DNMT3A | DNA (cytosine-5-)-methyltransferase 3 alpha | 0.516 | 9.87E-03 |  | enzyme |
| DOLPP1 | dolichyldiphosphatase 1 | 0.390 | 4.27E-02 |  | enzyme |
| DPYS | dihydropyrimidinase | -0.402 | 7.06E-02 |  | enzyme |
| ELOVL2 | ELOVL fatty acid elongase 2 | -0.449 | 3.45E-02 |  | enzyme |
| ETNPPL | ethanolamine-phosphate phospho-lyase | -0.751 | 9.08E-02 |  | enzyme |
| FADS6 | fatty acid desaturase 6 | 1.083 | 3.19E-02 |  | enzyme |
| FAM131C | family with sequence similarity 131. member C | 0.503 | 6.11E-02 |  | other |
| FAM13A | family with sequence similarity 13. member A | -0.421 | 2.66E-02 |  | other |
| FAM46C | family with sequence similarity 46. member C | -0.601 | 3.17E-02 | D | other |
| FAM46C | family with sequence similarity 46. member C | -0.443 | 1.38E-02 | D | other |
| FBXO2 | F-box protein 2 | -0.613 | 2.09E-03 |  | enzyme |
| FCGBP | Fc fragment of IgG binding protein | -0.687 | 5.23E-02 |  | other |
| FSTL1 | follistatin-like 1 | 0.455 | 9.56E-03 |  | other |
| GADD45A | growth arrest and DNA-damage-inducible. alpha | 0.396 | 9.17E-02 | D | other |
| GADD45A | growth arrest and DNA-damage-inducible. alpha | -0.941 | 4.59E-02 | D | other |
| GADD45A | growth arrest and DNA-damage-inducible. alpha | -0.844 | 4.88E-02 | D | other |
| GATM | glycine amidinotransferase (L-arginine:glycine amidinotransferase) | 0.435 | 4.89E-02 | D | enzyme |
| GATM | glycine amidinotransferase (L-arginine:glycine amidinotransferase) | 0.377 | 5.33E-02 | D | enzyme |
| GATM | glycine amidinotransferase (L-arginine:glycine amidinotransferase) | 0.417 | 4.60E-02 | D | enzyme |
| GCAT | glycine C-acetyltransferase | -0.904 | 2.06E-02 |  | enzyme |
| GCG | glucagon | -0.492 | 2.42E-02 |  | other |
| GCHFR | GTP cyclohydrolase I feedback regulator | -0.928 | 4.35E-02 |  | other |
| GDA | guanine deaminase | -0.524 | 5.50E-02 |  | enzyme |
| GGCT | gamma-glutamylcyclotransferase | 0.627 | 3.80E-02 |  | enzyme |
| GLDC | glycine dehydrogenase (decarboxylating) | -0.505 | 6.31E-02 | D | enzyme |
| GLDC | glycine dehydrogenase (decarboxylating) | -0.586 | 2.13E-02 | D | enzyme |
| GLDC | glycine dehydrogenase (decarboxylating) | -0.515 | 4.58E-02 | D | enzyme |
| GNG10 | guanine nucleotide binding protein (G protein). gamma 10 | -0.561 | 7.18E-03 |  | enzyme |
| GNPDA2 | glucosamine-6-phosphate deaminase 2 | 0.813 | 2.85E-02 |  | enzyme |
| GOT2 | glutamic-oxaloacetic transaminase 2. mitochondrial | -0.468 | 2.78E-02 |  | enzyme |
| GPT | glutamic-pyruvate transaminase (alanine aminotransferase) | 0.392 | 5.25E-02 | D | enzyme |
| GPT | glutamic-pyruvate transaminase (alanine aminotransferase) | 0.590 | 6.83E-02 | D | enzyme |
| GPT | glutamic-pyruvate transaminase (alanine aminotransferase) | 0.490 | 3.04E-02 | D | enzyme |
| GPT | glutamic-pyruvate transaminase (alanine aminotransferase) | 0.632 | 9.35E-02 | D | enzyme |
| GPX1 | glutathione peroxidase 1 | -0.770 | 2.77E-02 |  | enzyme |
| GRB10 | growth factor receptor-bound protein 10 | -1.053 | 2.88E-02 |  | other |
| GSR | glutathione reductase | 0.377 | 6.52E-02 |  | enzyme |
| GSTK1 | glutathione S-transferase kappa 1 | -0.415 | 1.97E-02 |  | enzyme |
| GSTO1 | glutathione S-transferase omega 1 | -0.470 | 7.39E-02 |  | enzyme |
| Gstt3 | glutathione S-transferase. theta 3 | 0.410 | 1.45E-02 |  | enzyme |
| HADH | hydroxyacyl-CoA dehydrogenase | -0.409 | 1.09E-02 |  | enzyme |
| HAGH | hydroxyacylglutathione hydrolase | -0.501 | 2.77E-02 |  | enzyme |
| HAO2 | hydroxyacid oxidase 2 (long chain) | -0.441 | 6.52E-02 |  | enzyme |
| HGD | homogentisate 1.2-dioxygenase | -0.534 | 7.20E-02 |  | enzyme |
| HMCES | 5-hydroxymethylcytosine (hmC) binding. ES cell-specific | -0.540 | 6.37E-02 |  | other |
| HMGCL | 3-hydroxymethyl-3-methylglutaryl-CoA lyase | -0.374 | 2.07E-02 |  | enzyme |
| HNMT | histamine N-methyltransferase | -0.431 | 1.15E-02 |  | enzyme |
| HPD | 4-hydroxyphenylpyruvate dioxygenase | -0.543 | 8.16E-02 |  | enzyme |
| HSD11B1L | hydroxysteroid (11-beta) dehydrogenase 1-like | -0.698 | 4.07E-02 |  | other |
| HSD11B2 | hydroxysteroid (11-beta) dehydrogenase 2 | -0.408 | 5.70E-02 |  | enzyme |
| HSD17B4 | hydroxysteroid (17-beta) dehydrogenase 4 | -0.450 | 4.12E-02 |  | enzyme |
| HSD3B7 | hydroxy-delta-5-steroid dehydrogenase. 3 beta- and steroid delta-isomerase 7 | -0.406 | 4.16E-02 |  | enzyme |
| HSP90B1 | heat shock protein 90kDa beta (Grp94). member 1 | 0.467 | 7.45E-02 | D | other |
| HSP90B1 | heat shock protein 90kDa beta (Grp94). member 1 | 0.486 | 4.70E-02 | D | other |
| IFRD1 | interferon-related developmental regulator 1 | 0.507 | 3.92E-02 |  | other |
| IGFBP1 | insulin-like growth factor binding protein 1 | 1.077 | 3.38E-02 | D | other |
| IGFBP1 | insulin-like growth factor binding protein 1 | 1.774 | 4.67E-03 | D | other |
| IGFBP1 | insulin-like growth factor binding protein 1 | 1.109 | 2.40E-02 | D | other |
| IGFBP1 | insulin-like growth factor binding protein 1 | 1.120 | 4.72E-02 | D | other |
| IGFBP1 | insulin-like growth factor binding protein 1 | 1.919 | 3.90E-03 | D | other |
| ING5 | inhibitor of growth family. member 5 | -0.734 | 4.22E-02 |  | other |
| INSIG1 | insulin induced gene 1 | 0.616 | 4.79E-02 |  | other |
| IRS1 | insulin receptor substrate 1 | 0.816 | 2.28E-03 |  | enzyme |
| ITIH3 | inter-alpha-trypsin inhibitor heavy chain 3 | -0.514 | 9.64E-02 | D | other |
| ITIH3 | inter-alpha-trypsin inhibitor heavy chain 3 | -0.528 | 5.99E-02 | D | other |
| ITLN1 | intelectin 1 (galactofuranose binding) | -1.274 | 4.88E-02 |  | other |
| JAKMIP1 | janus kinase and microtubule interacting protein 1 | 0.546 | 1.39E-03 |  | other |
| KIAA1324 | KIAA1324 | -0.623 | 8.12E-02 |  | other |
| KRT17 | keratin 17 | 0.499 | 2.06E-02 | D | other |
| KRT17 | keratin 17 | 0.444 | 8.65E-02 | D | other |
| KRT17 | keratin 17 | 0.393 | 7.13E-02 | D | other |
| KRT17 | keratin 17 | 0.520 | 2.04E-02 | D | other |
| LCT | lactase | -1.104 | 3.59E-02 |  | enzyme |
| LECT1 | leukocyte cell derived chemotaxin 1 | -0.532 | 9.60E-02 |  | other |
| LOC285556 | uncharacterized LOC285556 | 0.570 | 8.68E-02 |  | other |
| LOX | lysyl oxidase | 0.459 | 6.99E-02 |  | enzyme |
| LPL | lipoprotein lipase | -0.851 | 6.88E-02 | D | enzyme |
| LPL | lipoprotein lipase | -0.865 | 9.87E-02 | D | enzyme |
| MALRD1 | MAM and LDL receptor class A domain containing 1 | -0.909 | 3.42E-02 |  | other |
| MBOAT4 | membrane bound O-acyltransferase domain containing 4 | -0.404 | 7.76E-02 |  | enzyme |
| MCM7 | minichromosome maintenance complex component 7 | 0.373 | 5.31E-02 |  | enzyme |
| Mettl21e | methyltransferase like 21E | -0.381 | 9.44E-02 |  | other |
| METTL7A | methyltransferase like 7A | -0.512 | 1.11E-02 |  | other |
| MFSD4 | major facilitator superfamily domain containing 4 | -0.447 | 5.27E-02 |  | other |
| MID1 | midline 1 (Opitz/BBB syndrome) | 0.400 | 5.27E-02 |  | other |
| MIOX | myo-inositol oxygenase | 1.245 | 1.48E-02 |  | enzyme |
| MLEC | malectin | -0.396 | 2.20E-02 |  | other |
| MOCS1 | molybdenum cofactor synthesis 1 | -0.755 | 6.52E-02 |  | other |
| MOGAT1 | monoacylglycerol O-acyltransferase 1 | -0.395 | 9.35E-02 | D | enzyme |
| MOGAT1 | monoacylglycerol O-acyltransferase 1 | -0.588 | 4.41E-02 | D | enzyme |
| MOV10L1 | Mov10l1. Moloney leukemia virus 10-like 1. homolog (mouse) | 0.518 | 4.63E-03 |  | enzyme |
| MYH11 | myosin. heavy chain 11. smooth muscle | -0.524 | 8.62E-02 |  | other |
| MYH7 | myosin. heavy chain 7. cardiac muscle. beta | 0.479 | 7.96E-02 | D | enzyme |
| MYH7 | myosin. heavy chain 7. cardiac muscle. beta | 0.441 | 7.32E-02 | D | enzyme |
| MYL3 | myosin. light chain 3. alkali; ventricular. skeletal. slow | -0.396 | 4.13E-02 |  | other |
| NEFL | neurofilament. light polypeptide | 0.483 | 5.76E-02 | D | other |
| NEFL | neurofilament. light polypeptide | 0.497 | 3.51E-02 | D | other |
| NEFL | neurofilament. light polypeptide | 0.562 | 6.86E-02 | D | other |
| NEIL1 | nei endonuclease VIII-like 1 (E. coli) | -0.373 | 5.13E-02 |  | enzyme |
| NEURL2 | neuralized E3 ubiquitin protein ligase 2 | -0.459 | 3.82E-02 | D | other |
| NEURL2 | neuralized E3 ubiquitin protein ligase 2 | -0.492 | 3.83E-02 | D | other |
| NID1 | nidogen 1 | -0.453 | 8.81E-02 | D | other |
| NID1 | nidogen 1 | -0.674 | 5.18E-02 | D | other |
| NIPSNAP3A | nipsnap homolog 3A (C. elegans) | -0.391 | 6.85E-02 |  | other |
| NLGN4Y | neuroligin 4. Y-linked | 0.380 | 9.36E-02 |  | enzyme |
| NPHP3 | nephronophthisis 3 (adolescent) | -0.420 | 4.03E-02 |  | other |
| NUDT16 | nudix (nucleoside diphosphate linked moiety X)-type motif 16 | -0.420 | 6.90E-02 |  | enzyme |
| OLFM4 | olfactomedin 4 | -0.842 | 4.73E-02 | D | other |
| OLFM4 | olfactomedin 4 | -0.811 | 2.84E-03 | D | other |
| OXCT1 | 3-oxoacid CoA transferase 1 | 0.526 | 6.58E-02 |  | enzyme |
| PARD3 | par-3 family cell polarity regulator | 0.456 | 6.19E-02 |  | other |
| PARN | poly(A)-specific ribonuclease | 0.439 | 2.73E-02 |  | enzyme |
| PBLD | phenazine biosynthesis-like protein domain containing | -0.592 | 9.02E-02 | D | enzyme |
| PBLD | phenazine biosynthesis-like protein domain containing | -1.348 | 6.88E-02 | D | enzyme |
| PBLD | phenazine biosynthesis-like protein domain containing | -0.630 | 1.21E-02 | D | enzyme |
| PCCA | propionyl CoA carboxylase. alpha polypeptide | -0.377 | 2.84E-02 |  | enzyme |
| PDF | peptide deformylase (mitochondrial) | 0.704 | 4.78E-02 |  | enzyme |
| PDLIM5 | PDZ and LIM domain 5 | 0.555 | 2.57E-02 |  | other |
| PGM1 | phosphoglucomutase 1 | -0.511 | 5.01E-02 |  | enzyme |
| PKHD1L1 | polycystic kidney and hepatic disease 1 (autosomal recessive)-like 1 | 0.483 | 7.58E-02 | D | other |
| PKHD1L1 | polycystic kidney and hepatic disease 1 (autosomal recessive)-like 1 | 0.437 | 3.40E-02 | D | other |
| PLA1A | phospholipase A1 member A | -0.393 | 5.29E-02 |  | enzyme |
| PLD1 | phospholipase D1. phosphatidylcholine-specific | -0.380 | 1.92E-02 |  | enzyme |
| PLEKHS1 | pleckstrin homology domain containing. family S member 1 | 0.605 | 1.79E-02 | D | other |
| PLEKHS1 | pleckstrin homology domain containing. family S member 1 | 0.480 | 4.20E-03 | D | other |
| Plscr2 | phospholipid scramblase 2 | -0.541 | 4.32E-02 |  | other |
| PLTP | phospholipid transfer protein | 1.264 | 8.09E-02 |  | enzyme |
| POPDC3 | popeye domain containing 3 | -0.460 | 6.40E-03 |  | other |
| PPAT | phosphoribosyl pyrophosphate amidotransferase | 0.495 | 5.02E-04 |  | enzyme |
| PPDPF | pancreatic progenitor cell differentiation and proliferation factor | 0.451 | 6.11E-02 |  | other |
| PRAF2 | PRA1 domain family. member 2 | -0.461 | 5.63E-02 |  | other |
| PRRC2B | proline-rich coiled-coil 2B | 0.455 | 1.60E-03 |  | other |
| PRTFDC1 | phosphoribosyl transferase domain containing 1 | -0.464 | 6.13E-02 |  | enzyme |
| PTGR2 | prostaglandin reductase 2 | -0.460 | 1.87E-02 |  | enzyme |
| PTS | 6-pyruvoyltetrahydropterin synthase | -0.818 | 6.19E-02 |  | enzyme |
| PURG | purine-rich element binding protein G | 1.842 | 6.83E-02 |  | other |
| PYGB | phosphorylase. glycogen; brain | -0.475 | 5.29E-02 |  | enzyme |
| RCL1 | RNA terminal phosphate cyclase-like 1 | 0.378 | 8.17E-02 |  | enzyme |
| RCVRN | recoverin | -0.432 | 7.17E-02 |  | other |
| RGN | regucalcin | -0.492 | 6.19E-02 |  | enzyme |
| RGS21 | regulator of G-protein signaling 21 | -0.516 | 8.80E-02 |  | other |
| RHOG | ras homolog family member G | -0.401 | 9.83E-02 |  | enzyme |
| RND2 | Rho family GTPase 2 | 0.437 | 2.96E-02 | D | enzyme |
| RND2 | Rho family GTPase 2 | 0.690 | 1.08E-02 | D | enzyme |
| RPE65 | retinal pigment epithelium-specific protein 65kDa | -1.279 | 1.31E-02 |  | enzyme |
| SC5D | sterol-C5-desaturase | 0.680 | 2.80E-02 |  | enzyme |
| SEPP1 | selenoprotein P. plasma. 1 | -0.420 | 8.57E-02 |  | other |
| SERPINB6 | serpin peptidase inhibitor. clade B (ovalbumin). member 6 | -0.532 | 5.78E-02 |  | other |
| SERPINH1 | serpin peptidase inhibitor. clade H (heat shock protein 47). member 1. (collagen binding protein 1) | -0.467 | 6.14E-02 |  | other |
| SESN1 | sestrin 1 | -0.811 | 3.88E-02 | D | other |
| SESN1 | sestrin 1 | -0.697 | 5.81E-02 | D | other |
| SESN1 | sestrin 1 | -0.780 | 5.69E-02 | D | other |
| SH2D4A | SH2 domain containing 4A | 0.531 | 7.18E-02 |  | other |
| SLC16A12 | solute carrier family 16. member 12 | 0.999 | 3.26E-02 |  | other |
| SLC25A38 | solute carrier family 25. member 38 | -1.258 | 8.13E-02 |  | other |
| SLC25A47 | solute carrier family 25. member 47 | -0.721 | 1.18E-02 |  | other |
| SLC9A3R1 | solute carrier family 9. subfamily A (NHE3. cation proton antiporter 3). member 3 regulator 1 | -0.513 | 5.78E-02 | D | other |
| SLC9A3R1 | solute carrier family 9. subfamily A (NHE3. cation proton antiporter 3). member 3 regulator 1 | -0.510 | 9.15E-02 | D | other |
| SMPDL3B | sphingomyelin phosphodiesterase. acid-like 3B | -0.782 | 5.74E-02 |  | enzyme |
| SNRNP25 | small nuclear ribonucleoprotein 25kDa (U11/U12) | -0.392 | 7.20E-02 |  | other |
| SOCS1 | suppressor of cytokine signaling 1 | -1.494 | 6.57E-02 | D | other |
| SOCS1 | suppressor of cytokine signaling 1 | -1.321 | 6.60E-02 | D | other |
| STC2 | stanniocalcin 2 | -1.754 | 1.51E-02 |  | other |
| STEAP4 | STEAP family member 4 | 0.529 | 5.57E-02 |  | enzyme |
| STRA6 | stimulated by retinoic acid 6 | -0.477 | 3.85E-02 | D | other |
| STRA6 | stimulated by retinoic acid 6 | -0.566 | 2.60E-02 | D | other |
| SUCLG2 | succinate-CoA ligase. GDP-forming. beta subunit | -0.419 | 1.63E-02 |  | enzyme |
| SULT1C2 | sulfotransferase family. cytosolic. 1C. member 2 | -0.709 | 5.34E-02 |  | enzyme |
| SULT2B1 | sulfotransferase family. cytosolic. 2B. member 1 | 1.185 | 1.48E-02 | D | enzyme |
| SULT2B1 | sulfotransferase family. cytosolic. 2B. member 1 | 1.216 | 6.97E-03 | D | enzyme |
| SULT2B1 | sulfotransferase family. cytosolic. 2B. member 1 | 0.418 | 2.75E-02 | D | enzyme |
| SULT2B1 | sulfotransferase family. cytosolic. 2B. member 1 | 0.604 | 1.65E-02 | D | enzyme |
| TAT | tyrosine aminotransferase | 1.603 | 1.43E-02 | D | enzyme |
| TAT | tyrosine aminotransferase | 1.403 | 1.16E-02 | D | enzyme |
| TAT | tyrosine aminotransferase | 1.291 | 9.73E-03 | D | enzyme |
| TAT | tyrosine aminotransferase | 1.309 | 3.22E-03 | D | enzyme |
| TAT | tyrosine aminotransferase | 1.522 | 6.46E-03 | D | enzyme |
| TECTB | tectorin beta | 0.802 | 7.69E-02 | D | other |
| TECTB | tectorin beta | 0.897 | 8.15E-02 | D | other |
| TES | testis derived transcript (3 LIM domains) | 0.376 | 7.17E-02 |  | other |
| THBS2 | thrombospondin 2 | 0.747 | 4.31E-02 |  | other |
| TM4SF5 | transmembrane 4 L six family member 5 | -0.563 | 3.76E-02 |  | other |
| TMEM150B | transmembrane protein 150B | -0.761 | 6.28E-02 |  | other |
| TMEM205 | transmembrane protein 205 | -0.422 | 9.97E-03 |  | other |
| TMEM263 | transmembrane protein 263 | 0.872 | 5.33E-02 |  | other |
| TMOD4 | tropomodulin 4 (muscle) | -0.456 | 1.97E-02 |  | other |
| TMX4 | thioredoxin-related transmembrane protein 4 | 0.383 | 8.02E-02 |  | enzyme |
| TNNI2 | troponin I type 2 (skeletal. fast) | -0.403 | 9.93E-02 |  | enzyme |
| TP53INP1 | tumor protein p53 inducible nuclear protein 1 | 0.429 | 5.74E-02 |  | other |
| TREH | trehalase (brush-border membrane glycoprotein) | -0.550 | 7.09E-02 | D | enzyme |
| TREH | trehalase (brush-border membrane glycoprotein) | -0.632 | 7.69E-02 | D | enzyme |
| TRIM3 | tripartite motif containing 3 | -0.425 | 9.17E-02 |  | other |
| TSPAN1 | tetraspanin 1 | -0.929 | 3.23E-02 | D | other |
| TSPAN1 | tetraspanin 1 | -0.943 | 4.74E-02 | D | other |
| TTC36 | tetratricopeptide repeat domain 36 | -0.420 | 6.46E-02 |  | other |
| TTC38 | tetratricopeptide repeat domain 38 | -0.440 | 2.03E-02 |  | other |
| TTC7A | tetratricopeptide repeat domain 7A | -0.426 | 1.23E-02 |  | other |
| TUBA8 | tubulin. alpha 8 | 0.399 | 9.95E-02 |  | other |
| TUBB4B | tubulin. beta 4B class IVb | 0.405 | 1.80E-02 | D | other |
| TUBB4B | tubulin. beta 4B class IVb | 0.434 | 4.56E-03 | D | other |
| TUBB4B | tubulin. beta 4B class IVb | 0.379 | 3.67E-02 | D | other |
| TXNIP | thioredoxin interacting protein | -0.553 | 4.73E-02 |  | other |
| UGDH | UDP-glucose 6-dehydrogenase | -0.523 | 3.32E-02 |  | enzyme |
| UGT1A1 | UDP glucuronosyltransferase 1 family. polypeptide A1 | -0.922 | 1.56E-02 | D | enzyme |
| UGT1A1 | UDP glucuronosyltransferase 1 family. polypeptide A1 | -0.864 | 7.69E-03 | D | enzyme |
| UGT2A3 | UDP glucuronosyltransferase 2 family. polypeptide A3 | -0.398 | 4.79E-02 |  | enzyme |
| UPB1 | ureidopropionase. beta | -0.399 | 2.83E-02 |  | enzyme |
| URAD | ureidoimidazoline (2-oxo-4-hydroxy-4-carboxy-5-) decarboxylase | -0.677 | 7.23E-02 |  | enzyme |
| USH1C | Usher syndrome 1C (autosomal recessive. severe) | -0.820 | 2.63E-02 |  | other |
| VAPB | VAMP (vesicle-associated membrane protein)-associated protein B and C | 0.388 | 4.41E-02 |  | other |
| VASN | vasorin | 0.469 | 1.28E-02 |  | other |
| VIL1 | villin 1 | -0.589 | 3.05E-02 | D | other |
| VIL1 | villin 1 | -0.491 | 3.11E-02 | D | other |
| VIL1 | villin 1 | -0.556 | 5.08E-02 | D | other |
| VTN | vitronectin | 0.649 | 1.11E-02 |  | other |
| WNT3 | wingless-type MMTV integration site family. member 3 | 0.408 | 2.90E-02 |  | other |
| WSB1 | WD repeat and SOCS box containing 1 | -0.492 | 1.34E-02 |  | other |
| YWHAE | tyrosine 3-monooxygenase/tryptophan 5-monooxygenase activation protein. epsilon | 0.454 | 5.15E-02 |  | other |
| ZNF729 | zinc finger protein 729 | -0.465 | 5.26E-02 |  | other |
