# Supplementary material for: Zebrafish Bone and General Physiology Are Differently Affected by Hormones or Changes in Gravity
Source: PLoS One. 2015 Jun 10;10(6):e0126928. doi: 10.1371/journal.pone.0126928 (PMC4465622; doi:10.1371/journal.pone.0126928)
Supplement: S5 Table — Ingenuity Pathway Analysis of the list of genes affected at 6dpf after VitD3 treatment for 24 hours. Columns indicate respectively the function, the range of p-values (significance) associated to various sub-functions, and the number of genes concerned (N). (DOCX) [file pone.0126928.s012.docx]

| **Category** | **p-value** | **Number of Genes** |
| --- | --- | --- |
| Lipid Metabolism | 3.91E-12-1.79E-02 | 107 |
| Molecular Transport | 3.91E-12-1.79E-02 | 136 |
| Small Molecule Biochemistry | 3.91E-12-1.79E-02 | 155 |
| Amino Acid Metabolism | 1.53E-09-1.66E-02 | 46 |
| Carbohydrate Metabolism | 2.86E-09-1.71E-02 | 88 |
| Vitamin and Mineral Metabolism | 2.54E-07-1.3E-02 | 40 |
| Energy Production | 3.41E-07-1.66E-02 | 26 |
| Protein Synthesis | 5.67E-06-1.12E-02 | 81 |
| Cellular Function and Maintenance | 1.98E-05-1.65E-02 | 76 |
| Free Radical Scavenging | 2.08E-05-1.62E-02 | 33 |
| Endocrine System Development and Function | 6.95E-05-1.66E-02 | 35 |
| Drug Metabolism | 1.75E-04-5.66E-03 | 12 |
| Cellular Development | 2.21E-04-1.69E-02 | 59 |
| Cellular Growth and Proliferation | 2.21E-04-1.74E-02 | 43 |
| Hematological System Development and Function | 2.21E-04-1.74E-02 | 48 |
| Cell-To-Cell Signaling and Interaction | 3.6E-04-1.74E-02 | 30 |
| Post-Translational Modification | 3.91E-04-1.66E-02 | 32 |
| Protein Degradation | 3.91E-04-4.87E-03 | 27 |
| Embryonic Development | 3.91E-04-1.66E-02 | 48 |
| Organ Development | 3.91E-04-1.66E-02 | 50 |
| Organismal Development | 3.91E-04-1.66E-02 | 107 |
| Skeletal and Muscular System Development and Function | 3.91E-04-1.78E-02 | 57 |
| Tissue Development | 3.91E-04-1.69E-02 | 63 |
| Cell Cycle | 8.14E-04-1.66E-02 | 17 |
| Organ Morphology | 9.71E-04-1.78E-02 | 61 |
| Tissue Morphology | 9.86E-04-1.66E-02 | 90 |
| Cell Death and Survival | 1.18E-03-1.6E-02 | 46 |
| Cardiovascular System Development and Function | 1.22E-03-1.69E-02 | 72 |
| Humoral Immune Response | 1.22E-03-3.57E-03 | 3 |
| Hair and Skin Development and Function | 1.47E-03-1.66E-02 | 12 |
| Cell Morphology | 2.55E-03-1.66E-02 | 33 |
| Cellular Movement | 2.66E-03-1.45E-02 | 41 |
| Cellular Compromise | 2.77E-03-1.49E-02 | 13 |
| Reproductive System Development and Function | 3.03E-03-1.15E-02 | 41 |
| Behavior | 3.17E-03-3.17E-03 | 15 |
| Digestive System Development and Function | 3.17E-03-1.66E-02 | 52 |
| Hepatic System Development and Function | 3.19E-03-5.44E-03 | 17 |
| Renal and Urological System Development and Function | 3.45E-03-1.54E-02 | 56 |
| Organismal Functions | 3.51E-03-3.51E-03 | 9 |
| Protein Trafficking | 3.57E-03-3.57E-03 | 2 |
| Connective Tissue Development and Function | 3.59E-03-1.69E-02 | 33 |
| Lymphoid Tissue Structure and Development | 4.48E-03-7.35E-03 | 10 |
| Gene Expression | 4.67E-03-1.1E-02 | 10 |
| DNA Replication. Recombination. and Repair | 6.15E-03-6.15E-03 | 7 |
| Nucleic Acid Metabolism | 6.15E-03-6.15E-03 | 7 |
| Cell-mediated Immune Response | 6.97E-03-7.35E-03 | 5 |
| Cellular Assembly and Organization | 6.97E-03-1.66E-02 | 14 |
| Hematopoiesis | 6.97E-03-7.35E-03 | 5 |
| Cell Signaling | 7.35E-03-7.35E-03 | 3 |
| Nervous System Development and Function | 7.65E-03-7.65E-03 | 4 |
| Visual System Development and Function | 7.65E-03-1.13E-02 | 12 |

Table S5
