# Supplementary material for: Zebrafish Bone and General Physiology Are Differently Affected by Hormones or Changes in Gravity
Source: PLoS One. 2015 Jun 10;10(6):e0126928. doi: 10.1371/journal.pone.0126928 (PMC4465622; doi:10.1371/journal.pone.0126928)
Supplement: S6 Table — The table indicates the human homolog of the gene, its "Entrez" gene name, the log ratio of PTH-treated larvae compared to control, the presence of duplicate probes on the microarray (D) and the type of protein it encodes. Genes are arranged according to their type and in alphabetical order. (DOCX) [file pone.0126928.s013.docx]

| Symbol | **Entrez Gene Name** | **Log Ratio PTH** | **p-value** | **N** | **Type(s)** |
| --- | --- | --- | --- | --- | --- |
| BET1L | Bet1 golgi vesicular membrane trafficking protein-like | 1.210 | 7.53E-02 |  | transporter |
| CACNB1 | calcium channel. voltage-dependent. beta 1 subunit | -1.060 | 7.90E-02 |  | ion channel |
| CLCN1 | chloride channel. voltage-sensitive 1 | 0.797 | 5.64E-02 |  | ion channel |
| COMMD1 | copper metabolism (Murr1) domain containing 1 | 0.545 | 8.50E-02 |  | transporter |
| GJA9 | gap junction protein. alpha 9. 59kDa | 1.750 | 9.39E-02 |  | transporter |
| KCNK18 | potassium channel. subfamily K. member 18 | 1.040 | 6.67E-02 |  | ion channel |
| MB | myoglobin | -0.656 | 9.39E-02 |  | transporter |
| MTX1 | metaxin 1 | -0.415 | 5.90E-02 |  | transporter |
| NXF1 | nuclear RNA export factor 1 | -0.902 | 7.90E-02 |  | transporter |
| P2RX7 | purinergic receptor P2X. ligand-gated ion channel. 7 | -1.420 | 8.96E-02 |  | ion channel |
| PANX1 | pannexin 1 | -1.060 | 9.58E-02 |  | transporter |
| RPH3A | rabphilin 3A homolog (mouse) | 0.836 | 4.94E-02 |  | transporter |
| SLC12A3 | solute carrier family 12 (sodium/chloride transporter). member 3 | -0.763 | 5.64E-02 |  | transporter |
| SLC18A3 | solute carrier family 18 (vesicular acetylcholine transporter). member 3 | -1.930 | 5.58E-02 |  | transporter |
| SLC43A1 | solute carrier family 43 (amino acid system L transporter). member 1 | 0.405 | 9.81E-02 |  | transporter |
| SLC6A18 | solute carrier family 6 (neutral amino acid transporter). member 18 | -2.300 | 6.63E-02 |  | transporter |
| SLC7A10 | solute carrier family 7 (neutral amino acid transporter light chain. asc system). member 10 | -1.100 | 5.64E-02 |  | transporter |
| SLC9A6 | solute carrier family 9. subfamily A (NHE6. cation proton antiporter 6). member 6 | -0.985 | 7.90E-02 |  | transporter |
| SMC4 | structural maintenance of chromosomes 4 | 0.376 | 7.83E-02 |  | transporter |
| AATF | apoptosis antagonizing transcription factor | -1.050 | 6.63E-02 |  | transcription regulator |
| CALR | calreticulin | 1.060 | 6.36E-02 |  | transcription regulator |
| DMBX1 | diencephalon/mesencephalon homeobox 1 | 0.721 | 9.09E-02 |  | transcription regulator |
| EEF2 | eukaryotic translation elongation factor 2 | 0.722 | 9.81E-02 |  | translation regulator |
| EGR1 | early growth response 1 | -0.725 | 9.91E-02 |  | transcription regulator |
| EPC1 | enhancer of polycomb homolog 1 (Drosophila) | -0.698 | 7.95E-02 |  | transcription regulator |
| ESR2 | estrogen receptor 2 (ER beta) | -1.250 | 9.81E-02 |  | ligand-dependent nuclear receptor |
| FOXB2 | forkhead box B2 | -1.080 | 5.89E-02 |  | transcription regulator |
| GATA4 | GATA binding protein 4 | 0.879 | 8.83E-02 |  | transcription regulator |
| HOXA5 | homeobox A5 | -0.515 | 7.90E-02 |  | transcription regulator |
| INSM2 | insulinoma-associated 2 | 0.732 | 7.64E-02 |  | transcription regulator |
| IRX6 | iroquois homeobox 6 | 0.631 | 7.90E-02 |  | transcription regulator |
| JARID2 | jumonji. AT rich interactive domain 2 | -1.400 | 9.95E-02 |  | transcription regulator |
| LDB2 | LIM domain binding 2 | 0.597 | 4.94E-02 | D | transcription regulator |
| LDB2 | LIM domain binding 2 | 1.330 | 5.64E-02 | D | transcription regulator |
| LRCH4 | leucine-rich repeats and calponin homology (CH) domain containing 4 | 1.030 | 5.47E-02 |  | transcription regulator |
| MXI1 | MAX interactor 1. dimerization protein | 0.530 | 7.90E-02 |  | transcription regulator |
| NKX3-2 | NK3 homeobox 2 | 0.697 | 7.14E-02 |  | transcription regulator |
| PDLIM1 | PDZ and LIM domain 1 | -0.943 | 9.27E-02 |  | transcription regulator |
| PTRF | polymerase I and transcript release factor | 0.480 | 8.88E-02 |  | transcription regulator |
| RPS9 | ribosomal protein S9 | 0.613 | 7.19E-02 |  | translation regulator |
| RXRA | retinoid X receptor. alpha | 0.993 | 7.64E-02 |  | ligand-dependent nuclear receptor |
| TAF1 | TAF1 RNA polymerase II. TATA box binding protein (TBP)-associated factor. 250kDa | 0.416 | 7.96E-02 |  | transcription regulator |
| TOX2 | TOX high mobility group box family member 2 | 0.604 | 7.83E-02 |  | transcription regulator |
| ACKR3 | atypical chemokine receptor 3 | -2.210 | 6.67E-02 |  | G-protein coupled receptor |
| AVPR1A | arginine vasopressin receptor 1A | 2.150 | 4.94E-02 |  | G-protein coupled receptor |
| CALCRL | calcitonin receptor-like | 0.735 | 7.74E-02 |  | G-protein coupled receptor |
| CHRM2 | cholinergic receptor. muscarinic 2 | 0.500 | 9.48E-02 |  | G-protein coupled receptor |
| CHRNA6 | cholinergic receptor. nicotinic. alpha 6 (neuronal) | -2.090 | 6.63E-02 |  | transmembrane receptor |
| GFRA1 | GDNF family receptor alpha 1 | 1.200 | 9.81E-02 |  | transmembrane receptor |
| GPR132 | G protein-coupled receptor 132 | 0.973 | 8.14E-02 |  | G-protein coupled receptor |
| HLA-B | major histocompatibility complex. class I. B | 0.469 | 6.75E-02 |  | transmembrane receptor |
| ILDR1 | immunoglobulin-like domain containing receptor 1 | 0.788 | 6.63E-02 |  | transmembrane receptor |
| ITGA4 | integrin. alpha 4 (antigen CD49D. alpha 4 subunit of VLA-4 receptor) | 0.758 | 8.50E-02 |  | transmembrane receptor |
| LHCGR | luteinizing hormone/choriogonadotropin receptor | -1.130 | 9.91E-02 |  | G-protein coupled receptor |
| LY75 | lymphocyte antigen 75 | -1.390 | 5.90E-02 |  | transmembrane receptor |
| OR8G5 | olfactory receptor. family 8. subfamily G. member 5 | -2.060 | 9.02E-02 |  | G-protein coupled receptor |
| PTHR1 | parathyroid hormone receptor | 0.908 | 6.85E-02 |  | G-protein coupled receptor |
| TNFRSF1A | tumor necrosis factor receptor superfamily. member 1A | 1.010 | 7.74E-02 |  | transmembrane receptor |
| TNFRSF21 | tumor necrosis factor receptor superfamily. member 21 | 0.782 | 4.94E-02 |  | transmembrane receptor |
| FAM3C | family with sequence similarity 3. member C | -0.477 | 9.09E-02 |  | cytokine |
| FGF4 | fibroblast growth factor 4 | -1.150 | 7.87E-02 |  | growth factor |
| GDF9 | growth differentiation factor 9 | -1.900 | 7.90E-02 |  | growth factor |
| IGF1 | insulin-like growth factor 1 (somatomedin C) | 1.460 | 8.88E-02 |  | growth factor |
| INHBB | inhibin. beta B | -0.830 | 9.02E-02 |  | growth factor |
| AK3 | adenylate kinase 3 | -0.557 | 9.09E-02 |  | kinase |
| COASY | CoA synthase | -1.430 | 9.81E-02 |  | kinase |
| DAPK3 | death-associated protein kinase 3 | -1.300 | 4.94E-02 |  | kinase |
| DCLK2 | doublecortin-like kinase 2 | 1.240 | 7.39E-02 |  | kinase |
| EPHB2 | EPH receptor B2 | 1.540 | 9.81E-02 |  | kinase |
| GRK4 | G protein-coupled receptor kinase 4 | 1.840 | 5.60E-02 |  | kinase |
| ILKAP | integrin-linked kinase-associated serine/threonine phosphatase | -0.707 | 5.58E-02 |  | phosphatase |
| NAGK | N-acetylglucosamine kinase | -0.816 | 8.83E-02 |  | kinase |
| NME2 | NME/NM23 nucleoside diphosphate kinase 2 | 0.743 | 8.21E-02 |  | kinase |
| NRBP2 | nuclear receptor binding protein 2 | 1.330 | 5.64E-02 | D | kinase |
| RPS6KA2 | ribosomal protein S6 kinase. 90kDa. polypeptide 2 | -0.735 | 6.63E-02 |  | kinase |
| SGK1 | serum/glucocorticoid regulated kinase 1 | -0.505 | 7.96E-02 |  | kinase |
| SYNJ1 | synaptojanin 1 | -2.670 | 8.55E-02 |  | phosphatase |
| Afg3l1 | AFG3(ATPase family gene 3)-like 1 (yeast) | -4.740 | 3.48E-02 |  | peptidase |
| CPA2 | carboxypeptidase A2 (pancreatic) | -0.647 | 9.81E-02 |  | peptidase |
| IDE | insulin-degrading enzyme | -0.623 | 9.09E-02 |  | peptidase |
| RHBDL2 | rhomboid. veinlet-like 2 (Drosophila) | -0.679 | 9.26E-02 |  | peptidase |
| SPPL2A | signal peptide peptidase like 2A | 0.542 | 6.63E-02 |  | peptidase |
| USP24 | ubiquitin specific peptidase 24 | 1.240 | 6.63E-02 |  | peptidase |
| ABI1 | abl-interactor 1 | 1.040 | 7.14E-02 |  | other |
| ADAP2 | ArfGAP with dual PH domains 2 | -0.726 | 4.94E-02 |  | other |
| ALKBH5 | alkB. alkylation repair homolog 5 (E. coli) | -0.900 | 6.63E-02 |  | enzyme |
| ANLN | anillin. actin binding protein | -1.370 | 8.50E-02 |  | other |
| ARGLU1 | arginine and glutamate rich 1 | 0.595 | 9.81E-02 |  | other |
| ARHGEF11 | Rho guanine nucleotide exchange factor (GEF) 11 | 0.750 | 5.97E-02 |  | other |
| ARHGEF19 | Rho guanine nucleotide exchange factor (GEF) 19 | 1.420 | 7.80E-02 |  | other |
| ARL8B | ADP-ribosylation factor-like 8B | 1.270 | 9.39E-02 |  | enzyme |
| ARPC5 | actin related protein 2/3 complex. subunit 5. 16kDa | -1.050 | 5.64E-02 |  | other |
| ARRB2 | arrestin. beta 2 | -0.684 | 6.63E-02 |  | other |
| ASPN | asporin | 1.670 | 5.50E-02 |  | other |
| ASRGL1 | asparaginase like 1 | -0.765 | 4.94E-02 |  | enzyme |
| AXIN1 | axin 1 | 0.404 | 9.81E-02 |  | other |
| C15orf41 | chromosome 15 open reading frame 41 | -0.815 | 7.83E-02 |  | other |
| C2orf40 | chromosome 2 open reading frame 40 | 1.060 | 9.69E-02 |  | other |
| C2orf47 | chromosome 2 open reading frame 47 | -0.951 | 6.61E-02 |  | other |
| C3orf58 | chromosome 3 open reading frame 58 | -0.676 | 5.58E-02 |  | other |
| C4orf29 | chromosome 4 open reading frame 29 | -0.904 | 3.48E-02 |  | other |
| C7 | complement component 7 | 1.310 | 4.94E-02 |  | other |
| CA8 | carbonic anhydrase VIII | -0.650 | 8.50E-02 |  | enzyme |
| CAB39 | calcium binding protein 39 | 1.540 | 9.51E-02 |  | enzyme |
| CABLES2 | Cdk5 and Abl enzyme substrate 2 | 0.543 | 8.50E-02 |  | other |
| CABLES2 | Cdk5 and Abl enzyme substrate 2 | -2.030 | 9.69E-02 |  | other |
| CAD | carbamoyl-phosphate synthetase 2. aspartate transcarbamylase. and dihydroorotase | -1.570 | 6.78E-02 |  | enzyme |
| CARS | cysteinyl-tRNA synthetase | -0.417 | 9.09E-02 |  | enzyme |
| CBY1 | chibby homolog 1 (Drosophila) | 2.390 | 4.94E-02 |  | other |
| CCDC62 | coiled-coil domain containing 62 | 0.495 | 9.16E-02 |  | other |
| CD151 | CD151 molecule (Raph blood group) | -0.709 | 5.64E-02 |  | other |
| CDC34 | cell division cycle 34 | -0.878 | 6.85E-02 |  | enzyme |
| CDIPT | CDP-diacylglycerol--inositol 3-phosphatidyltransferase | -0.583 | 8.83E-02 |  | enzyme |
| CES1 | carboxylesterase 1 | 0.555 | 4.94E-02 |  | enzyme |
| CHD4 | chromodomain helicase DNA binding protein 4 | 0.425 | 9.39E-02 |  | enzyme |
| CNPY3 | canopy FGF signaling regulator 3 | -0.699 | 5.64E-02 |  | other |
| CPLX2 | complexin 2 | 0.392 | 9.69E-02 |  | other |
| CS | citrate synthase | -0.682 | 8.83E-02 |  | enzyme |
| CWC22 | CWC22 spliceosome-associated protein homolog (S. cerevisiae) | 0.924 | 8.45E-02 |  | other |
| CYP21A2 | cytochrome P450. family 21. subfamily A. polypeptide 2 | -1.260 | 5.89E-02 |  | enzyme |
| CYP2J2 | cytochrome P450. family 2. subfamily J. polypeptide 2 | -0.742 | 9.81E-02 |  | enzyme |
| DCPS | decapping enzyme. scavenger | -0.982 | 9.39E-02 |  | enzyme |
| DCTN1 | dynactin 1 | 0.694 | 6.63E-02 |  | other |
| DENND5A | DENN/MADD domain containing 5A | -1.240 | 7.96E-02 |  | other |
| DNAJB11 | DnaJ (Hsp40) homolog. subfamily B. member 11 | -0.892 | 4.94E-02 |  | other |
| DNASE1L3 | deoxyribonuclease I-like 3 | -0.544 | 8.83E-02 |  | enzyme |
| EFCAB4B | EF-hand calcium binding domain 4B | 1.590 | 6.63E-02 |  | other |
| EFEMP1 | EGF containing fibulin-like extracellular matrix protein 1 | -2.240 | 7.64E-02 |  | enzyme |
| EFNA1 | ephrin-A1 | 0.450 | 8.44E-02 |  | other |
| FAIM | Fas apoptotic inhibitory molecule | 1.940 | 7.74E-02 |  | other |
| FAM177A1 | family with sequence similarity 177. member A1 | -0.469 | 7.80E-02 |  | other |
| FBLN1 | fibulin 1 | 0.742 | 8.50E-02 |  | other |
| FOXRED1 | FAD-dependent oxidoreductase domain containing 1 | -0.846 | 7.80E-02 |  | other |
| GALNT2 | polypeptide N-acetylgalactosaminyltransferase 2 | -0.808 | 7.95E-02 |  | enzyme |
| GLB1 | galactosidase. beta 1 | 1.370 | 5.64E-02 |  | enzyme |
| GLB1L | galactosidase. beta 1-like | 0.654 | 9.09E-02 |  | other |
| Gm16500 | predicted gene 16500 | -0.625 | 5.58E-02 |  | other |
| GNAL | guanine nucleotide binding protein (G protein). alpha activating activity polypeptide. olfactory type | -0.768 | 9.37E-02 |  | enzyme |
| GPAM | glycerol-3-phosphate acyltransferase. mitochondrial | 0.894 | 9.39E-02 |  | enzyme |
| GPC3 | glypican 3 | 0.521 | 9.81E-02 |  | other |
| GRAMD1B | GRAM domain containing 1B | 0.924 | 4.94E-02 |  | other |
| GRAMD1C | GRAM domain containing 1C | -0.553 | 4.94E-02 |  | other |
| GSR | glutathione reductase | -0.847 | 5.64E-02 |  | enzyme |
| GUCA1A | guanylate cyclase activator 1A (retina) | 0.659 | 8.18E-02 |  | other |
| GUSB | glucuronidase. beta | 0.544 | 7.97E-02 |  | enzyme |
| HARS | histidyl-tRNA synthetase | -0.929 | 5.64E-02 |  | enzyme |
| HAUS6 | HAUS augmin-like complex. subunit 6 | -1.550 | 5.58E-02 |  | other |
| HDC | histidine decarboxylase | 0.796 | 9.16E-02 |  | enzyme |
| HIST2H2AB | histone cluster 2. H2ab | 2.200 | 5.64E-02 |  | other |
| HLA-A | major histocompatibility complex. class I. A | -1.210 | 9.31E-02 |  | other |
| Hmga2 | high mobility group AT-hook 2 | 0.749 | 5.64E-02 |  | enzyme |
| HSD3B7 | hydroxy-delta-5-steroid dehydrogenase. 3 beta- and steroid delta-isomerase 7 | -0.626 | 6.85E-02 |  | enzyme |
| ISM2 | isthmin 2 | -1.630 | 8.83E-02 |  | other |
| ITGA9 | integrin. alpha 9 | 1.620 | 6.61E-02 |  | other |
| KIAA1324L | KIAA1324-like | 0.789 | 6.84E-02 |  | other |
| KIF23 | kinesin family member 23 | 1.440 | 6.99E-02 |  | other |
| KLHDC8A | kelch domain containing 8A | 0.569 | 7.96E-02 |  | other |
| KLHL40 | kelch-like family member 40 | -1.080 | 8.45E-02 |  | other |
| KRT17 | keratin 17 | 0.961 | 7.64E-02 |  | other |
| L3HYPDH | L-3-hydroxyproline dehydratase (trans-) | 0.984 | 7.64E-02 |  | enzyme |
| LCTL | lactase-like | -1.250 | 5.64E-02 |  | enzyme |
| LGI1 | leucine-rich. glioma inactivated 1 | -1.340 | 6.63E-02 |  | other |
| LIPH | lipase. member H | -0.666 | 7.39E-02 |  | enzyme |
| LOC102551489 | protein unc-13 homolog C-like | 0.615 | 5.47E-02 |  | other |
| LOC391722 | myosin regulatory light chain 12B-like | 0.561 | 9.39E-02 |  | other |
| MARVELD1 | MARVEL domain containing 1 | 0.596 | 9.81E-02 |  | other |
| MCPH1 | microcephalin 1 | 0.952 | 5.58E-02 |  | other |
| MFAP3L | microfibrillar-associated protein 3-like | -0.835 | 5.64E-02 |  | other |
| MLEC | malectin | -0.645 | 7.14E-02 |  | other |
| MOCOS | molybdenum cofactor sulfurase | 1.700 | 7.90E-02 |  | enzyme |
| MRPL41 | mitochondrial ribosomal protein L41 | -0.438 | 7.14E-02 |  | other |
| MSI2 | musashi RNA-binding protein 2 | 0.945 | 9.81E-02 |  | other |
| MTSS1 | metastasis suppressor 1 | -0.852 | 9.76E-02 |  | other |
| MYO1G | myosin IG | -1.330 | 9.96E-02 |  | other |
| NDRG2 | NDRG family member 2 | 0.628 | 5.97E-02 |  | other |
| OSBPL2 | oxysterol binding protein-like 2 | -1.130 | 9.16E-02 |  | other |
| PALM2 | paralemmin 2 | 1.120 | 7.64E-02 |  | other |
| PAPL | iron/zinc purple acid phosphatase-like protein | 0.867 | 9.69E-02 |  | enzyme |
| PARP14 | poly (ADP-ribose) polymerase family. member 14 | 0.765 | 5.58E-02 |  | other |
| PDIA4 | protein disulfide isomerase family A. member 4 | -0.652 | 5.67E-02 |  | enzyme |
| PDZD8 | PDZ domain containing 8 | 0.876 | 9.03E-02 |  | other |
| PHLDA2 | pleckstrin homology-like domain. family A. member 2 | -0.853 | 5.64E-02 |  | other |
| PLA2G12A | phospholipase A2. group XIIA | 0.779 | 9.39E-02 |  | enzyme |
| PPIL2 | peptidylprolyl isomerase (cyclophilin)-like 2 | -0.408 | 7.90E-02 |  | enzyme |
| PPP1R14C | protein phosphatase 1. regulatory (inhibitor) subunit 14C | -1.640 | 5.64E-02 |  | other |
| PRMT1 | protein arginine methyltransferase 1 | -0.971 | 5.90E-02 |  | enzyme |
| PSMD5 | proteasome (prosome. macropain) 26S subunit. non-ATPase. 5 | -0.915 | 7.70E-02 |  | other |
| PTGES | prostaglandin E synthase | 0.509 | 9.70E-02 |  | enzyme |
| PTGES | prostaglandin E synthase | 1.510 | 9.81E-02 |  | enzyme |
| PTH1 | parathyroid hormone | -1.320 | 4.94E-02 |  | other |
| PTX3 | pentraxin 3. long | 1.820 | 3.48E-02 |  | other |
| RAD21 | RAD21 homolog (S. pombe) | 1.570 | 6.75E-02 |  | other |
| RALGDS | ral guanine nucleotide dissociation stimulator | -0.985 | 4.94E-02 |  | other |
| RBM18 | RNA binding motif protein 18 | -0.655 | 6.72E-02 |  | other |
| RHOF | ras homolog family member F (in filopodia) | -1.700 | 4.94E-02 |  | enzyme |
| RIT1 | Ras-like without CAAX 1 | 1.050 | 9.16E-02 |  | enzyme |
| RPAP1 | RNA polymerase II associated protein 1 | -1.220 | 9.81E-02 |  | other |
| RPL23 | ribosomal protein L23 | 0.705 | 9.39E-02 |  | other |
| RPL27A | ribosomal protein L27a | 0.977 | 7.90E-02 |  | other |
| RPUSD1 | RNA pseudouridylate synthase domain containing 1 | -0.862 | 5.64E-02 |  | enzyme |
| SAG | S-antigen; retina and pineal gland (arrestin) | 0.517 | 7.64E-02 |  | other |
| SASH1 | SAM and SH3 domain containing 1 | 1.970 | 9.25E-02 |  | other |
| SCARB2 | scavenger receptor class B. member 2 | 1.360 | 7.64E-02 |  | other |
| SERPINE1 | serpin peptidase inhibitor. clade E (nexin. plasminogen activator inhibitor type 1). member 1 | -0.981 | 9.81E-02 |  | other |
| SGCG | sarcoglycan. gamma (35kDa dystrophin-associated glycoprotein) | -1.510 | 3.48E-02 |  | other |
| SHISA2 | shisa family member 2 | 0.907 | 6.63E-02 |  | other |
| SLC25A51 | solute carrier family 25. member 51 | -0.658 | 8.20E-02 |  | other |
| SLC43A3 | solute carrier family 43. member 3 | -1.330 | 3.48E-02 |  | other |
| SMURF2 | SMAD specific E3 ubiquitin protein ligase 2 | -3.370 | 9.30E-02 |  | enzyme |
| SPTLC3 | serine palmitoyltransferase. long chain base subunit 3 | -1.180 | 6.85E-02 |  | enzyme |
| SRSF1 | serine/arginine-rich splicing factor 1 | -0.577 | 7.87E-02 | D | other |
| SRSF1 | serine/arginine-rich splicing factor 1 | -0.529 | 7.90E-02 | D | other |
| SSB | Sjogren syndrome antigen B (autoantigen La) | -0.657 | 6.41E-02 |  | enzyme |
| ST6GAL2 | ST6 beta-galactosamide alpha-2.6-sialyltranferase 2 | 0.645 | 7.63E-02 |  | enzyme |
| ST8SIA2 | ST8 alpha-N-acetyl-neuraminide alpha-2.8-sialyltransferase 2 | 0.641 | 7.95E-02 |  | enzyme |
| TANGO2 | transport and golgi organization 2 homolog (Drosophila) | -1.460 | 5.58E-02 |  | other |
| TBC1D1 | TBC1 (tre-2/USP6. BUB2. cdc16) domain family. member 1 | 1.990 | 5.50E-02 |  | other |
| TFPI | tissue factor pathway inhibitor (lipoprotein-associated coagulation inhibitor) | -2.390 | 5.64E-02 |  | other |
| TGFBRAP1 | transforming growth factor. beta receptor associated protein 1 | 0.705 | 6.75E-02 |  | other |
| THOC2 | THO complex 2 | 0.491 | 7.74E-02 |  | other |
| TMEM181 | transmembrane protein 181 | 2.470 | 4.94E-02 |  | other |
| TMEM30B | transmembrane protein 30B | -1.070 | 9.39E-02 |  | other |
| TMEM87B | transmembrane protein 87B | 0.501 | 9.26E-02 |  | other |
| TMX3 | thioredoxin-related transmembrane protein 3 | 0.600 | 6.63E-02 |  | enzyme |
| TPD52L1 | tumor protein D52-like 1 | -0.544 | 9.81E-02 |  | other |
| TSPEAR | thrombospondin-type laminin G domain and EAR repeats | 0.741 | 7.58E-02 |  | other |
| TTC14 | tetratricopeptide repeat domain 14 | -0.712 | 6.85E-02 |  | other |
| Ttc39a | tetratricopeptide repeat domain 39A | -0.906 | 7.14E-02 |  | other |
| TXNRD3 | thioredoxin reductase 3 | -1.490 | 9.37E-02 |  | enzyme |
| UNC93B1 | unc-93 homolog B1 (C. elegans) | 0.698 | 9.39E-02 |  | other |
| USP32 | ubiquitin specific peptidase 32 | 0.805 | 7.74E-02 |  | enzyme |
| VPS37B | vacuolar protein sorting 37 homolog B (S. cerevisiae) | 0.758 | 5.58E-02 |  | other |
| VSNL1 | visinin-like 1 | 0.441 | 9.25E-02 |  | other |
| WDR5 | WD repeat domain 5 | -1.400 | 9.09E-02 |  | other |
| XAF1 | XIAP associated factor 1 | 0.857 | 6.63E-02 |  | other |
| ZC3H6 | zinc finger CCCH-type containing 6 | 0.961 | 8.28E-02 |  | other |
| ZNF346 | zinc finger protein 346 | 0.867 | 9.39E-02 |  | other |
| ZNF729 | zinc finger protein 729 | -1.320 | 6.85E-02 | D | other |
| ZNF729 | zinc finger protein 729 | -0.981 | 8.50E-02 | D | other |
| ZNF729 | zinc finger protein 729 | 2.170 | 9.16E-02 | D | other |
