# Supplementary material for: Zebrafish Bone and General Physiology Are Differently Affected by Hormones or Changes in Gravity
Source: PLoS One. 2015 Jun 10;10(6):e0126928. doi: 10.1371/journal.pone.0126928 (PMC4465622; doi:10.1371/journal.pone.0126928)
Supplement: S7 Table — Ingenuity Pathway Analysis of the list of genes affected at 6dpf after PTH treatment for 24 hours. Columns indicate respectively the function, the range of p-values (significance) associated to various sub-functions, and the number of genes concerned (N). (DOCX) [file pone.0126928.s014.docx]

| **Category** | **p-value** | | **Number of Genes** |
| --- | --- | --- | --- |
| Cellular Development | | 1.83E-04-4.02E-02 | 13 |
| Connective Tissue Development and Function | | 1.83E-04-4.02E-02 | 13 |
| Embryonic Development | | 1.83E-04-4.02E-02 | 17 |
| Cell-To-Cell Signaling and Interaction | | 5.45E-04-5E-02 | 18 |
| Cellular Assembly and Organization | | 5.45E-04-4.16E-02 | 13 |
| Cellular Function and Maintenance | | 5.45E-04-4.16E-02 | 18 |
| Hair and Skin Development and Function | | 5.45E-04-4.02E-02 | 5 |
| Hematological System Development and Function | | 5.45E-04-5E-02 | 18 |
| Hematopoiesis | | 5.45E-04-4.02E-02 | 5 |
| Organ Morphology | | 5.45E-04-4.86E-02 | 22 |
| Skeletal and Muscular System Development and Function | | 5.45E-04-4.02E-02 | 13 |
| Tissue Development | | 5.45E-04-4.08E-02 | 25 |
| Cellular Movement | | 1.15E-03-5E-02 | 16 |
| Immune Cell Trafficking | | 1.15E-03-5E-02 | 15 |
| Cell Cycle | | 1.78E-03-4.02E-02 | 10 |
| Cell Morphology | | 1.78E-03-4.99E-02 | 19 |
| Organ Development | | 1.78E-03-4.02E-02 | 15 |
| Organismal Development | | 1.78E-03-4.86E-02 | 23 |
| Respiratory System Development and Function | | 1.78E-03-4.02E-02 | 3 |
| Tissue Morphology | | 1.78E-03-4.63E-02 | 14 |
| Cardiovascular System Development and Function | | 2.65E-03-4.86E-02 | 16 |
| Cellular Compromise | | 2.65E-03-4.02E-02 | 9 |
| Cell Death and Survival | | 3.55E-03-4.02E-02 | 31 |
| Inflammatory Response | | 5.07E-03-4.16E-02 | 13 |
| Cellular Growth and Proliferation | | 5.65E-03-1.36E-02 | 7 |
| Nervous System Development and Function | | 5.65E-03-4.02E-02 | 15 |
| Small Molecule Biochemistry | | 6.2E-03-4.02E-02 | 21 |
| Molecular Transport | | 7.68E-03-4.79E-02 | 15 |
| Humoral Immune Response | | 8.35E-03-4.79E-02 | 5 |
| Protein Synthesis | | 8.35E-03-4.79E-02 | 17 |
| Cell-mediated Immune Response | | 8.48E-03-2.7E-02 | 6 |
| Cardiovascular Disease | | 9.3E-03-4.49E-02 | 8 |
| Digestive System Development and Function | | 9.3E-03-2.7E-02 | 3 |
| Lymphoid Tissue Structure and Development | | 9.3E-03-4.49E-02 | 5 |
| Carbohydrate Metabolism | | 1.08E-02-2.7E-02 | 9 |
| Lipid Metabolism | | 1.11E-02-4.02E-02 | 8 |
| Amino Acid Metabolism | | 1.36E-02-4.02E-02 | 2 |
| Antimicrobial Response | | 1.36E-02-1.36E-02 | 1 |
| Cell Signaling | | 1.36E-02-4.02E-02 | 4 |
| Drug Metabolism | | 1.36E-02-2.7E-02 | 6 |
| Endocrine System Development and Function | | 1.36E-02-1.36E-02 | 1 |
| Gene Expression | | 1.36E-02-4.57E-02 | 11 |
| Hepatic System Development and Function | | 1.36E-02-2.7E-02 | 2 |
| Nucleic Acid Metabolism | | 1.36E-02-4.02E-02 | 5 |
| RNA Post-Transcriptional Modification | | 1.36E-02-2.7E-02 | 1 |
| Renal and Urological System Development and Function | | 1.36E-02-2.7E-02 | 4 |
| Reproductive System Development and Function | | 1.36E-02-4.16E-02 | 6 |
| Visual System Development and Function | | 1.36E-02-2.7E-02 | 2 |
| Vitamin and Mineral Metabolism | | 1.36E-02-4.02E-02 | 4 |
| Organismal Functions | | 2.18E-02-2.18E-02 | 2 |
| Behavior | | 2.7E-02-4.02E-02 | 3 |
| Free Radical Scavenging | | 2.7E-02-2.7E-02 | 1 |
| Post-Translational Modification | | 2.7E-02-3.02E-02 | 5 |
| Auditory and Vestibular System Development and Function | | 4.02E-02-4.02E-02 | 1 |
| RNA Trafficking | | 4.02E-02-4.49E-02 | 2 |

TableS7
