# Supplementary material for: Zebrafish Bone and General Physiology Are Differently Affected by Hormones or Changes in Gravity
Source: PLoS One. 2015 Jun 10;10(6):e0126928. doi: 10.1371/journal.pone.0126928 (PMC4465622; doi:10.1371/journal.pone.0126928)
Supplement: S8 Table — The fraction (in %) of larvae presenting the indicated score for each element is given, together with the statistical evaluation of a significant difference compared to control. (A) The bone structures distributed in 2 categories (early and advanced ossification) (B) The bone structures distributed in 3 categories (absent, early and advanced ossification) (DOCX) [file pone.0126928.s015.docx]

Table S8

A

|  |  | |  | |  |  | | | Score of ossificaiton (Y) | | | | X² pearson | | | Logistic regression | | |
| --- | --- | --- | --- | --- | --- | --- | --- | --- | --- | --- | --- | --- | --- | --- | --- | --- | --- | --- |
| Structures | | Treat | | | N | Mean | | early | | | advanced | | p-value | | OR (IC 95%) | | p-value | |
| branchiostegal ray1 down | | Control | | | 24 | 0.96 | | 3 (12.50%) | | | 21 (87.50%) | |  | | 1 | |  | |
|  | | LDC 1g > 3g | | | 24 | 1.00 | | 0 (0%) | | | 24 (100%) | | 0.074 | | / | | 0.995 | |
| branchiostegal ray1 up | | Control | | | 24 | 0.96 | | 3 (12.50%) | | | 21 (87.50%) | |  | | 1 | |  | |
|  | | LDC 1g > 3g | | | 24 | 1.00 | | 0 (0%) | | | 24 (100%) | | 0.074 | | / | | 0.995 | |
| dentary down | | Control | | | 24 | 1.00 | | 0 (0%) | | | 24 (100%) | |  | | 1 | |  | |
|  | | LDC 1g > 3g | | | 24 | 0.96 | | 1 (4.17%) | | | 23 (95.83%) | | 0.312 | | / | | 0.995 | |
| dentary up | | Control | | | 24 | 1.00 | | 0 (0%) | | | 24 (100%) | |  | | 1 | |  | |
|  | | LDC 1g > 3g | | | 24 | 0.96 | | 1 (4.17%) | | | 23 (95.83%) | | 0.312 | | / | | 0.995 | |
| entopterygoid down | | Control | | | 24 | 1.00 | | 0 (0%) | | | 24 (100%) | |  | | 1 | |  | |
|  | | LDC 1g > 3g | | | 24 | 1.00 | | 0 (0%) | | | 24 (100%) | | / | | / | | / | |
| entopterygoid up | | Control | | | 24 | 1.00 | | 0 (0%) | | | 24 (100%) | |  | | 1 | |  | |
|  | | LDC 3 g | | | 24 | 1.00 | | 0 (0%) | | | 24 (100%) | | / | | / | | / | |
| hyomandibular down | | Control | | | 24 | 0.71 | | 7 (29.17%) | | | 17 (70.83%) | |  | | 1 | |  | |
|  | | LDC 1g > 3g | | | 24 | 0.63 | | 9 (37.50%) | | | 15 (62.50%) | | 0.540 | | 0.686 (0.205-2.295) | | 0.541 | |
| hyomandibular up | | Control | | | 24 | 0.71 | | 7 (29.17%) | | | 17 (70.83%) | |  | | 1 | |  | |
|  | | LDC 3 g | | | 24 | 0.63 | | 9 (37.50%) | | | 15 (62.50%) | | 0.540 | | 0.686 (0.205-2.295) | | 0.541 | |
| maxilla down | | Control | | | 24 | 0.13 | | 21 (87.50%) | | | 3 (12.50%) | |  | | 1 | |  | |
|  | | LDC 1g > 3g | | | 24 | 0.08 | | 22 (91.67%) | | | 2 (8.33%) | | 0.637 | | 0.636 (0.096-4.197) | | 0.639 | |
| maxilla up | | Control | | | 24 | 0.13 | | 21 (87.50%) | | | 3 (12.50%) | |  | | 1 | |  | |
|  | | LDC 1g > 3 g | | | 24 | 0.08 | | 22 (91.67%) | | | 2 (8.33%) | | 0.637 | | 0.636 (0.096-4.197) | | 0.639 | |
| B | |  | | |  |  | |  | | |  | |  | |  | |  | |
|  |  | | |  |  | | Score of ossificaiton (Y) | | | | | | X² pearson | Ordinal logistic regression | | | | |
| Structures | Treat | | | N | Mean | absence | | | | early | | advanced | p-value | OR (IC 95%) | | | | p-value |
| anguloarticular down | Control | | | 24 | 1.08 | 8 (33.33%) | | | | 6 (25.00%) | | 10 (41.67%) |  | 1 | | | |  |
|  | LDC 1g > 3g | | | 24 | 1.83 | 1 (4.17%) | | | | 2 (8.33%) | | 21 (87.50%) | **0.003** | 9.993 (2.360-42.315) | | | | **0.002** |
| anguloarticular up | Control | | | 24 | 1.04 | 9 (37.50%) | | | | 5 (20.83%) | | 10 (41.67%) |  | 1 | | | |  |
|  | LDC 3 g | | | 24 | 1.83 | 1 (4.17%) | | | | 2 (8.33%) | | 21 (87.50%) | **0.003** | 10.249 (2.413-43.538) | | | | **0.002** |
| branchiostegal ray2 down | Control | | | 24 | 0.92 | 7 (29.17%) | | | | 12 (50.00%) | | 5 (20.83%) |  | 1 | | | |  |
|  | LDC 1g > 3g | | | 24 | 0.96 | 8 (33.33%) | | | | 9 (37.50%) | | 7 (29.17%) | 0.661 | 1.094 (0.382-3.129) | | | | 0.867 |
| branchiostegal ray2 up | Control | | | 24 | 1.04 | 7 (29.17%) | | | | 9 (37.50%) | | 8 (33.33%) |  | 1 | | | |  |
|  | LDC 3 g | | | 24 | 1.00 | 7 (29.17%) | | | | 10 (41.67%) | | 7 (29.17%) | 0.942 | 0.904 (0.319-2.568) | | | | 0.850 |
| ceratohyal down | Control | | | 24 | 0.54 | 11 (45.83%) | | | | 13 (54.17%) | | 0 (0.00%) |  | 1 | | | |  |
|  | LDC 1g > 3g | | | 24 | 1.33 | 3 (12.50%) | | | | 10 (41.67%) | | 11 (4583%) | **<0.001** | 12.584 (3.063-51.701) | | | | **<0.001** |
| ceratohyal up | Control | | | 24 | 0.54 | 11 (45.8%) | | | | 13 (54.17%) | | 0 (0.00%) |  | 1 | | | |  |
|  | LDC 1g > 3g | | | 24 | 1.38 | 2 (8.33%) | | | | 11 (45.83%) | | 11 (45.83%) | **<0.001** | 19.388 (3.831-98.128) | | | | **<0.001** |
