# Supplementary material for: Zebrafish Bone and General Physiology Are Differently Affected by Hormones or Changes in Gravity
Source: PLoS One. 2015 Jun 10;10(6):e0126928. doi: 10.1371/journal.pone.0126928 (PMC4465622; doi:10.1371/journal.pone.0126928)
Supplement: S9 Table — The indicates the human homolog of the gene, its "Entrez" gene name, the log ratio compared to larvae kept at 1g between 0 and 6dpf, the presence of duplicate probes on the microarray (D) and the type of protein it encodes. Genes are arranged according to their type and in alphabetical order. (DOC) [file pone.0126928.s016.doc]

| **Symbol** | **Entrez Gene Name** | | **Log Ratio**  **1g>3g** | | **p-value** | | **D** | | **Type(s)** | |
| --- | --- | --- | --- | --- | --- | --- | --- | --- | --- | --- |
| **ABCA5** | ATP-binding cassette. sub-family A (ABC1). member 5 | | -0.223 | | 8.38E-02 | |  | | transporter | |
| **APOA4** | apolipoprotein A-IV | | -0.353 | | 9.57E-02 | |  | | transporter | |
| **AQP3** | aquaporin 3 (Gill blood group) | | -0.166 | | 7.48E-02 | |  | | transporter | |
| **HBE1** | hemoglobin. epsilon 1 | | -0.311 | | 7.58E-02 | |  | | transporter | |
| **KCND3** | potassium voltage-gated channel. Shal-related subfamily. member 3 | | -0.252 | | 4.52E-02 | | D | | ion channel | |
| **KCND3** | potassium voltage-gated channel. Shal-related subfamily. member 3 | | -0.195 | | 8.61E-02 | | D | | ion channel | |
| **KPNA4** | karyopherin alpha 4 (importin alpha 3) | | 0.149 | | 8.59E-02 | |  | | transporter | |
| **NUP133** | nucleoporin 133kDa | | 0.188 | | 8.61E-02 | | D | | transporter | |
| **NUP133** | nucleoporin 133kDa | | 0.182 | | 9.53E-02 | | D | | transporter | |
| **REEP5** | receptor accessory protein 5 | | -0.280 | | 6.37E-02 | |  | | transporter | |
| **RHCG** | Rh family. C glycoprotein | | -0.556 | | 4.61E-02 | |  | | transporter | |
| **SCN5A** | sodium channel. voltage-gated. type V. alpha subunit | | -0.253 | | 7.07E-02 | |  | | ion channel | |
| **SEC23B** | Sec23 homolog B (S. cerevisiae) | | -0.251 | | 7.34E-02 | |  | | transporter | |
| **SEH1L** | SEH1-like (S. cerevisiae) | | -0.174 | | 7.69E-02 | |  | | transporter | |
| **SLC15A1** | solute carrier family 15 (oligopeptide transporter). member 1 | | -0.485 | | 5.01E-02 | | D | | transporter | |
| **SLC15A1** | solute carrier family 15 (oligopeptide transporter). member 1 | | -0.487 | | 8.61E-02 | | D | | transporter | |
| **SLC25A26** | solute carrier family 25 (S-adenosylmethionine carrier). member 26 | | -0.114 | | 7.99E-02 | |  | | transporter | |
| **SLC25A43** | solute carrier family 25. member 43 | | -0.496 | | 6.86E-02 | |  | | transporter | |
| **SLC5A6** | solute carrier family 5 (sodium/multivitamin and iodide cotransporter). member 6 | | 0.101 | | 9.94E-02 | |  | | transporter | |
| **SLC6A19** | solute carrier family 6 (neutral amino acid transporter). member 19 | | -0.185 | | 6.64E-02 | |  | | transporter | |
| **SLC9A3** | solute carrier family 9. subfamily A (NHE3. cation proton antiporter 3). member 3 | | -0.183 | | 9.20E-02 | |  | | ion channel | |
| **SYT11** | synaptotagmin XI | | -0.302 | | 5.97E-02 | |  | | transporter | |
| **VDAC3** | voltage-dependent anion channel 3 | | -0.155 | | 8.43E-02 | |  | | ion channel | |
| **VPS9D1** | VPS9 domain containing 1 | | -0.167 | | 9.22E-02 | |  | | transporter | |
| **ANKRD33** | ankyrin repeat domain 33 | | -0.135 | | 6.71E-02 | |  | | transcription regulator | |
| **ATF3** | activating transcription factor 3 | | -0.433 | | 8.23E-02 | |  | | transcription regulator | |
| **CITED2** | Cbp/p300-interacting transactivator. with Glu/Asp-rich carboxy-terminal domain. 2 | | 0.310 | | 8.61E-02 | |  | | transcription regulator | |
| **EIF5** | eukaryotic translation initiation factor 5 | | 0.222 | | 6.64E-02 | |  | | translation regulator | |
| **FOXD3** | forkhead box D3 | | -0.213 | | 4.52E-02 | |  | | transcription regulator | |
| **FOXP4** | forkhead box P4 | | -0.233 | | 6.64E-02 | |  | | transcription regulator | |
| **HDAC2** | histone deacetylase 2 | | 0.261 | | 7.07E-02 | |  | | transcription regulator | |
| **HDAC4** | histone deacetylase 4 | | -0.371 | | 6.30E-02 | |  | | transcription regulator | |
| **HEY1** | hes-related family bHLH transcription factor with YRPW motif 1 | | 0.130 | | 7.05E-02 | |  | | transcription regulator | |
| **KLF7** | Kruppel-like factor 7 (ubiquitous) | | -0.203 | | 5.97E-02 | |  | | transcription regulator | |
| **MEIS1** | Meis homeobox 1 | | -0.264 | | 5.79E-02 | |  | | transcription regulator | |
| **MYC** | v-myc avian myelocytomatosis viral oncogene homolog | | -0.587 | | 3.95E-02 | | D | | transcription regulator | |
| **MYC** | v-myc avian myelocytomatosis viral oncogene homolog | | -0.567 | | 4.52E-02 | | D | | transcription regulator | |
| **NFKBIA** | nuclear factor of kappa light polypeptide gene enhancer in B-cells inhibitor. alpha | | -0.475 | | 4.52E-02 | |  | | transcription regulator | |
| **NR1D1** | nuclear receptor subfamily 1. group D. member 1 | | -1.161 | | 5.79E-02 | |  | | ligand-dependent nuclear receptor | |
| **ONECUT1** | one cut homeobox 1 | | -0.097 | | 9.67E-02 | |  | | transcription regulator | |
| **POU3F3** | POU class 3 homeobox 3 | | 0.144 | | 7.29E-02 | |  | | transcription regulator | |
| **PPARG** | peroxisome proliferator-activated receptor gamma | | -0.318 | | 9.08E-02 | |  | | ligand-dependent nuclear receptor | |
| **PURA** | purine-rich element binding protein A | | -0.304 | | 7.75E-02 | |  | | transcription regulator | |
| **SHOX** | short stature homeobox | | -0.261 | | 5.77E-02 | |  | | transcription regulator | |
| **SIN3B** | SIN3 transcription regulator family member B | | 0.216 | | 6.79E-02 | |  | | transcription regulator | |
| **SOX3** | SRY (sex determining region Y)-box 3 | | 0.298 | | 9.51E-02 | |  | | transcription regulator | |
| **TANC2** | tetratricopeptide repeat. ankyrin repeat and coiled-coil containing 2 | | -0.155 | | 6.64E-02 | |  | | transcription regulator | |
| **TFAP2B** | transcription factor AP-2 beta (activating enhancer binding protein 2 beta) | | 0.144 | | 9.16E-02 | |  | | transcription regulator | |
| **TOB1** | transducer of ERBB2. 1 | | -0.325 | | 7.13E-02 | |  | | transcription regulator | |
| **VDR** | vitamin D (1.25- dihydroxyvitamin D3) receptor | | -0.245 | | 5.77E-02 | | D | | transcription regulator | |
| **VDR** | vitamin D (1.25- dihydroxyvitamin D3) receptor | | -0.240 | | 6.75E-02 | | D | | transcription regulator | |
| **ANTXR2** | anthrax toxin receptor 2 | | -0.235 | | 6.81E-02 | |  | | transmembrane receptor | |
| **CLK4** | CDC-like kinase 4 | | 0.615 | | 9.30E-02 | |  | | kinase | |
| **CSNK1A1L** | casein kinase 1. alpha 1-like | | -0.237 | | 8.43E-02 | |  | | kinase | |
| **DUSP2** | dual specificity phosphatase 2 | | -0.388 | | 4.52E-02 | |  | | phosphatase | |
| **DUSP5** | dual specificity phosphatase 5 | | -0.695 | | 8.43E-02 | |  | | phosphatase | |
| **GRK7** | G protein-coupled receptor kinase 7 | | -0.541 | | 4.52E-02 | |  | | kinase | |
| **IP6K2** | inositol hexakisphosphate kinase 2 | | 0.256 | | 4.52E-02 | |  | | kinase | |
| **JAK1** | Janus kinase 1 | | -0.198 | | 8.51E-02 | |  | | kinase | |
| **MAPK4** | mitogen-activated protein kinase 4 | | -0.111 | | 9.30E-02 | |  | | kinase | |
| **NT5C3A** | 5'-nucleotidase. cytosolic IIIA | | 0.259 | | 6.64E-02 | | D | | phosphatase | |
| **NT5C3A** | 5'-nucleotidase. cytosolic IIIA | | 0.264 | | 6.95E-02 | | D | | phosphatase | |
| **PFKFB4** | 6-phosphofructo-2-kinase/fructose-2.6-biphosphatase 4 | | -0.473 | | 6.64E-02 | |  | | kinase | |
| **PHKG1** | phosphorylase kinase. gamma 1 (muscle) | | -0.220 | | 9.94E-02 | |  | | kinase | |
| **PIM2** | pim-2 oncogene | | 0.335 | | 9.30E-02 | |  | | kinase | |
| **PRKAR1A** | protein kinase. cAMP-dependent. regulatory. type I. alpha | | 0.236 | | 7.13E-02 | |  | | kinase | |
| **PTPN1** | protein tyrosine phosphatase. non-receptor type 1 | | 0.163 | | 7.63E-02 | |  | | phosphatase | |
| **SGK1** | serum/glucocorticoid regulated kinase 1 | | -0.517 | | 7.07E-02 | |  | | kinase | |
| **STK35** | serine/threonine kinase 35 | | -0.634 | | 6.30E-02 | |  | | kinase | |
| **STRADA** | STE20-related kinase adaptor alpha | | 0.177 | | 7.07E-02 | |  | | kinase | |
| **BACE1** | beta-site APP-cleaving enzyme 1 | | 0.118 | | 8.57E-02 | |  | | peptidase | |
| **CFB** | | complement factor B | | -0.281 | | 5.79E-02 | |  | | peptidase |
| **CTSB** | cathepsin B | | 0.247 | | 6.11E-02 | |  | | peptidase | |
| **ENDOU** | endonuclease. polyU-specific | | -0.139 | | 6.64E-02 | |  | | peptidase | |
| **UCHL5** | ubiquitin carboxyl-terminal hydrolase L5 | | 0.120 | | 9.76E-02 | |  | | peptidase | |
| **USP14** | ubiquitin specific peptidase 14 (tRNA-guanine transglycosylase) | | 0.150 | | 9.22E-02 | |  | | peptidase | |
| **USP37** | ubiquitin specific peptidase 37 | | 0.207 | | 6.79E-02 | |  | | peptidase | |
| **ACBD6** | acyl-CoA binding domain containing 6 | | 0.182 | | 6.81E-02 | |  | | other | |
| **ACTA2** | actin. alpha 2. smooth muscle. aorta | | -0.141 | | 6.64E-02 | |  | | other | |
| **ADD3** | adducin 3 (gamma) | | -0.185 | | 8.98E-02 | |  | | other | |
| **AHCY** | adenosylhomocysteinase | | 0.149 | | 8.98E-02 | |  | | enzyme | |
| **ALAS2** | aminolevulinate. delta-. synthase 2 | | -0.111 | | 9.94E-02 | |  | | enzyme | |
| **ALDH4A1** | aldehyde dehydrogenase 4 family. member A1 | | -0.134 | | 8.43E-02 | |  | | enzyme | |
| **ALDH8A1** | aldehyde dehydrogenase 8 family. member A1 | | -0.202 | | 4.89E-02 | |  | | enzyme | |
| **ANXA1** | annexin A1 | | -0.416 | | 5.29E-02 | |  | | enzyme | |
| **ANXA4** | annexin A4 | | -0.421 | | 4.52E-02 | |  | | other | |
| **ARL5C** | ADP-ribosylation factor-like 5C | | -0.700 | | 5.19E-02 | |  | | other | |
| **ARR3** | arrestin 3. retinal (X-arrestin) | | -0.236 | | 6.95E-02 | |  | | other | |
| **ARRDC2** | arrestin domain containing 2 | | -0.427 | | 6.08E-02 | |  | | other | |
| **ATG10** | autophagy related 10 | | -0.108 | | 8.43E-02 | |  | | enzyme | |
| **ATL2** | atlastin GTPase 2 | | 0.214 | | 8.43E-02 | |  | | other | |
| **B3GAT2** | beta-1.3-glucuronyltransferase 2 (glucuronosyltransferase S) | | -0.315 | | 4.52E-02 | |  | | enzyme | |
| **BCMO1** | beta-carotene 15.15'-monooxygenase 1 | | -0.126 | | 7.07E-02 | |  | | enzyme | |
| **BLOC1S6** | biogenesis of lysosomal organelles complex-1. subunit 6. pallidin | | -0.252 | | 8.43E-02 | |  | | other | |
| **BOC** | BOC cell adhesion associated. oncogene regulated | | -0.167 | | 5.74E-02 | |  | | other | |
| **C10orf54** | chromosome 10 open reading frame 54 | | -0.116 | | 7.69E-02 | |  | | other | |
| **CA10** | carbonic anhydrase X | | -0.274 | | 4.52E-02 | | D | | enzyme | |
| **CA10** | carbonic anhydrase X | | -0.246 | | 6.37E-02 | | D | | enzyme | |
| **CA10** | carbonic anhydrase X | | -0.328 | | 6.64E-02 | | D | | enzyme | |
| **CAPG** | capping protein (actin filament). gelsolin-like | | -0.182 | | 6.64E-02 | |  | | other | |
| **CCDC124** | coiled-coil domain containing 124 | | -0.240 | | 5.77E-02 | |  | | other | |
| **CCDC85C** | coiled-coil domain containing 85C | | -0.323 | | 8.70E-02 | |  | | other | |
| **CDH7** | cadherin 7. type 2 | | -0.190 | | 5.77E-02 | |  | | other | |
| **CEP63** | centrosomal protein 63kDa | | 0.233 | | 5.23E-02 | |  | | other | |
| **CISH** | cytokine inducible SH2-containing protein | | -0.410 | | 4.52E-02 | | D | | other | |
| **CISH** | cytokine inducible SH2-containing protein | | -0.510 | | 7.75E-02 | | D | | other | |
| **CLDN9** | claudin 9 | | -0.349 | | 9.67E-02 | |  | | other | |
| **CRHBP** | corticotropin releasing hormone binding protein | | -0.220 | | 5.72E-02 | |  | | other | |
| **CSDC2** | cold shock domain containing C2. RNA binding | | -0.190 | | 6.64E-02 | |  | | other | |
| **CTDSPL** | CTD (carboxy-terminal domain. RNA polymerase II. polypeptide A) small phosphatase-like | | -0.253 | | 5.81E-02 | |  | | other | |
| **Cyp2ac1** | cytochrome P450. family 2. subfamily ac. polypeptide 1 | | 0.114 | | 8.43E-02 | |  | | other | |
| **CYP2J2** | cytochrome P450. family 2. subfamily J. polypeptide 2 | | 0.424 | | 6.37E-02 | |  | | enzyme | |
| **CYP2R1** | cytochrome P450. family 2. subfamily R. polypeptide 1 | | -0.465 | | 7.48E-02 | |  | | enzyme | |
| **CYR61** | cysteine-rich. angiogenic inducer. 61 | | -0.369 | | 5.77E-02 | |  | | other | |
| **DDX18** | DEAD (Asp-Glu-Ala-Asp) box polypeptide 18 | | 0.214 | | 8.68E-02 | |  | | enzyme | |
| **DDX51** | DEAD (Asp-Glu-Ala-Asp) box polypeptide 51 | | 0.179 | | 7.95E-02 | |  | | enzyme | |
| **ELOVL1** | ELOVL fatty acid elongase 1 | | -0.305 | | 4.52E-02 | |  | | enzyme | |
| **EMC1** | ER membrane protein complex subunit 1 | | 0.220 | | 5.43E-02 | |  | | other | |
| **ERGIC2** | ERGIC and golgi 2 | | -0.161 | | 6.41E-02 | |  | | other | |
| **ERRFI1** | ERBB receptor feedback inhibitor 1 | | -0.390 | | 6.41E-02 | |  | | other | |
| **FAM78B** | family with sequence similarity 78. member B | | -0.241 | | 7.69E-02 | |  | | other | |
| **FBLN7** | fibulin 7 | | -0.216 | | 5.77E-02 | |  | | other | |
| **FBXL3** | F-box and leucine-rich repeat protein 3 | | -0.310 | | 6.81E-02 | |  | | enzyme | |
| **FITM2** | fat storage-inducing transmembrane protein 2 | | -0.179 | | 9.94E-02 | |  | | other | |
| **FKBP5** | FK506 binding protein 5 | | -1.230 | | 6.86E-02 | |  | | enzyme | |
| **FUCA1** | fucosidase. alpha-L- 1. tissue | | -0.265 | | 7.07E-02 | |  | | enzyme | |
| **GADD45B** | growth arrest and DNA-damage-inducible. beta | | -0.230 | | 9.08E-02 | |  | | other | |
| **GBP1** | guanylate binding protein 1. interferon-inducible | | 0.128 | | 8.51E-02 | |  | | enzyme | |
| **GNAO1** | guanine nucleotide binding protein (G protein). alpha activating activity polypeptide O | | -0.278 | | 8.43E-02 | |  | | enzyme | |
| **GOT1** | glutamic-oxaloacetic transaminase 1. soluble | | 0.218 | | 6.95E-02 | |  | | enzyme | |
| **GPR137C** | G protein-coupled receptor 137C | | -0.329 | | 5.16E-02 | |  | | other | |
| **GRB2** | growth factor receptor-bound protein 2 | | -0.417 | | 3.03E-02 | |  | | other | |
| **GSTZ1** | glutathione S-transferase zeta 1 | | -0.211 | | 6.39E-02 | |  | | enzyme | |
| **GUSB** | glucuronidase. beta | | -0.147 | | 9.30E-02 | |  | | enzyme | |
| **HRAS** | Harvey rat sarcoma viral oncogene homolog | | -0.190 | | 8.43E-02 | |  | | enzyme | |
| **HRSP12** | heat-responsive protein 12 | | -0.162 | | 6.81E-02 | |  | | other | |
| **HSD11B2** | hydroxysteroid (11-beta) dehydrogenase 2 | | -0.383 | | 4.52E-02 | |  | | enzyme | |
| **HSD17B12** | hydroxysteroid (17-beta) dehydrogenase 12 | | -0.404 | | 3.58E-02 | |  | | enzyme | |
| **IGHMBP2** | immunoglobulin mu binding protein 2 | | 0.170 | | 6.81E-02 | |  | | enzyme | |
| **IMPACT** | impact RWD domain protein | | -0.106 | | 8.60E-02 | |  | | other | |
| **IMPDH2** | IMP (inosine 5'-monophosphate) dehydrogenase 2 | | -0.149 | | 6.86E-02 | |  | | enzyme | |
| **ITM2C** | integral membrane protein 2C | | -0.401 | | 4.52E-02 | |  | | other | |
| **KCTD5** | potassium channel tetramerization domain containing 5 | | -0.346 | | 9.94E-02 | |  | | other | |
| **KDSR** | 3-ketodihydrosphingosine reductase | | -0.147 | | 8.21E-02 | |  | | enzyme | |
| **LPL** | lipoprotein lipase | | -0.197 | | 6.64E-02 | |  | | enzyme | |
| **LRIT3** | leucine-rich repeat. immunoglobulin-like and transmembrane domains 3 | | -0.167 | | 6.37E-02 | |  | | other | |
| **LRPPRC** | leucine-rich pentatricopeptide repeat containing | | 0.305 | | 6.97E-02 | |  | | other | |

| **MBOAT2** | membrane bound O-acyltransferase domain containing 2 | 0.189 | 5.81E-02 |  | enzyme |
| --- | --- | --- | --- | --- | --- |
| **MED18** | mediator complex subunit 18 | 0.222 | 7.01E-02 | D | other |
| **MED18** | mediator complex subunit 18 | 0.243 | 7.07E-02 | D | other |
| **MGST1** | microsomal glutathione S-transferase 1 | -0.207 | 6.97E-02 |  | enzyme |
| **MIDN** | midnolin | -0.619 | 6.05E-02 |  | other |
| **MKLN1** | muskelin 1. intracellular mediator containing kelch motifs | 0.183 | 8.61E-02 |  | other |
| **MTMR10** | myotubularin related protein 10 | 0.264 | 8.98E-02 |  | other |
| **MTR** | 5-methyltetrahydrofolate-homocysteine methyltransferase | 0.152 | 8.21E-02 | D | enzyme |
| **MTR** | 5-methyltetrahydrofolate-homocysteine methyltransferase | 0.227 | 8.43E-02 | D | enzyme |
| **MYLPF** | myosin light chain. phosphorylatable. fast skeletal muscle | 0.280 | 4.72E-02 |  | other |
| **MYO9B** | myosin IXB | 0.190 | 5.18E-02 |  | enzyme |
| **NAP1L1** | nucleosome assembly protein 1-like 1 | -0.227 | 4.61E-02 | D | other |
| **NAP1L1** | nucleosome assembly protein 1-like 1 | -0.234 | 5.43E-02 | D | other |
| **NDC1** | NDC1 transmembrane nucleoporin | 0.160 | 6.81E-02 |  | other |
| **NDRG2** | NDRG family member 2 | -0.754 | 8.23E-02 |  | other |
| **NDUFAF5** | NADH dehydrogenase (ubiquinone) complex I. assembly factor 5 | -0.150 | 6.20E-02 |  | other |
| **NHLH2** | nescient helix loop helix 2 | -0.096 | 9.66E-02 |  | other |
| **NSA2** | NSA2 ribosome biogenesis homolog (S. cerevisiae) | -0.347 | 6.45E-02 |  | other |
| **OGT** | O-linked N-acetylglucosamine (GlcNAc) transferase | 0.169 | 7.13E-02 |  | enzyme |
| **OLIG3** | oligodendrocyte transcription factor 3 | 0.174 | 6.64E-02 |  | other |
| **ORC4** | origin recognition complex. subunit 4 | 0.124 | 7.69E-02 |  | other |
| **PCDH7** | protocadherin 7 | -0.269 | 6.64E-02 |  | other |
| **PCDHA8** | protocadherin alpha 8 | -0.253 | 8.61E-02 |  | other |
| **PCNA** | proliferating cell nuclear antigen | 0.225 | 4.52E-02 |  | enzyme |
| **PDE6C** | phosphodiesterase 6C. cGMP-specific. cone. alpha prime | 0.371 | 8.43E-02 |  | enzyme |
| **PHF10** | PHD finger protein 10 | 0.143 | 6.64E-02 |  | other |
| **PHYHIPL** | phytanoyl-CoA 2-hydroxylase interacting protein-like | -0.368 | 5.77E-02 |  | other |
| **PRPF39** | pre-mRNA processing factor 39 | 0.240 | 9.16E-02 |  | other |
| **PRPF4** | pre-mRNA processing factor 4 | 0.129 | 9.91E-02 |  | other |
| **PRPF40A** | PRP40 pre-mRNA processing factor 40 homolog A (S. cerevisiae) | 0.113 | 9.51E-02 |  | other |
| **PSMD11** | proteasome (prosome. macropain) 26S subunit. non-ATPase. 11 | 0.173 | 7.07E-02 |  | other |
| **PTCD3** | pentatricopeptide repeat domain 3 | 0.258 | 4.52E-02 | D | other |
| **PTCD3** | pentatricopeptide repeat domain 3 | 0.203 | 5.77E-02 | D | other |
| **PTCD3** | pentatricopeptide repeat domain 3 | 0.253 | 5.81E-02 | D | other |
| **PTCD3** | pentatricopeptide repeat domain 3 | 0.176 | 8.51E-02 | D | other |
| **PVALB** | parvalbumin | -0.290 | 4.52E-02 |  | other |
| **PWP1** | PWP1 homolog (S. cerevisiae) | 0.275 | 4.52E-02 | D | other |
| **PWP1** | PWP1 homolog (S. cerevisiae) | 0.241 | 5.77E-02 | D | other |
| **RAB10** | RAB10. member RAS oncogene family | -0.292 | 6.78E-02 |  | enzyme |
| **RABGGTB** | Rab geranylgeranyltransferase. beta subunit | 0.143 | 9.30E-02 |  | enzyme |
| **RFNG** | RFNG O-fucosylpeptide 3-beta-N-acetylglucosaminyltransferase | 0.219 | 9.78E-02 |  | enzyme |
| **RND3** | Rho family GTPase 3 | -0.236 | 8.43E-02 |  | enzyme |
| **RNF180** | ring finger protein 180 | 0.197 | 7.07E-02 |  | enzyme |
| **RNF7** | ring finger protein 7 | -0.144 | 7.69E-02 |  | enzyme |
| **RPL14** | ribosomal protein L14 | -0.231 | 8.59E-02 |  | other |
| **RPS19** | ribosomal protein S19 | -0.218 | 5.43E-02 | D | other |
| **RPS19** | ribosomal protein S19 | -0.295 | 7.40E-02 | D | other |
| **RPS28** | ribosomal protein S28 | -0.317 | 4.52E-02 |  | other |
| **RSL24D1** | ribosomal L24 domain containing 1 | -0.389 | 7.07E-02 |  | other |
| **RSPO1** | R-spondin 1 | -0.125 | 8.43E-02 |  | other |
| **RUFY2** | RUN and FYVE domain containing 2 | 0.188 | 5.79E-02 |  | other |
| **SEMA3F** | sema domain. immunoglobulin domain (Ig). short basic domain. secreted. (semaphorin) 3F | -0.346 | 6.39E-02 |  | other |
| **SERAC1** | serine active site containing 1 | 0.186 | 5.74E-02 | D | other |
| **SERAC1** | serine active site containing 1 | 0.188 | 6.64E-02 | D | other |
| **SETD3** | SET domain containing 3 | 0.219 | 9.94E-02 |  | enzyme |
| **SH3GL1** | SH3-domain GRB2-like 1 | -0.337 | 8.98E-02 |  | other |
| **SLTM** | SAFB-like. transcription modulator | 0.354 | 9.58E-02 |  | other |
| **SNF8** | SNF8. ESCRT-II complex subunit | -0.111 | 8.43E-02 | D | enzyme |
| **SNF8** | SNF8. ESCRT-II complex subunit | -0.178 | 9.67E-02 | D | enzyme |
| **SOCS1** | suppressor of cytokine signaling 1 | -0.827 | 4.52E-02 |  | other |
| **SPRY4** | sprouty homolog 4 (Drosophila) | -0.497 | 6.11E-02 | D | other |
| **SPRY4** | sprouty homolog 4 (Drosophila) | -0.569 | 6.37E-02 | D | other |
| **SPRYD3** | SPRY domain containing 3 | -0.353 | 4.52E-02 |  | other |
| **TOLLIP** | toll interacting protein | -0.314 | 5.19E-02 |  | other |
| **TPD52** | tumor protein D52 | -0.221 | 9.17E-02 |  | other |
| **Tsc22d3** | TSC22 domain family. member 3 | -0.280 | 6.64E-02 |  | other |
| **TSKU** | tsukushi. small leucine rich proteoglycan | -0.187 | 7.07E-02 |  | other |
| **TXNIP** | thioredoxin interacting protein | 0.902 | 2.04E-02 | D | other |
| **TXNIP** | thioredoxin interacting protein | 0.911 | 5.79E-02 | D | other |
| **UBR7** | ubiquitin protein ligase E3 component n-recognin 7 (putative) | 0.302 | 5.77E-02 | D | enzyme |
| **UBR7** | ubiquitin protein ligase E3 component n-recognin 7 (putative) | 0.350 | 7.01E-02 | D | enzyme |
| **WDR13** | WD repeat domain 13 | 0.125 | 9.08E-02 |  | other |
| **WDR6** | WD repeat domain 6 | 0.108 | 8.61E-02 |  | other |
| **YARS** | tyrosyl-tRNA synthetase | -0.155 | 8.12E-02 |  | enzyme |
| **ZC3H11A** | zinc finger CCCH-type containing 11A | 0.183 | 5.77E-02 |  | other |
| **ZNF729** | zinc finger protein 729 | -0.158 | 9.47E-02 |  | other |
| **ZSWIM8** | zinc finger. SWIM-type containing 8 | -0.145 | 7.13E-02 |  | other |
