# Supplementary material for: Zebrafish Bone and General Physiology Are Differently Affected by Hormones or Changes in Gravity
Source: PLoS One. 2015 Jun 10;10(6):e0126928. doi: 10.1371/journal.pone.0126928 (PMC4465622; doi:10.1371/journal.pone.0126928)
Supplement: S10 Table — The fold change and statistical significance (p-values) are given from the microarray data and the RT-qPCR confirmation experiments. (DOCX) [file pone.0126928.s017.docx]

Table S10

|  | **microarray** | |  | **RT-PCR** | |
| --- | --- | --- | --- | --- | --- |
| **Gene** | **Fold Change** | **p-value** |  | **Fold Change** | **p-value** |
| *nr1d1* | 0,447 | 0,058 |  | 0,518 | < 0,001 |
| *rhcg* | 0,68 | 0,046 |  | 0,779 | < 0,001 |
| *socs1* | 0,564 | 0,045 |  | 0,544 | < 0,001 |
| *spry4* | 0,674 | 0,064 |  | 0,739 | < 0,001 |
| *txnip* | 1,88 | 0,058 |  | 2,555 | < 0,001 |
