# Supplementary material for: Zebrafish Bone and General Physiology Are Differently Affected by Hormones or Changes in Gravity
Source: PLoS One. 2015 Jun 10;10(6):e0126928. doi: 10.1371/journal.pone.0126928 (PMC4465622; doi:10.1371/journal.pone.0126928)
Supplement: S11 Table — Ingenuity Pathway Analysis of the list of genes affected at 6dpf after 3g hypergravity treatment for 24 hours (1g>3g). Columns indicate respectively the category of function, the range of p-values (significance) associated to various sub-functions, and the number of genes concerned. (DOCX) [file pone.0126928.s018.docx]

| **Category** | **p-value** | | **Number of Genes** |
| --- | --- | --- | --- |
| Cellular Growth and Proliferation | | 2.42^E-07^-9.08^E-03^ | 66 |
| Molecular Transport | | 3.83^E-07^-9.08^E-03^ | 46 |
| Cellular Development | | 3.53^E-06^-9.08^E-03^ | 64 |
| Embryonic Development | | 1.44^E-05^-9.08^E-03^ | 36 |
| Cell Death and Survival | | 2.1^E-05^-9.08^E-03^ | 66 |
| Organ Development | | 2.51^E-05^-5.47^E-03^ | 29 |
| Organismal Development | | 2.51^E-05^-9.08^E-03^ | 57 |
| Skeletal and Muscular System Development and Function | | 2.51^E-05^-9.08^E-03^ | 25 |
| Tissue Development | | 2.51^E-05^-9.08^E-03^ | 50 |
| Connective Tissue Development and Function | | 3.48^E-05^-9.08^E-03^ | 22 |
| Nervous System Development and Function | | 3.98^E-05^-9.08^E-03^ | 14 |
| Endocrine System Development and Function | | 5.3^E-05^-9.04^E-03^ | 19 |
| Lipid Metabolism | | 5.3^E-05^-9.08^E-03^ | 23 |
| Small Molecule Biochemistry | | 5.3^E-05^-9.08^E-03^ | 39 |
| Gene Expression | | 6.34^E-05^-9.08^E-03^ | 46 |
| Organismal Survival | | 7.23^E-05^-5.31^E-03^ | 49 |
| Cell Morphology | | 8.19^E-05^-9.08^E-03^ | 40 |
| Hair and Skin Development and Function | | 1.16^E-04^-9.08^E-03^ | 14 |
| Renal and Urological System Development and Function | | 1.97^E-04^-1.67^E-03^ | 11 |
| Reproductive System Development and Function | | 1.97^E-04^-9.08^E-03^ | 12 |
| Tissue Morphology | | 1.98^E-04^-9.04^E-03^ | 44 |
| Cell Cycle | | 2.44^E-04^-9.08^E-03^ | 25 |
| Cell-To-Cell Signaling and Interaction | | 2.67^E-04^-9.08^E-03^ | 19 |
| Cellular Assembly and Organization | | 2.67^E-04^-9.08^E-03^ | 11 |
| Cellular Movement | | 4.34^E-04^-9.08^E-03^ | 40 |
| Hematological System Development and Function | | 4.37^E-04^-9.08^E-03^ | 34 |
| Hematopoiesis | | 4.37^E-04^-8.39^E-03^ | 23 |
| Cellular Function and Maintenance | | 4.86^E-04^-9.08^E-03^ | 13 |
| Carbohydrate Metabolism | | 6.66^E-04^-9.08^E-03^ | 23 |
| Organ Morphology | | 8.7^E-04^-9.08^E-03^ | 24 |
| Lymphoid Tissue Structure and Development | | 1.06^E-03^-8.47^E-03^ | 7 |
| Cardiovascular System Development and Function | | 1.14^E-03^-9.08^E-03^ | 29 |
| Amino Acid Metabolism | | 1.67^E-03^-9.08^E-03^ | 8 |
| Vitamin and Mineral Metabolism | | 1.83^E-03^-9.08^E-03^ | 8 |
| DNA Replication. Recombination. and Repair | | 1.91^E-03^-6.16^E-03^ | 15 |
| Digestive System Development and Function | | 2.26^E-03^-9.08^E-03^ | 17 |
| Behavior | | 2.28^E-03^-2.28^E-03^ | 5 |
| Hepatic System Development and Function | | 2.8^E-03^-2.8^E-03^ | 7 |
| Respiratory System Development and Function | | 2.83^E-03^-4.27^E-03^ | 2 |
| Nucleic Acid Metabolism | | 4.27^E-03^-9.08^E-03^ | 2 |
| Protein Synthesis | | 4.27^E-03^-9.04^E-03^ | 25 |
| Humoral Immune Response | | 6.71^E-03^-6.71^E-03^ | 7 |
| Post-Translational Modification | | 6.94^E-03^-6.94^E-03^ | 2 |
| Immune Cell Trafficking | | 7.97^E-03^-9.08^E-03^ | 13 |
| Free Radical Scavenging | | 8.78^E-03^-8.78^E-03^ | 12 |
| Cell-mediated Immune Response | | 9.08^E-03^-9.08^E-03^ | 1 |
| Cellular Compromise | | 9.08^E-03^-9.08^E-03^ | 1 |
| Cellular Response to Therapeutics | | 9.08^E-03^-9.08^E-03^ | 1 |
| Drug Metabolism | | 9.08^E-03^-9.08^E-03^ | 4 |

Table S11
