# Supplementary material for: Zebrafish Bone and General Physiology Are Differently Affected by Hormones or Changes in Gravity
Source: PLoS One. 2015 Jun 10;10(6):e0126928. doi: 10.1371/journal.pone.0126928 (PMC4465622; doi:10.1371/journal.pone.0126928)
Supplement: S12 Table — The fraction (in %) of larvae presenting the indicated score for each element is given, together with the statistical evaluation of a significant difference compared to control. (A) The bone structures distributed in 2 categories (early and advanced ossification) (B) The bone structures distributed in 3 categories (absent, early and advanced ossification) (DOCX) [file pone.0126928.s019.docx]

Table S12

A

|  |  |  |  | Score of ossification (Y) | | X² Pearson | | | Logistic regression | | |
| --- | --- | --- | --- | --- | --- | --- | --- | --- | --- | --- | --- |
| Structures | Treat | N | Mean | early | advanced | | p-value | OR (IC 95%) | | p-value | global p-value |
| branchiostegal ray1 down | 3g | 33 | 0.97 | 1 (3.03%) | 32 96.97%) | | **<0.001** | 2.783 (0.238-32.556) | | 0.415 | **0.039** |
|  | 3g > 1g | 25 | 0.68 | 8 (32.00%) | 17 (68%) | |  | 0.185 (0.035-0.983) | | 0.048 |  |
|  | 3g > axe | 25 | 1.00 | 0 (0%) | 25 (100%) | |  | / | | 0.995 |  |
|  | 1g | 25 | 0.92 | 2 (8.00%) | 23 (92%) | |  | 1.00 | |  |  |
| branchiostegal ray1 up | 3g | 33 | 0.97 | 1 (3.03%) | 32 (97%) | | **0.009** | 2.783 (0.238-32.556) | | 0.415 | 0.147 |
|  | 3g > 1g | 25 | 0.76 | 6 (24.00%) | 19 (76%) | |  | 0.275 (0.050-1.525) | | 0.140 |  |
|  | 3g > axe | 25 | 1.00 | 0 (0%) | 25 (100%) | |  | / | | 0.995 |  |
|  | 1g | 25 | 0.92 | 2 (8.00%) | 23 (92%) | |  | 1.00 | |  |  |
| dentary down | 3g | 33 | 0.73 | 9 (27.27%) | 24 (72.7%) | | **0.001** | 2.461 (0.822-7.370) | | 0.842 | 0.190 |
|  | 3g > 1g | 25 | 1.00 | 0 (0%) | 25 (100%) | |  | / | | 0.107 |  |
|  | 3g > axe | 25 | 0.80 | 5 (20%) | 20 (80%) | |  | 3.692 (1.052-12.957) | | 0.995 |  |
|  | 1g | 25 | 0.52 | 12 (48%) | 13 (52%) | |  | 1.00 | |  |  |
| dentary up | 3g | 33 | 0.73 | 9 (27.27%) | 24 (72.7%) | | **0.001** | 2.461 (0.822-7.370) | | 0.842 | 0.190 |
|  | 3g > 1g | 25 | 1.00 | 0 (0%) | 25 (100%) | |  | / | | 0.107 |  |
|  | 3g > axe | 25 | 0.80 | 5 (20%) | 20 (80%) | |  | 3.692 (1.052-12.957) | | 0.995 |  |
|  | 1g | 25 | 0.52 | 12 (48%) | 13 (52%) | |  | 1.00 | |  |  |
| entopterygoid down | 3g | 33 | 0.88 | 4 (12.12%) | 29 (88%) | | 0.075 | 3.412 (0.892-13.046) | | 0.079 | 0.098 |
|  | 3g > 1g | 25 | 0.72 | 7 (28.00%) | 18 (72%) | |  | 1.210 (0.360-4.065) | | 0.073 |  |
|  | 3g > axe | 25 | 0.92 | 2 (8.00%) | 23 (92%) | |  | 5.412 (1.017-28.791) | | 0.758 |  |
|  | 1g | 25 | 0.76 | 8 (32.00%) | 17 (68%) | |  | 1.00 | |  |  |
| entopterygoid up | 3g | 33 | 0.88 | 4 (12.12%) | 29 (88%) | | 0.226 | 2.819 (0.0722-11.01) | | 0.136 | 0.246 |
|  | 3g > 1g | 25 | 0.72 | 7 (28.00%) | 18 (72%) | |  | 1.000 (0.291-3.437) | | 1.000 |  |
|  | 3g > axe | 25 | 0.88 | 3 (12.00%) | 22 (88%) | |  | 2.852 (0.643-12.642) | | 0.168 |  |
|  | 1g | 25 | 0.80 | 7 (28.00%) | 18 (72%) | |  | 1.00 | |  |  |

B

|  |  |  |  | Score of ossification (Y) | | | X² pearson | Ordinal logistic regression | | |
| --- | --- | --- | --- | --- | --- | --- | --- | --- | --- | --- |
| Structures | Variable | N | Mean | absence | early | advanced | p-value | OR (IC 95%) | p-value | global p-value |
| anguloarticular down | 3g | 33 | 1.12 | 11 (33.33%) | 7 (21.21%) | 15 (45.45%) | **0.005** | 0.50 (0.19-1.35) | 0.171 | **0.045** |
|  | 3g > 1g | 25 | 1.24 | 8 (32%) | 3 (12%) | 14 (56%) |  | 0.38 (0.13-1.09) | 0.072 |  |
|  | 3g > axe | 25 | 1.44 | 7(28%) | 0 (0%) | 18 (72%) |  | 0.21 (0.07-0.63) | 0.006 |  |
|  | 1g | 25 | 0.76 | 11 (44%) | 9 (36%) | 5 (20%) |  | 1.00 |  |  |
| anguloarticular up | 3g | 33 | 1.09 | 12 (36.36%) | 6 (18.18%) | 15 (45.45%) | **0.008** | 0.49 (0.18-1.33) | 0.164 | **0.035** |
|  | 3g > 1g | 25 | 1.24 | 8 (32%) | 3 (12%) | 14 (56%) |  | 0.35 (0.12-1.03) | 0.057 |  |
|  | 3g > axe | 25 | 1.44 | 7 (28%) | 0 (0%) | 18 (72%) |  | 0.19 (0.06-0.60) | 0.004 |  |
|  | 1g | 25 | 0.72 | 12 (48%) | 8 (32%) | 5 (20%) |  | 1.00 |  |  |
| branchiostegal ray2 down | 3g | 33 | 0.21 | 26 (78.79%) | 7 (21.21%) | 0 (0%) | 0.169 | 0.93 (0.26-3.38) | 0.912 | 0.405 |
|  | 3g > 1g | 25 | 0.08 | 24 (96%) | 0 (0%) | 1 (4%) |  | 5.68 (0.63-51.07) | 0.121 |  |
|  | 3g > axe | 25 | 0.2 | 20(80%) | 5 (20%) | 0 (0%) |  | 1.00 (0.25-4.01) | 1.000 |  |
|  | 1g | 25 | 0.2 | 20(80%) | 5 (20%) | 0 (0%) |  | 1.00 |  |  |
| branchiostegal ray2 up | 3g | 33 | 0.24 | 26 (76.47%) | 8 (23.53%) | 0 (0%) | 0.247 | 0.99 (0.29-3.34) | 0.983 | 0.432 |
|  | 3g > 1g | 25 | 0.12 | 23 (92%) | 1(4%) | 1(4%) |  | 3.43 (0.63-18.64) | 0.153 |  |
|  | 3g > axe | 25 | 0.16 | 21 (84%) | 4 (16%) | 0 (0%) |  | 1.65 (0.40-6.76) | 0.489 |  |
|  | 1g | 25 | 0.24 | 19 (76%) | 6 (24%) | 0 (0%) |  | 1.00 |  |  |
| ceratohyal down | 3g | 33 | 0.57 | 20 (60.61%) | 7 (21.21%) | 6 (18.18%) | **0.003** | 0.74 (0.25-2.18) | 0.587 | 0.078 |
|  | 3g > 1g | 25 | 1 | 12 (48%) | 1 (4%) | 12 (48%) |  | 0.30 (0.10-0.91) | 0.033 |  |
|  | 3g > axe | 25 | 0.48 | 18 (72%) | 2 (8%) | 5 (20%) |  | 1.08 (0.33-3.55) | 0.894 |  |
|  | 1g | 25 | 0.57 | 16 (64%) | 8 (32%) | 1 (4%) |  | 1.00 |  |  |
| ceratohyal up | 3g | 33 | 0.67 | 18 (54.54%) | 8 (24.24%) | 7 (21.21%) | **0.011** | 0.52 (0.18-1.53) | 0.236 | 0.082 |
|  | 3g > 1g | 25 | 1.04 | 11 (44%) | 2 (8%) | 12 (48%) |  | 0.24 (0.08-074) | 0.013 |  |
|  | 3g > axe | 25 | 0.64 | 16 (64%) | 2 (8%) | 7 (28%) |  | 0.62 (019-1.97) | 0.416 |  |
|  | 1g | 25 | 0.36 | 17 (68%) | 7 (28%) | 1(4%) |  | 1.00 |  |  |
| hyomandibular down | 3g | 33 | 1.88 | 0 (0%) | 4 (12.12%) | 29 (87.88%) | 0.080 | 0.21 (0.06-0.78) | 0.020 | 0.083 |
|  | 3g > 1g | 25 | 1.52 | 4 (16%) | 4 (16%) | 17 (68%) |  | 0.83 (0.27-2.57) | 0.751 |  |
|  | 3g > axe | 25 | 1.52 | 2 (8%) | 8 (32%) | 15 (60%) |  | 1.00 (0.33-3.03) | 1.000 |  |
|  | 1g | 25 | 1.52 | 2 (8%) | 8 (32%) | 15 (60%) |  | 1.00 |  |  |
| hyomandibular up | 3g | 33 | 1.91 | 0 (0%) | 3 (9.09%) | 30 (90.91%) | 0.174 | 0.18 (0.04-0.76) | 0.020 | 0.140 |
|  | 3g > 1g | 25 | 1.64 | 3 (12%) | 3 (12%) | 19 (76%) |  | 0.63 (0.19-2.09) | 0.448 |  |
|  | 3g > axe | 25 | 1.72 | 1(4%) | 5 (20%) | 19 (76%) |  | 0.56 (0.17-1.91) | 0.358 |  |
|  | 1g | 25 | 1.56 | 2 (8%) | 7 (28%) | 16 (64%) |  | 1.00 |  |  |
| maxilla down | 3g | 33 | 1.64 | 3 (9.09%) | 6 (18.18%) | 24 (72.73%) | **0.005** | 0.24 (0.08-0.69) | 0.008 | **0.001** |
|  | 3g > 1g | 25 | 1.76 | 2 (8%) | 2 (8%) | 21 (84%) |  | 0.13 (0.03-0.46) | 0.002 |  |
|  | 3g > axe | 25 | 1.8 | 1 (4%) | 3 (12%) | 21 (84%) |  | 0.12 (0.03-0.44) | 0.001 |  |
|  | 1g | 25 | 1.12 | 6 (24%) | 10 (40%) | 9 (36%) |  | 1.00 |  |  |
| maxilla up | 3g | 33 | 1.57 | 5 (15.15%) | 4 (12.12%) | 24 (72.73%) | **0.001** | 0.29 (0.10-0.82) | 0.019 | **0.003** |
|  | 3g > 1g | 25 | 1.84 | 2 (8%) | 0 (0%) | 23 (92%) |  | 0.07 (0.01-0.33) | 0.001 |  |
|  | 3g > axe | 25 | 1.68 | 2 (8%) | 4 (16%) | 19 (76%) |  | 0.22 (0.07-0.72) | 0.012 |  |
|  | 1g | 25 | 1.12 | 6 (24%) | 10 (40%) | 9 (36%) |  | 1.00 |  |  |
