# Supplementary material for: Zebrafish Bone and General Physiology Are Differently Affected by Hormones or Changes in Gravity
Source: PLoS One. 2015 Jun 10;10(6):e0126928. doi: 10.1371/journal.pone.0126928 (PMC4465622; doi:10.1371/journal.pone.0126928)
Supplement: S13 Table — The indicates the human homolog of the gene, its "Entrez" gene name, the log ratio of (1g) larvae compared to larvae kept at 3g between 0 and 6dpf, the presence of duplicate probes on the microarray (D) and the type of protein it encodes. Genes are arranged according to their type and in alphabetical order. (DOCX) [file pone.0126928.s020.docx]

| **Symbol** | **Entrez Gene Name** | **Log Ratio 1g** | **p-value** | **N** | **Type(s)** |
| --- | --- | --- | --- | --- | --- |
| ABCF2 | ATP-binding cassette. sub-family F (GCN20). member 2 | -0.858 | 6.53E-02 |  | transporter |
| ABCG2 | ATP-binding cassette. sub-family G (WHITE). member 2 | 0.362 | 8.25E-02 |  | transporter |
| ANXA6 | annexin A6 | -0.576 | 9.86E-02 |  | ion channel |
| APOA4 | apolipoprotein A-IV | -0.615 | 8.17E-02 |  | transporter |
| ATP1A1 | ATPase. Na+/K+ transporting. alpha 1 polypeptide | 0.607 | 3.80E-02 | D | transporter |
| ATP1A1 | ATPase. Na+/K+ transporting. alpha 1 polypeptide | 0.614 | 6.53E-02 | D | transporter |
| ATP1B2 | ATPase. Na+/K+ transporting. beta 2 polypeptide | -0.257 | 9.21E-02 |  | transporter |
| ATP2B4 | ATPase. Ca++ transporting. plasma membrane 4 | -0.339 | 5.01E-02 |  | transporter |
| ATP5G3 | ATP synthase. H+ transporting. mitochondrial Fo complex. subunit C3 (subunit 9) | -0.185 | 7.86E-02 |  | transporter |
| ATP6V1B2 | ATPase. H+ transporting. lysosomal 56/58kDa. V1 subunit B2 | 0.249 | 9.37E-02 |  | transporter |
| CACNA2D2 | calcium channel. voltage-dependent. alpha 2/delta subunit 2 | 0.308 | 4.67E-02 |  | ion channel |
| CACNG6 | calcium channel. voltage-dependent. gamma subunit 6 | 0.166 | 5.50E-02 |  | ion channel |
| EXOC7 | exocyst complex component 7 | 0.315 | 8.39E-02 |  | transporter |
| GABRG2 | gamma-aminobutyric acid (GABA) A receptor. gamma 2 | 0.295 | 6.32E-02 |  | ion channel |
| HNRNPU | heterogeneous nuclear ribonucleoprotein U (scaffold attachment factor A) | -0.310 | 5.52E-02 |  | transporter |
| HSDL2 | hydroxysteroid dehydrogenase like 2 | -0.302 | 3.17E-02 |  | transporter |
| KCTD12 | potassium channel tetramerization domain containing 12 | -0.105 | 9.84E-02 | D | ion channel |
| KCTD12 | potassium channel tetramerization domain containing 12 | -0.150 | 3.96E-02 | D | ion channel |
| NDUFA10 | NADH dehydrogenase (ubiquinone) 1 alpha subcomplex. 10. 42kDa | -0.382 | 9.76E-02 |  | transporter |
| PDZK1 | PDZ domain containing 1 | 0.779 | 5.18E-02 |  | transporter |
| SEC14L2 | SEC14-like 2 (S. cerevisiae) | 0.268 | 9.62E-02 |  | transporter |
| SLC13A2 | solute carrier family 13 (sodium-dependent dicarboxylate transporter). member 2 | 0.674 | 1.62E-02 |  | transporter |
| SLC14A2 | solute carrier family 14 (urea transporter). member 2 | -0.237 | 9.37E-02 |  | transporter |
| SLC15A1 | solute carrier family 15 (oligopeptide transporter). member 1 | 0.380 | 9.30E-02 |  | transporter |
| SLC16A3 | solute carrier family 16 (monocarboxylate transporter). member 3 | -0.414 | 3.17E-02 | D | transporter |
| SLC16A3 | solute carrier family 16 (monocarboxylate transporter). member 3 | -0.476 | 4.16E-02 | D | transporter |
| SLC1A3 | solute carrier family 1 (glial high affinity glutamate transporter). member 3 | 0.154 | 8.39E-02 |  | transporter |
| SLC22A2 | solute carrier family 22 (organic cation transporter). member 2 | 0.481 | 3.05E-02 | D | transporter |
| SLC22A2 | solute carrier family 22 (organic cation transporter). member 2 | 0.507 | 3.80E-02 | D | transporter |
| SLC22A5 | solute carrier family 22 (organic cation/carnitine transporter). member 5 | 0.174 | 6.83E-02 |  | transporter |
| SLC25A19 | solute carrier family 25 (mitochondrial thiamine pyrophosphate carrier). member 19 | -0.215 | 3.96E-02 |  | transporter |
| SLC35D1 | solute carrier family 35 (UDP-GlcA/UDP-GalNAc transporter). member D1 | -0.147 | 7.04E-02 |  | transporter |
| SLC37A4 | solute carrier family 37 (glucose-6-phosphate transporter). member 4 | -0.353 | 3.19E-02 | D | transporter |
| SLC37A4 | solute carrier family 37 (glucose-6-phosphate transporter). member 4 | -0.365 | 3.80E-02 | D | transporter |
| SLC6A19 | solute carrier family 6 (neutral amino acid transporter). member 19 | 0.629 | 9.86E-02 |  | transporter |
| SNX12 | sorting nexin 12 | -0.205 | 6.83E-02 |  | transporter |
| SNX16 | sorting nexin 16 | 0.444 | 4.09E-02 |  | transporter |
| TAPBP | TAP binding protein (tapasin) | 0.241 | 4.57E-02 |  | transporter |
| TFRC | transferrin receptor | -0.184 | 3.29E-02 |  | transporter |
| TIMM13 | translocase of inner mitochondrial membrane 13 homolog (yeast) | -0.307 | 6.89E-02 |  | transporter |
| TMED3 | transmembrane emp24 protein transport domain containing 3 | -0.193 | 7.04E-02 |  | transporter |
| TMED9 | transmembrane emp24 protein transport domain containing 9 | -0.259 | 4.49E-02 |  | transporter |
| USO1 | USO1 vesicle transport factor | 0.161 | 5.41E-02 |  | transporter |
| VPS33A | vacuolar protein sorting 33 homolog A (S. cerevisiae) | -0.270 | 7.58E-02 |  | transporter |
| ANKS4B | ankyrin repeat and sterile alpha motif domain containing 4B | 0.313 | 9.11E-02 |  | transcription regulator |
| ATF3 | activating transcription factor 3 | -0.319 | 6.83E-02 |  | transcription regulator |
| BTG2 | BTG family. member 2 | -2.106 | 1.04E-02 | D | transcription regulator |
| BTG2 | BTG family. member 2 | -2.024 | 1.35E-02 | D | transcription regulator |
| C1QBP | complement component 1. q subcomponent binding protein | 0.331 | 6.56E-02 |  | transcription regulator |
| CEBPD | CCAAT/enhancer binding protein (C/EBP). delta | -0.193 | 5.92E-02 |  | transcription regulator |
| DLX1 | distal-less homeobox 1 | -0.153 | 9.30E-02 |  | transcription regulator |
| ECD | ecdysoneless homolog (Drosophila) | 0.137 | 5.19E-02 |  | transcription regulator |
| EEF2 | eukaryotic translation elongation factor 2 | 0.180 | 9.76E-02 |  | translation regulator |
| EGR1 | early growth response 1 | -0.388 | 3.99E-02 |  | transcription regulator |
| EGR2 | early growth response 2 | -0.292 | 4.32E-02 |  | transcription regulator |
| EGR3 | early growth response 3 | -0.305 | 6.08E-02 |  | transcription regulator |
| EIF3C | eukaryotic translation initiation factor 3. subunit C | 0.458 | 2.99E-02 |  | translation regulator |
| EIF4A1 | eukaryotic translation initiation factor 4A1 | -0.742 | 2.89E-02 | D | translation regulator |
| EIF4A1 | eukaryotic translation initiation factor 4A1 | -0.599 | 3.19E-02 | D | translation regulator |
| FOS | FBJ murine osteosarcoma viral oncogene homolog | -2.530 | 2.89E-02 |  | transcription regulator |
| FOSB | FBJ murine osteosarcoma viral oncogene homolog B | -2.124 | 9.40E-03 |  | transcription regulator |
| FOXQ1 | forkhead box Q1 | -0.411 | 3.99E-02 | D | transcription regulator |
| FOXQ1 | forkhead box Q1 | -0.577 | 3.05E-02 | D | transcription regulator |
| GTF2H4 | general transcription factor IIH. polypeptide 4. 52kDa | -0.181 | 5.08E-02 |  | transcription regulator |
| HLTF | helicase-like transcription factor | 0.234 | 6.53E-02 |  | transcription regulator |
| HSF2 | heat shock transcription factor 2 | 0.641 | 5.52E-02 | D | transcription regulator |
| HSF2 | heat shock transcription factor 2 | 0.624 | 6.53E-02 | D | transcription regulator |
| KLF11 | Kruppel-like factor 11 | -0.386 | 3.05E-02 | D | transcription regulator |
| KLF11 | Kruppel-like factor 11 | -0.518 | 6.30E-02 | D | transcription regulator |
| L3MBTL2 | l(3)mbt-like 2 (Drosophila) | -0.173 | 8.96E-02 |  | transcription regulator |
| MED27 | mediator complex subunit 27 | -0.194 | 6.15E-02 |  | transcription regulator |
| MYBL2 | v-myb avian myeloblastosis viral oncogene homolog-like 2 | -0.314 | 2.89E-02 |  | transcription regulator |
| MYCL | v-myc avian myelocytomatosis viral oncogene lung carcinoma derived homolog | 0.402 | 6.59E-02 |  | transcription regulator |
| MYOG | myogenin (myogenic factor 4) | -0.359 | 9.26E-02 |  | transcription regulator |
| NFIL3 | nuclear factor. interleukin 3 regulated | -0.370 | 4.69E-02 |  | transcription regulator |
| NFKBIA | nuclear factor of kappa light polypeptide gene enhancer in B-cells inhibitor. alpha | -0.468 | 5.67E-02 |  | transcription regulator |
| NKX2-2 | NK2 homeobox 2 | -0.168 | 9.39E-02 |  | transcription regulator |
| NMI | N-myc (and STAT) interactor | 0.542 | 5.76E-02 |  | transcription regulator |
| NPAS4 | neuronal PAS domain protein 4 | -1.722 | 3.86E-02 |  | transcription regulator |
| OVOL1 | ovo-like zinc finger 1 | -0.217 | 3.17E-02 |  | transcription regulator |
| PDLIM1 | PDZ and LIM domain 1 | 0.235 | 9.37E-02 |  | transcription regulator |
| Pou3f1 | POU domain. class 3. transcription factor 1 | -0.113 | 9.24E-02 |  | transcription regulator |
| PPARG | peroxisome proliferator-activated receptor gamma | 0.286 | 3.96E-02 |  | ligand-dependent nuclear receptor |
| PTGES2 | prostaglandin E synthase 2 | -0.269 | 9.67E-02 |  | transcription regulator |
| RXRG | retinoid X receptor. gamma | 0.201 | 7.17E-02 |  | ligand-dependent nuclear receptor |
| SOX14 | SRY (sex determining region Y)-box 14 | -0.248 | 5.62E-02 |  | transcription regulator |
| SQSTM1 | sequestosome 1 | 0.587 | 4.57E-02 |  | transcription regulator |
| TAF7 | TAF7 RNA polymerase II. TATA box binding protein (TBP)-associated factor. 55kDa | -0.124 | 8.25E-02 |  | transcription regulator |
| TRIP13 | thyroid hormone receptor interactor 13 | -0.240 | 5.52E-02 |  | transcription regulator |
| YWHAB | tyrosine 3-monooxygenase/tryptophan 5-monooxygenase activation protein. beta | -0.295 | 5.01E-02 | D | transcription regulator |
| YWHAB | tyrosine 3-monooxygenase/tryptophan 5-monooxygenase activation protein. beta | -0.230 | 5.87E-02 | D | transcription regulator |
| CXCR4 | chemokine (C-X-C motif) receptor 4 | -0.099 | 7.21E-02 |  | G-protein coupled receptor |
| DRD3 | dopamine receptor D3 | -0.172 | 4.88E-02 |  | G-protein coupled receptor |
| F3 | coagulation factor III (thromboplastin. tissue factor) | -0.352 | 5.52E-02 |  | transmembrane receptor |
| HMMR | hyaluronan-mediated motility receptor (RHAMM) | -0.471 | 9.96E-02 |  | transmembrane receptor |
| IFNAR1 | interferon (alpha. beta and omega) receptor 1 | 0.354 | 5.45E-02 |  | transmembrane receptor |
| OPN1LW | opsin 1 (cone pigments). long-wave-sensitive | -0.484 | 3.19E-02 | D | G-protein coupled receptor |
| OPN1LW | opsin 1 (cone pigments). long-wave-sensitive | -0.866 | 3.17E-02 | D | G-protein coupled receptor |
| TNFRSF19 | tumor necrosis factor receptor superfamily. member 19 | 0.287 | 3.99E-02 |  | transmembrane receptor |
| C19orf10 | chromosome 19 open reading frame 10 | -0.161 | 7.06E-02 |  | cytokine |
| CMTM3 | CKLF-like MARVEL transmembrane domain containing 3 | -0.156 | 9.53E-02 |  | cytokine |
| EDN1 | endothelin 1 | -0.268 | 6.66E-02 |  | cytokine |
| TNFSF10 | tumor necrosis factor (ligand) superfamily. member 10 | 0.263 | 7.96E-02 |  | cytokine |
| GAS6 | growth arrest-specific 6 | 0.220 | 6.31E-02 |  | growth factor |
| NOG | noggin | -0.221 | 3.45E-02 |  | growth factor |
| ACP2 | acid phosphatase 2. lysosomal | 0.343 | 8.19E-02 |  | phosphatase |
| ACVR1 | activin A receptor. type I | -0.415 | 3.96E-02 | D | kinase |
| ACVR1 | activin A receptor. type I | -0.351 | 5.32E-02 | D | kinase |
| AURKA | aurora kinase A | -0.248 | 9.47E-02 |  | kinase |
| AURKB | aurora kinase B | -0.532 | 5.92E-02 |  | kinase |
| BCKDK | branched chain ketoacid dehydrogenase kinase | -0.593 | 3.05E-02 |  | kinase |
| CCNB1 | cyclin B1 | -0.258 | 5.30E-02 |  | kinase |
| CDK2 | cyclin-dependent kinase 2 | -0.298 | 4.38E-02 |  | kinase |
| CDK7 | cyclin-dependent kinase 7 | -0.174 | 6.83E-02 |  | kinase |
| CKB | creatine kinase. brain | 0.231 | 3.80E-02 |  | kinase |
| CLK4 | CDC-like kinase 4 | 0.413 | 5.92E-02 |  | kinase |
| CMPK1 | cytidine monophosphate (UMP-CMP) kinase 1. cytosolic | -0.184 | 5.52E-02 |  | kinase |
| DAK | dihydroxyacetone kinase 2 homolog (S. cerevisiae) | 0.279 | 3.17E-02 |  | kinase |
| DUSP1 | dual specificity phosphatase 1 | -1.383 | 3.05E-02 | D | phosphatase |
| DUSP1 | dual specificity phosphatase 1 | -1.311 | 3.80E-02 | D | phosphatase |
| DUSP2 | dual specificity phosphatase 2 | -0.716 | 2.89E-02 |  | phosphatase |
| DUSP27 | dual specificity phosphatase 27 (putative) | 0.782 | 3.78E-02 |  | phosphatase |
| ILK | integrin-linked kinase | -0.562 | 1.04E-02 | D | kinase |
| ILK | integrin-linked kinase | -0.577 | 1.04E-02 | D | kinase |
| IMPA1 | inositol(myo)-1(or 4)-monophosphatase 1 | -0.279 | 5.17E-02 |  | phosphatase |
| INPP5D | inositol polyphosphate-5-phosphatase. 145kDa | 0.094 | 9.76E-02 |  | phosphatase |
| MKNK2 | MAP kinase interacting serine/threonine kinase 2 | 0.241 | 8.25E-02 |  | kinase |
| MYO3A | myosin IIIA | 0.301 | 5.01E-02 | D | kinase |
| MYO3A | myosin IIIA | 0.325 | 6.81E-02 | D | kinase |
| PFKFB4 | 6-phosphofructo-2-kinase/fructose-2.6-biphosphatase 4 | -0.355 | 6.83E-02 |  | kinase |
| PLK1 | polo-like kinase 1 | -0.492 | 3.94E-02 |  | kinase |
| PPP2R2D | protein phosphatase 2. regulatory subunit B. delta | -0.209 | 8.40E-02 |  | phosphatase |
| PPP2R4 | protein phosphatase 2A activator. regulatory subunit 4 | -0.169 | 7.17E-02 |  | phosphatase |
| PPP4C | protein phosphatase 4. catalytic subunit | -0.354 | 9.40E-02 |  | phosphatase |
| SIK2 | salt-inducible kinase 2 | 0.635 | 4.71E-02 |  | kinase |
| SLK | STE20-like kinase | 0.192 | 7.57E-02 |  | kinase |
| SOCS3 | suppressor of cytokine signaling 3 | -2.498 | 2.89E-02 | D | phosphatase |
| SOCS3 | suppressor of cytokine signaling 3 | -2.432 | 3.05E-02 | D | phosphatase |
| STK39 | serine threonine kinase 39 | -0.295 | 3.80E-02 |  | kinase |
| TEC | tec protein tyrosine kinase | 0.546 | 1.30E-02 |  | kinase |
| UBLCP1 | ubiquitin-like domain containing CTD phosphatase 1 | -0.384 | 5.36E-02 |  | phosphatase |
| VRK1 | vaccinia related kinase 1 | -0.226 | 8.25E-02 |  | kinase |
| ACAA2 | acetyl-CoA acyltransferase 2 | -0.475 | 6.82E-02 |  | enzyme |
| ACE | angiotensin I converting enzyme | 0.511 | 7.42E-02 |  | peptidase |
| ACOX1 | acyl-CoA oxidase 1. palmitoyl | 0.109 | 9.31E-02 |  | enzyme |
| ACTA2 | actin. alpha 2. smooth muscle. aorta | -0.411 | 8.89E-02 |  | other |
| ACTG1 | actin. gamma 1 | -0.309 | 9.37E-02 |  | other |
| ACTR2 | ARP2 actin-related protein 2 homolog (yeast) | -0.134 | 9.26E-02 |  | other |
| AGXT | alanine-glyoxylate aminotransferase | 0.572 | 5.55E-02 |  | enzyme |
| AHSG | alpha-2-HS-glycoprotein | 0.427 | 9.44E-02 |  | other |
| AKAP17A | A kinase (PRKA) anchor protein 17A | 0.258 | 9.73E-02 |  | other |
| AKIRIN1 | akirin 1 | -0.124 | 5.38E-02 |  | enzyme |
| AKR1A1 | aldo-keto reductase family 1. member A1 (aldehyde reductase) | -0.149 | 6.52E-02 |  | enzyme |
| ALDH2 | aldehyde dehydrogenase 2 family (mitochondrial) | -0.401 | 9.11E-02 |  | enzyme |
| ALG8 | ALG8. alpha-1.3-glucosyltransferase | -0.181 | 5.52E-02 |  | enzyme |
| AMD1 | adenosylmethionine decarboxylase 1 | 0.373 | 3.94E-02 | D | enzyme |
| AMD1 | adenosylmethionine decarboxylase 1 | 0.360 | 6.69E-02 | D | enzyme |
| AMY2A | amylase. alpha 2A (pancreatic) | 0.757 | 3.96E-02 | D | enzyme |
| AMY2A | amylase. alpha 2A (pancreatic) | 0.822 | 5.33E-02 | D | enzyme |
| AMY2A | amylase. alpha 2A (pancreatic) | 0.841 | 4.89E-02 | D | enzyme |
| AMY2B | amylase. alpha 2B (pancreatic) | 1.047 | 3.80E-02 |  | enzyme |
| ANLN | anillin. actin binding protein | -0.459 | 7.42E-02 |  | other |
| API5 | apoptosis inhibitor 5 | -0.301 | 3.99E-02 |  | other |
| Arf2 | ADP-ribosylation factor 2 | -0.325 | 8.25E-02 |  | other |
| ARHGDIB | Rho GDP dissociation inhibitor (GDI) beta | -0.252 | 8.55E-02 | D | other |
| ARHGDIB | Rho GDP dissociation inhibitor (GDI) beta | -0.309 | 9.95E-02 | D | other |
| ARHGEF28 | Rho guanine nucleotide exchange factor (GEF) 28 | 0.214 | 8.25E-02 |  | other |
| ARHGEF39 | Rho guanine nucleotide exchange factor (GEF) 39 | -0.199 | 9.37E-02 |  | other |
| ARR3 | arrestin 3. retinal (X-arrestin) | 1.025 | 4.16E-02 |  | other |
| ASL | argininosuccinate lyase | -0.218 | 5.65E-02 |  | enzyme |
| B4GALT1 | UDP-Gal:betaGlcNAc beta 1.4- galactosyltransferase. polypeptide 1 | 0.200 | 6.83E-02 |  | enzyme |
| BCL2L1 | BCL2-like 1 | 0.218 | 7.66E-02 |  | other |
| BCO1 | beta-carotene oxygenase 1 | 0.387 | 3.19E-02 |  | enzyme |
| BIN1 | bridging integrator 1 | 0.183 | 7.97E-02 |  | other |
| BOD1 | biorientation of chromosomes in cell division 1 | -0.164 | 4.69E-02 |  | other |
| BTBD6 | BTB (POZ) domain containing 6 | -0.349 | 6.52E-02 |  | other |
| C10orf54 | chromosome 10 open reading frame 54 | 0.191 | 3.70E-02 |  | other |
| C10orf88 | chromosome 10 open reading frame 88 | 0.108 | 9.16E-02 |  | other |
| C12orf65 | chromosome 12 open reading frame 65 | -0.106 | 7.73E-02 |  | other |
| C1orf106 | chromosome 1 open reading frame 106 | 0.180 | 9.30E-02 |  | other |
| C2orf40 | chromosome 2 open reading frame 40 | -0.281 | 3.96E-02 |  | other |
| CA10 | carbonic anhydrase X | 0.145 | 9.30E-02 |  | enzyme |
| CALU | calumenin | -0.434 | 3.14E-02 |  | other |
| CAMK2N2 | calcium/calmodulin-dependent protein kinase II inhibitor 2 | 0.153 | 7.28E-02 |  | other |
| CAPRIN1 | cell cycle associated protein 1 | 0.335 | 2.89E-02 | D | other |
| CAPRIN1 | cell cycle associated protein 1 | 0.312 | 4.12E-02 | D | other |
| CASQ1 | calsequestrin 1 (fast-twitch. skeletal muscle) | -0.319 | 4.71E-02 |  | other |
| CAV3 | caveolin 3 | -0.217 | 5.62E-02 |  | enzyme |
| CBWD1 | COBW domain containing 1 | -0.190 | 6.31E-02 |  | other |
| CCDC25 | coiled-coil domain containing 25 | -0.221 | 9.39E-02 |  | other |
| CCNA2 | cyclin A2 | -0.493 | 3.94E-02 |  | other |
| CCNB2 | cyclin B2 | -0.250 | 5.41E-02 |  | other |
| CCNE2 | cyclin E2 | -0.203 | 9.37E-02 |  | other |
| CCNF | cyclin F | -0.250 | 3.29E-02 |  | other |
| CD2AP | CD2-associated protein | -0.154 | 4.66E-02 |  | other |
| CD82 | CD82 molecule | -0.237 | 8.80E-02 |  | other |
| CDA | cytidine deaminase | 0.294 | 5.55E-02 |  | enzyme |
| CDC20 | cell division cycle 20 | -0.379 | 3.19E-02 |  | other |
| CDC5L | cell division cycle 5-like | 0.223 | 6.20E-02 |  | other |
| CDC6 | cell division cycle 6 | -0.236 | 5.87E-02 | D | other |
| CDC6 | cell division cycle 6 | -0.167 | 6.29E-02 | D | other |
| CEL | carboxyl ester lipase | 1.208 | 4.99E-02 | D | enzyme |
| CEL | carboxyl ester lipase | 1.219 | 5.32E-02 | D | enzyme |
| CEL | carboxyl ester lipase | 0.998 | 7.78E-02 | D | enzyme |
| CETP | cholesteryl ester transfer protein. plasma | 0.495 | 9.64E-02 |  | enzyme |
| CFB | complement factor B | 0.322 | 3.96E-02 | D | peptidase |
| CFB | complement factor B | 0.160 | 7.90E-02 | D | peptidase |
| CFP | complement factor properdin | -0.313 | 5.32E-02 |  | other |
| CGRRF1 | cell growth regulator with ring finger domain 1 | -0.089 | 9.76E-02 |  | other |
| CHAC2 | ChaC. cation transport regulator homolog 2 (E. coli) | -0.128 | 6.99E-02 |  | other |
| CHCHD4 | coiled-coil-helix-coiled-coil-helix domain containing 4 | -0.275 | 3.17E-02 |  | enzyme |
| CHIA | chitinase. acidic | 0.357 | 3.96E-02 |  | enzyme |
| CHODL | chondrolectin | -0.109 | 8.11E-02 |  | other |
| CHPF | chondroitin polymerizing factor | 0.125 | 7.04E-02 |  | enzyme |
| CKAP2 | cytoskeleton associated protein 2 | -0.478 | 3.80E-02 |  | other |
| CNN3 | calponin 3. acidic | -0.302 | 3.24E-02 |  | other |
| COL4A1 | collagen. type IV. alpha 1 | 0.481 | 5.49E-02 |  | other |
| COL9A3 | collagen. type IX. alpha 3 | -0.534 | 8.51E-02 |  | other |
| COPS8 | COP9 signalosome subunit 8 | -0.122 | 7.51E-02 | D | other |
| COPS8 | COP9 signalosome subunit 8 | -0.125 | 9.37E-02 | D | other |
| CPA1 | carboxypeptidase A1 (pancreatic) | 1.029 | 5.65E-02 | D | peptidase |
| CPA1 | carboxypeptidase A1 (pancreatic) | 0.545 | 6.29E-02 | D | peptidase |
| CPB1 | carboxypeptidase B1 (tissue) | 0.731 | 4.50E-02 |  | peptidase |
| CPT2 | carnitine palmitoyltransferase 2 | -0.372 | 8.55E-02 |  | enzyme |
| CRIP2 | cysteine-rich protein 2 | -0.211 | 7.06E-02 |  | other |
| CRY1 | cryptochrome circadian clock 1 | 0.557 | 3.96E-02 |  | enzyme |
| CRYGN | crystallin. gamma N | -0.456 | 4.86E-02 |  | other |
| CTNNBIP1 | catenin. beta interacting protein 1 | -0.251 | 3.80E-02 | D | other |
| CTNNBIP1 | catenin. beta interacting protein 1 | -0.227 | 8.37E-02 | D | other |
| CTRB2 | chymotrypsinogen B2 | 0.432 | 7.86E-02 |  | peptidase |
| CTSS | cathepsin S | 0.176 | 9.39E-02 |  | peptidase |
| CYB5A | cytochrome b5 type A (microsomal) | 0.150 | 6.33E-02 |  | enzyme |
| Cyb5r3 | cytochrome b5 reductase 3 | -0.140 | 6.83E-02 |  | enzyme |
| CYP24A1 | cytochrome P450. family 24. subfamily A. polypeptide 1 | 1.264 | 3.97E-02 |  | enzyme |
| CYP27A1 | cytochrome P450. family 27. subfamily A. polypeptide 1 | 0.185 | 4.89E-02 |  | enzyme |
| Cyp2ac1 | cytochrome P450. family 2. subfamily ac. polypeptide 1 | 0.385 | 7.04E-02 |  | other |
| CYP2J2 | cytochrome P450. family 2. subfamily J. polypeptide 2 | 0.233 | 5.92E-02 | D | enzyme |
| CYP2J2 | cytochrome P450. family 2. subfamily J. polypeptide 2 | 0.379 | 5.18E-02 | D | enzyme |
| CYP2J2 | cytochrome P450. family 2. subfamily J. polypeptide 2 | 0.418 | 2.89E-02 | D | enzyme |
| CYP2J2 | cytochrome P450. family 2. subfamily J. polypeptide 2 | 0.750 | 3.17E-02 | D | enzyme |
| CYP2J2 | cytochrome P450. family 2. subfamily J. polypeptide 2 | 0.806 | 8.37E-02 | D | enzyme |
| CYP2J2 | cytochrome P450. family 2. subfamily J. polypeptide 2 | 0.282 | 3.45E-02 | D | enzyme |
| CYR61 | cysteine-rich. angiogenic inducer. 61 | -0.797 | 1.35E-02 |  | other |
| DBT | dihydrolipoamide branched chain transacylase E2 | -0.470 | 3.80E-02 |  | enzyme |
| DCAF13 | DDB1 and CUL4 associated factor 13 | -0.331 | 5.65E-02 | D | other |
| DCAF13 | DDB1 and CUL4 associated factor 13 | -0.364 | 6.53E-02 | D | other |
| DCHS1 | dachsous cadherin-related 1 | -0.113 | 9.98E-02 |  | other |
| DCN | decorin | -0.293 | 7.13E-02 |  | other |
| DCUN1D1 | DCN1. defective in cullin neddylation 1. domain containing 1 | -0.169 | 7.87E-02 |  | other |
| DDB2 | damage-specific DNA binding protein 2. 48kDa | 0.300 | 9.57E-02 |  | other |
| DDC | dopa decarboxylase (aromatic L-amino acid decarboxylase) | -0.212 | 6.38E-02 |  | enzyme |
| DDX4 | DEAD (Asp-Glu-Ala-Asp) box polypeptide 4 | 0.286 | 9.37E-02 |  | enzyme |
| DHRS1 | dehydrogenase/reductase (SDR family) member 1 | 0.176 | 9.20E-02 |  | enzyme |
| DLST | dihydrolipoamide S-succinyltransferase (E2 component of 2-oxo-glutarate complex) | 0.180 | 8.66E-02 |  | enzyme |
| DNAJB11 | DnaJ (Hsp40) homolog. subfamily B. member 11 | -0.226 | 4.09E-02 | D | other |
| DNAJB11 | DnaJ (Hsp40) homolog. subfamily B. member 11 | -0.230 | 4.12E-02 | D | other |
| DPP7 | dipeptidyl-peptidase 7 | 0.269 | 7.90E-02 |  | peptidase |
| DSG2 | desmoglein 2 | -0.230 | 5.33E-02 |  | other |
| DSP | desmoplakin | 0.324 | 7.86E-02 |  | other |
| EBAG9 | estrogen receptor binding site associated. antigen. 9 | -0.137 | 5.18E-02 |  | other |
| ECI1 | enoyl-CoA delta isomerase 1 | -0.406 | 7.86E-02 |  | enzyme |
| EEPD1 | endonuclease/exonuclease/phosphatase family domain containing 1 | 0.200 | 8.37E-02 |  | other |
| EIF2B3 | eukaryotic translation initiation factor 2B. subunit 3 gamma. 58kDa | -0.109 | 9.21E-02 |  | other |
| ELAC2 | elaC ribonuclease Z 2 | 0.211 | 7.34E-02 |  | enzyme |
| ELOVL4 | ELOVL fatty acid elongase 4 | -0.512 | 3.96E-02 |  | enzyme |
| ELOVL7 | ELOVL fatty acid elongase 7 | -1.211 | 1.04E-02 | D | enzyme |
| ELOVL7 | ELOVL fatty acid elongase 7 | -1.199 | 1.30E-02 | D | enzyme |
| ELP6 | elongator acetyltransferase complex subunit 6 | -0.127 | 6.39E-02 |  | other |
| EME1 | essential meiotic structure-specific endonuclease 1 | -0.179 | 9.76E-02 |  | other |
| ENDOU | endonuclease. polyU-specific | 0.845 | 3.17E-02 |  | peptidase |
| ERRFI1 | ERBB receptor feedback inhibitor 1 | -0.417 | 5.74E-02 |  | other |
| ETNPPL | ethanolamine-phosphate phospho-lyase | 0.254 | 3.85E-02 |  | enzyme |
| EXOSC2 | exosome component 2 | -0.269 | 3.05E-02 |  | enzyme |
| EXT2 | exostosin glycosyltransferase 2 | 0.162 | 6.69E-02 |  | enzyme |
| FAAH2 | fatty acid amide hydrolase 2 | 0.350 | 6.83E-02 |  | enzyme |
| FAIM | Fas apoptotic inhibitory molecule | -0.119 | 9.16E-02 |  | other |
| FAM212A | family with sequence similarity 212. member A | -0.155 | 8.74E-02 |  | other |
| FARSA | phenylalanyl-tRNA synthetase. alpha subunit | 0.410 | 6.33E-02 |  | enzyme |
| FBL | fibrillarin | -0.287 | 4.38E-02 |  | other |
| FBXO5 | F-box protein 5 | -0.113 | 7.23E-02 |  | enzyme |
| FCGBP | Fc fragment of IgG binding protein | -0.335 | 6.08E-02 |  | other |
| FEN1 | flap structure-specific endonuclease 1 | -0.258 | 6.53E-02 | D | enzyme |
| FEN1 | flap structure-specific endonuclease 1 | -0.323 | 7.73E-02 | D | enzyme |
| FGL2 | fibrinogen-like 2 | 0.172 | 8.29E-02 |  | peptidase |
| FKBP1B | FK506 binding protein 1B. 12.6 kDa | -0.280 | 3.70E-02 |  | enzyme |
| FNIP1 | folliculin interacting protein 1 | 0.278 | 7.17E-02 |  | other |
| G2E3 | G2/M-phase specific E3 ubiquitin protein ligase | -0.256 | 4.95E-02 |  | enzyme |
| G3BP1 | GTPase activating protein (SH3 domain) binding protein 1 | -0.360 | 4.50E-02 |  | enzyme |
| GADD45B | growth arrest and DNA-damage-inducible. beta | -0.636 | 5.76E-02 |  | other |
| GDI2 | GDP dissociation inhibitor 2 | -0.419 | 5.08E-02 |  | other |
| GINS4 | GINS complex subunit 4 (Sld5 homolog) | -0.191 | 6.54E-02 |  | other |
| GOLGA7 | golgin A7 | -0.317 | 3.94E-02 |  | other |
| GPI | glucose-6-phosphate isomerase | -0.303 | 6.91E-02 |  | enzyme |
| GPX7 | glutathione peroxidase 7 | -0.184 | 6.95E-02 |  | enzyme |
| GTPBP4 | GTP binding protein 4 | 0.334 | 7.59E-02 |  | enzyme |
| GYG1 | glycogenin 1 | 0.293 | 6.71E-02 |  | enzyme |
| H1f0 | H1 histone family. member 0 | -0.399 | 4.29E-02 | D | other |
| H1f0 | H1 histone family. member 0 | -0.408 | 6.33E-02 | D | other |
| HADH | hydroxyacyl-CoA dehydrogenase | -0.407 | 8.04E-02 |  | enzyme |
| HAUS4 | HAUS augmin-like complex. subunit 4 | -0.307 | 7.90E-02 |  | other |
| HGD | homogentisate 1.2-dioxygenase | 0.348 | 9.84E-02 |  | enzyme |
| HIBADH | 3-hydroxyisobutyrate dehydrogenase | -0.301 | 3.86E-02 | D | enzyme |
| HIBADH | 3-hydroxyisobutyrate dehydrogenase | -0.338 | 4.57E-02 | D | enzyme |
| HIST2H2BE | histone cluster 2. H2be | -0.321 | 5.52E-02 |  | other |
| HLCS | holocarboxylase synthetase (biotin-(proprionyl-CoA-carboxylase (ATP-hydrolysing)) ligase) | 0.443 | 7.80E-02 |  | enzyme |
| HMBS | hydroxymethylbilane synthase | -0.213 | 6.83E-02 |  | enzyme |
| HMGCL | 3-hydroxymethyl-3-methylglutaryl-CoA lyase | -0.288 | 5.81E-02 |  | enzyme |
| HMGCS1 | 3-hydroxy-3-methylglutaryl-CoA synthase 1 (soluble) | -0.372 | 8.25E-02 |  | enzyme |
| HMGN3 | high mobility group nucleosomal binding domain 3 | -0.444 | 9.30E-02 |  | other |
| HPRT1 | hypoxanthine phosphoribosyltransferase 1 | -0.138 | 7.87E-02 |  | enzyme |
| HSD17B4 | hydroxysteroid (17-beta) dehydrogenase 4 | 0.442 | 7.86E-02 |  | enzyme |
| HSP90AA1 | heat shock protein 90kDa alpha (cytosolic). class A member 1 | 0.382 | 4.50E-02 |  | enzyme |
| HSP90B1 | heat shock protein 90kDa beta (Grp94). member 1 | -0.285 | 6.32E-02 |  | other |
| IARS | isoleucyl-tRNA synthetase | 0.736 | 8.66E-02 |  | enzyme |
| IDH3A | isocitrate dehydrogenase 3 (NAD+) alpha | -0.307 | 4.95E-02 |  | enzyme |
| IGFBP1 | insulin-like growth factor binding protein 1 | 1.006 | 5.52E-02 |  | other |
| IMP4 | IMP4. U3 small nucleolar ribonucleoprotein | -0.154 | 4.71E-02 |  | other |
| IMPDH1 | IMP (inosine 5'-monophosphate) dehydrogenase 1 | 0.262 | 7.10E-02 |  | enzyme |
| ING3 | inhibitor of growth family. member 3 | 0.325 | 3.96E-02 |  | other |
| INSIG1 | insulin induced gene 1 | 0.307 | 4.32E-02 |  | other |
| IRS2 | insulin receptor substrate 2 | 0.266 | 2.89E-02 |  | enzyme |
| KDELC2 | KDEL (Lys-Asp-Glu-Leu) containing 2 | -0.212 | 8.29E-02 |  | other |
| KDM8 | lysine (K)-specific demethylase 8 | -0.206 | 4.86E-02 |  | other |
| KIAA0196 | KIAA0196 | 0.154 | 8.47E-02 |  | other |
| KIAA1524 | KIAA1524 | -0.177 | 6.83E-02 |  | other |
| KIAA1919 | KIAA1919 | 0.203 | 7.18E-02 |  | peptidase |
| KIF23 | kinesin family member 23 | -0.473 | 7.86E-02 |  | other |
| KNTC1 | kinetochore associated 1 | -0.169 | 8.11E-02 |  | other |
| KRT18 | keratin 18 | 0.301 | 8.39E-02 |  | other |
| LECT1 | leukocyte cell derived chemotaxin 1 | -1.097 | 9.40E-03 | D | other |
| LECT1 | leukocyte cell derived chemotaxin 1 | -1.062 | 2.17E-02 | D | other |
| LENG9 | leukocyte receptor cluster (LRC) member 9 | 0.202 | 9.37E-02 |  | other |
| LGSN | lengsin. lens protein with glutamine synthetase domain | -0.475 | 7.21E-02 |  | enzyme |
| LIMCH1 | LIM and calponin homology domains 1 | 0.710 | 3.80E-02 |  | other |
| LMO7 | LIM domain 7 | 0.281 | 3.86E-02 |  | enzyme |
| LOX | lysyl oxidase | -0.117 | 9.11E-02 |  | enzyme |
| LRIT1 | leucine-rich repeat. immunoglobulin-like and transmembrane domains 1 | 0.613 | 3.80E-02 |  | other |
| LRIT3 | leucine-rich repeat. immunoglobulin-like and transmembrane domains 3 | -0.232 | 6.91E-02 |  | other |
| LRRC39 | leucine rich repeat containing 39 | -0.138 | 5.28E-02 |  | other |
| LSM1 | LSM1. U6 small nuclear RNA associated | -0.174 | 8.04E-02 |  | other |
| LXN | latexin | 0.312 | 6.83E-02 |  | other |
| MANF | mesencephalic astrocyte-derived neurotrophic factor | -0.291 | 6.33E-02 |  | other |
| MAPRE1 | microtubule-associated protein. RP/EB family. member 1 | -0.354 | 4.38E-02 | D | other |
| MAPRE1 | microtubule-associated protein. RP/EB family. member 1 | -0.314 | 8.37E-02 | D | other |
| MASP1 | mannan-binding lectin serine peptidase 1 (C4/C2 activating component of Ra-reactive factor) | 0.168 | 7.96E-02 |  | peptidase |
| MATN1 | matrilin 1. cartilage matrix protein | -0.391 | 4.58E-02 |  | other |
| MCM10 | minichromosome maintenance complex component 10 | -0.152 | 5.78E-02 |  | other |
| MCM2 | minichromosome maintenance complex component 2 | -0.163 | 4.38E-02 |  | enzyme |
| MED28 | mediator complex subunit 28 | -0.171 | 9.26E-02 |  | other |
| MFAP2 | microfibrillar-associated protein 2 | -0.287 | 8.11E-02 |  | other |
| MOB3A | MOB kinase activator 3A | -0.119 | 6.53E-02 |  | other |
| MOGAT1 | monoacylglycerol O-acyltransferase 1 | 0.329 | 3.96E-02 |  | enzyme |
| MON1A | MON1 secretory trafficking family member A | -0.298 | 3.05E-02 |  | other |
| MOSPD2 | motile sperm domain containing 2 | 0.201 | 7.51E-02 |  | other |
| MPC2 | mitochondrial pyruvate carrier 2 | -0.326 | 4.50E-02 | D | other |
| MPC2 | mitochondrial pyruvate carrier 2 | -0.230 | 9.76E-02 | D | other |
| MSH2 | mutS homolog 2 | -0.303 | 8.51E-02 |  | enzyme |
| MTFR2 | mitochondrial fission regulator 2 | -0.200 | 7.87E-02 |  | other |
| MYBPH | myosin binding protein H | -0.244 | 4.66E-02 |  | other |
| MYL6 | myosin. light chain 6. alkali. smooth muscle and non-muscle | 0.243 | 7.97E-02 |  | other |
| MYL7 | myosin. light chain 7. regulatory | -0.237 | 5.42E-02 |  | enzyme |
| N6AMT1 | N-6 adenine-specific DNA methyltransferase 1 (putative) | -0.256 | 8.47E-02 |  | enzyme |
| NCAPG | non-SMC condensin I complex. subunit G | -0.275 | 8.11E-02 |  | other |
| NCAPG2 | non-SMC condensin II complex. subunit G2 | -0.153 | 6.33E-02 |  | other |
| NDC1 | NDC1 transmembrane nucleoporin | -0.229 | 6.91E-02 |  | other |
| NDC80 | NDC80 kinetochore complex component | -0.224 | 4.34E-02 |  | other |
| NDOR1 | NADPH dependent diflavin oxidoreductase 1 | -0.131 | 8.89E-02 |  | enzyme |
| NDRG4 | NDRG family member 4 | -0.334 | 9.64E-02 |  | other |
| NELFA | negative elongation factor complex member A | 0.132 | 9.39E-02 |  | other |
| NID1 | nidogen 1 | 0.233 | 3.86E-02 |  | other |
| NKAIN1 | Na+/K+ transporting ATPase interacting 1 | -0.259 | 3.71E-02 |  | other |
| NOS1 | nitric oxide synthase 1 (neuronal) | 0.101 | 8.74E-02 |  | enzyme |
| NPTN | neuroplastin | 0.277 | 9.11E-02 |  | other |
| NRAS | neuroblastoma RAS viral (v-ras) oncogene homolog | -0.195 | 4.32E-02 |  | enzyme |
| NUDT18 | nudix (nucleoside diphosphate linked moiety X)-type motif 18 | 0.182 | 9.96E-02 |  | other |
| NUF2 | NUF2. NDC80 kinetochore complex component | -0.358 | 5.94E-02 |  | other |
| NXN | nucleoredoxin | -0.223 | 3.86E-02 |  | enzyme |
| ORC6 | origin recognition complex. subunit 6 | -0.219 | 6.83E-02 | D | other |
| ORC6 | origin recognition complex. subunit 6 | -0.243 | 7.04E-02 | D | other |
| Otub1 | OTU domain. ubiquitin aldehyde binding 1 | -0.205 | 9.21E-02 |  | enzyme |
| Otud5 | OTU domain containing 5 | 0.197 | 7.06E-02 |  | enzyme |
| PAH | phenylalanine hydroxylase | 0.371 | 5.33E-02 |  | enzyme |
| PAPD5 | PAP associated domain containing 5 | 0.175 | 6.53E-02 |  | enzyme |
| PC | pyruvate carboxylase | -0.226 | 3.05E-02 | D | enzyme |
| PC | pyruvate carboxylase | -0.175 | 8.25E-02 | D | enzyme |
| PDHX | pyruvate dehydrogenase complex. component X | 0.230 | 8.17E-02 |  | enzyme |
| PER3 | period circadian clock 3 | 0.197 | 3.96E-02 |  | other |
| PGM1 | phosphoglucomutase 1 | -0.322 | 4.65E-02 |  | enzyme |
| PGP | phosphoglycolate phosphatase | -0.522 | 5.13E-02 |  | enzyme |
| PGPEP1 | pyroglutamyl-peptidase I | -0.124 | 5.52E-02 |  | peptidase |
| PHF23 | PHD finger protein 23 | -0.151 | 7.04E-02 |  | other |
| PIGQ | phosphatidylinositol glycan anchor biosynthesis. class Q | -0.111 | 8.74E-02 |  | enzyme |
| PLA2G12B | phospholipase A2. group XIIB | 0.153 | 9.85E-02 |  | enzyme |
| PM20D1 | peptidase M20 domain containing 1 | 0.460 | 7.34E-02 |  | peptidase |
| POLR2H | polymerase (RNA) II (DNA directed) polypeptide H | -0.284 | 5.33E-02 |  | enzyme |
| POSTN | periostin. osteoblast specific factor | -0.448 | 4.66E-02 |  | other |
| PPP1R3B | protein phosphatase 1. regulatory subunit 3B | -0.340 | 6.33E-02 |  | other |
| PRC1 | protein regulator of cytokinesis 1 | -0.247 | 6.91E-02 |  | other |
| PRDM11 | PR domain containing 11 | -0.459 | 5.92E-02 |  | other |
| PRIM1 | primase. DNA. polypeptide 1 (49kDa) | -0.185 | 4.32E-02 |  | enzyme |
| PRMT6 | protein arginine methyltransferase 6 | -0.242 | 4.32E-02 |  | enzyme |
| PRPF31 | pre-mRNA processing factor 31 | 0.118 | 7.42E-02 |  | other |
| PRPH | peripherin | 0.293 | 7.43E-02 |  | other |
| PRRC1 | proline-rich coiled-coil 1 | -0.230 | 5.92E-02 |  | other |
| PSMC6 | proteasome (prosome. macropain) 26S subunit. ATPase. 6 | -0.222 | 9.76E-02 |  | peptidase |
| PSMD11 | proteasome (prosome. macropain) 26S subunit. non-ATPase. 11 | -0.154 | 8.33E-02 |  | other |
| PSME3 | proteasome (prosome. macropain) activator subunit 3 (PA28 gamma; Ki) | -0.354 | 3.29E-02 |  | peptidase |
| PXMP2 | peroxisomal membrane protein 2. 22kDa | -0.147 | 9.38E-02 |  | other |
| PYGM | phosphorylase. glycogen. muscle | -0.265 | 4.29E-02 |  | enzyme |
| RAB1A | RAB1A. member RAS oncogene family | -0.421 | 4.00E-02 | D | enzyme |
| RAB1A | RAB1A. member RAS oncogene family | -0.306 | 6.81E-02 | D | enzyme |
| RAC1 | ras-related C3 botulinum toxin substrate 1 (rho family. small GTP binding protein Rac1) | -0.334 | 4.66E-02 |  | enzyme |
| RAE1 | ribonucleic acid export 1 | -0.131 | 5.25E-02 | D | other |
| RAE1 | ribonucleic acid export 1 | -0.156 | 7.10E-02 | D | other |
| RALA | v-ral simian leukemia viral oncogene homolog A (ras related) | -0.226 | 8.29E-02 |  | enzyme |
| RCC1 | regulator of chromosome condensation 1 | -0.449 | 6.83E-02 |  | other |
| RDH13 | retinol dehydrogenase 13 (all-trans/9-cis) | 0.195 | 9.57E-02 |  | enzyme |
| RGD1563307 | similar to Set beta isoform | -0.302 | 4.88E-02 | D | other |
| RGD1563307 | similar to Set beta isoform | -0.197 | 7.46E-02 | D | other |
| RGS1 | regulator of G-protein signaling 1 | -0.192 | 9.26E-02 |  | other |
| RHOQ | ras homolog family member Q | -0.210 | 9.37E-02 |  | enzyme |
| RMI2 | RecQ mediated genome instability 2 | -0.156 | 9.28E-02 |  | other |
| RNASET2 | ribonuclease T2 | -0.369 | 7.80E-02 |  | enzyme |
| RNF8 | ring finger protein 8. E3 ubiquitin protein ligase | 0.172 | 6.37E-02 |  | enzyme |
| RNFT1 | ring finger protein. transmembrane 1 | -0.271 | 9.86E-02 |  | other |
| RNLS | renalase. FAD-dependent amine oxidase | -0.519 | 8.29E-02 |  | other |
| RPL31 | ribosomal protein L31 | 0.236 | 5.52E-02 |  | other |
| RPRM | reprimo. TP53 dependent G2 arrest mediator candidate | -0.207 | 3.80E-02 |  | other |
| RPUSD2 | RNA pseudouridylate synthase domain containing 2 | -0.172 | 8.34E-02 |  | enzyme |
| RRM1 | ribonucleotide reductase M1 | -0.398 | 4.81E-02 |  | enzyme |
| RTCA | RNA 3'-terminal phosphate cyclase | -0.394 | 8.51E-02 |  | enzyme |
| SAE1 | SUMO1 activating enzyme subunit 1 | -0.331 | 7.22E-02 |  | enzyme |
| SAMM50 | SAMM50 sorting and assembly machinery component | -0.186 | 5.01E-02 | D | other |
| SAMM50 | SAMM50 sorting and assembly machinery component | -0.285 | 7.87E-02 | D | other |
| SCCPDH | saccharopine dehydrogenase (putative) | 0.312 | 5.92E-02 |  | other |
| SDAD1 | SDA1 domain containing 1 | 0.250 | 8.39E-02 |  | other |
| SEPHS1 | selenophosphate synthetase 1 | -0.283 | 4.50E-02 |  | enzyme |
| SEPN1 | selenoprotein N. 1 | -0.394 | 5.91E-02 |  | other |
| SERHL2 | serine hydrolase-like 2 | -0.181 | 8.80E-02 |  | enzyme |
| SERPINB6 | serpin peptidase inhibitor. clade B (ovalbumin). member 6 | 0.408 | 3.94E-02 | D | other |
| SERPINB6 | serpin peptidase inhibitor. clade B (ovalbumin). member 6 | 0.397 | 6.53E-02 | D | other |
| SGPL1 | sphingosine-1-phosphate lyase 1 | 0.324 | 9.26E-02 |  | enzyme |
| Sh3bgr | SH3-binding domain glutamic acid-rich protein | -0.248 | 3.71E-02 |  | other |
| SH3BGRL | SH3 domain binding glutamate-rich protein like | -0.207 | 3.91E-02 |  | other |
| SIAE | sialic acid acetylesterase | -0.140 | 7.04E-02 |  | enzyme |
| SLC25A47 | solute carrier family 25. member 47 | 0.133 | 9.30E-02 |  | other |
| SLMO2 | slowmo homolog 2 (Drosophila) | -0.479 | 3.17E-02 | D | other |
| SLMO2 | slowmo homolog 2 (Drosophila) | -0.495 | 7.87E-02 | D | other |
| SMARCAD1 | SWI/SNF-related. matrix-associated actin-dependent regulator of chromatin. subfamily a. containing DEAD/H box 1 | -0.414 | 6.56E-02 |  | enzyme |
| SMPDL3B | sphingomyelin phosphodiesterase. acid-like 3B | 0.280 | 9.11E-02 |  | enzyme |
| SNRPA | small nuclear ribonucleoprotein polypeptide A | -0.213 | 8.74E-02 |  | other |
| SNRPB2 | small nuclear ribonucleoprotein polypeptide B | -0.220 | 3.17E-02 |  | other |
| SPCS2 | signal peptidase complex subunit 2 homolog (S. cerevisiae) | -0.519 | 5.08E-02 |  | other |
| SPDL1 | spindle apparatus coiled-coil protein 1 | -0.360 | 4.66E-02 |  | other |
| SRSF1 | serine/arginine-rich splicing factor 1 | -0.424 | 5.92E-02 | D | other |
| SRSF1 | serine/arginine-rich splicing factor 1 | -0.368 | 9.28E-02 | D | other |
| SRSF9 | serine/arginine-rich splicing factor 9 | -0.195 | 9.20E-02 |  | enzyme |
| SSB | Sjogren syndrome antigen B (autoantigen La) | -0.334 | 9.76E-02 |  | enzyme |
| ST7 | suppression of tumorigenicity 7 | 0.268 | 2.89E-02 |  | other |
| STARD10 | StAR-related lipid transfer (START) domain containing 10 | 0.159 | 4.98E-02 |  | other |
| STMN2 | stathmin 2 | -0.235 | 3.14E-02 | D | other |
| STMN2 | stathmin 2 | -0.195 | 4.12E-02 | D | other |
| STMN2 | stathmin 2 | -0.253 | 5.92E-02 | D | other |
| STMN2 | stathmin 2 | -0.161 | 6.02E-02 | D | other |
| STMN2 | stathmin 2 | -0.180 | 6.33E-02 | D | other |
| STMN2 | stathmin 2 | -0.202 | 7.90E-02 | D | other |
| SULT1A1 | sulfotransferase family. cytosolic. 1A. phenol-preferring. member 1 | 0.222 | 4.65E-02 | D | enzyme |
| SULT1A1 | sulfotransferase family. cytosolic. 1A. phenol-preferring. member 1 | 0.226 | 5.41E-02 | D | enzyme |
| SUMO3 | small ubiquitin-like modifier 3 | -0.376 | 7.97E-02 |  | other |
| SYBU | syntabulin (syntaxin-interacting) | 0.582 | 4.57E-02 |  | other |
| TACC3 | transforming. acidic coiled-coil containing protein 3 | -0.500 | 5.67E-02 |  | other |
| TAT | tyrosine aminotransferase | 1.037 | 3.94E-02 |  | enzyme |
| TCTA | T-cell leukemia translocation altered | -0.229 | 9.67E-02 |  | other |
| TDG | thymine-DNA glycosylase | -0.372 | 9.83E-02 |  | enzyme |
| TDO2 | tryptophan 2.3-dioxygenase | 0.538 | 6.33E-02 |  | enzyme |
| TGFBI | transforming growth factor. beta-induced. 68kDa | -0.411 | 3.05E-02 |  | other |
| TGM1 | transglutaminase 1 | -0.568 | 7.56E-02 |  | enzyme |
| THOC6 | THO complex 6 homolog (Drosophila) | -0.197 | 3.94E-02 |  | other |
| THOP1 | thimet oligopeptidase 1 | -0.174 | 9.37E-02 |  | peptidase |
| THYN1 | thymocyte nuclear protein 1 | -0.311 | 8.15E-02 |  | other |
| TIAM1 | T-cell lymphoma invasion and metastasis 1 | 0.150 | 5.65E-02 |  | other |
| TMEM189 | transmembrane protein 189 | -0.266 | 2.99E-02 |  | other |
| TMEM254 | transmembrane protein 254 | 0.627 | 3.94E-02 | D | other |
| TMEM254 | transmembrane protein 254 | 0.638 | 5.58E-02 | D | other |
| TMIGD1 | transmembrane and immunoglobulin domain containing 1 | 0.365 | 3.29E-02 |  | other |
| TMPRSS13 | transmembrane protease. serine 13 | 0.277 | 7.42E-02 |  | peptidase |
| TNKS | tankyrase. TRF1-interacting ankyrin-related ADP-ribose polymerase | 0.151 | 7.09E-02 |  | enzyme |
| TNR | tenascin R | 0.221 | 5.01E-02 | D | other |
| TNR | tenascin R | 0.191 | 7.42E-02 | D | other |
| TP53BP2 | tumor protein p53 binding protein 2 | 0.365 | 6.89E-02 |  | other |
| TRAM1 | translocation associated membrane protein 1 | 0.319 | 5.41E-02 |  | other |
| TRMT13 | tRNA methyltransferase 13 homolog (S. cerevisiae) | -0.139 | 6.38E-02 |  | other |
| TRMT61A | tRNA methyltransferase 61 homolog A (S. cerevisiae) | -0.149 | 8.37E-02 |  | enzyme |
| TSPAN4 | tetraspanin 4 | 0.635 | 3.94E-02 |  | other |
| TSPAN6 | tetraspanin 6 | -0.452 | 6.20E-02 |  | other |
| TTC5 | tetratricopeptide repeat domain 5 | -0.122 | 6.69E-02 |  | other |
| TUBA1C | tubulin. alpha 1c | -0.329 | 8.29E-02 | D | other |
| TUBA1C | tubulin. alpha 1c | -0.307 | 8.98E-02 | D | other |
| TUBA8 | tubulin. alpha 8 | -0.715 | 5.91E-02 |  | other |
| TUBB4B | tubulin. beta 4B class IVb | -0.301 | 2.89E-02 |  | other |
| TXNDC12 | thioredoxin domain containing 12 (endoplasmic reticulum) | -0.459 | 6.83E-02 |  | enzyme |
| TYRP1 | tyrosinase-related protein 1 | -0.276 | 7.04E-02 |  | enzyme |
| UCHL1 | ubiquitin carboxyl-terminal esterase L1 (ubiquitin thiolesterase) | -0.184 | 3.96E-02 |  | peptidase |
| UFL1 | UFM1-specific ligase 1 | 0.352 | 6.53E-02 |  | other |
| UGCG | UDP-glucose ceramide glucosyltransferase | -0.129 | 8.76E-02 |  | enzyme |
| UGGT2 | UDP-glucose glycoprotein glucosyltransferase 2 | -0.273 | 7.94E-02 |  | enzyme |
| USP13 | ubiquitin specific peptidase 13 (isopeptidase T-3) | 0.290 | 6.53E-02 |  | peptidase |
| USP28 | ubiquitin specific peptidase 28 | 0.426 | 4.15E-02 |  | peptidase |
| USP48 | ubiquitin specific peptidase 48 | 0.268 | 3.96E-02 |  | peptidase |
| UTP11L | UTP11-like. U3 small nucleolar ribonucleoprotein (yeast) | -0.181 | 6.71E-02 |  | other |
| VMP1 | vacuole membrane protein 1 | -0.232 | 4.15E-02 |  | other |
| VWA5A | von Willebrand factor A domain containing 5A | 0.413 | 9.40E-02 |  | other |
| WDR5 | WD repeat domain 5 | -0.335 | 9.16E-02 |  | other |
| WRNIP1 | Werner helicase interacting protein 1 | -0.209 | 9.30E-02 |  | enzyme |
| XPOT | exportin. tRNA | -0.339 | 8.25E-02 |  | other |
| YAE1D1 | Yae1 domain containing 1 | -0.336 | 5.40E-02 |  | other |
| YWHAQ | tyrosine 3-monooxygenase/tryptophan 5-monooxygenase activation protein. theta | -0.314 | 7.87E-02 | D | other |
| YWHAQ | tyrosine 3-monooxygenase/tryptophan 5-monooxygenase activation protein. theta | -0.384 | 7.97E-02 | D | other |
| ZBTB11 | zinc finger and BTB domain containing 11 | 0.335 | 3.85E-02 |  | other |
| ZBTB49 | zinc finger and BTB domain containing 49 | 0.234 | 3.29E-02 |  | other |
| ZFYVE27 | zinc finger. FYVE domain containing 27 | -0.134 | 5.38E-02 |  | other |
| ZMYND19 | zinc finger. MYND-type containing 19 | -0.138 | 8.98E-02 |  | other |
| ZNF410 | zinc finger protein 410 | -0.198 | 8.25E-02 |  | other |
| ZNF729 | zinc finger protein 729 | -0.278 | 3.34E-02 | D | other |
| ZNF729 | zinc finger protein 729 | -0.158 | 5.74E-02 | D | other |
| ZNF729 | zinc finger protein 729 | -0.280 | 5.28E-02 | D | other |
| ZNF800 | zinc finger protein 800 | -0.177 | 5.16E-02 | D | other |
| ZNF800 | zinc finger protein 800 | -0.196 | 6.83E-02 | D | other |
