# Supplementary material for: Zebrafish Bone and General Physiology Are Differently Affected by Hormones or Changes in Gravity
Source: PLoS One. 2015 Jun 10;10(6):e0126928. doi: 10.1371/journal.pone.0126928 (PMC4465622; doi:10.1371/journal.pone.0126928)
Supplement: S14 Table — The indicates the human homolog of the gene, its "Entrez" gene name, the log ratio of (3g>axe) larvae compared to larvae kept at 3g between 0 and 6dpf, the presence of duplicate probes on the microarray (D) and the type of protein it encodes. Genes are arranged according to their type and in alphabetical order. (DOCX) [file pone.0126928.s021.docx]

| **Symbol** | **Entrez Gene Name** | **Log Ratio** | **p-value** | **N** | **Type(s)** |
| --- | --- | --- | --- | --- | --- |
| AMBP | alpha-1-microglobulin/bikunin precursor | 0.264 | 4.22E-02 |  | transporter |
| APOA4 | apolipoprotein A-IV | -0.574 | 9.90E-02 |  | transporter |
| ATP1A1 | ATPase. Na+/K+ transporting. alpha 1 polypeptide | -0.270 | 6.58E-02 |  | transporter |
| ATP1B2 | ATPase. Na+/K+ transporting. beta 2 polypeptide | -0.291 | 3.84E-02 |  | transporter |
| ATP5J | ATP synthase. H+ transporting. mitochondrial Fo complex. subunit F6 | 0.179 | 9.90E-02 |  | transporter |
| GLRB | glycine receptor. beta | 0.127 | 9.81E-02 |  | ion channel |
| GOLGA3 | golgin A3 | -0.113 | 9.10E-02 |  | transporter |
| HBE1 | hemoglobin. epsilon 1 | 0.429 | 9.61E-02 |  | transporter |
| NSF | N-ethylmaleimide-sensitive factor | 0.221 | 7.77E-02 |  | transporter |
| RYR2 | ryanodine receptor 2 (cardiac) | -0.218 | 9.04E-02 |  | ion channel |
| SLC25A24 | solute carrier family 25 (mitochondrial carrier; phosphate carrier). member 24 | 0.148 | 6.58E-02 |  | transporter |
| SNX15 | sorting nexin 15 | 0.098 | 9.81E-02 |  | transporter |
| STX6 | syntaxin 6 | -0.266 | 9.32E-02 |  | transporter |
| VPS33A | vacuolar protein sorting 33 homolog A (S. cerevisiae) | 0.257 | 3.84E-02 |  | transporter |
| BTG2 | BTG family. member 2 | -1.337 | 3.30E-02 | D | transcription regulator |
| BTG2 | BTG family. member 2 | -1.504 | 4.80E-02 | D | transcription regulator |
| CSHL1 | chorionic somatomammotropin hormone-like 1 | -0.434 | 5.73E-02 |  | transcription regulator |
| EGR1 | early growth response 1 | -0.319 | 9.90E-02 |  | transcription regulator |
| FOS | FBJ murine osteosarcoma viral oncogene homolog | -2.020 | 9.81E-02 |  | transcription regulator |
| FOSB | FBJ murine osteosarcoma viral oncogene homolog B | -1.985 | 8.82E-02 |  | transcription regulator |
| FOXM1 | forkhead box M1 | 0.268 | 8.62E-02 |  | transcription regulator |
| FOXQ1 | forkhead box Q1 | -0.719 | 3.37E-02 |  | transcription regulator |
| HDAC4 | histone deacetylase 4 | -0.273 | 4.80E-02 |  | transcription regulator |
| HES1 | hes family bHLH transcription factor 1 | -0.397 | 3.66E-02 |  | transcription regulator |
| KLF2 | Kruppel-like factor 2 | -0.758 | 3.30E-02 |  | transcription regulator |
| KLF2 | Kruppel-like factor 2 | -0.679 | 3.83E-02 |  | transcription regulator |
| LHX1 | LIM homeobox 1 | -0.173 | 6.86E-02 |  | transcription regulator |
| MSX2 | msh homeobox 2 | -0.310 | 3.84E-02 |  | transcription regulator |
| NPAT | nuclear protein. ataxia-telangiectasia locus | 0.233 | 6.58E-02 |  | transcription regulator |
| ONECUT1 | one cut homeobox 1 | -0.133 | 6.86E-02 | D | transcription regulator |
| ONECUT1 | one cut homeobox 1 | -0.126 | 9.17E-02 | D | transcription regulator |
| PAX9 | paired box 9 | -0.226 | 5.32E-02 |  | transcription regulator |
| PPARG | peroxisome proliferator-activated receptor gamma | 0.339 | 9.90E-02 |  | ligand-dependent nuclear receptor |
| PRPF6 | pre-mRNA processing factor 6 | 0.130 | 9.39E-02 |  | transcription regulator |
| SOX10 | SRY (sex determining region Y)-box 10 | 0.120 | 9.21E-02 |  | transcription regulator |
| TAF9 | TAF9 RNA polymerase II. TATA box binding protein (TBP)-associated factor. 32kDa | -0.134 | 9.39E-02 |  | transcription regulator |
| ACKR3 | atypical chemokine receptor 3 | -0.201 | 4.65E-02 |  | G-protein coupled receptor |
| B2M | beta-2-microglobulin | -0.184 | 5.32E-02 |  | transmembrane receptor |
| KTN1 | kinectin 1 (kinesin receptor) | 0.186 | 6.86E-02 |  | transmembrane receptor |
| LGALS3BP | lectin. galactoside-binding. soluble. 3 binding protein | 0.169 | 4.80E-02 |  | transmembrane receptor |
| LRPAP1 | low density lipoprotein receptor-related protein associated protein 1 | 0.175 | 6.74E-02 |  | transmembrane receptor |
| OPN1LW | opsin 1 (cone pigments). long-wave-sensitive | 0.198 | 9.55E-02 |  | G-protein coupled receptor |
| TNFRSF14 | tumor necrosis factor receptor superfamily. member 14 | 0.205 | 9.76E-02 |  | transmembrane receptor |
| EDN1 | endothelin 1 | -0.175 | 7.78E-02 | D | cytokine |
| EDN1 | endothelin 1 | -0.184 | 9.47E-02 | D | cytokine |
| AGT | angiotensinogen (serpin peptidase inhibitor. clade A. member 8) | 0.236 | 8.33E-02 |  | growth factor |
| CAMK2G | calcium/calmodulin-dependent protein kinase II gamma | -0.412 | 3.30E-02 |  | kinase |
| CKM | creatine kinase. muscle | -0.279 | 4.22E-02 |  | kinase |
| DLG1 | discs. large homolog 1 (Drosophila) | 0.163 | 9.76E-02 |  | kinase |
| GK5 | glycerol kinase 5 (putative) | 0.155 | 6.86E-02 |  | kinase |
| GNE | glucosamine (UDP-N-acetyl)-2-epimerase/N-acetylmannosamine kinase | 0.187 | 9.39E-02 |  | kinase |
| MAP3K5 | mitogen-activated protein kinase kinase kinase 5 | -0.103 | 9.90E-02 |  | kinase |
| NDRG1 | N-myc downstream regulated 1 | 0.288 | 3.84E-02 |  | kinase |
| PAK4 | p21 protein (Cdc42/Rac)-activated kinase 4 | -0.149 | 9.39E-02 |  | kinase |
| PIM2 | Pim-2 proto-oncogene. serine/threonine kinase | -0.451 | 9.61E-02 |  | kinase |
| PTPN13 | protein tyrosine phosphatase. non-receptor type 13 (APO-1/CD95 (Fas)-associated phosphatase) | 0.174 | 6.67E-02 |  | phosphatase |
| SOCS3 | suppressor of cytokine signaling 3 | -2.038 | 8.50E-02 |  | phosphatase |
| TWF2 | twinfilin actin-binding protein 2 | -0.188 | 8.44E-02 |  | kinase |
| 2610028H24Rik | RIKEN cDNA 2610028H24 gene | -0.098 | 9.39E-02 |  | other |
| ACAA1 | acetyl-CoA acyltransferase 1 | 0.180 | 9.39E-02 |  | enzyme |
| ACHE | acetylcholinesterase (Yt blood group) | -0.269 | 9.39E-02 |  | enzyme |
| ACO1 | aconitase 1. soluble | -0.405 | 4.38E-02 |  | enzyme |
| ACTR10 | actin-related protein 10 homolog (S. cerevisiae) | 0.164 | 6.86E-02 |  | other |
| AKTIP | AKT interacting protein | -0.098 | 9.61E-02 |  | other |
| AMY2B | amylase. alpha 2B (pancreatic) | 0.262 | 4.78E-02 |  | enzyme |
| ANKRD9 | ankyrin repeat domain 9 | -0.290 | 8.47E-02 |  | other |
| ARRDC3 | arrestin domain containing 3 | 0.243 | 9.47E-02 |  | other |
| ASL | argininosuccinate lyase | -0.176 | 4.80E-02 |  | enzyme |
| ATL3 | atlastin GTPase 3 | -0.194 | 9.39E-02 |  | other |
| BABAM1 | BRISC and BRCA1 A complex member 1 | 0.184 | 5.29E-02 |  | other |
| BCO1 | beta-carotene oxygenase 1 | 0.189 | 8.01E-02 |  | enzyme |
| BIN3 | bridging integrator 3 | -0.238 | 9.39E-02 |  | other |
| BYSL | bystin-like | -0.147 | 9.39E-02 |  | other |
| C14orf166 | chromosome 14 open reading frame 166 | 0.119 | 9.44E-02 |  | other |
| CA13 | carbonic anhydrase XIII | 0.174 | 9.39E-02 |  | enzyme |
| CAP2 | CAP. adenylate cyclase-associated protein. 2 (yeast) | -0.223 | 9.47E-02 |  | other |
| CAPZA1 | capping protein (actin filament) muscle Z-line. alpha 1 | 0.227 | 9.81E-02 |  | other |
| CASP6 | caspase 6. apoptosis-related cysteine peptidase | -0.377 | 3.84E-02 |  | peptidase |
| CCDC93 | coiled-coil domain containing 93 | 0.201 | 8.00E-02 |  | other |
| CD2BP2 | CD2 (cytoplasmic tail) binding protein 2 | 0.313 | 5.39E-02 |  | other |
| CDC6 | cell division cycle 6 | 0.149 | 9.39E-02 |  | other |
| CKAP2 | cytoskeleton associated protein 2 | 0.363 | 9.39E-02 |  | other |
| CMTR1 | cap methyltransferase 1 | 0.181 | 6.32E-02 |  | enzyme |
| CRBN | cereblon | 0.140 | 6.86E-02 |  | enzyme |
| CRYL1 | crystallin. lambda 1 | 0.338 | 7.15E-02 |  | enzyme |
| CWC25 | CWC25 spliceosome-associated protein homolog (S. cerevisiae) | 0.175 | 6.32E-02 |  | other |
| CYP26C1 | cytochrome P450. family 26. subfamily C. polypeptide 1 | -0.236 | 9.81E-02 |  | enzyme |
| CYP2J2 | cytochrome P450. family 2. subfamily J. polypeptide 2 | -0.231 | 8.62E-02 |  | enzyme |
| DBR1 | debranching RNA lariats 1 | 0.142 | 7.63E-02 |  | enzyme |
| DDX24 | DEAD (Asp-Glu-Ala-Asp) box helicase 24 | -0.255 | 3.84E-02 |  | enzyme |
| DNAJA3 | DnaJ (Hsp40) homolog. subfamily A. member 3 | -0.330 | 9.53E-02 |  | other |
| EFCAB14 | EF-hand calcium binding domain 14 | 0.414 | 3.97E-02 |  | other |
| EIF2B3 | eukaryotic translation initiation factor 2B. subunit 3 gamma. 58kDa | -0.161 | 8.44E-02 |  | other |
| ELAVL4 | ELAV like neuron-specific RNA binding protein 4 | -0.384 | 9.32E-02 |  | other |
| ELOVL7 | ELOVL fatty acid elongase 7 | -1.122 | 6.86E-02 | D | enzyme |
| ELOVL7 | ELOVL fatty acid elongase 7 | -1.130 | 8.00E-02 | D | enzyme |
| ERGIC1 | endoplasmic reticulum-golgi intermediate compartment (ERGIC) 1 | 0.347 | 4.65E-02 |  | other |
| ERI1 | exoribonuclease 1 | 0.165 | 8.82E-02 |  | enzyme |
| ESF1 | ESF1. nucleolar pre-rRNA processing protein. homolog (S. cerevisiae) | -0.274 | 6.37E-02 | D | other |
| ESF1 | ESF1. nucleolar pre-rRNA processing protein. homolog (S. cerevisiae) | -0.213 | 6.39E-02 | D | other |
| ESF1 | ESF1. nucleolar pre-rRNA processing protein. homolog (S. cerevisiae) | -0.239 | 6.37E-02 | D | other |
| FAM195A | family with sequence similarity 195. member A | -0.179 | 5.32E-02 |  | other |
| FBXL3 | F-box and leucine-rich repeat protein 3 | -0.157 | 9.39E-02 |  | enzyme |
| FBXW11 | F-box and WD repeat domain containing 11 | 0.109 | 9.39E-02 |  | enzyme |
| FKBP5 | FK506 binding protein 5 | 0.516 | 3.30E-02 |  | enzyme |
| GADD45B | growth arrest and DNA-damage-inducible. beta | -0.313 | 9.76E-02 |  | other |
| GRHL3 | grainyhead-like 3 (Drosophila) | -0.152 | 7.15E-02 |  | other |
| HES5 | hes family bHLH transcription factor 5 | -0.191 | 8.44E-02 |  | other |
| HEXA | hexosaminidase A (alpha polypeptide) | -0.174 | 9.32E-02 |  | enzyme |
| HS6ST2 | heparan sulfate 6-O-sulfotransferase 2 | 0.175 | 9.39E-02 |  | enzyme |
| HSP90AA1 | heat shock protein 90kDa alpha (cytosolic). class A member 1 | -0.233 | 9.04E-02 |  | enzyme |
| HSPG2 | heparan sulfate proteoglycan 2 | -0.211 | 9.68E-02 |  | enzyme |
| LETM2 | leucine zipper-EF-hand containing transmembrane protein 2 | -0.155 | 6.58E-02 |  | other |
| LOX | lysyl oxidase | 0.139 | 9.32E-02 |  | enzyme |
| LRIT1 | leucine-rich repeat. immunoglobulin-like and transmembrane domains 1 | -0.257 | 9.88E-02 |  | other |
| LSM12 | LSM12 homolog (S. cerevisiae) | 0.226 | 9.90E-02 |  | other |
| MAB21L3 | mab-21-like 3 (C. elegans) | 0.134 | 6.58E-02 |  | other |
| METAP1D | methionyl aminopeptidase type 1D (mitochondrial) | -0.252 | 9.39E-02 |  | peptidase |
| METRN | meteorin. glial cell differentiation regulator | 0.136 | 8.62E-02 |  | other |
| MGME1 | mitochondrial genome maintenance exonuclease 1 | -0.281 | 6.86E-02 |  | enzyme |
| MLF2 | myeloid leukemia factor 2 | -0.205 | 9.39E-02 |  | other |
| MNAT1 | MNAT CDK-activating kinase assembly factor 1 | -0.139 | 8.00E-02 |  | other |
| MVP | major vault protein | 0.286 | 3.84E-02 |  | other |
| NCKIPSD | NCK interacting protein with SH3 domain | -0.242 | 9.76E-02 |  | other |
| NEIL3 | nei endonuclease VIII-like 3 (E. coli) | -0.177 | 6.58E-02 |  | enzyme |
| NIPAL4 | NIPA-like domain containing 4 | -0.237 | 9.39E-02 |  | other |
| NPEPL1 | aminopeptidase-like 1 | 0.114 | 9.39E-02 |  | peptidase |
| Nrxn3 | neurexin III | -0.228 | 4.16E-02 |  | other |
| NUP205 | nucleoporin 205kDa | 0.178 | 4.80E-02 |  | other |
| PARP1 | poly (ADP-ribose) polymerase 1 | 0.224 | 8.00E-02 |  | enzyme |
| PC | pyruvate carboxylase | 0.205 | 4.65E-02 |  | enzyme |
| PCDH11X | protocadherin 11 X-linked | -0.208 | 8.62E-02 |  | other |
| PCDHA8 | protocadherin alpha 8 | 0.506 | 6.22E-02 | D | other |
| PCDHA8 | protocadherin alpha 8 | -0.196 | 9.39E-02 | D | other |
| PDCD2L | programmed cell death 2-like | -0.158 | 9.39E-02 |  | other |
| PDCL3 | phosducin-like 3 | -0.270 | 4.22E-02 |  | other |
| PDHA1 | pyruvate dehydrogenase (lipoamide) alpha 1 | -0.224 | 7.77E-02 |  | enzyme |
| PLA2G12B | phospholipase A2. group XIIB | 0.222 | 4.78E-02 |  | enzyme |
| PLA2G15 | phospholipase A2. group XV | 0.221 | 4.78E-02 |  | enzyme |
| PLXDC2 | plexin domain containing 2 | -0.293 | 9.39E-02 |  | other |
| POLR3B | polymerase (RNA) III (DNA directed) polypeptide B | -0.177 | 9.61E-02 |  | enzyme |
| POPDC3 | popeye domain containing 3 | -0.125 | 7.15E-02 |  | other |
| PPP1R37 | protein phosphatase 1. regulatory subunit 37 | 0.149 | 7.78E-02 |  | other |
| PRC1 | protein regulator of cytokinesis 1 | 0.137 | 9.39E-02 |  | other |
| PRPH | peripherin | 0.157 | 8.01E-02 |  | other |
| PSEN1 | presenilin 1 | 0.176 | 6.46E-02 |  | peptidase |
| RAB3C | RAB3C. member RAS oncogene family | 0.276 | 7.14E-02 |  | enzyme |
| RNF130 | ring finger protein 130 | 0.196 | 4.80E-02 |  | peptidase |
| RNF182 | ring finger protein 182 | -0.314 | 9.39E-02 |  | enzyme |
| RNF34 | ring finger protein 34. E3 ubiquitin protein ligase | -0.136 | 6.58E-02 |  | enzyme |
| SCD | stearoyl-CoA desaturase (delta-9-desaturase) | -0.241 | 8.00E-02 |  | enzyme |
| SEPT8 | septin 8 | 0.137 | 6.58E-02 |  | other |
| SERPINA10 | serpin peptidase inhibitor. clade A (alpha-1 antiproteinase. antitrypsin). member 10 | -0.463 | 5.25E-02 |  | other |
| SERPINH1 | serpin peptidase inhibitor. clade H (heat shock protein 47). member 1. (collagen binding protein 1) | -0.581 | 9.17E-02 |  | other |
| SLBP | stem-loop binding protein | 0.302 | 6.58E-02 |  | other |
| SLMO2 | slowmo homolog 2 (Drosophila) | -0.225 | 9.81E-02 |  | other |
| SLTM | SAFB-like. transcription modulator | 0.316 | 9.04E-02 |  | other |
| SMYD5 | SMYD family member 5 | -0.190 | 4.30E-02 |  | other |
| SPPL2A | signal peptide peptidase like 2A | 0.202 | 4.22E-02 |  | peptidase |
| SPTLC1 | serine palmitoyltransferase. long chain base subunit 1 | 0.151 | 9.81E-02 |  | enzyme |
| STC2 | stanniocalcin 2 | -0.358 | 4.78E-02 |  | other |
| STMN2 | stathmin 2 | 0.200 | 5.32E-02 | D | other |
| STMN2 | stathmin 2 | 0.134 | 8.91E-02 | D | other |
| STRC | stereocilin | 0.147 | 8.62E-02 |  | other |
| TGM1 | transglutaminase 1 | -0.234 | 7.20E-02 |  | enzyme |
| TMUB1 | transmembrane and ubiquitin-like domain containing 1 | -0.117 | 9.47E-02 |  | other |
| TTC7A | tetratricopeptide repeat domain 7A | 0.129 | 7.17E-02 |  | other |
| TUBA1A | tubulin. alpha 1a | 0.309 | 9.39E-02 |  | other |
| UBE2QL1 | ubiquitin-conjugating enzyme E2Q family-like 1 | -0.672 | 9.39E-02 |  | other |
| UGGT2 | UDP-glucose glycoprotein glucosyltransferase 2 | 0.229 | 4.22E-02 |  | enzyme |
| VIL1 | villin 1 | 0.359 | 9.88E-02 |  | other |
| VTN | vitronectin | 0.274 | 4.22E-02 | D | other |
| VTN | vitronectin | 0.316 | 9.39E-02 | D | other |
| XKR4 | XK. Kell blood group complex subunit-related family. member 4 | -0.230 | 9.32E-02 |  | other |
| XPOT | exportin. tRNA | -0.119 | 9.57E-02 |  | other |
| ZNF503 | zinc finger protein 503 | -0.199 | 9.04E-02 |  | other |
| ZNF729 | zinc finger protein 729 | -0.123 | 9.39E-02 |  | other |
