# Supplementary material for: Zebrafish Bone and General Physiology Are Differently Affected by Hormones or Changes in Gravity
Source: PLoS One. 2015 Jun 10;10(6):e0126928. doi: 10.1371/journal.pone.0126928 (PMC4465622; doi:10.1371/journal.pone.0126928)
Supplement: S15 Table — The indicates the human homolog of the gene, its "Entrez" gene name, the log ratio of (3g>1g) larvae compared to larvae kept at 3g between 0 and 6dpf, the presence of duplicate probes on the microarray (D) and the type of protein it encodes. Genes are arranged according to their type and in alphabetical order. (DOCX) [file pone.0126928.s022.docx]

| **Symbol** | **Entrez Gene Name** | **Log Ratio** | **p-value** | **N** | **Type(s)** |
| --- | --- | --- | --- | --- | --- |
| ABCA1 | ATP-binding cassette, sub-family A (ABC1), member 1 | -0,346 | 8,67E-02 |  | transporter |
| ABCA4 | ATP-binding cassette, sub-family A (ABC1), member 4 | 0,297 | 6,97E-02 |  | transporter |
| ABCB9 | ATP-binding cassette, sub-family B (MDR/TAP), member 9 | 0,164 | 6,09E-02 |  | transporter |
| ABCD3 | ATP-binding cassette, sub-family D (ALD), member 3 | 0,180 | 7,68E-02 |  | transporter |
| ABCE1 | ATP-binding cassette, sub-family E (OABP), member 1 | 0,118 | 9,61E-02 |  | transporter |
| ACTR6 | ARP6 actin-related protein 6 homolog (yeast) | -0,183 | 7,54E-02 |  | transporter |
| ANKH | ANKH inorganic pyrophosphate transport regulator | -0,221 | 3,25E-02 |  | transporter |
| AQP3 | aquaporin 3 (Gill blood group) | -0,254 | 9,64E-02 |  | transporter |
| ATP6V1C1 | ATPase, H+ transporting, lysosomal 42kDa, V1 subunit C1 | 0,103 | 8,42E-02 |  | transporter |
| CACNG1 | calcium channel, voltage-dependent, gamma subunit 1 | -0,246 | 8,70E-02 |  | ion channel |
| CACNG6 | calcium channel, voltage-dependent, gamma subunit 6 | -0,203 | 8,33E-02 |  | ion channel |
| CDH17 | cadherin 17, LI cadherin (liver-intestine) | 0,171 | 8,36E-02 |  | transporter |
| DDI2 | DNA-damage inducible 1 homolog 2 (S. cerevisiae) | 0,178 | 6,13E-02 |  | transporter |
| FDX1L | ferredoxin 1-like | 0,237 | 7,76E-02 |  | transporter |
| GJB3 | gap junction protein, beta 3, 31kDa | -0,185 | 8,08E-02 |  | transporter |
| GPM6A | glycoprotein M6A | -0,485 | 9,71E-02 |  | ion channel |
| GRID2 | glutamate receptor, ionotropic, delta 2 | 0,142 | 8,69E-02 |  | ion channel |
| HBE1 | hemoglobin, epsilon 1 | 0,377 | 8,20E-02 |  | transporter |
| HDLBP | high density lipoprotein binding protein | 0,274 | 7,74E-02 |  | transporter |
| HSDL2 | hydroxysteroid dehydrogenase like 2 | -0,188 | 2,44E-02 |  | transporter |
| KCNC2 | potassium voltage-gated channel, Shaw-related subfamily, member 2 | 0,183 | 6,03E-02 |  | ion channel |
| KCNG4 | potassium voltage-gated channel, subfamily G, member 4 | 0,189 | 6,03E-02 |  | ion channel |
| LRRCC1 | leucine rich repeat and coiled-coil centrosomal protein 1 | 0,175 | 4,25E-02 |  | transporter |
| MCL1 | myeloid cell leukemia 1 | -0,595 | 3,78E-02 |  | transporter |
| NPC1 | Niemann-Pick disease, type C1 | 0,215 | 2,96E-02 |  | transporter |
| NUP160 | nucleoporin 160kDa | -0,365 | 6,05E-02 |  | transporter |
| NUTF2 | nuclear transport factor 2 | -0,164 | 4,94E-02 |  | transporter |
| RHBG | Rh family, B glycoprotein (gene/pseudogene) | -0,436 | 1,42E-02 |  | transporter |
| RHCG | Rh family, C glycoprotein | -0,381 | 4,32E-02 |  | transporter |
| SCAMP2 | secretory carrier membrane protein 2 | 0,332 | 2,99E-02 |  | transporter |
| SCARB1 | scavenger receptor class B, member 1 | 0,110 | 5,13E-02 |  | transporter |
| SCFD1 | sec1 family domain containing 1 | -0,111 | 7,15E-02 |  | transporter |
| SCN8A | sodium channel, voltage gated, type VIII, alpha subunit | -0,224 | 7,81E-02 |  | ion channel |
| SEC61A1 | Sec61 alpha 1 subunit (S. cerevisiae) | 0,183 | 2,85E-02 |  | transporter |
| SEC63 | SEC63 homolog (S. cerevisiae) | -0,144 | 8,09E-02 |  | transporter |
| SFXN2 | sideroflexin 2 | -0,101 | 6,97E-02 |  | transporter |
| SLC15A1 | solute carrier family 15 (oligopeptide transporter), member 1 | 0,165 | 9,61E-02 |  | transporter |
| SLC25A4 | solute carrier family 25 (mitochondrial carrier; adenine nucleotide translocator), member 4 | -0,279 | 6,78E-02 |  | transporter |
| SLC43A2 | solute carrier family 43 (amino acid system L transporter), member 2 | -0,121 | 4,25E-02 |  | transporter |
| SLC5A1 | solute carrier family 5 (sodium/glucose cotransporter), member 1 | 0,645 | 4,25E-02 |  | transporter |
| SLC6A1 | solute carrier family 6 (neurotransmitter transporter), member 1 | 0,224 | 3,69E-02 |  | transporter |
| SLC6A19 | solute carrier family 6 (neutral amino acid transporter), member 19 | 0,350 | 6,30E-02 |  | transporter |
| SMC1A | structural maintenance of chromosomes 1A | -0,141 | 5,83E-02 |  | transporter |
| SRI | sorcin | -0,170 | 2,81E-02 | D | transporter |
| SRI | sorcin | -0,268 | 7,17E-02 | D | transporter |
| STAR | steroidogenic acute regulatory protein | 0,159 | 7,52E-02 |  | transporter |
| SYT12 | synaptotagmin XII | 0,219 | 5,16E-02 |  | transporter |
| TAPBP | TAP binding protein (tapasin) | 0,158 | 8,44E-02 |  | transporter |
| TMED7 | transmembrane emp24 protein transport domain containing 7 | 0,161 | 4,32E-02 |  | transporter |
| TMEM38A | transmembrane protein 38A | -0,161 | 7,70E-02 |  | ion channel |
| TRPC4AP | transient receptor potential cation channel, subfamily C, member 4 associated protein | 0,172 | 6,97E-02 |  | transporter |
| TRPM3 | transient receptor potential cation channel, subfamily M, member 3 | -0,081 | 7,77E-02 |  | ion channel |
| TRPV1 | transient receptor potential cation channel, subfamily V, member 1 | 0,171 | 2,56E-02 |  | ion channel |
| TSPAN1 | tetraspanin 1 | -0,172 | 2,68E-02 |  | transporter |
| TUSC3 | tumor suppressor candidate 3 | -0,225 | 2,53E-02 |  | transporter |
| VDAC3 | voltage-dependent anion channel 3 | 0,127 | 5,03E-02 |  | ion channel |
| VPS13A | vacuolar protein sorting 13 homolog A (S. cerevisiae) | 0,252 | 1,56E-02 |  | transporter |
| VPS4B | vacuolar protein sorting 4 homolog B (S. cerevisiae) | -0,081 | 9,61E-02 |  | transporter |
| XPO4 | exportin 4 | 0,230 | 7,26E-02 |  | transporter |
| ATF3 | activating transcription factor 3 | -0,321 | 1,42E-02 | D | transcription regulator |
| ATF3 | activating transcription factor 3 | -0,347 | 3,25E-02 | D | transcription regulator |
| BTAF1 | BTAF1 RNA polymerase II, B-TFIID transcription factor-associated, 170kDa | 0,141 | 6,24E-02 |  | transcription regulator |
| BTG2 | BTG family, member 2 | -2,141 | 1,42E-02 | D | transcription regulator |
| BTG2 | BTG family, member 2 | -2,186 | 3,04E-02 | D | transcription regulator |
| CBX4 | chromobox homolog 4 | -0,152 | 5,61E-02 |  | transcription regulator |
| CCAR1 | cell division cycle and apoptosis regulator 1 | 0,254 | 1,98E-02 |  | transcription regulator |
| CEBPD | CCAAT/enhancer binding protein (C/EBP), delta | -0,100 | 7,79E-02 |  | transcription regulator |
| CLUH | clustered mitochondria (cluA/CLU1) homolog | 0,190 | 7,24E-02 |  | translation regulator |
| CNBP | CCHC-type zinc finger, nucleic acid binding protein | -0,208 | 6,03E-02 | D | transcription regulator |
| CNBP | CCHC-type zinc finger, nucleic acid binding protein | -0,386 | 7,60E-02 | D | transcription regulator |
| CTCF | CCCTC-binding factor (zinc finger protein) | -0,174 | 2,56E-02 |  | transcription regulator |
| EED | embryonic ectoderm development | 0,135 | 8,81E-02 |  | transcription regulator |
| EGR1 | early growth response 1 | -0,406 | 7,41E-03 |  | transcription regulator |
| EGR2 | early growth response 2 | -0,424 | 4,43E-02 |  | transcription regulator |
| EGR3 | early growth response 3 | -0,350 | 8,40E-02 |  | transcription regulator |
| EIF1AY | eukaryotic translation initiation factor 1A, Y-linked | -0,263 | 7,79E-02 |  | translation regulator |
| EIF2B1 | eukaryotic translation initiation factor 2B, subunit 1 alpha, 26kDa | -0,170 | 9,69E-02 |  | translation regulator |
| EIF2S2 | eukaryotic translation initiation factor 2, subunit 2 beta, 38kDa | -0,237 | 4,81E-02 |  | translation regulator |
| EN2 | engrailed homeobox 2 | -0,096 | 7,68E-02 |  | transcription regulator |
| ERCC6 | excision repair cross-complementation group 6 | 0,565 | 1,04E-02 |  | transcription regulator |
| ETV6 | ets variant 6 | 0,219 | 3,25E-02 |  | transcription regulator |
| FOS | FBJ murine osteosarcoma viral oncogene homolog | -2,902 | 2,28E-02 |  | transcription regulator |
| FOSB | FBJ murine osteosarcoma viral oncogene homolog B | -2,076 | 3,61E-02 |  | transcription regulator |
| FOXK1 | forkhead box K1 | 0,084 | 8,54E-02 |  | transcription regulator |
| FOXQ1 | forkhead box Q1 | -0,339 | 7,07E-02 | D | transcription regulator |
| FOXQ1 | forkhead box Q1 | -0,598 | 5,07E-02 | D | transcription regulator |
| GABPA | GA binding protein transcription factor, alpha subunit 60kDa | 0,152 | 5,95E-02 |  | transcription regulator |
| GFM2 | G elongation factor, mitochondrial 2 | 0,165 | 7,27E-02 |  | translation regulator |
| HES1 | hes family bHLH transcription factor 1 | -0,132 | 8,75E-02 |  | transcription regulator |
| HEY1 | hes-related family bHLH transcription factor with YRPW motif 1 | -0,219 | 2,81E-02 |  | transcription regulator |
| HLF | hepatic leukemia factor | -0,117 | 6,48E-02 |  | transcription regulator |
| HNF4G | hepatocyte nuclear factor 4, gamma | 0,177 | 3,37E-02 |  | transcription regulator |
| HTATSF1 | HIV-1 Tat specific factor 1 | -0,166 | 3,31E-02 |  | transcription regulator |
| ID1 | inhibitor of DNA binding 1, dominant negative helix-loop-helix protein | -0,413 | 1,04E-02 |  | transcription regulator |
| ID2 | inhibitor of DNA binding 2, dominant negative helix-loop-helix protein | -0,270 | 1,84E-02 |  | transcription regulator |
| IGF2BP1 | insulin-like growth factor 2 mRNA binding protein 1 | -0,484 | 7,54E-02 |  | translation regulator |
| JUN | jun proto-oncogene | -0,462 | 3,01E-02 | D | transcription regulator |
| JUN | jun proto-oncogene | -0,410 | 6,90E-02 | D | transcription regulator |
| KCNIP3 | Kv channel interacting protein 3, calsenilin | -0,173 | 5,63E-02 |  | transcription regulator |
| KLF6 | Kruppel-like factor 6 | -0,185 | 5,16E-02 |  | transcription regulator |
| KMT2C | lysine (K)-specific methyltransferase 2C | 0,414 | 2,60E-02 |  | transcription regulator |
| MED16 | mediator complex subunit 16 | 0,267 | 5,01E-02 |  | transcription regulator |
| MED24 | mediator complex subunit 24 | 0,240 | 9,63E-02 |  | transcription regulator |
| MTRF1 | mitochondrial translational release factor 1 | 0,141 | 9,03E-02 |  | translation regulator |
| MYC | v-myc avian myelocytomatosis viral oncogene homolog | -0,408 | 5,62E-02 | D | transcription regulator |
| MYC | v-myc avian myelocytomatosis viral oncogene homolog | -0,273 | 7,24E-02 | D | transcription regulator |
| MYC | v-myc avian myelocytomatosis viral oncogene homolog | -0,410 | 3,41E-02 | D | transcription regulator |
| MYC | v-myc avian myelocytomatosis viral oncogene homolog | -0,377 | 5,09E-02 | D | transcription regulator |
| MYOG | myogenin (myogenic factor 4) | -0,206 | 7,60E-02 |  | transcription regulator |
| MYT1 | myelin transcription factor 1 | -0,224 | 5,13E-02 |  | transcription regulator |
| NCOA4 | nuclear receptor coactivator 4 | 0,164 | 7,59E-02 |  | transcription regulator |
| NCOR1 | nuclear receptor corepressor 1 | 0,276 | 2,68E-02 |  | transcription regulator |
| NEO1 | neogenin 1 | 0,242 | 5,22E-02 |  | transcription regulator |
| NFKBIA | nuclear factor of kappa light polypeptide gene enhancer in B-cells inhibitor, alpha | -0,734 | 3,99E-02 | D | transcription regulator |
| NFKBIA | nuclear factor of kappa light polypeptide gene enhancer in B-cells inhibitor, alpha | -0,606 | 1,67E-02 | D | transcription regulator |
| NFKBIA | nuclear factor of kappa light polypeptide gene enhancer in B-cells inhibitor, alpha | -0,617 | 5,85E-02 | D | transcription regulator |
| NFX1 | nuclear transcription factor, X-box binding 1 | 0,284 | 1,98E-02 |  | transcription regulator |
| NPAS4 | neuronal PAS domain protein 4 | -2,407 | 6,61E-02 |  | transcription regulator |
| NPM1 | nucleophosmin (nucleolar phosphoprotein B23, numatrin) | -0,238 | 8,05E-02 |  | transcription regulator |
| NR4A1 | nuclear receptor subfamily 4, group A, member 1 | -0,646 | 3,61E-02 |  | ligand-dependent nuclear receptor |
| PAX2 | paired box 2 | -0,100 | 9,43E-02 | D | transcription regulator |
| PAX2 | paired box 2 | -0,107 | 6,07E-02 | D | transcription regulator |
| PAX6 | paired box 6 | -0,250 | 7,52E-02 |  | transcription regulator |
| PAX9 | paired box 9 | -0,144 | 5,13E-02 |  | transcription regulator |
| PCID2 | PCI domain containing 2 | 0,210 | 1,62E-02 |  | transcription regulator |
| PER2 | period circadian clock 2 | 0,133 | 5,46E-02 |  | transcription regulator |
| PFDN5 | prefoldin subunit 5 | -0,297 | 4,25E-02 |  | transcription regulator |
| PIAS4 | protein inhibitor of activated STAT, 4 | -0,076 | 9,63E-02 |  | transcription regulator |
| PIR | pirin (iron-binding nuclear protein) | 0,222 | 3,81E-02 |  | transcription regulator |
| PITX2 | paired-like homeodomain 2 | -0,196 | 1,91E-02 |  | transcription regulator |
| PPP1R27 | protein phosphatase 1, regulatory subunit 27 | -0,216 | 5,30E-02 |  | transcription regulator |
| RAD54L2 | RAD54-like 2 (S. cerevisiae) | 0,251 | 4,83E-02 |  | transcription regulator |
| RB1 | retinoblastoma 1 | -0,091 | 8,94E-02 |  | transcription regulator |
| RUNX3 | runt-related transcription factor 3 | 0,182 | 8,46E-02 |  | transcription regulator |
| SCRT1 | scratch family zinc finger 1 | 0,235 | 1,84E-02 |  | transcription regulator |
| SIN3B | SIN3 transcription regulator family member B | 0,308 | 8,70E-02 |  | transcription regulator |
| SIX6 | SIX homeobox 6 | -0,105 | 5,80E-02 |  | transcription regulator |
| SKP1 | S-phase kinase-associated protein 1 | -0,202 | 1,84E-02 | D | transcription regulator |
| SKP1 | S-phase kinase-associated protein 1 | -0,189 | 4,30E-02 | D | transcription regulator |
| SKP1 | S-phase kinase-associated protein 1 | -0,204 | 6,37E-02 | D | transcription regulator |
| SKP1 | S-phase kinase-associated protein 1 | -0,139 | 9,10E-02 | D | transcription regulator |
| SMARCE1 | SWI/SNF related, matrix associated, actin dependent regulator of chromatin, subfamily e, member 1 | 0,450 | 1,98E-02 |  | transcription regulator |
| SOX10 | SRY (sex determining region Y)-box 10 | 0,146 | 3,25E-02 |  | transcription regulator |
| SOX2 | SRY (sex determining region Y)-box 2 | -0,113 | 6,44E-02 |  | transcription regulator |
| SOX3 | SRY (sex determining region Y)-box 3 | -0,154 | 6,14E-02 | D | transcription regulator |
| SOX3 | SRY (sex determining region Y)-box 3 | -0,173 | 9,40E-02 | D | transcription regulator |
| SP1 | Sp1 transcription factor | 0,331 | 1,17E-02 |  | transcription regulator |
| SUPT5H | suppressor of Ty 5 homolog (S. cerevisiae) | 0,184 | 4,25E-02 |  | transcription regulator |
| TAF5 | TAF5 RNA polymerase II, TATA box binding protein (TBP)-associated factor, 100kDa | 0,311 | 7,52E-02 |  | transcription regulator |
| TBX15 | T-box 15 | 0,094 | 6,78E-02 |  | transcription regulator |
| TEF | thyrotrophic embryonic factor | 0,402 | 4,38E-02 |  | transcription regulator |
| TFAP2C | transcription factor AP-2 gamma (activating enhancer binding protein 2 gamma) | -0,215 | 5,85E-02 |  | transcription regulator |
| THRB | thyroid hormone receptor, beta | -0,241 | 9,55E-02 |  | ligand-dependent nuclear receptor |
| TOB1 | transducer of ERBB2, 1 | -0,614 | 2,32E-02 | D | transcription regulator |
| TOB1 | transducer of ERBB2, 1 | -0,562 | 3,25E-02 | D | transcription regulator |
| TOB1 | transducer of ERBB2, 1 | -0,265 | 6,10E-02 | D | transcription regulator |
| TSFM | Ts translation elongation factor, mitochondrial | 0,216 | 2,35E-02 |  | translation regulator |
| USF2 | upstream transcription factor 2, c-fos interacting | -0,402 | 7,60E-02 |  | transcription regulator |
| WDR77 | WD repeat domain 77 | 0,193 | 5,71E-02 |  | transcription regulator |
| YAP1 | Yes-associated protein 1 | -0,140 | 5,77E-02 |  | transcription regulator |
| ZFP36L2 | ZFP36 ring finger protein-like 2 | -0,139 | 4,65E-02 |  | transcription regulator |
| ZNF131 | zinc finger protein 131 | 0,095 | 8,17E-02 |  | transcription regulator |
| ADRB2 | adrenoceptor beta 2, surface | -0,167 | 8,63E-02 |  | G-protein coupled receptor |
| B2M | beta-2-microglobulin | -0,258 | 4,50E-02 |  | transmembrane receptor |
| CALCRL | calcitonin receptor-like | -0,302 | 3,76E-02 |  | G-protein coupled receptor |
| CCR9 | chemokine (C-C motif) receptor 9 | -0,251 | 1,91E-02 |  | G-protein coupled receptor |
| CLDN3 | claudin 3 | -0,396 | 2,44E-02 | D | transmembrane receptor |
| CLDN3 | claudin 3 | -0,365 | 3,42E-02 | D | transmembrane receptor |
| F3 | coagulation factor III (thromboplastin, tissue factor) | -0,357 | 7,73E-03 |  | transmembrane receptor |
| FAM155B | family with sequence similarity 155, member B | 0,217 | 6,44E-02 |  | transmembrane receptor |
| GPR143 | G protein-coupled receptor 143 | 0,209 | 6,49E-02 |  | G-protein coupled receptor |
| GPR85 | G protein-coupled receptor 85 | -0,152 | 8,69E-02 |  | G-protein coupled receptor |
| IFNAR1 | interferon (alpha, beta and omega) receptor 1 | 0,236 | 2,63E-02 |  | transmembrane receptor |
| ILDR1 | immunoglobulin-like domain containing receptor 1 | 0,107 | 7,26E-02 |  | transmembrane receptor |
| LGALS3BP | lectin, galactoside-binding, soluble, 3 binding protein | 0,083 | 8,83E-02 |  | transmembrane receptor |
| SFRP5 | secreted frizzled-related protein 5 | -0,086 | 7,79E-02 |  | transmembrane receptor |
| TNFRSF19 | tumor necrosis factor receptor superfamily, member 19 | 0,210 | 4,13E-02 |  | transmembrane receptor |
| TNFRSF21 | tumor necrosis factor receptor superfamily, member 21 | 0,196 | 6,17E-02 |  | transmembrane receptor |
| CMTM7 | CKLF-like MARVEL transmembrane domain containing 7 | -0,232 | 4,71E-02 |  | cytokine |
| CXCL14 | chemokine (C-X-C motif) ligand 14 | -0,327 | 3,67E-02 |  | cytokine |
| EDN1 | endothelin 1 | -0,348 | 5,53E-02 |  | cytokine |
| BMP2 | bone morphogenetic protein 2 | -0,194 | 3,25E-02 |  | growth factor |
| CTGF | connective tissue growth factor | -0,539 | 3,68E-02 |  | growth factor |
| GMFB | glia maturation factor, beta | -0,283 | 7,33E-02 |  | growth factor |
| GRN | granulin | 0,428 | 3,25E-02 |  | growth factor |
| MST1 | macrophage stimulating 1 (hepatocyte growth factor-like) | 0,293 | 1,58E-02 |  | growth factor |
| OGN | osteoglycin | -0,144 | 9,58E-02 |  | growth factor |
| CKB | creatine kinase, brain | 0,299 | 5,01E-02 |  | kinase |
| DAK | dihydroxyacetone kinase 2 homolog (S. cerevisiae) | 0,152 | 3,36E-02 |  | kinase |
| Dclk1 | doublecortin-like kinase 1 | 0,320 | 7,09E-02 |  | kinase |
| DSTYK | dual serine/threonine and tyrosine protein kinase | 0,326 | 8,62E-02 |  | kinase |
| DUSP1 | dual specificity phosphatase 1 | -1,633 | 5,35E-03 | D | phosphatase |
| DUSP1 | dual specificity phosphatase 1 | -1,561 | 2,28E-02 | D | phosphatase |
| DUSP2 | dual specificity phosphatase 2 | -1,027 | 2,23E-02 |  | phosphatase |
| DUSP5 | dual specificity phosphatase 5 | -0,605 | 7,44E-02 |  | phosphatase |
| DUSP6 | dual specificity phosphatase 6 | -0,402 | 6,30E-02 |  | phosphatase |
| FXN | frataxin | -0,109 | 4,86E-02 |  | kinase |
| MAPK4 | mitogen-activated protein kinase 4 | 0,233 | 1,84E-02 |  | kinase |
| MINPP1 | multiple inositol-polyphosphate phosphatase 1 | 0,083 | 9,74E-02 |  | phosphatase |
| NT5C2 | 5'-nucleotidase, cytosolic II | -0,329 | 2,28E-02 |  | phosphatase |
| NT5E | 5'-nucleotidase, ecto (CD73) | 0,230 | 3,78E-02 | D | phosphatase |
| NT5E | 5'-nucleotidase, ecto (CD73) | 0,242 | 3,78E-02 | D | phosphatase |
| PACSIN1 | protein kinase C and casein kinase substrate in neurons 1 | 0,137 | 8,26E-02 |  | kinase |
| PAK1 | p21 protein (Cdc42/Rac)-activated kinase 1 | 0,162 | 4,37E-02 |  | kinase |
| PANK4 | pantothenate kinase 4 | 0,130 | 8,08E-02 |  | kinase |
| PDK2 | pyruvate dehydrogenase kinase, isozyme 2 | -0,371 | 2,35E-02 |  | kinase |
| PGK1 | phosphoglycerate kinase 1 | 0,114 | 8,40E-02 |  | kinase |
| PI4KA | phosphatidylinositol 4-kinase, catalytic, alpha | -0,149 | 7,79E-02 |  | kinase |
| PIK3C2A | phosphatidylinositol-4-phosphate 3-kinase, catalytic subunit type 2 alpha | 0,255 | 7,15E-02 |  | kinase |
| PIM2 | Pim-2 proto-oncogene, serine/threonine kinase | -0,282 | 6,61E-02 |  | kinase |
| PPP1R3D | protein phosphatase 1, regulatory subunit 3D | 0,197 | 8,33E-02 |  | phosphatase |
| PPP2R4 | protein phosphatase 2A activator, regulatory subunit 4 | -0,091 | 8,33E-02 |  | phosphatase |
| PPP3R1 | protein phosphatase 3, regulatory subunit B, alpha | 0,096 | 9,19E-02 |  | phosphatase |
| PRKAG1 | protein kinase, AMP-activated, gamma 1 non-catalytic subunit | -0,272 | 9,35E-02 |  | kinase |
| PRKAR1A | protein kinase, cAMP-dependent, regulatory, type I, alpha | 0,375 | 8,69E-02 |  | kinase |
| PRKCE | protein kinase C, epsilon | -0,137 | 8,08E-02 |  | kinase |
| RIPK4 | receptor-interacting serine-threonine kinase 4 | 0,214 | 4,25E-02 |  | kinase |
| ROCK2 | Rho-associated, coiled-coil containing protein kinase 2 | 0,267 | 9,32E-02 | D | kinase |
| ROCK2 | Rho-associated, coiled-coil containing protein kinase 2 | 0,248 | 9,43E-02 | D | kinase |
| SCYL3 | SCY1-like 3 (S. cerevisiae) | -0,113 | 9,64E-02 |  | kinase |
| SGK1 | serum/glucocorticoid regulated kinase 1 | -0,585 | 1,23E-02 | D | kinase |
| SGK1 | serum/glucocorticoid regulated kinase 1 | -0,585 | 2,31E-02 | D | kinase |
| SOCS3 | suppressor of cytokine signaling 3 | -2,729 | 3,84E-03 | D | phosphatase |
| SOCS3 | suppressor of cytokine signaling 3 | -2,775 | 9,54E-03 | D | phosphatase |
| SRPK1 | SRSF protein kinase 1 | 0,131 | 6,03E-02 |  | kinase |
| STK39 | serine threonine kinase 39 | -0,359 | 6,17E-02 |  | kinase |
| TBK1 | TANK-binding kinase 1 | 0,275 | 3,31E-02 |  | kinase |
| TEC | tec protein tyrosine kinase | 0,392 | 1,91E-02 |  | kinase |
| UCK2 | uridine-cytidine kinase 2 | -0,096 | 7,52E-02 |  | kinase |
| 2010107G12Rik | RIKEN cDNA 2010107G12 gene | 0,121 | 5,53E-02 |  | other |
| ABAT | 4-aminobutyrate aminotransferase | -0,636 | 5,20E-02 |  | enzyme |
| ACACB | acetyl-CoA carboxylase beta | -0,082 | 8,03E-02 |  | enzyme |
| ACE | angiotensin I converting enzyme | 0,524 | 9,22E-02 |  | peptidase |
| ACTA1 | actin, alpha 1, skeletal muscle | -0,412 | 8,56E-02 |  | other |
| ACTG1 | actin, gamma 1 | -14,161 | 2,61E00 | D | other |
| ACTG1 | actin, gamma 1 | -14,128 | 1,46E00 | D | other |
| ACTG1 | actin, gamma 1 | -12,414 | 4,61E-01 | D | other |
| ACTG2 | actin, gamma 2, smooth muscle, enteric | 0,541 | 7,52E-02 |  | other |
| ACTN4 | actinin, alpha 4 | 0,279 | 2,50E-02 | D | other |
| ACTN4 | actinin, alpha 4 | 0,296 | 5,16E-02 | D | other |
| ACTR10 | actin-related protein 10 homolog (S. cerevisiae) | 0,180 | 5,13E-02 |  | other |
| ACTR8 | ARP8 actin-related protein 8 homolog (yeast) | 0,252 | 4,62E-02 |  | other |
| ADCY8 | adenylate cyclase 8 (brain) | 0,413 | 1,04E-02 |  | enzyme |
| AIG1 | androgen-induced 1 | 0,130 | 2,99E-02 |  | other |
| ALDH6A1 | aldehyde dehydrogenase 6 family, member A1 | -0,112 | 4,38E-02 |  | enzyme |
| AMDHD1 | amidohydrolase domain containing 1 | 0,177 | 7,06E-02 |  | enzyme |
| ANKRD13C | ankyrin repeat domain 13C | 0,284 | 8,58E-02 |  | other |
| ANKRD9 | ankyrin repeat domain 9 | -0,574 | 6,98E-03 |  | other |
| ANXA4 | annexin A4 | -0,375 | 1,92E-02 |  | other |
| APC | adenomatous polyposis coli | 0,095 | 7,91E-02 |  | enzyme |
| API5 | apoptosis inhibitor 5 | -0,193 | 8,89E-02 |  | other |
| ARIH2 | ariadne RBR E3 ubiquitin protein ligase 2 | -0,181 | 9,60E-02 |  | enzyme |
| ARL3 | ADP-ribosylation factor-like 3 | -0,126 | 8,40E-02 |  | enzyme |
| ARRDC2 | arrestin domain containing 2 | 0,131 | 6,31E-02 |  | other |
| ATPAF2 | ATP synthase mitochondrial F1 complex assembly factor 2 | -0,132 | 4,54E-02 |  | other |
| ATXN3 | ataxin 3 | 0,535 | 3,31E-02 | D | peptidase |
| ATXN3 | ataxin 3 | 0,374 | 3,86E-02 | D | peptidase |
| BBS5 | Bardet-Biedl syndrome 5 | -0,133 | 5,13E-02 |  | other |
| BCAT1 | branched chain amino-acid transaminase 1, cytosolic | -0,123 | 6,46E-02 |  | enzyme |
| BFSP2 | beaded filament structural protein 2, phakinin | -0,876 | 4,83E-02 |  | other |
| BTF3L4 | basic transcription factor 3-like 4 | -0,330 | 3,67E-02 |  | other |
| BVES | blood vessel epicardial substance | -0,321 | 6,98E-03 | D | other |
| BVES | blood vessel epicardial substance | -0,299 | 3,28E-02 | D | other |
| C12orf66 | chromosome 12 open reading frame 66 | -0,086 | 7,43E-02 |  | other |
| C14orf119 | chromosome 14 open reading frame 119 | -0,181 | 5,62E-02 |  | other |
| C15orf27 | chromosome 15 open reading frame 27 | 0,105 | 6,36E-02 |  | other |
| C15orf59 | chromosome 15 open reading frame 59 | 0,202 | 5,75E-02 |  | other |
| C2orf47 | chromosome 2 open reading frame 47 | -0,124 | 7,49E-02 |  | other |
| C3 | complement component 3 | 0,275 | 5,47E-02 | D | peptidase |
| C3 | complement component 3 | 0,178 | 7,60E-02 | D | peptidase |
| CA10 | carbonic anhydrase X | 0,118 | 4,25E-02 |  | enzyme |
| CAPN1 | calpain 1, (mu/I) large subunit | 0,267 | 7,47E-02 |  | peptidase |
| CAPN3 | calpain 3, (p94) | -0,625 | 4,25E-02 |  | peptidase |
| CAPNS1 | calpain, small subunit 1 | -0,400 | 4,25E-02 |  | peptidase |
| CBWD1 | COBW domain containing 1 | -0,163 | 6,76E-02 |  | other |
| CCBL2 | cysteine conjugate-beta lyase 2 | -0,167 | 8,91E-02 |  | enzyme |
| CCDC28A | coiled-coil domain containing 28A | -0,233 | 2,56E-02 |  | other |
| CCDC53 | coiled-coil domain containing 53 | -0,156 | 4,39E-02 | D | other |
| CCDC53 | coiled-coil domain containing 53 | -0,129 | 6,79E-02 | D | other |
| CCT4 | chaperonin containing TCP1, subunit 4 (delta) | 0,170 | 3,04E-02 |  | other |
| CDC40 | cell division cycle 40 | 0,121 | 9,27E-02 |  | other |
| Cdc42 | cell division cycle 42 | -0,264 | 5,02E-02 |  | enzyme |
| CDC5L | cell division cycle 5-like | 0,277 | 8,59E-02 |  | other |
| CDC6 | cell division cycle 6 | -0,129 | 9,09E-02 |  | other |
| CDH8 | cadherin 8, type 2 | 0,151 | 7,79E-02 |  | other |
| CECR1 | cat eye syndrome chromosome region, candidate 1 | -0,139 | 3,31E-02 |  | enzyme |
| CEL | carboxyl ester lipase | 0,451 | 4,25E-02 |  | enzyme |
| CETP | cholesteryl ester transfer protein, plasma | 0,191 | 1,98E-02 |  | enzyme |
| CHAC1 | ChaC, cation transport regulator homolog 1 (E. coli) | -0,750 | 1,84E-02 |  | other |
| CHMP1A | charged multivesicular body protein 1A | 0,193 | 4,62E-02 |  | peptidase |
| Chmp4b | charged multivesicular body protein 4B | -0,274 | 3,87E-02 |  | other |
| CHMP5 | charged multivesicular body protein 5 | -0,216 | 2,49E-02 |  | other |
| CHORDC1 | cysteine and histidine-rich domain (CHORD) containing 1 | -0,217 | 5,58E-02 |  | other |
| CIAPIN1 | cytokine induced apoptosis inhibitor 1 | -0,182 | 7,31E-02 |  | other |
| CISH | cytokine inducible SH2-containing protein | -0,424 | 2,86E-02 |  | other |
| CLASP1 | cytoplasmic linker associated protein 1 | -0,125 | 5,25E-02 |  | other |
| CLDN12 | claudin 12 | -0,188 | 4,32E-02 |  | other |
| CLDN7 | claudin 7 | -0,164 | 5,38E-02 |  | other |
| CLDN9 | claudin 9 | -0,361 | 7,41E-03 | D | other |
| CLDN9 | claudin 9 | -0,201 | 1,71E-02 | D | other |
| CLDN9 | claudin 9 | -0,312 | 3,99E-02 | D | other |
| CLPX | caseinolytic mitochondrial matrix peptidase chaperone subunit | -0,175 | 6,25E-02 |  | enzyme |
| CLTC | clathrin, heavy chain (Hc) | 0,210 | 7,54E-02 |  | other |
| COL10A1 | collagen, type X, alpha 1 | 0,271 | 5,15E-02 |  | other |
| COL15A1 | collagen, type XV, alpha 1 | 0,434 | 7,84E-02 |  | other |
| COL4A1 | collagen, type IV, alpha 1 | 0,272 | 7,54E-02 |  | other |
| COL6A6 | collagen, type VI, alpha 6 | 0,090 | 8,60E-02 |  | other |
| COQ2 | coenzyme Q2 4-hydroxybenzoate polyprenyltransferase | -0,089 | 7,26E-02 |  | enzyme |
| CPA2 | carboxypeptidase A2 (pancreatic) | 0,166 | 6,08E-02 |  | peptidase |
| CPSF2 | cleavage and polyadenylation specific factor 2, 100kDa | 0,338 | 1,96E-02 |  | other |
| CPXM2 | carboxypeptidase X (M14 family), member 2 | 0,185 | 5,13E-02 |  | peptidase |
| CRY1 | cryptochrome circadian clock 1 | 0,796 | 9,03E-02 |  | enzyme |
| CRYBA2 | crystallin, beta A2 | -0,764 | 5,78E-02 |  | other |
| CRYGN | crystallin, gamma N | -0,161 | 5,13E-02 |  | other |
| CSNK2A3 | casein kinase 2, alpha 3 polypeptide | -0,311 | 5,53E-02 | D | other |
| CSNK2A3 | casein kinase 2, alpha 3 polypeptide | -0,314 | 7,74E-02 | D | other |
| CSTB | cystatin B (stefin B) | -0,354 | 5,31E-02 |  | peptidase |
| CTC1 | CTS telomere maintenance complex component 1 | 0,316 | 3,42E-02 |  | other |
| CTR9 | CTR9, Paf1/RNA polymerase II complex component | 0,188 | 3,96E-02 |  | other |
| CTSS | cathepsin S | 0,251 | 3,42E-02 | D | peptidase |
| CTSS | cathepsin S | 0,303 | 4,56E-02 | D | peptidase |
| CYP27C1 | cytochrome P450, family 27, subfamily C, polypeptide 1 | 0,257 | 4,90E-02 |  | other |
| CYR61 | cysteine-rich, angiogenic inducer, 61 | -1,160 | 9,40E-03 |  | other |
| DAB1 | Dab, reelin signal transducer, homolog 1 (Drosophila) | 0,361 | 3,68E-02 |  | other |
| DARS | aspartyl-tRNA synthetase | -0,283 | 3,25E-02 |  | enzyme |
| DCN | decorin | -0,191 | 7,16E-02 | D | other |
| DCN | decorin | -0,304 | 9,79E-02 | D | other |
| DDA1 | DET1 and DDB1 associated 1 | 0,151 | 2,99E-02 |  | other |
| DDB2 | damage-specific DNA binding protein 2, 48kDa | 0,154 | 6,63E-02 |  | other |
| DDIT4 | DNA-damage-inducible transcript 4 | -0,558 | 9,35E-03 | D | other |
| DDIT4 | DNA-damage-inducible transcript 4 | -0,546 | 2,44E-02 | D | other |
| DDOST | dolichyl-diphosphooligosaccharide--protein glycosyltransferase subunit (non-catalytic) | 0,251 | 2,60E-02 |  | enzyme |
| DDX27 | DEAD (Asp-Glu-Ala-Asp) box polypeptide 27 | -0,149 | 3,36E-02 |  | enzyme |
| DDX31 | DEAD (Asp-Glu-Ala-Asp) box polypeptide 31 | 0,120 | 3,78E-02 |  | enzyme |
| DDX39B | DEAD (Asp-Glu-Ala-Asp) box polypeptide 39B | 0,079 | 1,01E-01 |  | enzyme |
| DDX49 | DEAD (Asp-Glu-Ala-Asp) box polypeptide 49 | -0,159 | 7,64E-02 |  | enzyme |
| DDX51 | DEAD (Asp-Glu-Ala-Asp) box polypeptide 51 | 0,247 | 7,12E-02 |  | enzyme |
| DEDD2 | death effector domain containing 2 | -0,090 | 9,74E-02 |  | other |
| DENND5B | DENN/MADD domain containing 5B | 0,289 | 8,74E-02 |  | other |
| DEPTOR | DEP domain containing MTOR-interacting protein | -0,458 | 2,44E-02 |  | other |
| DHX16 | DEAH (Asp-Glu-Ala-His) box polypeptide 16 | 0,336 | 3,86E-02 | D | enzyme |
| DHX16 | DEAH (Asp-Glu-Ala-His) box polypeptide 16 | 0,229 | 6,24E-02 | D | enzyme |
| DNAJA3 | DnaJ (Hsp40) homolog, subfamily A, member 3 | -0,150 | 5,53E-02 |  | other |
| DSP | desmoplakin | 0,241 | 3,17E-02 |  | other |
| DTX2 | deltex 2, E3 ubiquitin ligase | 0,279 | 3,51E-02 |  | other |
| EFCAB14 | EF-hand calcium binding domain 14 | 0,422 | 8,67E-02 |  | other |
| EGLN1 | egl-9 family hypoxia-inducible factor 1 | 0,420 | 1,25E-02 |  | other |
| EHBP1 | EH domain binding protein 1 | 0,275 | 1,36E-02 |  | other |
| EHHADH | enoyl-CoA, hydratase/3-hydroxyacyl CoA dehydrogenase | 0,282 | 4,32E-02 |  | enzyme |
| EIF2B3 | eukaryotic translation initiation factor 2B, subunit 3 gamma, 58kDa | -0,232 | 6,79E-02 |  | other |
| EIF4EBP3 | eukaryotic translation initiation factor 4E binding protein 3 | 0,156 | 7,73E-02 |  | other |
| ELMOD2 | ELMO/CED-12 domain containing 2 | -0,103 | 7,24E-02 |  | other |
| ELOVL7 | ELOVL fatty acid elongase 7 | -1,400 | 1,48E-02 | D | enzyme |
| ELOVL7 | ELOVL fatty acid elongase 7 | -1,377 | 1,84E-02 | D | enzyme |
| EMC8 | ER membrane protein complex subunit 8 | -0,109 | 4,25E-02 | D | other |
| EMC8 | ER membrane protein complex subunit 8 | -0,112 | 9,25E-02 | D | other |
| ERGIC2 | ERGIC and golgi 2 | 0,196 | 9,19E-02 |  | other |
| ERRFI1 | ERBB receptor feedback inhibitor 1 | -0,522 | 1,39E-02 |  | other |
| ESRP2 | epithelial splicing regulatory protein 2 | 0,173 | 8,80E-02 |  | other |
| EVPL | envoplakin | 0,253 | 7,54E-02 |  | other |
| EXT2 | exostosin glycosyltransferase 2 | 0,491 | 5,85E-02 |  | enzyme |
| EXTL3 | exostosin-like glycosyltransferase 3 | 0,077 | 9,53E-02 |  | enzyme |
| F11R | F11 receptor | -0,141 | 9,25E-02 |  | other |
| F2 | coagulation factor II (thrombin) | 0,384 | 8,87E-02 |  | peptidase |
| FAAH2 | fatty acid amide hydrolase 2 | 0,179 | 3,78E-02 | D | enzyme |
| FAAH2 | fatty acid amide hydrolase 2 | 0,202 | 8,47E-02 | D | enzyme |
| FAM135A | family with sequence similarity 135, member A | 0,198 | 2,44E-02 |  | enzyme |
| FAM173A | family with sequence similarity 173, member A | -0,148 | 8,33E-02 |  | other |
| FAM195B | family with sequence similarity 195, member B | -0,266 | 9,43E-02 |  | other |
| FAM212A | family with sequence similarity 212, member A | -0,123 | 8,70E-02 |  | other |
| FAM213A | family with sequence similarity 213, member A | -0,196 | 9,09E-02 |  | other |
| FAM213B | family with sequence similarity 213, member B | 0,179 | 2,44E-02 |  | enzyme |
| FAM43A | family with sequence similarity 43, member A | -0,355 | 2,44E-02 |  | other |
| FAM57B | family with sequence similarity 57, member B | 0,227 | 8,33E-02 |  | enzyme |
| FAM91A1 | family with sequence similarity 91, member A1 | 0,207 | 5,95E-02 |  | other |
| FBLN2 | fibulin 2 | 0,230 | 3,88E-02 |  | other |
| FBXL4 | F-box and leucine-rich repeat protein 4 | 0,102 | 7,24E-02 |  | other |
| FBXO9 | F-box protein 9 | 0,193 | 8,75E-02 |  | enzyme |
| FEZ1 | fasciculation and elongation protein zeta 1 (zygin I) | 0,368 | 9,64E-02 |  | other |
| FIBCD1 | fibrinogen C domain containing 1 | 0,244 | 6,03E-02 |  | other |
| FLCN | folliculin | -0,131 | 9,25E-02 |  | other |
| FNBP4 | formin binding protein 4 | -0,225 | 8,26E-02 |  | other |
| FNIP1 | folliculin interacting protein 1 | 0,402 | 7,41E-03 |  | other |
| Folh1 | folate hydrolase 1 | 0,233 | 3,81E-02 |  | peptidase |
| FSCN2 | fascin actin-bundling protein 2, retinal | -0,117 | 4,90E-02 |  | other |
| FST | follistatin | -0,136 | 7,07E-02 |  | other |
| GADD45A | growth arrest and DNA-damage-inducible, alpha | -0,563 | 5,47E-02 |  | other |
| GADD45B | growth arrest and DNA-damage-inducible, beta | -0,956 | 2,34E-02 | D | other |
| GADD45B | growth arrest and DNA-damage-inducible, beta | -0,538 | 5,35E-03 | D | other |
| GALNT6 | polypeptide N-acetylgalactosaminyltransferase 6 | 0,316 | 5,75E-02 |  | enzyme |
| GAREM | GRB2 associated, regulator of MAPK1 | 0,175 | 9,50E-02 |  | other |
| GCSH | glycine cleavage system protein H (aminomethyl carrier) | -0,224 | 7,68E-02 |  | enzyme |
| GDI1 | GDP dissociation inhibitor 1 | -0,162 | 7,57E-02 |  | other |
| GDPD1 | glycerophosphodiester phosphodiesterase domain containing 1 | 0,157 | 6,63E-02 |  | enzyme |
| GMPR | guanosine monophosphate reductase | -0,187 | 7,47E-02 |  | enzyme |
| GNPNAT1 | glucosamine-phosphate N-acetyltransferase 1 | -0,157 | 8,05E-02 |  | enzyme |
| GOT2 | glutamic-oxaloacetic transaminase 2, mitochondrial | -0,229 | 8,53E-02 |  | enzyme |
| GPALPP1 | GPALPP motifs containing 1 | -0,151 | 4,26E-02 |  | other |
| GPHN | gephyrin | -0,120 | 4,25E-02 |  | enzyme |
| H1f0 | H1 histone family, member 0 | -0,611 | 8,33E-03 |  | other |
| H2AFY | H2A histone family, member Y | 0,171 | 2,28E-02 |  | other |
| HABP2 | hyaluronan binding protein 2 | 0,234 | 8,27E-02 |  | peptidase |
| HAL | histidine ammonia-lyase | 0,433 | 4,02E-02 |  | enzyme |
| HECTD1 | HECT domain containing E3 ubiquitin protein ligase 1 | -0,100 | 9,32E-02 |  | enzyme |
| HEMK1 | HemK methyltransferase family member 1 | -0,095 | 6,38E-02 |  | enzyme |
| HES5 | hes family bHLH transcription factor 5 | -0,156 | 8,75E-02 |  | other |
| HHIP | hedgehog interacting protein | 0,212 | 9,74E-02 |  | other |
| HIBADH | 3-hydroxyisobutyrate dehydrogenase | -0,226 | 5,68E-02 | D | enzyme |
| HIBADH | 3-hydroxyisobutyrate dehydrogenase | -0,160 | 7,37E-02 | D | enzyme |
| HIST2H2BE | histone cluster 2, H2be | -0,246 | 1,56E-02 |  | other |
| HMBS | hydroxymethylbilane synthase | -0,101 | 5,77E-02 |  | enzyme |
| HNRNPAB | heterogeneous nuclear ribonucleoprotein A/B | 0,085 | 7,84E-02 |  | enzyme |
| HPRT1 | hypoxanthine phosphoribosyltransferase 1 | -0,156 | 8,64E-02 |  | enzyme |
| HPS3 | Hermansky-Pudlak syndrome 3 | 0,147 | 5,27E-02 |  | other |
| HS2ST1 | heparan sulfate 2-O-sulfotransferase 1 | -0,105 | 6,90E-02 |  | enzyme |
| IARS | isoleucyl-tRNA synthetase | 0,490 | 2,44E-02 |  | enzyme |
| ICA1L | islet cell autoantigen 1,69kDa-like | 0,108 | 4,77E-02 |  | other |
| IDI1 | isopentenyl-diphosphate delta isomerase 1 | -0,262 | 5,13E-02 |  | enzyme |
| IGFBP1 | insulin-like growth factor binding protein 1 | -0,606 | 1,33E-02 |  | other |
| ISCU | iron-sulfur cluster assembly enzyme | -0,138 | 8,41E-02 | D | other |
| ISCU | iron-sulfur cluster assembly enzyme | -0,162 | 2,44E-02 | D | other |
| IST1 | increased sodium tolerance 1 homolog (yeast) | -0,260 | 3,88E-02 |  | other |
| ITM2C | integral membrane protein 2C | -0,239 | 1,39E-02 |  | other |
| IVD | isovaleryl-CoA dehydrogenase | -0,186 | 9,35E-02 |  | enzyme |
| JADE3 | jade family PHD finger 3 | 0,169 | 9,69E-02 |  | other |
| KDM2A | lysine (K)-specific demethylase 2A | 0,179 | 9,16E-02 |  | other |
| KDM4C | lysine (K)-specific demethylase 4C | 0,182 | 9,43E-02 |  | other |
| KIAA0196 | KIAA0196 | 0,303 | 3,78E-02 | D | other |
| KIAA0196 | KIAA0196 | 0,301 | 4,81E-02 | D | other |
| KIAA0907 | KIAA0907 | 0,208 | 9,35E-02 |  | other |
| KIAA1191 | KIAA1191 | 0,138 | 8,08E-02 |  | other |
| KIAA1429 | KIAA1429 | 0,215 | 1,84E-02 |  | other |
| KIAA2013 | KIAA2013 | 0,108 | 5,34E-02 |  | other |
| KLHL21 | kelch-like family member 21 | -0,253 | 2,44E-02 |  | other |
| KLHL35 | kelch-like family member 35 | 0,085 | 9,15E-02 |  | other |
| KRT222 | keratin 222 | -0,117 | 9,09E-02 |  | other |
| LAMA4 | laminin, alpha 4 | 0,230 | 2,49E-02 |  | enzyme |
| LAMB1 | laminin, beta 1 | -0,193 | 8,13E-02 |  | other |
| LAPTM4B | lysosomal protein transmembrane 4 beta | -0,176 | 2,72E-02 |  | other |
| LCP1 | lymphocyte cytosolic protein 1 (L-plastin) | 0,559 | 3,58E-02 |  | other |
| LCTL | lactase-like | -0,436 | 4,32E-02 |  | enzyme |
| LEPROT | leptin receptor overlapping transcript | -0,078 | 9,60E-02 |  | other |
| LGSN | lengsin, lens protein with glutamine synthetase domain | -0,752 | 7,85E-02 |  | enzyme |
| LOC100359916 | heterogeneous nuclear ribonucleoprotein K-like | 0,312 | 4,04E-02 |  | other |
| LRAT | lecithin retinol acyltransferase (phosphatidylcholine--retinol O-acyltransferase) | -0,091 | 7,95E-02 |  | enzyme |
| LRIT1 | leucine-rich repeat, immunoglobulin-like and transmembrane domains 1 | -0,173 | 3,31E-02 |  | other |
| LSM1 | LSM1, U6 small nuclear RNA associated | -0,141 | 9,79E-02 |  | other |
| LYSMD2 | LysM, putative peptidoglycan-binding, domain containing 2 | -0,098 | 6,03E-02 |  | other |
| LYSMD4 | LysM, putative peptidoglycan-binding, domain containing 4 | 0,082 | 7,54E-02 |  | other |
| MAB21L1 | mab-21-like 1 (C. elegans) | 0,129 | 4,73E-02 |  | other |
| MAF1 | MAF1 homolog (S. cerevisiae) | -0,318 | 8,38E-02 |  | other |
| Marcks | myristoylated alanine rich protein kinase C substrate | -0,313 | 3,42E-02 |  | other |
| MCM3 | minichromosome maintenance complex component 3 | -0,137 | 9,31E-02 |  | enzyme |
| MCTS1 | malignant T cell amplified sequence 1 | -0,110 | 7,55E-02 |  | other |
| MED28 | mediator complex subunit 28 | -0,107 | 6,44E-02 |  | other |
| METTL3 | methyltransferase like 3 | 0,195 | 8,46E-02 |  | enzyme |
| MFGE8 | milk fat globule-EGF factor 8 protein | -0,153 | 3,78E-02 |  | other |
| MGME1 | mitochondrial genome maintenance exonuclease 1 | -0,260 | 5,03E-02 |  | enzyme |
| MGST1 | microsomal glutathione S-transferase 1 | -0,267 | 2,49E-02 |  | enzyme |
| MKRN2 | makorin ring finger protein 2 | 0,244 | 1,91E-02 |  | other |
| MPC2 | mitochondrial pyruvate carrier 2 | -0,282 | 1,21E-02 | D | other |
| MPC2 | mitochondrial pyruvate carrier 2 | -0,372 | 5,28E-02 | D | other |
| MROH1 | maestro heat-like repeat family member 1 | -0,088 | 6,67E-02 |  | other |
| MRPL15 | mitochondrial ribosomal protein L15 | -0,155 | 7,92E-02 |  | other |
| MRPS18B | mitochondrial ribosomal protein S18B | -0,102 | 9,15E-02 |  | other |
| MRPS27 | mitochondrial ribosomal protein S27 | -0,176 | 3,92E-02 |  | other |
| MTHFD2 | methylenetetrahydrofolate dehydrogenase (NADP+ dependent) 2, methenyltetrahydrofolate cyclohydrolase | 0,174 | 4,54E-02 |  | enzyme |
| MVP | major vault protein | 0,255 | 7,52E-02 |  | other |
| MYH10 | myosin, heavy chain 10, non-muscle | 0,364 | 4,49E-02 |  | other |
| MYH11 | myosin, heavy chain 11, smooth muscle | 0,417 | 1,98E-02 |  | other |
| MYH4 | myosin, heavy chain 4, skeletal muscle | -0,294 | 3,25E-02 | D | enzyme |
| MYH4 | myosin, heavy chain 4, skeletal muscle | -0,357 | 8,26E-02 | D | enzyme |
| MYL6 | myosin, light chain 6, alkali, smooth muscle and non-muscle | 0,201 | 3,11E-02 |  | other |
| MYO15A | myosin XVA | 0,216 | 7,60E-02 |  | other |
| MYO1B | myosin IB | 0,189 | 4,11E-02 |  | other |
| NCF1 | neutrophil cytosolic factor 1 | 0,310 | 8,69E-02 |  | enzyme |
| NCKAP1L | NCK-associated protein 1-like | 0,322 | 3,36E-02 |  | other |
| NCOA5 | nuclear receptor coactivator 5 | 0,413 | 3,31E-02 |  | other |
| NDOR1 | NADPH dependent diflavin oxidoreductase 1 | 0,125 | 5,04E-02 |  | enzyme |
| NDUFAF1 | NADH dehydrogenase (ubiquinone) complex I, assembly factor 1 | -0,141 | 9,24E-02 |  | other |
| NEDD1 | neural precursor cell expressed, developmentally down-regulated 1 | -0,220 | 3,76E-02 |  | other |
| NIFK | nucleolar protein interacting with the FHA domain of MKI67 | -0,188 | 3,50E-02 |  | other |
| NLGN4X | neuroligin 4, X-linked | -0,226 | 2,29E-02 |  | enzyme |
| NOL9 | nucleolar protein 9 | 0,183 | 3,99E-02 |  | other |
| NOP58 | NOP58 ribonucleoprotein | -0,271 | 7,00E-02 |  | enzyme |
| NSUN2 | NOP2/Sun RNA methyltransferase family, member 2 | -0,122 | 4,62E-02 |  | enzyme |
| NUCB2 | nucleobindin 2 | 0,096 | 8,83E-02 |  | other |
| NUDCD1 | NudC domain containing 1 | 0,134 | 4,30E-02 |  | other |
| NUF2 | NUF2, NDC80 kinetochore complex component | -0,099 | 9,19E-02 |  | other |
| NUP205 | nucleoporin 205kDa | 0,350 | 6,48E-02 |  | other |
| NVL | nuclear VCP-like | 0,273 | 6,46E-02 |  | other |
| OBSL1 | obscurin-like 1 | -0,120 | 4,84E-02 |  | other |
| ODF3L1 | outer dense fiber of sperm tails 3-like 1 | 0,144 | 4,32E-02 |  | other |
| OLIG3 | oligodendrocyte transcription factor 3 | 0,235 | 6,34E-02 |  | other |
| OPTN | optineurin | 0,115 | 8,46E-02 |  | other |
| Otud5 | OTU domain containing 5 | 0,241 | 9,10E-02 |  | enzyme |
| PAPLN | papilin, proteoglycan-like sulfated glycoprotein | 0,223 | 6,03E-02 |  | other |
| PARN | poly(A)-specific ribonuclease | 0,305 | 6,03E-02 |  | enzyme |
| PDCD11 | programmed cell death 11 | -0,157 | 4,62E-02 |  | other |
| PDCL3 | phosducin-like 3 | -0,107 | 4,56E-02 |  | other |
| PDXDC1 | pyridoxal-dependent decarboxylase domain containing 1 | 0,207 | 6,50E-02 |  | other |
| PECAM1 | platelet/endothelial cell adhesion molecule 1 | 0,173 | 5,81E-02 |  | other |
| PEX26 | peroxisomal biogenesis factor 26 | -0,128 | 6,03E-02 |  | other |
| PFDN4 | prefoldin subunit 4 | -0,131 | 7,52E-02 |  | other |
| PHACTR3 | phosphatase and actin regulator 3 | 0,126 | 7,26E-02 |  | other |
| PICALM | phosphatidylinositol binding clathrin assembly protein | 0,333 | 8,86E-02 |  | other |
| PLAA | phospholipase A2-activating protein | 0,280 | 6,30E-02 |  | other |
| PLS1 | plastin 1 | -0,101 | 5,95E-02 |  | other |
| PLS3 | plastin 3 | -0,151 | 4,02E-02 |  | other |
| POLDIP2 | polymerase (DNA-directed), delta interacting protein 2 | 0,145 | 6,18E-02 |  | other |
| POLR1C | polymerase (RNA) I polypeptide C, 30kDa | -0,118 | 3,92E-02 |  | enzyme |
| PON2 | paraoxonase 2 | -0,114 | 6,10E-02 |  | enzyme |
| PRICKLE2 | prickle homolog 2 (Drosophila) | 0,101 | 7,79E-02 |  | other |
| PROC | protein C (inactivator of coagulation factors Va and VIIIa) | 0,266 | 4,61E-02 |  | peptidase |
| PRPF39 | pre-mRNA processing factor 39 | 0,190 | 5,26E-02 |  | other |
| PRPH | peripherin | 0,148 | 3,48E-02 |  | other |
| Prps1l3 | phosphoribosyl pyrophosphate synthetase 1-like 3 | 0,111 | 8,08E-02 |  | other |
| PSD3 | pleckstrin and Sec7 domain containing 3 | 0,144 | 9,74E-02 |  | other |
| PSMB7 | proteasome (prosome, macropain) subunit, beta type, 7 | -0,172 | 5,13E-02 |  | peptidase |
| PTCD3 | pentatricopeptide repeat domain 3 | -0,103 | 5,85E-02 |  | other |
| PTGIS | prostaglandin I2 (prostacyclin) synthase | -0,126 | 8,56E-02 |  | enzyme |
| Pwp2 | PWP2 periodic tryptophan protein homolog (yeast) | -0,100 | 7,64E-02 |  | other |
| QRICH1 | glutamine-rich 1 | 0,240 | 5,25E-02 |  | other |
| RAB28 | RAB28, member RAS oncogene family | 0,247 | 8,12E-02 |  | enzyme |
| RAB37 | RAB37, member RAS oncogene family | -0,118 | 7,54E-02 | D | enzyme |
| RAB37 | RAB37, member RAS oncogene family | -0,090 | 8,68E-02 | D | enzyme |
| RAB3IP | RAB3A interacting protein | 0,240 | 3,78E-02 |  | other |
| RAB40C | RAB40C, member RAS oncogene family | 0,207 | 3,79E-02 |  | enzyme |
| RAB5A | RAB5A, member RAS oncogene family | -0,114 | 5,96E-02 |  | enzyme |
| RAD23A | RAD23 homolog A (S. cerevisiae) | 0,080 | 9,63E-02 |  | other |
| RAD54L | RAD54-like (S. cerevisiae) | 0,080 | 9,15E-02 |  | enzyme |
| RANBP9 | RAN binding protein 9 | -0,207 | 8,62E-02 |  | other |
| RARS | arginyl-tRNA synthetase | 0,294 | 1,95E-02 |  | enzyme |
| RASAL2 | RAS protein activator like 2 | 0,203 | 4,83E-02 |  | other |
| RASD1 | RAS, dexamethasone-induced 1 | -0,240 | 8,08E-02 |  | enzyme |
| RBBP5 | retinoblastoma binding protein 5 | 0,204 | 7,52E-02 |  | other |
| RBM28 | RNA binding motif protein 28 | -0,344 | 2,04E-02 |  | other |
| RDH12 | retinol dehydrogenase 12 (all-trans/9-cis/11-cis) | 0,132 | 9,95E-02 |  | enzyme |
| REEP3 | receptor accessory protein 3 | -0,133 | 5,68E-02 |  | other |
| RETSAT | retinol saturase (all-trans-retinol 13,14-reductase) | 0,157 | 4,32E-02 |  | enzyme |
| RFC2 | replication factor C (activator 1) 2, 40kDa | -0,096 | 5,55E-02 |  | other |
| RGS1 | regulator of G-protein signaling 1 | -0,213 | 6,03E-02 |  | other |
| RGS14 | regulator of G-protein signaling 14 | 0,158 | 6,64E-02 | D | other |
| RGS14 | regulator of G-protein signaling 14 | 0,100 | 7,89E-02 | D | other |
| RGS16 | regulator of G-protein signaling 16 | -0,425 | 9,53E-02 |  | other |
| RHOC | ras homolog family member C | -0,143 | 6,85E-02 |  | enzyme |
| RNF103 | ring finger protein 103 | -0,126 | 4,81E-02 |  | enzyme |
| RNF144B | ring finger protein 144B | -0,197 | 9,48E-02 |  | enzyme |
| RNF20 | ring finger protein 20, E3 ubiquitin protein ligase | 0,158 | 3,72E-02 |  | enzyme |
| RNF213 | ring finger protein 213 | 0,230 | 2,99E-02 |  | enzyme |
| RNF7 | ring finger protein 7 | -0,229 | 9,15E-02 |  | enzyme |
| RP2 | retinitis pigmentosa 2 (X-linked recessive) | -0,140 | 7,81E-02 |  | enzyme |
| RPAP3 | RNA polymerase II associated protein 3 | 0,174 | 9,15E-02 |  | enzyme |
| RPP40 | ribonuclease P/MRP 40kDa subunit | -0,106 | 9,35E-02 |  | enzyme |
| RPS19 | ribosomal protein S19 | -0,331 | 9,60E-02 |  | other |
| RRAD | Ras-related associated with diabetes | -0,216 | 2,57E-02 | D | enzyme |
| RRAD | Ras-related associated with diabetes | -0,240 | 6,03E-02 | D | enzyme |
| RRM1 | ribonucleotide reductase M1 | -0,152 | 8,84E-02 |  | enzyme |
| RTTN | rotatin | 0,159 | 5,18E-02 |  | other |
| SAMSN1 | SAM domain, SH3 domain and nuclear localization signals 1 | 0,090 | 7,50E-02 |  | other |
| SAR1A | secretion associated, Ras related GTPase 1A | -0,356 | 5,22E-02 |  | enzyme |
| SARDH | sarcosine dehydrogenase | 0,212 | 7,48E-02 |  | enzyme |
| SARS2 | seryl-tRNA synthetase 2, mitochondrial | 0,184 | 9,40E-02 |  | enzyme |
| SCAPER | S-phase cyclin A-associated protein in the ER | 0,228 | 3,37E-02 |  | other |
| SCRN3 | secernin 3 | 0,164 | 2,53E-02 |  | other |
| SCUBE2 | signal peptide, CUB domain, EGF-like 2 | 0,262 | 7,54E-02 |  | other |
| SDHA | succinate dehydrogenase complex, subunit A, flavoprotein (Fp) | 0,329 | 3,17E-02 |  | enzyme |
| SEC23IP | SEC23 interacting protein | -0,130 | 4,88E-02 |  | other |
| SECISBP2L | SECIS binding protein 2-like | -0,201 | 9,15E-02 |  | other |
| SEPSECS | Sep (O-phosphoserine) tRNA:Sec (selenocysteine) tRNA synthase | 0,124 | 7,75E-02 |  | enzyme |
| Sept4 | septin 4 | 0,172 | 4,84E-02 |  | other |
| SERINC4 | serine incorporator 4 | 0,292 | 6,42E-02 |  | other |
| SERPINA9 | serpin peptidase inhibitor, clade A (alpha-1 antiproteinase, antitrypsin), member 9 | 0,142 | 7,48E-02 |  | other |
| SESTD1 | SEC14 and spectrin domains 1 | 0,221 | 7,04E-02 |  | other |
| SFSWAP | splicing factor, suppressor of white-apricot family | 0,376 | 7,93E-02 |  | other |
| SGPL1 | sphingosine-1-phosphate lyase 1 | 0,280 | 8,46E-02 |  | enzyme |
| SGSM3 | small G protein signaling modulator 3 | 0,328 | 8,25E-02 | D | other |
| SGSM3 | small G protein signaling modulator 3 | 0,293 | 9,15E-02 | D | other |
| SIRT6 | sirtuin 6 | 0,124 | 5,13E-02 |  | enzyme |
| SLC16A9 | solute carrier family 16, member 9 | -0,370 | 4,36E-02 |  | other |
| SLMO2 | slowmo homolog 2 (Drosophila) | -0,410 | 6,30E-02 |  | other |
| SLTM | SAFB-like, transcription modulator | 0,182 | 2,49E-02 |  | other |
| SMAP1 | small ArfGAP 1 | 0,195 | 9,63E-02 |  | other |
| SNX27 | sorting nexin family member 27 | -0,108 | 7,38E-02 |  | other |
| SOCS1 | suppressor of cytokine signaling 1 | -0,486 | 4,92E-02 |  | other |
| SOCS7 | suppressor of cytokine signaling 7 | 0,184 | 9,03E-02 |  | other |
| SOGA3 | SOGA family member 3 | -0,206 | 4,26E-02 |  | other |
| SORD | sorbitol dehydrogenase | 0,286 | 5,79E-02 |  | enzyme |
| SPAG7 | sperm associated antigen 7 | -0,154 | 5,90E-02 |  | other |
| SPPL2A | signal peptide peptidase like 2A | 0,308 | 8,08E-02 |  | peptidase |
| SSFA2 | sperm specific antigen 2 | 0,248 | 2,07E-02 |  | other |
| STAU2 | staufen double-stranded RNA binding protein 2 | -0,511 | 8,36E-02 | D | other |
| STAU2 | staufen double-stranded RNA binding protein 2 | -0,428 | 8,69E-02 | D | other |
| STIP1 | stress-induced phosphoprotein 1 | -0,148 | 7,31E-02 |  | other |
| STRC | stereocilin | 0,128 | 9,35E-02 |  | other |
| STRN | striatin, calmodulin binding protein | 0,126 | 6,24E-02 |  | other |
| STXBP4 | syntaxin binding protein 4 | -0,108 | 8,08E-02 |  | other |
| SVIL | supervillin | 0,228 | 2,44E-02 |  | other |
| SYBU | syntabulin (syntaxin-interacting) | 0,224 | 6,90E-02 |  | other |
| SYMPK | symplekin | 0,200 | 7,25E-02 |  | other |
| SYNPO2L | synaptopodin 2-like | -0,148 | 7,54E-02 |  | other |
| TAX1BP1 | Tax1 (human T-cell leukemia virus type I) binding protein 1 | 0,205 | 4,81E-02 |  | other |
| TCAP | titin-cap | -0,598 | 8,33E-02 |  | other |
| TCTN2 | tectonic family member 2 | 0,126 | 7,38E-02 |  | other |
| TEX10 | testis expressed 10 | 0,141 | 3,25E-02 |  | other |
| TFIP11 | tuftelin interacting protein 11 | 0,261 | 4,45E-02 |  | other |
| THEM4 | thioesterase superfamily member 4 | -0,102 | 5,78E-02 |  | enzyme |
| THNSL2 | threonine synthase-like 2 (S. cerevisiae) | 0,102 | 6,21E-02 |  | other |
| THOC3 | THO complex 3 | -0,120 | 8,27E-02 |  | other |
| TIPARP | TCDD-inducible poly(ADP-ribose) polymerase | -0,405 | 6,71E-03 |  | enzyme |
| TKTL2 | transketolase-like 2 | 0,444 | 2,85E-02 |  | enzyme |
| TMBIM4 | transmembrane BAX inhibitor motif containing 4 | -0,236 | 6,46E-02 |  | other |
| TMEM147 | transmembrane protein 147 | 0,315 | 4,69E-02 |  | other |
| TMEM183A | transmembrane protein 183A | -0,215 | 3,25E-02 |  | other |
| TMEM223 | transmembrane protein 223 | -0,169 | 5,61E-02 |  | other |
| TMEM237 | transmembrane protein 237 | 0,084 | 8,12E-02 |  | other |
| TMEM51 | transmembrane protein 51 | 0,314 | 1,98E-02 |  | other |
| TMOD4 | tropomodulin 4 (muscle) | -0,215 | 2,44E-02 |  | other |
| TMTC4 | transmembrane and tetratricopeptide repeat containing 4 | 0,151 | 3,78E-02 |  | other |
| TMX3 | thioredoxin-related transmembrane protein 3 | 0,452 | 5,95E-02 |  | enzyme |
| TPD52L2 | tumor protein D52-like 2 | 0,298 | 4,99E-02 |  | other |
| TPRKB | TP53RK binding protein | -0,115 | 8,08E-02 |  | other |
| TREH | trehalase (brush-border membrane glycoprotein) | 0,111 | 8,36E-02 |  | enzyme |
| TRMT61B | tRNA methyltransferase 61 homolog B (S. cerevisiae) | 0,169 | 3,42E-02 |  | enzyme |
| Tsc22d3 | TSC22 domain family, member 3 | -0,261 | 8,80E-02 |  | other |
| TSPAN12 | tetraspanin 12 | -0,179 | 3,61E-02 |  | other |
| TSPAN4 | tetraspanin 4 | 0,256 | 4,49E-02 |  | other |
| TTC7A | tetratricopeptide repeat domain 7A | 0,270 | 9,54E-03 |  | other |
| TXNIP | thioredoxin interacting protein | -0,472 | 9,43E-02 | D | other |
| TXNIP | thioredoxin interacting protein | -0,341 | 9,35E-02 | D | other |
| TYRP1 | tyrosinase-related protein 1 | 0,172 | 5,45E-02 |  | enzyme |
| U2AF2 | U2 small nuclear RNA auxiliary factor 2 | 0,093 | 9,79E-02 |  | other |
| UBA1 | ubiquitin-like modifier activating enzyme 1 | 0,106 | 9,09E-02 |  | enzyme |
| UBE2G1 | ubiquitin-conjugating enzyme E2G 1 | 0,192 | 3,04E-02 |  | enzyme |
| UBE2H | ubiquitin-conjugating enzyme E2H | -0,176 | 3,25E-02 |  | enzyme |
| UBE2I | ubiquitin-conjugating enzyme E2I | -0,200 | 2,44E-02 |  | enzyme |
| UBE2Q2 | ubiquitin-conjugating enzyme E2Q family member 2 | 0,362 | 8,33E-02 |  | enzyme |
| UBE4B | ubiquitination factor E4B | -0,154 | 9,10E-02 |  | enzyme |
| UBXN1 | UBX domain protein 1 | 0,203 | 2,12E-02 |  | other |
| UGGT2 | UDP-glucose glycoprotein glucosyltransferase 2 | 0,132 | 7,47E-02 |  | enzyme |
| UNC50 | unc-50 homolog (C. elegans) | -0,191 | 6,79E-02 |  | other |
| USP25 | ubiquitin specific peptidase 25 | 0,302 | 6,69E-02 | D | peptidase |
| USP25 | ubiquitin specific peptidase 25 | 0,259 | 8,67E-02 | D | peptidase |
| USP48 | ubiquitin specific peptidase 48 | 0,135 | 2,86E-02 |  | peptidase |
| USP5 | ubiquitin specific peptidase 5 (isopeptidase T) | 0,143 | 3,61E-02 |  | peptidase |
| UTP11L | UTP11-like, U3 small nucleolar ribonucleoprotein (yeast) | -0,188 | 5,28E-02 |  | other |
| VAMP3 | vesicle-associated membrane protein 3 | -0,315 | 2,07E-02 |  | other |
| Vma21 | VMA21 vacuolar H+-ATPase homolog (S. cerevisiae) | -0,106 | 6,12E-02 |  | other |
| VPS8 | vacuolar protein sorting 8 homolog (S. cerevisiae) | 0,291 | 9,09E-02 |  | other |
| WDR5 | WD repeat domain 5 | -0,151 | 6,34E-02 |  | other |
| WDR6 | WD repeat domain 6 | 0,119 | 4,81E-02 |  | other |
| WDR62 | WD repeat domain 62 | 0,223 | 6,86E-02 |  | other |
| WDR74 | WD repeat domain 74 | -0,177 | 2,28E-02 |  | other |
| WDR76 | WD repeat domain 76 | 0,163 | 4,25E-02 |  | other |
| WRAP73 | WD repeat containing, antisense to TP73 | 0,160 | 6,24E-02 |  | other |
| XKR9 | XK, Kell blood group complex subunit-related family, member 9 | -0,176 | 3,25E-02 |  | other |
| XPC | xeroderma pigmentosum, complementation group C | 0,447 | 5,46E-02 |  | other |
| YAE1D1 | Yae1 domain containing 1 | -0,306 | 7,85E-02 |  | other |
| YKT6 | YKT6 v-SNARE homolog (S. cerevisiae) | -0,143 | 4,04E-02 |  | enzyme |
| YTHDF3 | YTH domain family, member 3 | 0,121 | 8,62E-02 |  | other |
| ZMYM2 | zinc finger, MYM-type 2 | 0,121 | 5,18E-02 |  | other |
