# Supplementary material for: Zebrafish Bone and General Physiology Are Differently Affected by Hormones or Changes in Gravity
Source: PLoS One. 2015 Jun 10;10(6):e0126928. doi: 10.1371/journal.pone.0126928 (PMC4465622; doi:10.1371/journal.pone.0126928)
Supplement: S16 Table — The fold change and statistical significance (p-values) are given from the microarray data and the RT-qPCR confirmation experiments. In the 3g>axe experiment, the human KLF2 gene in S12 is actually the klf2b zebrafish ortholog, in contrast to the klf2a ortholog shown here. (DOCX) [file pone.0126928.s023.docx]

Table S16

|  | **1g (Inc)** | | | | **3g>axe** | | | | **3g>1g** | | | |
| --- | --- | --- | --- | --- | --- | --- | --- | --- | --- | --- | --- | --- |
|  | **microarray** | | **RT-PCR** | | **microarray** | | **RT-PCR** | | **microarray** | | **RT-PCR** | |
| **Gene** | **FC** | **p-value** | **FC** | **p-value** | **FC** | **p-value** | **FC** | **p-value** | **FC** | **p-value** | **FC** | **p-value** |
| *btg2* | 0.232 | 0.029 | 0.206 | < 0.001 | 0.353 | 0.048 | 0.335 | < 0.001 | 0.220 | 0.014 | 0.134 | < 0.001 |
| *cebpb* | 0.386 | 0.022 | 0.323 | < 0.001 | 0.395 | 0.033 | 0.326 | < 0.001 | 0.462 | 0.030 | 0.351 | < 0.001 |
| *fos* | 0.173 | 0.029 | 0.056 | < 0.001 | 0.247 | 0.098 | 0.202 | < 0.001 | 0.134 | 0.023 | 0.050 | < 0.001 |
| *fos b* | 0.229 | 0.009 | 0.494 | < 0.001 | 0.253 | 0.088 | 0.879 | < 0.001 | 0.237 | 0.036 | 0.311 | < 0.001 |
| *klf2a* | 0.616 | 0.087 | 0.476 | < 0.001 |  |  | 0.820 | < 0.001 | 0.533 | 0.010 | 0.419 | < 0.001 |
| *socs3a* | 0.177 | 0.029 | 0.177 | < 0.001 | 0.244 | 0.085 | 0.289 | < 0.001 | 0.146 | 0.004 | 0.132 | < 0.001 |
