# Supplementary material for: Zebrafish Bone and General Physiology Are Differently Affected by Hormones or Changes in Gravity
Source: PLoS One. 2015 Jun 10;10(6):e0126928. doi: 10.1371/journal.pone.0126928 (PMC4465622; doi:10.1371/journal.pone.0126928)
Supplement: S17 Table — The gene symbol and name is given, as well as the log(fold-change) values in the different experiments. Induction values are underlined in red (>1) or orange (between 0.378 and 1), repression values are underlined in blue (-0.378/-1) or green (<-1). (DOCX) [file pone.0126928.s024.docx]

| Symbol | Entrez Gene Name | 3g | 3g>axe | 3g>1g | 1g>3g | PTH | VitD3 |
| --- | --- | --- | --- | --- | --- | --- | --- |
| GADD45B | growth arrest and DNA-damage-inducible. beta | **-0.64** | **-0.31** | **-0.96** | **-0.23** |  |  |
| FOSB | FBJ murine osteosarcoma viral oncogene homolog B | **-2.12** | **-1.99** | **-2.08** |  |  | **-0.99** |
| FOXQ1 | forkhead box Q1 | **-0.58** | **-0.72** | **-0.60** |  |  | **0.69** |
| EGR1 | early growth response 1 | **-0.39** | **-0.32** | **-0.41** |  | **-0.73** |  |
| FOS | FBJ murine osteosarcoma viral oncogene homolog | **-2.53** | **-2.02** | **-2.90** |  |  |  |
| SOCS3 | suppressor of cytokine signaling 3 | **-2.50** | **-2.04** | **-2.78** |  |  |  |
| BTG2 | BTG family. member 2 | **-2.11** | **-1.50** | **-2.19** |  |  |  |
| ELOVL7 | ELOVL fatty acid elongase 7 | **-1.21** | **-1.13** | **-1.40** |  |  |  |
| SLMO2 | slowmo homolog 2 (Drosophila) | **-0.50** | **-0.23** | **-0.41** |  |  |  |
| LRIT1 | leucine-rich repeat. immunoglobulin-like and transmembrane domains 1 | **0.61** | **-0.26** | **-0.17** |  |  |  |
| ZNF729 | zinc finger protein 729 | **-0.28** | **-0.12** |  | **-0.16** | **-1.32** | **-0.47** |
| CYP2J2 | cytochrome P450. family 2. subfamily J. polypeptide 2 | **0.81** | **-0.23** |  | **0.42** | **-0.74** | **-1.02** |
| APOA4 | apolipoprotein A-IV | **-0.62** | **-0.57** |  | **-0.35** |  | **-1.76** |
| SLC6A19 | solute carrier family 6 (neutral amino acid transporter). member 19 | **0.63** |  | **0.35** | **-0.19** |  | **-0.77** |
| CYR61 | cysteine-rich. angiogenic inducer. 61 | **-0.80** |  | **-1.16** | **-0.37** |  |  |
| DUSP2 | dual specificity phosphatase 2 | **-0.72** |  | **-1.03** | **-0.39** |  |  |
| NFKBIA | nuclear factor of kappa light polypeptide gene enhancer in B-cells inhibitor. a | **-0.47** |  | **-0.73** | **-0.48** |  |  |
| ERRFI1 | ERBB receptor feedback inhibitor 1 | **-0.42** |  | **-0.52** | **-0.39** |  |  |
| ATF3 | activating transcription factor 3 | **-0.32** |  | **-0.35** | **-0.43** |  |  |
| HBE1 | hemoglobin. epsilon 1 |  | **0.43** | **0.38** | **-0.31** |  |  |
| PIM2 | Pim-2 proto-oncogene. serine/threonine kinase |  | **-0.45** | **-0.28** | **0.34** |  |  |
| SLTM | SAFB-like. transcription modulator |  | **0.32** | **0.18** | **0.35** |  |  |
| SLC15A1 | solute carrier family 15 (oligopeptide transporter). member 1 | **0.38** |  | **0.17** | **-0.49** |  |  |
| BCO1 | beta-carotene oxygenase 1 | **0.39** | **0.19** |  | **-0.13** |  |  |
| OPN1LW | opsin 1 (cone pigments). long-wave-sensitive | **-0.87** | **0.20** |  |  |  | **-1.54** |
| MYOG | myogenin (myogenic factor 4) | **-0.36** |  | **-0.21** |  |  | **-0.55** |
| WDR5 | WD repeat domain 5 | **-0.34** |  | **-0.15** |  | **-1.40** |  |
| STK39 | serine threonine kinase 39 | **-0.30** |  | **-0.36** |  |  | **-0.55** |
| LOX | lysyl oxidase | **-0.12** | **0.14** |  |  |  | **0.46** |
| SPPL2A | signal peptide peptidase like 2A |  | **0.20** | **0.31** |  | **0.54** |  |
| TTC7A | tetratricopeptide repeat domain 7A |  | **0.13** | **0.27** |  |  | **-0.43** |
| ATP1A1 | ATPase. Na+/K+ transporting. alpha 1 polypeptide | **0.61** | **-0.27** |  |  |  | **-0.39** |
| IGFBP1 | insulin-like growth factor binding protein 1 | **1.01** |  | **-0.61** |  |  | **1.92** |
| NPAS4 | neuronal PAS domain protein 4 | **-1.72** |  | **-2.41** |  |  |  |
| DUSP1 | dual specificity phosphatase 1 | **-1.38** |  | **-1.63** |  |  |  |
| TGM1 | transglutaminase 1 | **-0.57** | **-0.23** |  |  |  |  |
| CKAP2 | cytoskeleton associated protein 2 | **-0.48** | **0.36** |  |  |  |  |
| LGSN | lengsin. lens protein with glutamine synthetase domain | **-0.48** |  | **-0.75** |  |  |  |
| CRYGN | crystallin. gamma N | **-0.46** |  | **-0.16** |  |  |  |
| H1f0 | H1 histone family. member 0 | **-0.41** |  | **-0.61** |  |  |  |
| RRM1 | ribonucleotide reductase M1 | **-0.40** |  | **-0.15** |  |  |  |
| EGR2 | early growth response 2 | **-0.29** |  | **-0.42** |  |  |  |
| ANKRD9 | ankyrin repeat domain 9 |  | **-0.29** | **-0.57** |  |  |  |
| EFCAB14 | EF-hand calcium binding domain 14 |  | **0.41** | **0.42** |  |  |  |
| HES1 | hes family bHLH transcription factor 1 |  | **-0.40** | **-0.13** |  |  |  |
| EXT2 | exostosin glycosyltransferase 2 | **0.16** |  | **0.49** |  |  |  |
| FNIP1 | folliculin interacting protein 1 | **0.28** |  | **0.40** |  |  |  |
| HSP90AA1 | heat shock protein 90kDa alpha (cytosolic). class A member 1 | **0.38** | **-0.23** |  |  |  |  |
| COL4A1 | collagen. type IV. alpha 1 | **0.48** |  | **0.27** |  |  |  |
| CETP | cholesteryl ester transfer protein. plasma | **0.50** |  | **0.19** |  |  |  |
| ACE | angiotensin I converting enzyme | **0.51** |  | **0.52** |  |  |  |
| TEC | tec protein tyrosine kinase | **0.55** |  | **0.39** |  |  |  |
| CRY1 | cryptochrome circadian clock 1 | **0.56** |  | **0.80** |  |  |  |
| SYBU | syntabulin (syntaxin-interacting) | **0.58** |  | **0.22** |  |  |  |
| TSPAN4 | tetraspanin 4 | **0.64** |  | **0.26** |  |  |  |
| IARS | isoleucyl-tRNA synthetase | **0.74** |  | **0.49** |  |  |  |
| AMY2B | amylase. alpha 2B (pancreatic) | **1.05** | **0.26** |  |  |  |  |
| CEL | carboxyl ester lipase | **1.22** |  | **0.45** |  |  |  |
| SOCS1 | suppressor of cytokine signaling 1 |  |  | **-0.49** | **-0.83** |  | **-1.49** |
| CISH | cytokine inducible SH2-containing protein |  |  | **-0.42** | **-0.51** |  | **-1.78** |
| TXNIP | thioredoxin interacting protein |  |  | **-0.47** | **0.91** |  | **-0.55** |
| SGK1 | serum/glucocorticoid regulated kinase 1 |  |  | **-0.59** | **-0.52** | **-0.51** |  |
| MYC | v-myc avian myelocytomatosis viral oncogene homolog |  |  | **-0.41** | **-0.59** |  | **-0.44** |
| ARRDC2 | arrestin domain containing 2 |  |  | **0.13** | **-0.43** |  | **0.38** |
| ACTA2 | actin. alpha 2. smooth muscle. aorta | **-0.41** |  |  | **-0.14** |  |  |
| PFKFB4 | 6-phosphofructo-2-kinase/fructose-2.6-biphosphatase 4 | **-0.36** |  |  | **-0.47** |  |  |
| FKBP5 | FK506 binding protein 5 |  | **0.52** |  | **-1.23** |  |  |
| DUSP5 | dual specificity phosphatase 5 |  |  | **-0.61** | **-0.70** |  |  |
| TOB1 | transducer of ERBB2. 1 |  |  | **-0.61** | **-0.33** |  |  |
| RHCG | Rh family. C glycoprotein |  |  | **-0.38** | **-0.56** |  |  |
| ANXA4 | annexin A4 |  |  | **-0.38** | **-0.42** |  |  |
| PCDHA8 | protocadherin alpha 8 |  | **0.51** |  | **-0.25** |  |  |
| ITM2C | integral membrane protein 2C |  |  | **-0.24** | **-0.40** |  |  |
| CLK4 | CDC-like kinase 4 | **0.41** |  |  | **0.62** |  |  |
| ENDOU | endonuclease. polyU-specific | **0.85** |  |  | **-0.14** |  |  |
| ARR3 | arrestin 3. retinal (X-arrestin) | **1.03** |  |  | **-0.24** |  |  |
| CPA2 | carboxypeptidase A2 (pancreatic) |  |  | **0.17** |  | **-0.65** | **-0.64** |
| LECT1 | leukocyte cell derived chemotaxin 1 | **-1.10** |  |  |  |  | **-0.53** |
| TUBA8 | tubulin. alpha 8 | **-0.72** |  |  |  |  | **0.40** |
| BCKDK | branched chain ketoacid dehydrogenase kinase | **-0.59** |  |  |  |  | **-0.41** |
| KLF11 | Kruppel-like factor 11 | **-0.52** |  |  |  |  | **2.63** |
| KIF23 | kinesin family member 23 | **-0.47** |  |  |  | **1.44** |  |
| DBT | dihydrolipoamide branched chain transacylase E2 | **-0.47** |  |  |  |  | **-0.46** |
| ANLN | anillin. actin binding protein | **-0.46** |  |  |  | **-1.37** |  |
| SRSF1 | serine/arginine-rich splicing factor 1 | **-0.42** |  |  |  | **-0.58** |  |
| HADH | hydroxyacyl-CoA dehydrogenase | **-0.41** |  |  |  |  | **-0.41** |
| SLC37A4 | solute carrier family 37 (glucose-6-phosphate transporter). member 4 | **-0.37** |  |  |  |  | **-0.40** |
| PPP4C | protein phosphatase 4. catalytic subunit | **-0.35** |  |  |  |  | **0.40** |
| FCGBP | Fc fragment of IgG binding protein | **-0.34** |  |  |  |  | **-0.69** |
| SSB | Sjogren syndrome antigen B (autoantigen La) | **-0.33** |  |  |  | **-0.66** |  |
| PGM1 | phosphoglucomutase 1 | **-0.32** |  |  |  |  | **-0.51** |
| TUBB4B | tubulin. beta 4B class IVb | **-0.30** |  |  |  |  | **0.43** |
| HSP90B1 | heat shock protein 90kDa beta (Grp94). member 1 | **-0.29** |  |  |  |  | **0.49** |
| C2orf40 | chromosome 2 open reading frame 40 | **-0.28** |  |  |  | **1.06** |  |
| DNAJB11 | DnaJ (Hsp40) homolog. subfamily B. member 11 | **-0.23** |  |  |  | **-0.89** |  |
| DDC | dopa decarboxylase (aromatic L-amino acid decarboxylase) | **-0.21** |  |  |  |  | **-0.54** |
| FAIM | Fas apoptotic inhibitory molecule | **-0.12** |  |  |  | **1.94** |  |
| ACKR3 | atypical chemokine receptor 3 |  | **-0.20** |  |  | **-2.21** |  |
| STC2 | stanniocalcin 2 |  | **-0.36** |  |  |  | **-1.75** |
| LCTL | lactase-like |  |  | **-0.44** |  | **-1.25** |  |
| GADD45A | growth arrest and DNA-damage-inducible. alpha |  |  | **-0.56** |  |  | **-0.94** |
| HABP2 | hyaluronan binding protein 2 |  |  | **0.23** |  |  | **-1.00** |
| TSPAN1 | tetraspanin 1 |  |  | **-0.17** |  |  | **-0.94** |
| ACTA1 | actin. alpha 1. skeletal muscle |  |  | **-0.41** |  |  | **-0.69** |
| C2orf47 | chromosome 2 open reading frame 47 |  |  | **-0.12** |  | **-0.95** |  |
| PDK2 | pyruvate dehydrogenase kinase. isozyme 2 |  |  | **-0.37** |  |  | **-0.70** |
| TMX3 | thioredoxin-related transmembrane protein 3 |  |  | **0.45** |  | **0.60** |  |
| SERPINH1 | serpin peptidase inhibitor. clade H (heat shock protein 47). member 1. (collagen binding protein 1) |  | **-0.58** |  |  |  | **-0.47** |
| CALCRL | calcitonin receptor-like |  |  | **-0.30** |  | **0.74** |  |
| TNFRSF21 | tumor necrosis factor receptor superfamily. member 21 |  |  | **0.20** |  | **0.78** |  |
| CNBP | CCHC-type zinc finger. nucleic acid binding protein |  |  | **-0.39** |  |  | **-0.59** |
| VTN | vitronectin |  | **0.32** |  |  |  | **0.65** |
| VIL1 | villin 1 |  | **0.36** |  |  |  | **-0.59** |
| MYH11 | myosin. heavy chain 11. smooth muscle |  |  | **0.42** |  |  | **-0.52** |
| ILDR1 | immunoglobulin-like domain containing receptor 1 |  |  | **0.11** |  | **0.79** |  |
| ACTR6 | ARP6 actin-related protein 6 homolog (yeast) |  |  | **-0.18** |  |  | **-0.64** |
| ADRB2 | adrenoceptor beta 2. surface |  |  | **-0.17** |  |  | **-0.60** |
| PARN | poly(A)-specific ribonuclease |  |  | **0.31** |  |  | **0.44** |
| TREH | trehalase (brush-border membrane glycoprotein) |  |  | **0.11** |  |  | **-0.63** |
| GOT2 | glutamic-oxaloacetic transaminase 2. mitochondrial |  |  | **-0.23** |  |  | **-0.47** |
| TMOD4 | tropomodulin 4 (muscle) |  |  | **-0.22** |  |  | **-0.46** |
| GJB3 | gap junction protein. beta 3. 31kDa |  |  | **-0.19** |  |  | **-0.48** |
| ACAA1 | acetyl-CoA acyltransferase 1 |  | **0.18** |  |  |  | **-0.47** |
| NCOA4 | nuclear receptor coactivator 4 |  |  | **0.16** |  |  | **0.47** |
| TWF2 | twinfilin actin-binding protein 2 |  | **-0.19** |  |  |  | **-0.42** |
| GNE | glucosamine (UDP-N-acetyl)-2-epimerase/N-acetylmannosamine kinase |  | **0.19** |  |  |  | **-0.40** |
| POPDC3 | popeye domain containing 3 |  | **-0.13** |  |  |  | **-0.46** |
| CCBL2 | cysteine conjugate-beta lyase 2 |  |  | **-0.17** |  |  | **-0.38** |
| FOXK1 | forkhead box K1 |  |  | **0.08** |  |  | **0.43** |
| ACOX1 | acyl-CoA oxidase 1. palmitoyl | **0.11** |  |  |  |  | **-0.85** |
| SLC25A47 | solute carrier family 25. member 47 | **0.13** |  |  |  |  | **-0.72** |
| EEF2 | eukaryotic translation elongation factor 2 | **0.18** |  |  |  | **0.72** |  |
| CYP27A1 | cytochrome P450. family 27. subfamily A. polypeptide 1 | **0.19** |  |  |  |  | **-0.48** |
| NID1 | nidogen 1 | **0.23** |  |  |  |  | **-0.67** |
| PDLIM1 | PDZ and LIM domain 1 | **0.24** |  |  |  | **-0.94** |  |
| ETNPPL | ethanolamine-phosphate phospho-lyase | **0.25** |  |  |  |  | **-0.75** |
| TMPRSS13 | transmembrane protease. serine 13 | **0.28** |  |  |  |  | **0.38** |
| SMPDL3B | sphingomyelin phosphodiesterase. acid-like 3B | **0.28** |  |  |  |  | **-0.78** |
| INSIG1 | insulin induced gene 1 | **0.31** |  |  |  |  | **0.62** |
| CACNA2D2 | calcium channel. voltage-dependent. alpha 2/delta subunit 2 | **0.31** |  |  |  |  | **0.38** |
| MOGAT1 | monoacylglycerol O-acyltransferase 1 | **0.33** |  |  |  |  | **-0.59** |
| HGD | homogentisate 1.2-dioxygenase | **0.35** |  |  |  |  | **-0.53** |
| SERPINB6 | serpin peptidase inhibitor. clade B (ovalbumin). member 6 | **0.41** |  |  |  |  | **-0.53** |
| CTRB2 | chymotrypsinogen B2 | **0.43** |  |  |  |  | **-0.79** |
| HSD17B4 | hydroxysteroid (17-beta) dehydrogenase 4 | **0.44** |  |  |  |  | **-0.45** |
| TAT | tyrosine aminotransferase | **1.04** |  |  |  |  | **1.60** |
| CYP24A1 | cytochrome P450. family 24. subfamily A. polypeptide 1 | **1.26** |  |  |  |  | **3.16** |
| NDRG2 | NDRG family member 2 |  |  |  | **-0.75** | **0.63** |  |
| GRK7 | G protein-coupled receptor kinase 7 |  |  |  | **-0.54** |  | **-0.75** |
| SLC25A43 | solute carrier family 25. member 43 |  |  |  | **-0.50** |  | **-0.69** |
| LPL | lipoprotein lipase |  |  |  | **-0.20** |  | **-0.87** |
| CTDSPL | CTD (carboxy-terminal domain. RNA polymerase II. polypeptide A) small phosphatase-like |  |  |  | **-0.25** |  | **0.56** |
| ALAS2 | 5'-aminolevulinate synthase 2 |  |  |  | **-0.11** |  | **-0.68** |
| HSD11B2 | hydroxysteroid (11-beta) dehydrogenase 2 |  |  |  | **-0.38** |  | **-0.41** |
| BOC | BOC cell adhesion associated. oncogene regulated |  |  |  | **-0.17** |  | **-0.56** |
| GUSB | glucuronidase. beta |  |  |  | **-0.15** | **0.54** |  |
| ANKRD33 | ankyrin repeat domain 33 |  |  |  | **-0.14** |  | **-0.55** |
| USP14 | ubiquitin specific peptidase 14 (tRNA-guanine transglycosylase) |  |  |  | **0.15** |  | **0.49** |
| ALDH4A1 | aldehyde dehydrogenase 4 family. member A1 |  |  |  | **-0.13** |  | **-0.46** |
| USP37 | ubiquitin specific peptidase 37 |  |  |  | **0.21** |  | **0.39** |
| NRBP2 | nuclear receptor binding protein 2 |  |  |  |  | **-3.12** | **-0.48** |
| CES1 | carboxylesterase 1 |  |  |  |  | **0.56** | **-2.24** |
| SLC6A18 | solute carrier family 6 (neutral amino acid transporter). member 18 |  |  |  |  | **-2.30** | **-0.46** |
| CAD | carbamoyl-phosphate synthetase 2. aspartate transcarbamylase. and dihydroorotase |  |  |  |  | **-1.57** | **0.51** |
| C7 | complement component 7 |  |  |  |  | **1.31** | **0.67** |
| KRT17 | keratin 17. type I |  |  |  |  | **0.96** | **0.52** |
| SLC43A1 | solute carrier family 43 (amino acid system L transporter). member 1 |  |  |  |  | **0.41** | **-1.07** |
| GSR | glutathione reductase |  |  |  |  | **-0.85** | **0.38** |
| MLEC | malectin |  |  |  |  | **-0.65** | **-0.40** |
| HSD3B7 | hydroxy-delta-5-steroid dehydrogenase. 3 beta- and steroid delta-isomerase 7 |  |  |  |  | **-0.63** | **-0.41** |
