# Supplementary material for: Zebrafish Bone and General Physiology Are Differently Affected by Hormones or Changes in Gravity
Source: PLoS One. 2015 Jun 10;10(6):e0126928. doi: 10.1371/journal.pone.0126928 (PMC4465622; doi:10.1371/journal.pone.0126928)
Supplement: S18 Table — The corresponding—Log(p-value) obtained in IPA analysis was used for classification and are coded by underlining: red means >3, orange between 1 and 3, and yellow means <1. (DOCX) [file pone.0126928.s025.docx]

| Canonical Pathway | 3g | 3g>axe | 3g>1g | 1g>3g | PTH | VitD3 |
| --- | --- | --- | --- | --- | --- | --- |
| IGF-1 Signaling | 2.77 | 0.72 | 3.87 | 3.59 |  | 2.24 |
| Tight Junction Signaling | 1.42 | 0.39 | 7.63 | 0.35 | 0.55 |  |
| JAK/Stat Signaling | 1.08 | 0.92 | 2.90 | 4.31 |  | 0.92 |
| Mitotic Roles of Polo-Like Kinase | 6.57 | 0.98 | 0.51 |  | 1.19 |  |
| Prolactin Signaling | 1.07 | 0.91 | 3.54 | 2.36 |  | 1.26 |
| ERK/MAPK Signaling | 2.41 | 1.15 | 2.01 | 2.14 |  |  |
| Glucocorticoid Receptor Signaling | 2.55 | 1.66 | 1.06 | 2.00 |  | 0.60 |
| Erythropoietin Signaling | 1.17 | 0.97 | 2.45 | 2.49 |  | 0.23 |
| ILK Signaling | 2.42 |  | 3.87 | 0.62 | 0.94 | 2.14 |
| GADD45 Signaling | 3.10 | 0.83 | 0.97 | 1.90 |  | 0.38 |
| PPAR Signaling | 1.68 | 1.36 | 0.52 | 2.78 | 0.93 | 0.25 |
| Remodeling of Epithelial Adherens Junctions | 3.81 | 0.36 | 1.82 | 0.33 | 0.46 | 0.53 |
| VEGF Signaling | 1.24 | 0.27 | 3.46 | 1.30 |  | 0.63 |
| Insulin Receptor Signaling | 1.06 | 0.51 | 1.74 | 2.85 | 0.66 | 0.35 |
| Sertoli Cell-Sertoli Cell Junction Signaling | 1.65 | 0.73 | 3.05 | 0.65 |  |  |
| Epithelial Adherens Junction Signaling | 3.87 |  | 1.53 | 0.42 | 0.22 | 1.58 |
| CDK5 Signaling | 0.72 | 0.70 | 3.16 | 1.21 | 0.32 |  |
| PI3K/AKT Signaling | 3.28 | 0.57 | 0.31 | 1.59 |  | 0.37 |
| Growth Hormone Signaling | 0.33 | 2.59 | 1.79 | 0.89 | 0.43 | 1.89 |
| Cell Cycle Control of Chromosomal Replication | 3.49 | 0.70 | 0.72 | 0.66 |  | 0.25 |
| Role of JAK2 in Hormone-like Cytokine Signaling | 1.34 | 0.60 | 1.08 | 2.41 |  | 1.30 |
| Integrin Signaling | 2.61 |  | 1.77 | 0.95 | 1.42 | 0.58 |
| FAK Signaling | 1.31 | 0.29 | 2.38 | 1.35 |  |  |
| Renin-Angiotensin Signaling | 0.62 | 1.21 | 2.30 | 1.11 |  |  |
| Cell Cycle: G2/M DNA Damage Checkpoint Regulation | 4.86 |  | 0.37 |  |  | 0.37 |
| GNRH Signaling | 0.47 | 2.35 | 1.42 | 0.95 |  |  |
| Valine Degradation I | 1.20 |  | 3.86 |  |  | 0.98 |
| Cellular Effects of Sildenafil (Viagra) | 0.76 |  | 3.35 | 0.95 |  | 0.50 |
| IL-2 Signaling | 0.47 | 0.45 | 1.15 | 2.87 |  | 0.40 |
| Telomerase Signaling | 0.72 | 0.70 | 0.77 | 2.68 |  | 0.36 |
| Gα12/13 Signaling | 0.88 | 0.20 | 2.10 | 1.66 |  |  |
| Agrin Interactions at Neuromuscular Junction | 1.14 | 0.36 | 2.38 | 0.89 |  | 0.24 |
| Neuregulin Signaling | 1.29 | 0.78 | 0.58 | 2.07 |  | 0.37 |
| Regulation of IL-2 Expression in Activated and Anergic T Lymphocytes | 0.97 | 0.32 | 1.07 | 2.24 |  |  |
| Notch Signaling |  | 2.42 | 1.60 | 0.53 |  |  |
| STAT3 Pathway | 0.63 | 0.34 | 1.19 | 2.36 |  | 1.26 |
| Cyclins and Cell Cycle Regulation | 2.69 | 0.32 | 0.70 | 0.80 |  |  |
| Mechanisms of Viral Exit from Host Cells | 0.62 |  | 1.49 | 2.22 |  |  |
| PDGF Signaling | 1.00 | 0.32 | 0.71 | 2.28 |  | 0.44 |
| TNFR1 Signaling | 0.51 | 2.11 | 1.25 | 0.44 | 0.58 |  |
| Gap Junction Signaling | 0.84 |  | 2.19 | 1.28 |  |  |
| Role of JAK1 and JAK3 in γc Cytokine Signaling | 0.76 | 0.39 | 0.55 | 2.59 |  | 1.37 |
| Huntington's Disease Signaling |  | 0.90 | 2.06 | 1.23 | 1.21 |  |
| TNFR2 Signaling | 0.85 | 0.67 | 2.00 | 0.63 |  |  |
| LXR/RXR Activation | 0.84 | 2.47 | 0.32 | 0.52 | 0.72 | 2.97 |
| FXR/RXR Activation | 0.78 | 2.38 |  | 0.96 | 0.24 | 4.75 |
| Leukocyte Extravasation Signaling | 0.52 |  | 3.56 |  |  |  |
| Actin Cytoskeleton Signaling | 0.90 |  | 2.28 | 0.87 | 0.39 | 0.51 |
| Thyroid Cancer Signaling | 1.20 | 0.55 |  | 2.25 | 0.64 |  |
| Amyloid Processing |  | 0.46 | 2.42 | 1.11 |  |  |
| Mouse Embryonic Stem Cell Pluripotency |  |  | 1.21 | 2.76 |  | 0.31 |
| Urate Biosynthesis/Inosine 5'-phosphate Degradation | 0.56 |  | 1.20 | 2.16 |  |  |
| Small Cell Lung Cancer Signaling | 2.25 |  | 0.80 | 0.87 | 0.43 | 0.25 |
| IL-9 Signaling | 0.74 | 0.61 | 1.11 | 1.42 |  | 2.05 |
| Cell Cycle Regulation by BTG Family Proteins | 2.09 | 0.60 | 1.08 |  | 0.70 |  |
| Tyrosine Degradation I | 2.30 |  |  | 1.35 |  | 3.40 |
| Fatty Acid β-oxidation I | 2.34 | 0.66 | 0.65 |  |  | 2.91 |
| IL-1 Signaling |  | 0.27 | 1.28 | 2.02 |  |  |
| Protein Kinase A Signaling | 0.42 |  | 0.97 | 2.07 |  | 0.39 |
| Citrulline-Nitric Oxide Cycle | 2.13 | 1.31 |  |  |  |  |
| Chronic Myeloid Leukemia Signaling |  | 0.27 | 0.28 | 2.80 |  |  |
| DNA damage-induced 14-3-3σ Signaling | 3.10 |  |  |  |  |  |
| Protein Ubiquitination Pathway | 1.83 |  | 0.87 | 0.39 | 2.23 |  |
| β-alanine Degradation I |  |  | 3.06 |  |  |  |
| Amyotrophic Lateral Sclerosis Signaling | 0.73 | 0.25 | 2.07 |  |  | 0.69 |
| Acute Myeloid Leukemia Signaling |  | 0.32 | 0.40 | 2.28 |  | 0.95 |
| Tryptophan Degradation X (Mammalian, via Tryptamine) | 2.13 |  |  | 0.82 |  | 1.43 |
| Histidine Degradation III |  |  | 2.89 |  |  |  |
| Glutathione Redox Reactions I | 0.47 |  | 0.38 | 1.94 | 0.84 | 2.03 |
| Superpathway of Methionine Degradation |  |  | 0.22 | 2.56 | 0.47 | 0.81 |
| Agranulocyte Adhesion and Diapedesis | 0.36 |  | 2.38 |  |  | 1.33 |
| Oncostatin M Signaling | 0.26 |  |  | 2.45 |  | 0.25 |
| G Beta Gamma Signaling |  |  | 0.58 | 2.07 |  |  |
| nNOS Signaling in Neurons |  |  | 2.61 |  |  |  |
| IL-4 Signaling | 0.28 |  |  | 2.30 |  | 0.77 |
| Xenobiotic Metabolism Signaling |  | 1.12 |  | 1.43 | 0.29 | 2.12 |
| EIF2 Signaling | 0.38 |  |  | 2.16 | 0.48 |  |
| Ethanol Degradation IV | 1.20 |  | 0.38 | 0.82 |  | 2.37 |
| Phenylalanine Degradation IV (Mammalian, via Side Chain) | 0.56 |  | 0.46 | 0.92 |  | 2.52 |
| Glutathione-mediated Detoxification |  |  | 0.25 | 1.58 |  | 3.40 |
| Melatonin Degradation I | 0.90 | 0.44 |  | 0.41 |  | 2.83 |
| Superpathway of Melatonin Degradation | 0.82 | 0.41 |  | 0.38 |  | 2.62 |
| PXR/RXR Activation |  | 0.37 | 0.87 | 0.34 | 0.44 | 3.06 |
| Complement System | 0.76 |  | 0.20 | 0.58 | 0.72 | 2.43 |
| Histamine Degradation | 0.59 |  |  | 0.95 |  | 2.88 |
| Nicotine Degradation III | 0.49 | 0.46 |  | 0.43 |  | 2.96 |
| Nicotine Degradation II | 0.40 | 0.40 |  | 0.37 |  | 2.83 |
| Thyroid Hormone Metabolism II (via Conjugation and/or Degradation) | 0.78 |  | 0.21 |  |  | 2.68 |
| LPS/IL-1 Mediated Inhibition of RXR Function |  |  | 0.49 | 0.49 | 0.39 | 4.90 |
| Bile Acid Biosynthesis, Neutral Pathway | 0.59 |  |  |  | 0.58 | 3.11 |
| Glucocorticoid Biosynthesis |  |  |  |  | 2.05 | 0.88 |
| Mineralocorticoid Biosynthesis |  |  |  |  | 2.05 | 0.88 |
| Phosphatidylcholine Biosynthesis I |  |  |  |  |  | 2.88 |
| Eumelanin Biosynthesis |  |  |  |  |  | 2.16 |
| Choline Biosynthesis III |  |  |  |  |  | 2.13 |
