# Supplementary material for: Zebrafish Bone and General Physiology Are Differently Affected by Hormones or Changes in Gravity
Source: PLoS One. 2015 Jun 10;10(6):e0126928. doi: 10.1371/journal.pone.0126928 (PMC4465622; doi:10.1371/journal.pone.0126928)
Supplement: S19 Table — The corresponding—Log(p-value) obtained in IPA analysis was used for classification and are coded by underlining: red means >4, orange between 1 and 3, and yellow means <1. (DOCX) [file pone.0126928.s026.docx]

| Diseases and Bio Functions | 3g | 3g>axe | 3g>1g | 1g>3g | PTH | VitD3 |
| --- | --- | --- | --- | --- | --- | --- |
| proliferation of cells | 10.24 | 4.95 | 8.24 | 3.30 |  |  |
| apoptosis | 5.95 | 5.48 | 9.83 | 3.64 | 2.36 |  |
| cell death of tumor cell lines | 4.41 | 8.22 | 5.51 | 3.76 | 2.86 |  |
| organismal death | 8.09 | 3.91 | 5.73 | 4.14 |  |  |
| cell death | 6.20 | 4.18 | 7.15 | 3.77 | 2.46 |  |
| necrosis | 5.38 | 5.71 | 6.78 | 3.00 | 2.45 |  |
| apoptosis of tumor cell lines | 3.27 | 7.03 | 5.58 | 4.32 | 1.56 |  |
| differentiation of cells | 3.53 | 3.72 | 5.66 | 5.12 |  |  |
| proliferation of tumor cell lines | 4.86 | 3.71 | 4.82 | 4.39 |  |  |
| morphology of cells | 3.75 | 3.47 | 7.15 | 2.69 | 2.11 |  |
| colony formation of tumor cell lines | 2.51 | 3.78 | 5.17 | 5.56 |  |  |
| necrosis of epithelial tissue | 6.01 | 2.49 | 6.24 | 2.14 |  |  |
| development of body trunk | 2.31 | 4.23 | 6.61 | 3.59 |  |  |
| proliferation of connective tissue cells | 4.91 | 2.53 | 5.60 | 3.46 |  |  |
| proliferation of fibroblasts | 7.31 | 2.20 | 3.80 | 2.96 |  |  |
| transcription of RNA | 3.07 | 2.39 | 6.57 | 4.14 |  |  |
| differentiation of tumor cell lines | 3.15 | 2.36 | 4.93 | 5.45 |  |  |
| cell cycle progression | 9.94 |  | 3.40 | 2.45 |  |  |
| colony formation of cells | 2.98 | 2.44 | 4.52 | 5.78 |  |  |
| cell death of epithelial cells | 6.75 | 2.79 | 6.13 |  | 1.61 |  |
| quantity of cells | 2.43 | 3.50 | 5.60 | 3.70 |  | 1.78 |
| apoptosis of epithelial cells | 2.41 | 2.76 | 6.76 | 2.50 | 1.53 |  |
| organization of cytoplasm | 3.89 | 5.38 | 4.74 |  | 1.80 |  |
| expression of RNA |  | 2.58 | 7.16 | 4.20 | 1.84 |  |
| binding of DNA |  | 3.51 | 5.83 | 4.08 |  |  |
| cell movement of tumor cell lines | 4.58 | 3.61 | 5.14 |  |  |  |
| transactivation | 3.35 | 3.35 | 6.53 |  |  |  |
| differentiation of embryonic tissue |  | 7.16 | 3.74 | 2.28 |  |  |
| cell movement | 2.59 | 2.37 | 5.74 | 2.46 |  |  |
| interphase | 5.13 | 4.44 | 3.35 |  |  |  |
| cell death of central nervous system cells | 2.37 | 6.14 | 4.26 |  |  |  |
| apoptosis of cervical cancer cell lines | 2.65 | 3.56 | 3.82 | 2.66 |  |  |
| differentiation of epithelial cells |  | 5.25 | 4.51 | 2.39 |  |  |
| activation of DNA endogenous promoter |  | 2.73 | 5.31 | 4.10 |  |  |
| cell survival | 3.22 | 2.79 | 2.92 | 3.14 |  |  |
| colony formation | 4.20 | 2.61 | 5.23 |  |  |  |
| organization of cytoskeleton | 3.12 | 4.36 | 4.48 |  | 1.53 |  |
| mitosis | 6.84 |  | 2.73 | 2.21 |  |  |
| cell death of cervical cancer cell lines | 3.03 | 3.29 | 2.73 | 2.45 |  |  |
| transactivation of RNA | 2.89 | 2.73 | 5.81 |  |  |  |
| transcription of DNA |  | 2.51 | 5.25 | 3.52 | 1.55 |  |
| transport of molecule | 2.63 | 2.82 | 2.91 | 2.80 |  | 3.33 |
| cell viability | 3.48 | 2.66 | 2.56 | 2.43 |  |  |
| arrest in interphase | 4.63 | 3.65 | 2.85 |  |  |  |
| metabolism of protein | 2.53 | 2.89 | 3.58 | 2.09 |  | 2.36 |
| invasion of cells | 2.60 |  | 5.12 | 3.36 |  |  |
| G1 phase | 3.46 | 3.95 | 3.66 |  |  |  |
| microtubule dynamics | 2.56 | 3.89 | 4.41 |  |  |  |
| concentration of aldosterone |  | 2.21 | 4.34 | 4.28 |  |  |
| formation of cells | 3.58 | 3.06 | 4.14 |  |  |  |
| concentration of lipid | 3.28 | 2.22 | 2.58 | 2.52 |  | 11.41 |
| differentiation of blood cells |  | 2.21 | 5.04 | 3.34 |  |  |
| development of cardiovascular system |  | 2.81 | 5.43 | 2.32 |  | 2.04 |
| apoptosis of prostate cancer cell lines |  | 2.29 | 6.03 | 2.10 |  |  |
| quantity of blood cells |  | 3.48 | 4.16 | 2.57 |  | 2.19 |
| eye development |  | 5.90 | 4.24 |  |  |  |
| migration of tumor cell lines | 3.97 | 2.53 | 3.55 |  |  | 2.57 |
| migration of cells |  | 2.57 | 4.73 | 2.73 |  |  |
| quantity of K+ |  |  | 3.52 | 6.42 |  | 2.43 |
| abnormal morphology of digestive system |  | 3.79 | 3.60 | 2.45 |  |  |
| development of body axis |  | 3.49 | 6.30 |  |  |  |
| development of neurons | 2.63 | 2.47 | 4.62 |  |  |  |
| binding of DNA fragment |  | 2.63 | 4.59 | 2.49 |  |  |
| apoptosis of beta islet cells | 2.58 |  | 3.39 | 3.64 |  |  |
| cell viability of epithelial cell lines | 3.18 |  | 2.47 | 3.93 |  |  |
| quantity of filaments | 2.91 | 3.83 |  | 2.80 |  |  |
| development of sensory organ |  | 5.16 | 4.36 |  |  |  |
| abnormal morphology of body cavity |  | 2.97 | 4.38 | 2.14 | 2.25 |  |
| abnormal morphology of abdomen |  | 2.63 | 3.58 | 3.28 |  | 2.94 |
| size of body |  | 2.60 | 4.58 | 2.17 |  |  |
| proliferation of muscle cells | 2.37 |  | 4.57 | 2.26 |  | 1.94 |
| proliferation of liver cells |  | 2.77 | 3.86 | 2.55 |  |  |
| differentiation of keratinocytes |  | 2.73 | 3.15 | 3.27 |  |  |
| cell death of breast cancer cell lines | 3.37 | 2.93 | 2.73 |  |  |  |
| growth of epithelial tissue |  | 2.10 | 4.68 | 2.13 |  |  |
| arrest in G0/G1 phase transition | 2.61 | 2.81 | 3.44 |  |  |  |
| abnormal morphology of eye |  | 4.36 | 4.48 |  |  |  |
| cell death of bone marrow cells | 3.76 | 2.59 |  | 2.45 |  |  |
| behavior |  | 3.44 | 5.26 |  |  |  |
| abnormal morphology of cells |  | 2.12 | 4.37 | 2.20 |  |  |
| cell death of connective tissue cells | 2.72 |  | 2.60 | 3.36 |  |  |
| development of head |  | 3.24 | 5.22 |  |  |  |
| uptake of D-glucose | 3.28 | 2.36 |  | 2.75 |  | 5.79 |
| apoptosis of glomerular cells | 5.00 |  | 3.37 |  |  |  |
| vasculogenesis |  | 2.15 | 3.71 | 2.48 |  | 2.08 |
| G1/S phase transition | 2.73 | 2.59 | 3.02 |  |  |  |
| cell death of pancreatic cancer cell lines | 2.73 |  | 2.50 | 3.08 |  |  |
| cell death of kidney cells | 5.59 |  | 2.64 |  |  |  |
| differentiation of connective tissue cells | 2.45 | 2.21 | 3.53 |  |  |  |
| differentiation of leukocytes |  | 2.11 | 2.69 | 3.36 |  |  |
| development of muscle |  |  | 5.65 | 2.51 |  | 3.41 |
| apoptosis of kidney cells | 4.49 |  | 3.58 |  |  |  |
| morphology of head |  | 5.39 | 2.57 |  | 1.69 |  |
| development of epithelial tissue |  |  | 5.20 | 2.54 |  |  |
| cell death of brain cells |  | 4.27 | 3.46 |  |  |  |
| development of lymphatic system component |  | 2.93 | 4.65 |  |  |  |
| quantity of hematopoietic progenitor cells |  |  | 5.20 | 2.38 |  |  |
| myogenesis |  |  | 5.31 | 2.26 |  |  |
| differentiation of central nervous system cells |  | 4.29 | 3.19 |  |  |  |
| abnormal morphology of head |  | 4.77 | 2.58 |  |  |  |
| development of abdomen |  |  | 3.85 | 3.48 |  |  |
| metabolism of amino acids | 4.37 |  | 2.74 |  |  | 8.82 |
| differentiation of connective tissue | 2.40 |  | 4.68 |  |  |  |
| binding of synthetic promoter |  | 2.11 | 4.94 |  |  | 2.33 |
| development of blood vessel |  | 2.11 | 4.90 |  |  | 2.18 |
| differentiation of neural precursor cells |  | 4.10 | 2.91 |  |  |  |
| development of digestive system |  | 2.72 | 4.20 |  |  |  |
| quantity of leukocytes |  | 3.92 | 3.00 |  |  |  |
| growth of organism | 3.01 |  | 3.88 |  |  |  |
| fatty acid metabolism | 3.62 | 3.24 |  |  |  | 7.62 |
| apoptosis of central nervous system cells |  | 4.05 | 2.78 |  |  |  |
| differentiation of brain cells |  | 3.66 | 3.15 |  |  |  |
| quantity of centrosome | 4.11 |  | 2.65 |  |  |  |
| proliferation of colon cancer cell lines |  |  | 4.27 | 2.49 |  |  |
| apoptosis of epithelial cell lines | 3.76 |  | 2.99 |  |  |  |
| pluripotency of cells |  |  |  | 6.62 |  |  |
| concentration of cholesterol | 2.69 | 3.92 |  |  |  | 6.28 |
| differentiation of astrocytes |  | 2.92 | 3.65 |  |  |  |
| cell viability of fibroblast cell lines |  |  | 4.00 | 2.48 |  |  |
| morphology of vessel |  | 3.60 | 2.85 |  | 1.55 |  |
| development of muscle cells | 2.98 |  | 3.44 |  |  | 2.17 |
| expression of DNA |  |  | 6.40 |  | 1.61 |  |
| morphology of digestive system |  |  | 3.74 | 2.65 |  | 1.82 |
| differentiation of skin |  |  | 2.87 | 3.50 |  |  |
| metabolism of thymocytes | 3.28 |  | 3.06 |  |  |  |
| immortalization |  |  | 3.83 | 2.49 |  |  |
| abnormal morphology of olfactory placode |  | 3.69 | 2.59 |  |  |  |
| arrest in G2/M phase of bone marrow cells |  | 3.69 | 2.59 |  |  |  |
| fate determination of hair cells |  | 3.69 | 2.59 |  |  |  |
| apoptosis of leukocyte cell lines |  |  | 4.21 | 2.07 |  |  |
| development of thymus gland |  | 2.68 | 3.59 |  |  |  |
| degradation of amino acids | 3.12 |  | 3.15 |  |  | 3.85 |
| repression of RNA |  |  | 3.76 | 2.51 |  |  |
| morphogenesis of ventricular septum |  |  | 3.32 | 2.94 |  |  |
| metabolism of nucleoside triphosphate | 2.87 | 3.39 |  |  |  |  |
| abnormal morphology of retina |  | 2.35 | 3.90 |  |  |  |
| contraction of aortic ring tissue |  | 3.93 |  | 2.29 |  |  |
| skin development |  |  | 3.18 | 3.03 |  |  |
| arrest in cell cycle progression of keratinocytes |  |  | 2.59 | 3.61 |  |  |
| development of neuroglia | 3.08 |  | 3.11 |  |  |  |
| abnormal morphology of hepatobiliary system |  | 2.47 | 3.69 |  |  |  |
| cell viability of breast cancer cell lines | 3.50 | 2.66 |  |  |  |  |
| fate determination of cells |  | 2.63 | 3.52 |  |  |  |
| abnormal morphology of epithelial tissue |  | 2.38 | 3.74 |  |  |  |
| assembly of protein-protein complex |  | 3.08 | 2.98 |  |  |  |
| differentiation of embryonic cells |  | 3.53 | 2.53 |  |  |  |
| neuronal cell death |  | 3.18 | 2.88 |  |  |  |
| arrest in growth of fibroblast cell lines |  |  | 3.52 | 2.52 |  |  |
| proliferation of smooth muscle cells |  | 2.31 | 3.72 |  |  |  |
| cytokinesis | 3.02 |  | 3.00 |  |  |  |
| senescence of fibroblast cell lines | 3.39 |  | 2.61 |  |  |  |
| perinatal death |  | 3.16 | 2.83 |  |  |  |
| binding of gene | 2.36 | 3.63 |  |  |  |  |
| G2 phase | 3.42 | 2.54 |  |  |  |  |
| checkpoint control | 5.95 |  |  |  |  |  |
| formation of hair cells |  | 2.85 | 3.09 |  |  |  |
| size of animal |  | 2.85 | 3.04 |  |  |  |
| proliferation of bone marrow cells | 3.10 | 2.76 |  |  |  |  |
| accumulation of cells |  | 2.15 | 3.69 |  |  |  |
| growth of lymphatic system component | 3.64 | 2.20 |  |  |  |  |
| G1 phase of tumor cell lines |  | 2.52 | 3.32 |  |  |  |
| growth of embryonic tissue |  | 3.33 | 2.49 |  |  |  |
| synthesis of rRNA |  |  | 5.80 |  |  |  |
| development of oligodendrocytes |  | 2.49 | 3.23 |  |  |  |
| necrosis of kidney | 5.70 |  |  |  |  |  |
| apoptosis of leukemia cell lines |  | 2.30 | 3.29 |  |  |  |
| uptake of monosaccharide | 3.17 | 2.41 |  |  |  | 5.87 |
| differentiation of lymphocytes |  |  | 3.04 | 2.49 |  |  |
| interphase of epithelial cells |  |  | 3.16 | 2.34 |  |  |
| apoptosis of B-lymphocyte derived cell lines |  |  | 3.32 | 2.17 |  |  |
| migration of smooth muscle cells | 2.44 |  | 3.05 |  |  |  |
| quantity of steroid hormone |  |  | 3.02 | 2.46 |  | 2.15 |
| adipogenesis of mesenchymal cells | 2.30 | 3.17 |  |  |  |  |
| oxygenation | 2.30 | 3.17 |  |  |  |  |
| synthesis of DNA | 3.25 |  |  | 2.21 |  |  |
| catabolism of amino acids | 2.65 |  | 2.77 |  |  | 3.25 |
| cell death of carcinoma cell lines | 3.27 |  |  | 2.15 |  |  |
| metabolism of carbohydrate | 2.58 |  |  | 2.82 |  | 6.67 |
| interphase of tumor cell lines |  | 2.13 | 3.25 |  |  |  |
| differentiation of hematopoietic cells |  |  | 3.05 | 2.33 |  |  |
| quantity of carbohydrate |  | 2.19 |  | 3.18 |  | 8.54 |
| morphology of eye |  |  | 5.34 |  |  |  |
| contraction of heart | 3.06 |  |  | 2.23 |  | 2.01 |
| arrest in G2 phase | 3.04 |  |  | 2.22 |  |  |
| growth of connective tissue |  |  | 5.25 |  |  |  |
| heart rate | 3.10 |  |  | 2.13 |  | 1.80 |
| M phase | 5.16 |  |  |  |  |  |
| arrest in mitosis | 5.14 |  |  |  |  |  |
| proliferation of neuronal cells |  | 5.06 |  |  | 2.06 |  |
| cell cycle progression of epidermal cells |  |  | 5.06 |  |  |  |
| systolic pressure | 5.01 |  |  |  |  |  |
| arrest in metaphase | 4.99 |  |  |  |  |  |
| catabolism of neutral amino acid |  |  | 4.97 |  |  |  |
| apoptosis of breast cell lines |  |  | 4.97 |  |  |  |
| quantity of steroid | 2.45 | 2.51 |  |  |  | 8.04 |
| vasoconstriction of afferent arterioles |  | 4.96 |  |  |  |  |
| secretion of lipid |  | 2.17 | 2.57 |  |  | 3.13 |
| concentration of acylglycerol |  |  | 2.56 | 2.05 |  | 6.07 |
| cellular homeostasis |  |  | 4.60 |  |  | 2.82 |
| morphology of cardiovascular system |  |  | 4.25 |  |  | 2.30 |
| influx of cholesterol |  |  |  | 4.23 |  | 2.68 |
| abnormal morphology of cardiovascular system |  |  | 4.22 |  | 2.35 | 2.13 |
| elongation of mitotic spindle | 3.94 |  |  |  | 1.43 |  |
| beta-oxidation of fatty acid | 3.76 |  |  |  |  | 5.07 |
| cell viability of kidney cell lines | 3.76 |  |  |  |  | 2.16 |
| synthesis of fatty acid |  | 3.70 |  |  |  | 3.40 |
| proliferation of lung cancer cell lines |  |  |  | 3.57 |  | 1.95 |
| differentiation of trophoblast |  | 3.48 |  |  | 1.49 |  |
| contraction of mesangial cells |  | 3.39 |  |  | 1.51 |  |
| kidney development |  |  |  | 3.33 |  | 2.13 |
| beta-oxidation of docosahexaenoic acid | 3.28 |  |  |  |  | 2.91 |
| beta-oxidation of tetracosahexaenoic acid | 3.28 |  |  |  |  | 2.91 |
| leukocyte migration |  |  | 3.24 |  | 1.64 |  |
| uptake of cholesterol ester |  |  | 3.23 |  |  | 2.13 |
| metabolism of membrane lipid derivative | 3.22 |  |  |  |  | 3.91 |
| concentration of sterol | 3.20 |  |  |  |  | 7.55 |
| adipogenesis |  |  | 3.15 |  | 1.78 |  |
| concentration of D-glucose |  |  |  | 3.09 |  | 7.08 |
| metabolism of glutamine family amino acid | 3.07 |  |  |  |  | 1.99 |
| muscle contraction |  |  | 2.99 |  |  | 3.01 |
| metabolism of vitamin |  |  |  | 2.74 |  | 3.67 |
| arrest in interphase of epithelial cell lines |  |  |  | 2.74 |  | 3.09 |
| concentration of triacylglycerol |  |  | 2.73 |  |  | 5.85 |
| uptake of carbohydrate |  | 2.73 |  |  |  | 5.93 |
| quantity of amino acids | 2.67 |  |  |  |  | 4.19 |
| oxidation of polyunsaturated fatty acids | 2.65 |  |  |  |  | 3.11 |
| efflux of cholesterol |  |  |  | 2.64 |  | 4.63 |
| abnormal quantity of lipid |  | 2.63 |  |  |  | 6.91 |
| gluconeogenesis | 2.51 |  |  |  |  | 6.33 |
| synthesis of lipid | 2.50 |  |  |  |  | 6.40 |
| metabolism of sterol | 2.45 |  |  |  |  | 3.91 |
| synthesis of amino acids |  |  |  | 2.42 |  | 3.78 |
| quantity of vldl triglyceride in blood |  |  |  | 2.37 |  | 5.25 |
| glucose tolerance |  |  |  | 2.29 |  | 4.16 |
| metabolism of terpenoid |  |  |  |  |  | 6.59 |
| oxidation of fatty acid |  |  |  |  |  | 6.47 |
| oxidation of lipid |  |  |  |  |  | 6.39 |
| synthesis of bile acid |  |  |  |  | 1.63 | 3.98 |
| steroid metabolism |  |  |  |  |  | 5.59 |
| quantity of vitamin |  |  |  |  |  | 5.54 |
| concentration of fatty acid |  |  |  |  |  | 5.30 |
| homeostasis of lipid |  |  |  |  |  | 4.91 |
